# Active Ester Functionalized Azobenzenes as Versatile Building Blocks

**DOI:** 10.3390/molecules26133916

**Published:** 2021-06-26

**Authors:** Sven Schultzke, Melanie Walther, Anne Staubitz

**Affiliations:** 1 Institute for Analytical and Organic Chemistry, University of Bremen, Leobener Straße 7, D-28359 Bremen, Germany; schultzke@uni-bremen.de (S.S.); melanie.walther@uni-bremen.de (M.W.); 2 MAPEX Center for Materials and Processes, University of Bremen, Bibliothekstraße 1, D-28359 Bremen, Germany

**Keywords:** azobenzene, diazocine, molecular switches, palladium-catalyzed carbonylation, photo-switchable NHS ester, *N*-hydroxysuccinimide, chemical biology

## Abstract

Azobenzenes are important molecular switches that can still be difficult to functionalize selectively. A high yielding Pd-catalyzed cross-coupling method under mild conditions for the introduction of NHS esters to azobenzenes and diazocines has been established. Yields were consistently high with very few exceptions. The NHS functionalized azobenzenes react with primary amines quantitatively. These amines are ubiquitous in biological systems and in material science.

## 1. Introduction

Photoswitchable molecules add photoresponsiveness to diverse materials such as polymers [1,2,3,4,5,6], dendrimers [7,8] or biomolecules [9,10,11,12,13,14,15,16,17,18,19,20,21,22,23]. By precise adjustment of the synthetic strategy the switch can be incorporated into the target material and its switching properties can be utilized. However, in many cases, it is exactly this tailored functionalization of the photoswitch that is the major challenge and the bottleneck in a successful project.

**Figure 1:**
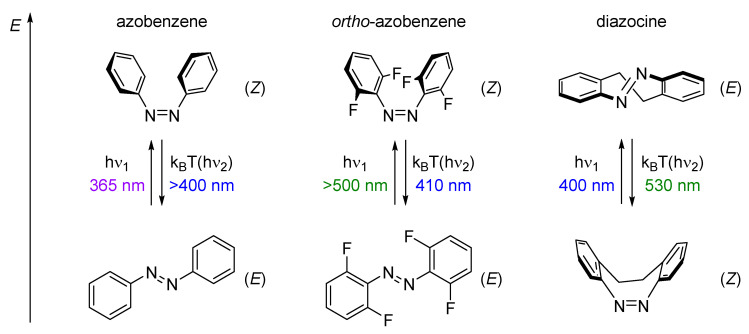
Isomerization of azobenzene [24], tetra-ortho-fluorinated azobenzene [48] and diazocine [51].

Due to its fast and efficient photoswitching characteristics, as well as its high fatigue-resistance [24], azobenzene is widely used in versatile applications such as molecular machines [3,25,26,27,28], data storage materials [29,30,31,32], or photopharmacology [9,10,11,12,13,14,15,16,17,18,19,20,21,22,23]. Upon irradiation with UV-light, typical azobenzenes undergo isomerization from the thermodynamically favored (*E*)-isomer to the less stable (*Z*)-isomer (Figure 1, left). The re-isomerization can occur either by irradiation with light or by thermal relaxation [24,33]. The half-lives of the latter strongly depend on the azobenzenes’ substitution pattern and are therefore tunable for the specific application [34]. The isomerization is accompanied by a change of several physicochemical characteristics: the geometry [35,36], the end-to end-distance [35,36], and the electronic properties [37] (e.g., absorption and dipole moment). The thermodynamically favored (*E*)-form is planar [35] and without a dipole moment [37]. The (*Z*)-isomer on the other hand has a bent geometry where the phenyl rings are twisted ~55° out of plane to the azo group [36]. Moreover, the (*Z*)-isomer shows a dipole moment of 3.0 Debye [37]. The end-to-end distance of the (*E*)- and (*Z*)-isomer differs by ~3.5 Å [35,36]. In biological systems, azobenzenes bind typically in an elongated confirmation; its biological activity is controllable via the switching process [9]. The isomerization to the (*Z*)-isomer occurs by irradiation with UV-light, which might harm cells and tissues [38,39,40]. Therefore, a red-shifting of the absorption wavelength is desirable and in biological applications may be even necessary [9]. In addition, a separation of the absorption spectra of both isomers is important to avoid mixtures of isomers. Starting from 2009, *ortho*-substituted azobenzenes [41,42,43,44,45,46,47,48,49,50] and ethylene-bridged azobenzenes, the so-called diazocines [51,52], have been discovered to be promising candidates to overcome the two limitations of a typical azobenzene. Depending on the substituents in *ortho*-position, not only the absorption is shifted towards longer wavelength but also the thermal half-life may be drastically extended due to steric and electronic interactions [48]. Diazocines are also a particularly important class of azobenzenes, because the bent (*Z*)-isomer is thermodynamically favored due to the ring strain of the eight-membered ring (Figure 1, right). The isomerization of diazocines is in both directions possible using visible light; the isomerization to the elongated (*E*)-form occurs upon irradiation with light in the range of 400 nm, the re-isomerization to (*Z*) with 530 nm [51].

To incorporate such azobenzenes into a larger system, a suitable functional group is necessary that reacts selectively with the specific target position of the material. *N*-Hydroxysuccinimide (NHS) esters react selectively under mild conditions with primary amines, which can be found for example in commercially available polymers but of course also in all proteins and peptides as well as in nucleic acids. Due to the ubiquity of primary amines, NHS esters are versatile building blocks for instance in peptide synthesis [53,54,55], bioconjugate chemistry [56,57,58,59,60] as well as functionalized materials and polymers [61,62,63,64,65,66,67,68,69,70,71]. The use of NHS esters for the formation of amides has several advantages: NHS esters are comparatively easy to prepare. They are the only active esters that can be isolated [72]. In comparison to the coupling reaction of a primary amine with a carboxylic acid using a carbodiimide coupling agent such as *N,N′*-dicyclohexylcarbodiimide (DCC), NHS esters are less toxic. They can be used under physiologic or slightly basic conditions [72]. Moreover, the products of their transformations are easier to purify as water-soluble NHS is released during the amide formation [53].

Traditionally, NHS esters are synthesized by a coupling reaction of the carboxylic acid with NHS in the presence of a carbodiimide coupling agent [73,74], which is known to have an allergenic potential and to form urea as byproduct. Thus, the product purification might be challenging [72]. The carboxylic acid can also be activated by using anhydrides and their analogues for example *N,N′*-disuccinimidyl carbonate (DSC) [75]. More recently, coupling reactions of alcohols [76] and aldehydes [77] with NHS under oxidizing conditions have been investigated. Carbonylative cross-coupling reactions represents another synthetic strategy towards NHS esters [78,79]. Until recently, a carbon monoxide atmosphere was needed for this type of reaction prohibiting a safe and easy-to-handle implementation of the reaction. By using NHS formate as CO surrogate this drawback could be avoided and an efficient protocol on Pd-catalyzed carbonylation reactions under mild conditions developed [78].

**Scheme 1:**
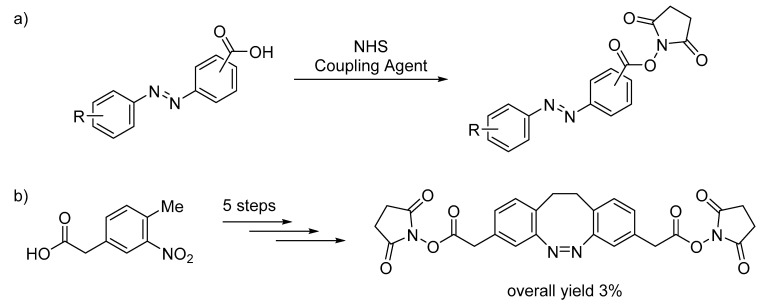
Synthetic approach towards NHS functionalized azobenzenes [80] (**a**) and diazocine [93] (**b**).

The synthesis of NHS functionalized azobenzenes has been, so far, mainly performed via the traditional coupling approach of the corresponding carboxylic acid with NHS and a carbodiimide coupling agent [80,81,82,83,84] (Scheme 1a). Unsymmetric azobenzenes with a carboxylic acid moiety can be obtained via Mills reaction [85,86] or azo coupling [68,81,82,83,84], symmetric ones via a reductive coupling of nitrobenzoic acid [70,84,87,88,89]. Only a few examples exist with the NHS ester in *meta*-position and even fewer are reported for the *ortho*-position [80]. However, NHS functionalized azobenzenes with other *ortho*-substituents that shift the absorption wavelength in the region of visible light have not been reported so far. In case of the diazocines, their incorporation into materials is often performed via peptide coupling of an amine functionalized diazocine [90,91,92]. Only one example of a diazocine with an NHS ester in *meta*-position to the azo group has been reported (Scheme 1b) [93]. Here, the final diazocine was obtained in an overall yield of 3% after five steps. The carbonyl function was connected to the phenyl rings of the diazocine with a methylene bridge allowing more rotational degrees of freedom compared to a direct linkage [93].

We could recently show that cross-coupling reactions are a versatile tool for late-stage functionalization of *para*-substituted azobenzene derivatives [94]. For diazocines, only few examples on cross-coupling reactions with differing yields have been reported, mainly Buchwald-Hartwig cross-coupling [95] or Stille reactions [11,96].

We report an efficient Pd-catalyzed carbonylation protocol, based on previously work of Barré et al. [78], to transform iodinated azobenzenes into the corresponding active NHS ester under mild conditions with NHS formate as CO surrogate. The iodinated precursors are easily accessible and a high tolerance of functional groups was achieved, including other halogen substituents. The usefulness of the obtained NHS functionalized azobenzenes was demonstrated in two examples by coupling the NHS esters with an amino acid.

## 2. Results

**Scheme 2:**
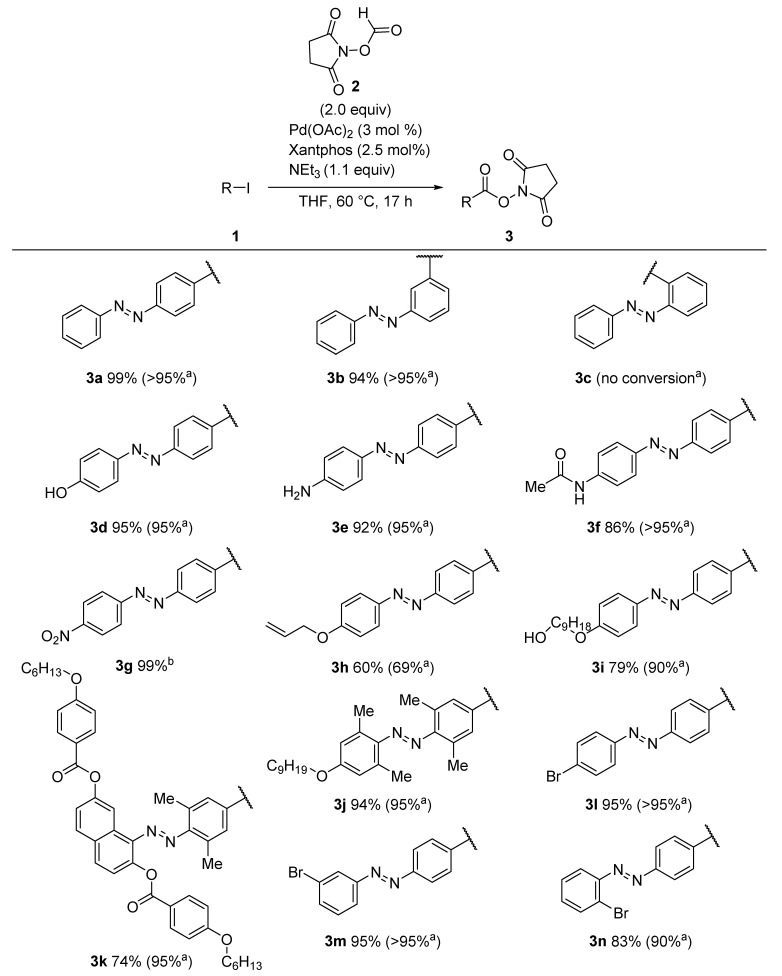
Scope of the Pd-catalyzed NHS functionalization of mono-iodinated azobenzenes. ^a^ Yields in brackets were determined by using 1,3,5-trimethoxybenzene as internal reference for ^1^H NMR analysis; all other yields are isolated yields. ^b^ In DMSO.

The combination of palladium acetate as catalyst and Xantphos as ligand was demonstrated to be a high yielding synthetic method in the literature for Pd-catalyzed carbonylation reactions [78,79]. Therefore, we started our investigation with this catalytic system for the functionalization of mono-iodinated azobenzenes **1** (Scheme 2). Although other possible ligands were screened, the combination of palladium acetate with Xantphos was shown to be the best condition for the catalytic carbonylation of iodinated azobenzenes. In this way, mono-iodinated azobenzenes, at first without further substituents were reacted with NHS formate (**2**). ^1^H NMR analysis with an internal reference revealed a quantitative conversion to the desired *para*- **3a** and *meta*-derivative **3b**. Unfortunately, no conversion to the *ortho*-functionalized azobenzene **3c** was detected; and the starting material was completely recovered. It is very common that the functionalization of azobenzenes by cross-coupling is low yielding or entirely unsuccessful. It also aligns with our own findings that cross-coupling reactions at *ortho*-position of azobenzenes are rarely possible [94]. Next, azobenzene derivatives with further substitutions were tested (**3d**–**3n**). A wide range of functional groups was tolerated: Starting with a hydroxy group in *para*-position, **3d** was obtained in an isolated yield of 95%. Also, an amine function on the azobenzene did not cause problems even though it might have been possible that the formed NHS ester directly reacts with this aromatic primary amine; **3e** could be isolated in 92%. The acetamide functionalized azobenzene **3f** was isolated in a yield of 86%. However, the conversion was determined by ^1^H NMR analysis to be over 95%. Moreover, azobenzene derivatives containing an electron withdrawing group, such as 4-nitro-4′-iodoazobenzene (**1g**), were converted to the corresponding NHS ester in quantitative yield. Even the vinylated species **1h** was successfully transformed to **3h** and isolated in a yield of 60%. Moreover, the functionalization of three additional azobenzene derivatives proofs the versatility of the demonstrated reaction: In particular, an azobenzene with both an ether and a primary hydroxyl group (**3i**, 79%), an alkoxylated tetra-*ortho*-azobenzene (**3j**, 94%) and a unique sterically hindered azobenzene with a naphthalene motif (**3k**, 74%) were all successfully synthesized.

The coupling reaction proceeded chemo-selectively in excellent yields when azobenzene derivatives were used that had both, an iodine and a bromine substituent. Thus, the obtained NHS functionalized azobenzene derivatives **3l**–**n** enable potential further functionalization reactions by cross-coupling strategies.

**Scheme 3:**
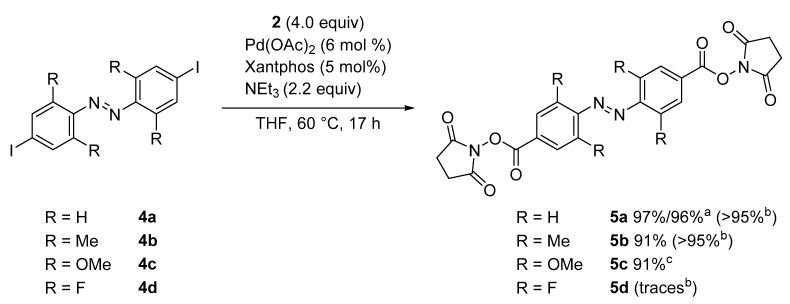
Scope of the Pd-catalyzed NHS functionalization of di-iodinated azobenzenes. ^a^ 2.00 mmol scale. ^b^ Yields in brackets were determined by using 1,3,5-trimethoxybenzene as internal reference for ^1^H NMR analysis; all other yields are isolated yields. ^c^ In DMSO.

Considering the latest research trends to use azobenzene derivatives that can be switched with visible light and to incorporate these into different types of applications, we were interested to transfer our results for mono-iodinated azobenzenes to di-iodinated azobenzenes (Scheme 3). This posed no problems for **5a** and **5b** (Scheme 3), which were synthesized in excellent yields. The synthesis of **5a** was additionally performed on a 2.00 mmol scale leading to a comparable yield as the 100 µmol batch (96%). Here, the reaction setup was slightly modified: A pressure tube was used as a reaction vessel which was closed quickly after the addition of triethylamine. However, difficulties occurred for **5c** and **5d**. For **5c,** we initially observed that the reaction was incomplete. A mixture of mono-substituted NHS ester-azobenzene and di-substituted NHS ester-azobenzene was detected. We hypothesized that the solubility of the iodinated azobenzene **4c** might be too low. Therefore, we changed the solvent to the more polar DMF, as this solvent was reported to lead to comparable yields as THF [78]. However, DMF is slightly basic [97] and thus initializes the decomposition of the NHS formate (**2**). Traces of dimethylamine, a common impurity in DMF [98], can enhance this decomposition. The concomitant release of carbon dioxide increased the pressure inside the vial so much that a subsequent addition of triethyl amine via syringe was difficult. Without the additional base, however, the decomposition of **2** was not sufficient for a full conversion. Changing the solvent to DMSO instead, resolved this problem. The conversion of **4c** increased drastically for the tetra-*ortho*-methoxylated-azobenzene; **5c** was synthesized in a yield of 91%.

**Scheme 4:**
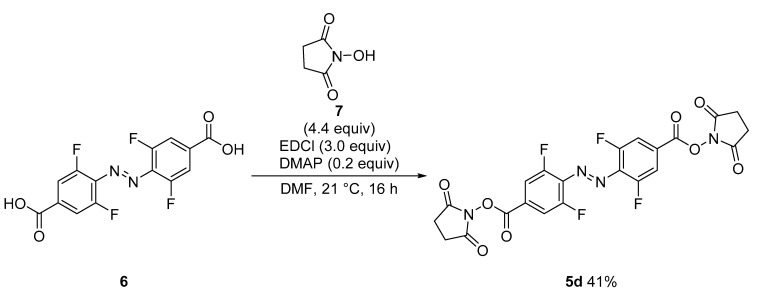
Alternative synthetic route to obtain the NHS functionalized tetra-*ortho*-fluorinated azobenzene.

Unfortunately, the tetra-*ortho*-fluorinated azobenzene **4d** was only converted in traces to **5d** as detected by ^1^H NMR analysis. Even the change to a more polar solvent did not increase the yield. Instead, there were so many reaction products that they could not be further analyzed. In these rare cases, in which the cross-coupling approach fails, alternative strategies need to be developed. In this instance, we were able to synthesize the fluorinated target molecule **5d** by the coupling of the carboxylic acid **6** with NHS (**7**) using 1-ethyl-3-(3-dimethylaminopropyl)carbodiimide (EDCl) and 4-dimethylaminopyridine (DMAP) as coupling agent (Scheme 4). The yield could not be improved by using other coupling agents such as DCC, HATU, HSPyU, 1,1′-carbonyldiimidazol (CDI) or 2,4,6-trichlorobenzoic acid (TCBC).

**Scheme 5:**
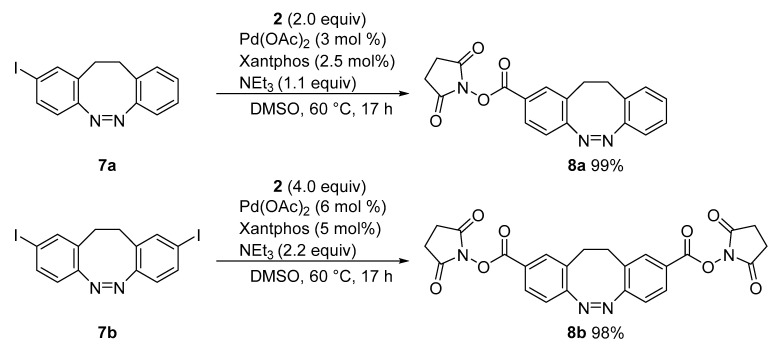
Pd-catalyzed NHS functionalization of iodinated diazocines.

In contrast to typical azobenzenes, the (*Z*)-isomer is thermodynamically favored in diazocines making them a desirable molecule for applications. Moreover, the switching is possible with visible light. Diazocines bearing a carboxyl group are demanding to synthesize with moderate overall yields [99,100]. As iodinated diazocines can be easily obtained [95], we were interested to transfer our method to diazocines. Analogously to the difficulties for di-iodinated azobenzenes, the use of THF as solvent led only to a partial conversion; in case of the di-iodinated diazocine, a mixture of mono- and di-functionalized derivatives was obtained. Again, these problems were resolved by using DMSO as solvent and diazocines **6a** and **6b** were obtained in near-quantitative yields (Scheme 5).

**Scheme 6:**
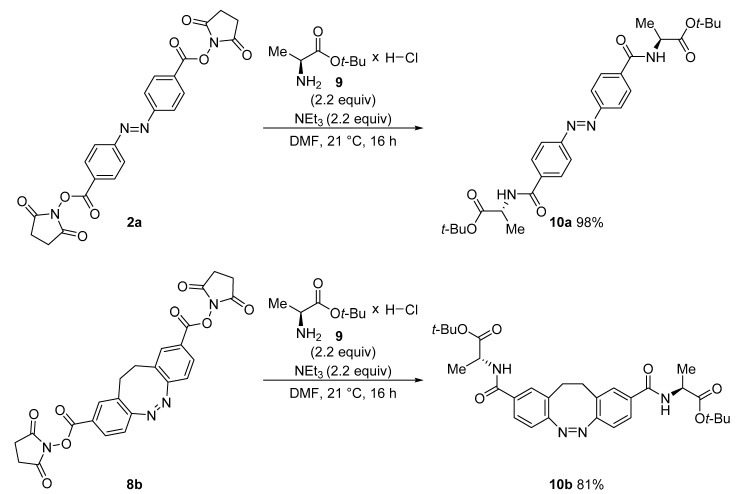
Condensation reaction of NHS derivatives with L-alanine *tert*-butyl ester hydrochloride.

To demonstrate the usefulness of the obtained NHS ester derivatives for reactions with a primary amine, azobenzene **2a** and diazocine **8b** were reacted with l-alanine *tert*-butyl ester hydrochloride under slightly basic conditions yielding the corresponding amino acid derivatives **9a** and **9b** in excellent to quantitative yield (Scheme 6).

## 3. Discussion

This robust and high-yielding protocol enables new possibilities for the molecular design of azobenzene and diazocines derivatives. A large scope of 14 mono-functionalized and 5 di-functionalized azobenzenes and diazocines were prepared in high to excellent yields. Iodinated azobenzenes are fairly easy to synthesize and a vast number of protocols are available. In contrast to this, the traditional synthetic route requires a carboxyl group on the azobenzene for the esterification to yield the NHS functionality. This is a severe synthetic limitation. Indeed, the carboxylated analogous of **3g**, **3k**–**l** have not been reported, but the corresponding NHS derivatives can now be synthesized via our synthetic approach. These advantages are even more important considering that the solubility of di-carboxylated azobenzenes in organic solvents is low. Furthermore, the synthesis of more complex azobenzenes with a carboxyl-functionality requires many synthetical steps or are unknown until today. The practicality of the approach was shown by the condensation of two di-functionalized NHS esters with a protected amino acid.

## 4. Materials and Methods

All synthetic procedures and characterization data for all compounds and precursors can be found in the supporting information.

### 4.1. General Information

For reactions under inert conditions, a nitrogen filled glovebox (Pure Lab^HE^ from Inert, Amesbury, MA USA) and standard Schlenk techniques were used (Supplementary Materials). All carbonylation reactions were performed in microwave reaction vials sealed with a septum cap from Biotage (Biotage, Uppsala, Sweden). All glassware was dried in an oven at 200 °C for several hours prior to use. NMR tubes were dried in an oven at 110 °C for several hours prior to use. NMR spectra were recorded on a Bruker Avance Neo 600 (Bruker BioSpin, Rheinstetten, Germany) (600 MHz (^1^H), 151 MHz (^13^C{^1^H}), 565 MHz (^19^F) at 298 K. All ^1^H NMR and ^13^C{^1^H} NMR spectra were referenced to the residual proton signals of the solvent (^1^H) or the solvent itself (^13^C{^1^H}). ^19^F NMR spectra were referenced internally against trichlorofluoromethane. The exact assignment of the peaks was performed by two-dimensional NMR spectroscopy such as ^1^H COSY, ^13^C{^1^H} HSQC and ^1^H/^13^C{^1^H} HMBC when possible. High-resolution EI mass spectra were recorded on a MAT 95XL double-focusing mass spectrometer from Finnigan MAT (Thermo Fisher Scientific, Waltham, MA, USA) at an ionization energy of 70 eV. Samples were measured by a direct or indirect inlet method with a source temperature of 200 °C. High-resolution ESI and APCI mass spectra were measured by a direct inlet method on an Impact II mass spectrometer from Bruker Daltonics (Bruker Daltonics, Bremen, Germany). ESI mass spectra were recorded in the positive ion collection mode.

IR spectra were recorded on a Nicolet i510 FT-IR spectrometer from Thermo Fisher Scientific (Thermo Fisher Scientific, Waltham, MA, USA) with a diamond window in an area from 500–4000 cm^−1^ with a resolution of 4 cm^−1^. All samples were measured 16 times against a background scan. Melting points were recorded on a Büchi Melting Point M-560 (Büchi, Essen, Germany) and are reported corrected. Thin layer chromatography (TLC) was performed using TLC Silica gel 60 F_254_ from Merck (Merck, Darmstadt, Germany) and compounds were visualized by exposure to UV light at a wavelength of 254 nm. Column chromatography was performed either manually using silica gel 60 (0.015–0.040 mm) from Merck (Merck, Darmstadt, Germany) or by using a PuriFlash 4250 column machine (Interchim, Mannheim, Germany). Silica gel columns of the type CHROMABOND Flash RS 15 SPHERE SiOH 15 µm (Macherey-Nagel, Düren, Germany) were used. The sample was applied via dry load with Celite^®^ 503 (Macherey-Nagel, Düren, Germany) as column material. If stated, Celite^®^ 503 (Macherey-Nagel, Düren, Germany) was used as filtration aid. The use of abbreviations follows the conventions from the ACS Style guide [101].

### 4.2. General Procedures

#### 4.2.1. General Procedure 1 Carbonylation

(a) Mono-iodinated molecular switch

Under inert conditions, the corresponding mono-iodinated molecular switch (200 µmol, 2.00 equiv), Pd(OAc)_2_ (1.35 mg, 6.00 µmol, 3 mol%), Xantphos (2.89 mg, 5.00 µmol, 2.5 mol%) and 2,5-dioxopyrrolidin-1-yl formate (57.2 mg, 400 µmol, 4.00 equiv) were dissolved in dry THF (3 mL) in a pressure reaction vial. A solution of 1,3,5-trimethoxybenzene (16.8 mg, 100 µmol) as an internal standard in dry THF (1 mL) was added. The vial was sealed and heated to 60 °C. A solution of triethyl amine (22.2 mg, 220 µmol, 2.20 equiv) in dry THF (1 mL) was quickly added. Fast gas evolution was observed and the reaction was stirred for 17 h at 60 °C. After cooling to 21 °C, the solvent was removed under reduced pressure. The residue was re-dissolved in DCM (10 mL), filtered through Celite^®^ and the solvent removed under reduced pressure.

(b) Di-iodinated molecular switch

Under inert conditions, the corresponding di-iodinated molecular switch (100 µmol, 1.00 equiv), Pd(OAc)_2_ (1.35 mg, 6.00 µmol, 6 mol%), Xantphos (2.89 mg, 5.00 µmol, 5 mol%) and 2,5-dioxopyrrolidin-1-yl formate (57.2 mg, 400 µmol, 4.00 equiv) were dissolved in dry THF (3 mL) in a pressure reaction vial. A solution of 1,3,5-trimethoxybenzene (16.8 mg, 100 µmol) as an internal standard in dry THF (1 mL) was added. The vial was sealed and heated to 60 °C. A solution of triethyl amine (22.2 mg, 220 µmol, 2.20 equiv) in dry THF (1 mL) was quickly added. Fast gas evolution was observed and the reaction was stirred for 17 h at 60 °C. After cooling to 21 °C, the solvent was removed under reduced pressure. The residue was re-dissolved in DCM (10 mL), filtered through Celite^®^ and the solvent removed under reduced pressure.

#### 4.2.2. General Procedure 2 Carbonylation

(a) Mono-iodinated molecular switch

Under inert conditions, the corresponding mono-iodinated switch (200 µmol, 2.00 equiv), Pd(OAc)_2_ (1.35 mg, 6.00 µmol, 3 mol%), Xantphos (2.89 mg, 5.00 µmol, 2.5 mol%) and 2,5-dioxopyrrolidin-1-yl formate (57.2 mg, 400 µmol, 4.00 equiv) were dissolved in dry THF (4 mL) in a pressure reaction vial. The vial was sealed and heated to 60 °C. A solution of triethyl amine (22.2 mg, 220 µmol, 2.20 equiv) in dry THF (1 mL) was quickly added. Fast gas evolution was observed and the reaction was stirred for 17 h at 60 °C. After cooling to 21 °C, the solvent was removed under reduced pressure. The residue was re-dissolved in DCM (10 mL) and extracted with water (20 mL) and brine (20 mL). The combined organic layers were dried over magnesium sulfate, filtered and the solvent removed under reduced pressure.

(b) Di-iodinated molecular switch

Under inert conditions, the corresponding di-iodinated switch (100 µmol, 1.00 equiv), Pd(OAc)_2_ (1.35 mg, 6.00 µmol, 6 mol%), Xantphos (2.89 mg, 5.00 µmol, 5 mol%) and 2,5-dioxopyrrolidin-1-yl formate (57.2 mg, 400 µmol, 4.00 equiv) were dissolved in dry THF (4 mL) in a pressure reaction vial. The vial was sealed and heated to 60 °C. A solution of triethyl amine (22.2 mg, 220 µmol, 2.20 equiv) in dry THF (1 mL) was quickly added. Fast gas evolution was observed and the reaction was stirred for 17 h at 60 °C. After cooling to 21 °C, the solvent was removed under reduced pressure. The residue was re-dissolved in DCM (10 mL) and extracted with water (20 mL) and brine (20 mL). The combined organic layers were dried over magnesium sulfate, filtered and the solvent removed under reduced pressure.

#### 4.2.3. General Procedure Condensation Reaction

Triethyl amine (2.20 equiv) was added to a solution of L-alanine *tert*-butyl ester hydrochloride (2.20 equiv) in dry DMF. A solution of the corresponding di-NHS functionalized molecular switch (1.00 equiv) in dry DMF was added and the resulting mixture was stirred at 21 °C for 16 h. Water and ethyl acetate were added, and the mixture was washed with brine (3×). The combined organic phases were dried over magnesium sulfate, filtered and the solvent removed under reduced pressure yielding the product.

## Data Availability

The data presented in this study are openly available in the supporting information.

## Supplementary Materials

The following are available online: general information, a list of all reagents and solvents, experimental procedures and analytical data, determination of ^1^H NMR yield, images of all NMR spectra.

## Sample Availability

Samples of the compounds may be available from the authors. Please contact the authors for further details.
