# Supplementary material for: Active Ester Functionalized Azobenzenes as Versatile Building Blocks
Source: Molecules. 2021 Jun 26;26(13):3916. doi: 10.3390/molecules26133916 (PMC8272017; doi:10.3390/molecules26133916)
Supplement: Supplementary file 1 [file molecules-26-03916-s001.zip › molecules-1263038-supplementary.pdf]

Supporting Information

Active Ester Functionalized Azobenzenes as Versatile Building Blocks

Sven Schultzke [1,2,†], Melanie Walther [1,2,†] and Anne Staubitz [1,2,*]

[1] University of Bremen, Institute for Analytical and Organic Chemistry, Leobener Straße 7, D-28359 Bremen, Germany

[2] University of Bremen, MAPEX Center for Materials and Processes, Bibliothekstraße 1, D-28359 Bremen, Germany

* Correspondence: staubitz@uni-bremen.de; Tel.: +49421/21863210

† Both authors contributed equally to this work.

Table of Contents

General Information ... 3

Reagents ... 4

Solvents ... 5

Syntheses ... 6

General procedure 1 ... 6

General procedure 2 ... 6

2,5-Dioxopyrrolidin-1-yl (*E*)-4-(phenyldiazenyl)benzoate[2] (**3a**) ... 7

2,5-Dioxopyrrolidin-1-yl (*E*)-3-(phenyldiazenyl)benzoate[2] (**3b**) ... 7

2,5-Dioxopyrrolidin-1-yl (*E*)-4-((4-hydroxyphenyl)diazenyl)benzoate[3] (**3d**) ... 8

2,5-Dioxopyrrolidin-1-yl (*E*)-4-((4-aminophenyl)diazenyl)benzoate (**3e**) ... 8

2,5-Dioxopyrrolidin-1-yl (*E*)-4-((4-acetamidophenyl)diazenyl)benzoate (**3f**) ... 9

2,5-Dioxopyrrolidin-1-yl (*E*)-4-((4-nitrophenyl)diazenyl)benzoate (**3g**) ... 9

2,5-Dioxopyrrolidin-1-yl (*E*)-4-((4-(allyloxy)phenyl)diazenyl)benzoate (**3h**) ... 10

2,5-Dioxopyrrolidin-1-yl (*E*)-4-((4-((9-hydroxynonyl)oxy)phenyl)diazenyl)benzoate (**3i**) ... 10

2,5-Dioxopyrrolidin-1-yl (*E*)-4-((2,6-dimethyl-4-(nonyloxy)phenyl)diazenyl)-3,5-dimethylbenzoate (**3j**) ... 11

2,5-Dioxopyrrolidin-1-yl (*E*)-4-((2,7-bis(4-(hexyloxy)phenoxy)naphthalen-1-yl)diazenyl)-3,5-dimethylbenzoate (**3k**) 12

2,5-Dioxopyrrolidin-1-yl (*E*)-4-((4-bromophenyl)diazenyl)benzoate (**3l**) 12

2,5-Dioxopyrrolidin-1-yl (*E*)-4-((3-bromophenyl)diazenyl)benzoate (**3m**) 13

2,5-Dioxopyrrolidin-1-yl (*E*)-4-((2-bromophenyl)diazenyl)benzoate (**3n**) 13

Bis(2,5-dioxopyrrolidin-1-yl)4,4´-(diazene-1,2-diyl)(*E*)-dibenzoate[5](**5a**) 14

Bis(2,5-dioxopyrrolidin-1-yl) 4,4'-(diazene-1,2-diyl)(*E*)-bis(3,5-dimethoxybenzoate) (**5b**) 14

Bis(2,5-dioxopyrrolidin-1-yl) 4,4'-(diazene-1,2-diyl)(*E*)-bis(3,5-dimethylbenzoate) (**5c**) 15

Bis(2,5-dioxopyrrolidin-1-yl) 4,4'-(diazene-1,2-diyl)(*E*)-bis(3,5-difluorobenzoate) (**5d**) 15

2,5-Dioxopyrrolidin-1-yl (Z)-11,12-dihydrodibenzo[c,g][1,2]diazocine-2-carboxylate (**8a**) 16

Bis(2,5-dioxopyrrolidin-1-yl) (Z)-11,12-dihydrodibenzo[c,g][1,2]diazocine-2,9-dicarboxylate (**8b**) . 17

Di-*tert*-butyl 2,2'-((4,4'-(diazene-1,2-diyl)bis(benzoyl))bis(azanediyl))(*E*)-dipropionate (**10a**) 17

Di-*tert*-butyl 2,2'-((11,12-dihydrodibenzo[c,g][1,2]diazocine-2,9-dicarbonyl)bis(azane-diyl))(*Z*)-dipropionate (**10b**) 18

Determination of the ^{1}H NMR Yields 19

N-Hydroxysuccinimidyl formate[5] (**2**) 21

(*E*)-1-(4-Iodophenyl)-2-phenyldiazene[6] (**1a**) 21

(*E*)-1-(3-Iodophenyl)-2-phenyldiazene[6] (**1b**) 22

(*E*)-1-(2-Iodophenyl)-2-phenyldiazene[6] (**1c**) 22

(*E*)-4-((4-Iodophenyl)diazenyl)phenol[7] (**1d**) 23

(*E*)-4-((4-Iodophenyl)diazenyl)aniline (**1e**) 23

(*E*)-*N*-(4-((4-Iodophenyl)diazenyl)phenyl)acetamide (**1f**) 24

(*E*)-1-(4-Iodophenyl)-2-(4-nitrophenyl)diazene[8] (**1g**) 24

(*E*)-1-(4-(Allyloxy)phenyl)-2-(4-iodophenyl)diazene[9] (**1h**) 25

(*E*)-9-(4-((4-Iodophenyl)diazenyl)phenoxy)nonan-1-ol (**1i**) 26

(*E*)-4-((4-Iodo-2,6-dimethylphenyl)diazenyl)-3,5-dimethylphenol (**S1**) 26

(*E*)-1-(2,6-Dimethyl-4-(nonyloxy)phenyl)-2-(4-iodo-2,6-dimethylphenyl)diazene (**1j**) 27

(*E*)-1-((4-Iodo-2,6-dimethylphenyl)diazenyl)naphthalene-2,7-diol (**S2**) 27

(*E*)-1-((4-Iodo-2,6-dimethylphenyl)diazenyl)naphthalene-2,7-diyl bis(4-(hexyloxy)benzoate) (**1k**). 28

(*E*)-1-(4-Bromophenyl)-2-(4-iodophenyl)diazene[10] (**1l**) 29

(*E*)-1-(3-Bromophenyl)-2-(4-iodophenyl)diazene (**1m**) 29

(*E*)-1-(2-Bromophenyl)-2-(4-iodophenyl)diazene (**1n**) 30

(*E*)-1,2-Bis(4-iodophenyl)diazene[11] (**4a**) 31

4-Iodo-2,6-dimethylaniline[12] (**S3**) 31

(*E*)-1,2-Bis(4-iodo-2,6-dimethylphenyl)diazene[13] (**4b**) 32

4-Bromo-2,6-dimethoxyaniline[14] (**S4**) 32

(*E*)-1,2-Bis(4-bromo-2,6-dimethoxyphenyl)diazene[15] (**S5**) 33

(*E*)-1,2-Bis(4-iodo-2,6-dimethoxyphenyl)diazene (**4c**) ... 33
4-Iodo-2,6-difluoro-aniline[16] (**S6**) ... 34
(*E*)-1,2-bis(2,6-difluoro-4-iodophenyl)diazene[17] (**4d**) ... 34
4-Bromo-2,6-difluoroaniline[18] (**S7**) ... 35
4-Amino-3,5-difluorobenzonitrile[19] (**S8**) ... 35
4-Amino-3,5-difluorobenzoic acid[20] (**S9**) ... 36
Ethyl 4-amino-3,5-difluorobenzoate[20] (**S10**) ... 36
(*E*)-4,4'-(Diazene-1,2-diyl)bis(3,5-difluorobenzoic acid)[17] (**S11**) ... 37
(*E*)-4,4'-(Diazene-1,2-diyl)bis(3,5-difluorobenzoic acid)[20] (**6**) ... 37
2-(2-(2-Aminophenethyl)phenyl)isoindoline-1,3-dione[21] (**S12**) ... 38
2-(2-Aminophenethyl)-4-iodoaniline[21] (**S13**) ... 39
(*Z*)-2-Iodo-11,12-dihydrodibenzo[c,g][1,2]diazocine[21] (**7a**) ... 39
2,2'-(Ethane-1,2-diyl)bis(4-iodoaniline)[21] (**S14**) ... 40
(*Z*)-2,9-Diiodo-11,12-dihydrodibenzo[c,g][1,2]diazocine[21] (**7b**) ... 40
References ... 41
^{1}H, ^{13}C{^{1}H} and ^{19}F NMR Spectra of the Purified Compounds ... 43
^{1}H NMR Spectra for the Determination of the ^{1}H NMR Yield ... 105

General Information

For reactions under inert conditions, a nitrogen filled glovebox (Pure Lab^{HE} from Inert, Amesbury, MA USA) and standard Schlenk techniques were used.

All carbonylation reactions were performed in microwave reaction vials sealed with a septum cap from Biotage (Biotage, Uppsala, Sweden).

All glassware was dried in an oven at 200 °C for several hours prior to use. NMR tubes were dried in an oven at 110 °C for several hours prior to use.

NMR spectra were recorded on a Bruker Avance Neo 600 (Bruker BioSpin, Rheinstetten, Germany) (600 MHz (^{1}H), 151 MHz (^{13}C{^{1}H}), 565 MHz (^{19}F) at 298 K. All ^{1}H NMR and ^{13}C{^{1}H} NMR spectra were referenced to the residual proton signals of the solvent (^{1}H) or the solvent itself (^{13}C{^{1}H}). ^{19}F NMR spectra were referenced internally against trichlorofluoromethane. The exact assignment of the peaks was performed by two-dimensional NMR spectroscopy such as ^{1}H COSY, ^{13}C{^{1}H} HSQC and ^{1}H/^{13}C{^{1}H} HMBC when possible.

High-resolution EI mass spectra were recorded on a MAT 95XL double-focusing mass spectrometer from Finnigan MAT (Thermo Fisher Scientific, Waltham, MA, USA) at an ionization energy of 70 eV. Samples were measured by a direct or indirect inlet method with a source temperature of 200 °C. High-resolution ESI and APCI mass spectra were measured by a direct inlet method on an Impact II mass spectrometer from Bruker Daltonics (Bruker Daltonics, Bremen, Germany). ESI mass spectra were recorded in the positive ion collection mode.

IR spectra were recorded on a Nicolet i510 FT-IR spectrometer from Thermo Fisher Scientific (Thermo Fisher Scientific, Waltham, MA, USA) with a diamond window in an area from 500 – 4000 cm^{-1} with a resolution of 4 cm^{-1}. All samples were measured 16 times against a background scan.

Melting points were recorded on a Büchi Melting Point M-560 (Büchi, Essen, Germany) and are reported corrected.

Thin layer chromatography (TLC) was performed using TLC Silica gel 60 F_{254} from Merck (Merck, Darmstadt, Germany) and compounds were visualized by exposure to UV light at a wavelength of 254 nm. Column chromatography was performed either manually using silica gel 60 (0.015-0.040 mm) from Merck (Merck, Darmstadt, Germany) or by using a PuriFlash 4250 column machine (Interchim, Mannheim, Germany). Silica gel columns of the type CHROMABOND Flash RS 15 SPHERE SiOH 15 µm (Macherey-Nagel, Düren, Germany) were used. The sample was applied via dry load with Celite® 503 (Macherey-Nagel, Düren, Germany) as column material. If stated, Celite® 503 (Macherey-Nagel, Düren, Germany) was used as filtration aid.

The use of abbreviations follows the conventions from the ACS Style guide[1].

Reagents

All chemicals were commercially available and used without purification unless stated otherwise.

Table S1. List of supplier and purity of used chemicals.

Reagent	Supplier	Purity	Comments
L-Alanine *tert*-butyl ester hydrochloride	Apollo Scientific		stored in a fridge in a glovebox
4-Aminoacetanilide	TCI	>98%	
2-Bromoaniline	Fluorochem	97%	
3-Bromoaniline	TCI	>98.0%	
4-Bromoaniline	Sigma Aldrich	97%	
9-Bromo-1-nonanol	Sigma Aldrich	95%	
N-Bromosuccinimide	Sigma Aldrich	98%	
1,8-Diazabicyclo[5.4.0]-undec-7-ene	Apollo	99.6%	
2,6-Difluoroanilin	chemPur	97%	
2,7-Dihydroxynapthalene	TCI	99%	
2,6-Dimethoxyanilin	BLDPharm	97%	
2,6-Dimethylaniline	TCI	99%	
3,5-Dimethylphenol	Sigma Aldrich	98%	
2,2'-Ethylenedianiline	TCI	>98%	
Hydrazine monohydrate	abcr	98%	
Hydrochloric acid	Sigma Aldrich		fuming, ≥37%
N-Hydroxysuccinimide	Apollo Scientific	>97%	
Iodine	abcr	99%	
4-Iodoaniline	Sigma Aldrich	98%	
N-Iodosuccinimide	Apollo Scientific	98%	
Magnesium sulfate	VWR		dried
Manganese dioxide	Merck		(precipitated active) for synthesis

meta-Chloroperoxybenzoic acid	Acros	70 – 75%[1]	
Nitrosobenzene	TCI	>98.0%	stored in a fridge
Oxone®	abcr		monopersulfate
Palladium acetate	Carbolution	98%	
Phenol	Sigma Aldrich	99%	
Phthalic anhydride	Acros	99%	
Potassium iodide	abcr	99%	
Sodium carbonate	Org Laborchemie	99.9%	
Sodium chloride	Th. Geyer	min 99.0%	
Sodium hydrogen carbonate	VWR	ACS, Reag. Ph. Eur.	
Sodium hydroxide	VWR	98%	
Sodium nitrite	Sigma Aldrich	97%	
Triethyl amine	Fluorochem	anhydrous	degassed, stored in a glovebox
1,3,5-Trimethoxybenzene	Sigma Aldrich	≥99%	
Xantphos	BLD Pharm	98%	

Solvents

All solvents for purification and extraction were used as received. All solvents used for synthesis under inert conditions were dried by a solvent purification system (SPS) from Inert Technologies.

Table S2. List of supplier and purity of used solvents.

**Solvent**	**Supplier**	**Purity**	**Comments**
Acetic Acid	Merck	≥99.8%	
Acetone	Sigma Aldrich	>99.5%	
Acetonitrile	Fisher Scientific	≥99.5%	
Cyclohexane	Merck	≥99.5%	
Chloroform	Sigma Aldrich	>99%	
Chloroform-d_1	Euroisotop	99.8%	
DCM	Merck	≥99.9%	
DMF	Acros Organics	99.8%	Extra dry, AcroSeal™
DMSO	VWR	technical grade	
DMSO	Acros Organics	99.7%	Extra dry, AcroSeal™
DMSO-d_6		>99.00%	
Ethanol	VWR	≥99.8%	absolute
Ethyl acetate	Merck	≥99.5%	
Formic acid	Sigma Aldrich	97%	
Methanol	VWR	≥99.8%	
1-Methyl-2-pyrrolidone	TCI	>99%	Low water content
Tetrahydrofuran	VWR	HPLC grade	anhydrous from SPS, stored in a glovebox
Tetrahydrofuran	Fisher Scientific	≥99.8%	
Toluene	Merck	≥99.7%	
Water		deionized	

[1] The exact concentration was determined by iodometric titration against sodium thiosulfate.

Syntheses

General procedure 1

a) Mono-iodinated molecular switch

Under inert conditions, the corresponding mono-iodinated switch (200 µmol, 2.00 equiv), $Pd(OAc)_2$ (1.35 mg, 6.00 µmol, 3 mol%), Xantphos (2.89 mg, 5.00 µmol, 2.5 mol%) and 2,5-dioxopyrrolidin-1-yl formate (**2**) (57.2 mg, 400 µmol, 4.00 equiv) were dissolved in dry THF (3 mL) in a pressure reaction vial. A solution of 1,3,5-trimethoxybenzene (16.8 mg, 100 µmol) as an internal standard in dry THF (1 mL) was added. The vial was sealed and heated to 60 °C. A solution of triethyl amine (22.2 mg, 220 µmol, 2.20 equiv) in dry THF (1 mL) was quickly added. Fast gas evolution was observed and the reaction was stirred for 17 h at 60 °C. After cooling to 21 °C, the solvent was removed under reduced pressure. The residue was re-dissolved in DCM (10 mL), filtered through Celite® and the solvent removed under reduced pressure.

b) Di-iodinated molecular switch

Under inert conditions, the corresponding di-iodinated molecular switch (100 µmol, 1.00 equiv), $Pd(OAc)_2$ (1.35 mg, 6.00 µmol, 6 mol%), Xantphos (2.89 mg, 5.00 µmol, 5 mol%) and 2,5-dioxopyrrolidin-1-yl formate (**2**) (57.2 mg, 400 µmol, 4.00 equiv) were dissolved in dry THF (3 mL) in a pressure reaction vial. A solution of 1,3,5-trimethoxybenzene (16.8 mg, 100 µmol) as an internal standard in dry THF (1 mL) was added. The vial was sealed and heated to 60 °C. A solution of triethyl amine (22.2 mg, 220 µmol, 2.20 equiv) in dry THF (1 mL) was quickly added. Fast gas evolution was observed and the reaction was stirred for 17 h at 60 °C. After cooling to 21 °C, the solvent was removed under reduced pressure. The residue was re-dissolved in DCM (10 mL), filtered through Celite® and the solvent removed under reduced pressure.

General procedure 2

a) Mono-iodinated molecular switch

Under inert conditions, the corresponding mono-iodinated switch (200 µmol, 2.00 equiv), $Pd(OAc)_2$ (1.35 mg, 6.00 µmol, 3 mol%), Xantphos (2.89 mg, 5.00 µmol, 2.5 mol%) and 2,5-dioxopyrrolidin-1-yl formate (**2**) (57.2 mg, 400 µmol, 4.00 equiv) were dissolved in dry THF (4 mL) in a pressure reaction vial. The vial was sealed and heated to 60 °C. A solution of triethyl amine (22.2 mg, 220 µmol, 2.20 equiv) in dry THF (1 mL) was quickly added. Fast gas evolution was observed and the reaction was stirred for 17 h at 60 °C. After cooling to 21 °C, the solvent was removed under reduced pressure. The residue was re-dissolved in DCM (10 mL) and extracted with water (20 mL) and brine (20 mL). The combined organic layers were dried over $MgSO_4$, filtered and the solvent removed under reduced pressure.

b) Di-iodinated molecular switch

Under inert conditions, the corresponding di-iodinated switch (100 µmol, 1.00 equiv), $Pd(OAc)_2$ (1.35 mg, 6.00 µmol, 6 mol%), Xantphos (2.89 mg, 5.00 µmol, 5 mol%) and 2,5-dioxopyrrolidin-1-yl formate (**2**) (57.2 mg, 400 µmol, 4.00 equiv) were dissolved in dry THF (4 mL) in a pressure reaction vial. The vial was sealed and heated to 60 °C. A solution of triethyl amine (22.2 mg, 220 µmol, 2.20 equiv) in dry THF (1 mL) was quickly added. Fast gas evolution was observed and the reaction was stirred for 17 h at 60 °C. After cooling to 21 °C, the solvent was removed under reduced pressure. The residue was re-dissolved in DCM (10 mL) and extracted with water (20 mL) and brine (20 mL). The combined organic layers were dried over $MgSO_4$, filtered and the solvent removed under reduced pressure.

2,5-Dioxopyrrolidin-1-yl (*E*)-4-(phenyldiazenyl)benzoate[2] (3a)

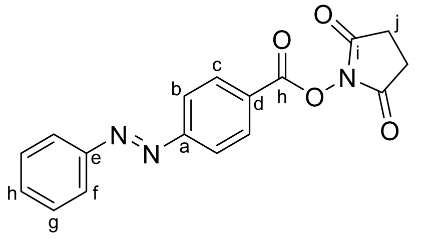

Compound **3a** was synthesized according to general procedure 2a from (*E*)-1-(4-iodophenyl)-2-phenyldiazene (**1a**) (61.6 mg, 200 µmol, 2.00 equiv). The product **3b** was obtained after crystallization from *n*-hexane/DCM (3:1) at -21 °C as a red solid (64.1 mg, 198 µmol, 99%, Lit.:[2] 84%[2]).

^{1}H NMR (600 MHz, $CDCl_3$) δ = 8.30 (d, ^3J = 8.5 Hz, 2H, *H*-c), 8.02 (d, ^3J = 8.5 Hz, 2H, *H*-b), 7.99 – 7.96 (m, 2H, *H*-f), 7.57 – 7.52 (m, 3H, *H*-g/h), 2.94 (s, br, 4H, *H*-j) ppm.
^{13}C{^{1}H} NMR (151 MHz, $CDCl_3$) δ = 169.3 (*C*-i), 161.6 (*C*-h), 156.3 (*C*-a), 152.6 (*C*-e), 132.3 (*C*-h), 131.9 (*C*-c), 129.4 (*C*-g), 126.7 (*C*-d), 123.5 (*C*-f), 123.2 (*C*-b), 25.9 (*C*-j) ppm.
IR (ATR): $\tilde{\nu}$ = 2999 (w), 2359 (w), 1790 (s), 1726 (s), 1600 (m), 1496 (w), 1427 (m), 1408 (m), 1366 (m), 1306 (w), 1256 (w), 1204 (s), 1151 (m), 1070 (s), 1019 (m), 999 (s), 928 (m), 867 (s), 849 (m), 812 (m), 771 (s) cm^{-1}.
HRMS (EI, 70 eV) *m/z* (%): $[M]^+$ calcd for $[C_{17}H_{13}N_3O_4]^+$ 323.09006; found 323.09003 (10), 209.2 (100).
Mp: 226 °C.
R*f*: 0.65 (*n*-hexane/DCM 1:1).

2,5-Dioxopyrrolidin-1-yl (*E*)-3-(phenyldiazenyl)benzoate[2] (3b)

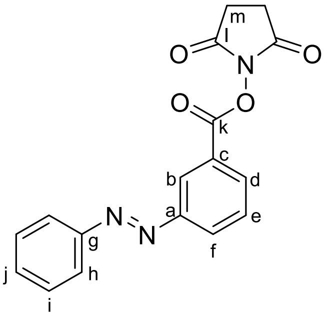

Compound **3b** was synthesized according to general procedure 1a from (*E*)-1-(4-iodophenyl)-2-phenyldiazene (**1b**) (61.6 mg, 200 µmol, 2.00 equiv). The compound was purified by column chromatography on silica (eluent: gradient *n*-hexane/ethyl acetate 1:0 → 0:1) yielding **3b** as an orange solid (60.5 mg, 188 µmol, 94%, Lit.:[2] 95%[3]).

^{1}H NMR (600 MHz, $CDCl_3$) δ = 8.68 (at, ^4J = 1.9 Hz, 1H, *H*-b), 8.25 – 8.22 (m, 2H, *H*-d/f), 7.97 – 7.94 (m, 2H, *H*-h), 7.69 (t, ^3J = 7.8 Hz, 1H, *H*-e), 7.57 – 7.50 (m, 3H, *H*-i/j), 2.94 (s, br, 4H, *H*-m) ppm.
^{13}C{^{1}H} NMR (151 MHz, $CDCl_3$) δ = 169.2 (*C*-l), 161.6 (*C*-k), 152.8 (*C*-a), 152.5 (*C*-g), 132.5 (*C*-d), 131.9 (*C*-j), 129.9 (*C*-e), 129.4 (*C*-i), 129.1 (*C*-f), 126.4 (*C*-d), 125.0 (*C*-b), 123.3 (*C*-h), 25.9 (*C*-m) ppm.
IR (ATR): $\tilde{\nu}$ = 2921 (w), 2359 (w), 1766 (m), 1727 (s), 1431 (m), 1371 (m), 1287 (w), 1257 (m), 1203 (s), 1149 (m), 1135 (m), 1070 (s), 1038 (m), 1014 (m), 923 (m), 867 (m), 812 (m), 765 (s), 740 (s) cm^{-1}.
HRMS (EI, 70 eV) *m/z* (%): $[M]^+$ calcd for $[C_{17}H_{13}N_3O_4]^+$ 323.09006; found 323.09006 (7), 77.0 (100).
Mp: 151 °C.
R*f*: 0.59 (*n*-hexane/DCM 1:1).

---

[2] In this report, the product was prepared by coupling of the corresponding carboxylic acid with NHS using DCC.
[3] In this report, the product was prepared by coupling of the corresponding carboxylic acid with NHS using DCC.

2,5-Dioxopyrrolidin-1-yl (*E*)-4-((4-hydroxyphenyl)diazenyl)benzoate[3] (**3d**)

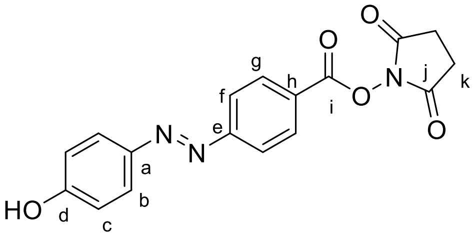

Compound **3d** was synthesized according to general procedure 1a from (*E*)-4-((4-iodophenyl)diazenyl)phenol (**1d**) (64.8 mg, 200 µmol, 2.00 equiv). The compound was purified by column chromatography on silica (eluent: gradient *n*-hexane/ethyl acetate 1:0 → 0:1) yielding **3d** as an orange powder (64.5 mg, 190 µmol, 95%, Lit.:[3] 90%[4]).

^{1}H NMR (600 MHz, DMSO-d_6) δ = 10.60 (s, 1H, O*H*-d), 8.27 (d, ^3J = 8.2 Hz, 2H, *H*-g), 8.01 (d, ^3J = 8.2 Hz, 2H, *H*-f), 7.89 (d, ^3J = 8.4 Hz, 2H, *H*-b), 6.99 (d, ^3J = 8.4 Hz, 2H, *H*-c), 2.92 (s, 4H, *H*-k*)* ppm.
^{13}C{^{1}H} NMR (151 MHz, DMSO-d_6) δ = 170.79 (C-j), 162.66 (C-a), 161.82 (C-i), 156.45 (C-e), 145.85 (C-d), 132.01 (*C*-g), 126.20 (*C*-b), 125.62 (C-h), 123.33 (*C*-f), 116.68 (*C*-c), 26.03 (*C*-k).
IR (ATR): $\tilde{\nu}$ = 3487 (w), 1764 (m), 1719 (s), 1601 (m), 1591 (w), 1431 (m), 1405 (m), 1275 (m), 1206 (s), 1156 (m), 1098 (m), 1015 (m), 1005 (s), 991 (m), 859 (w), 841 (s), 738 (m), 723 (m), 690 (m), 661 (m) cm^{-1}.
HRMS (EI, 70 eV) *m/z* (%): [M]$^+$ calcd for [$C_{17}H_{13}N_3O_5$]$^+$ 339.08497; found 339.08536 (15), 93.0 (100).
Mp: 241 °C.
R*f*: 0.25 (*n*-hexane/ethyl acetate 1:1).

2,5-Dioxopyrrolidin-1-yl (*E*)-4-((4-aminophenyl)diazenyl)benzoate (**3e**)

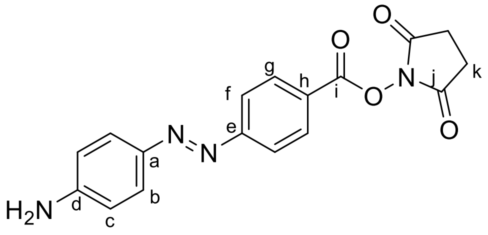

Compound **3e** was synthesized according to general procedure 1a from (*E*)-4-((4-iodophenyl)diazenyl)-aniline (**1e**) (64.8 mg, 200 µmol, 2.00 equiv). The solid was dissolved in ethyl acetate, washed with water (2 × 20 mL) and brine (20 mL). The combined organic phases were dried over $MgSO_4$, filtered and the solvent removed under reduced pressure. The crude product was crystalized from *n*-hexane/DCM (3:1) to obtain **3e** as an orange solid (65.6 mg, 172 µmol, 86%).

^{1}H NMR (600 MHz, DMSO-d_6*)* δ = 8.22 (d, ^3J = 8.6 Hz, 2H, *H*-g), 7.93 (d, ^3J = 8.6 Hz, 2H, *H*- f), 7.75 (d, ^3J = 8.9 Hz, 2H, *H*- b), 6.71 (d, ^3J = 8.9 Hz, 2H, *H*-c), 6.47 (s, 2H, NH_2-d) 2.91 (s, br, 4H, *H*-k) ppm.
^{13}C{^{1}H} NMR (151 MHz, DMSO-d_6*)* δ = 170.37 (*C*-j), 161.43 (*C*-i), 156.65 (*C*-e), 154.35 (*C*-a), 143.04 (*C*-d), 131.46 (*C*-g), 126.37 (*C*-b), 123.82 (*C*-h), 122.37 (*C*-f), 113.54 (*C*-c), 25.57 (*C*-k) ppm.
IR (ATR): $\tilde{\nu}$ = 3451 (w), 3366 (w), 1761 (s), 1725 (s), 1597 (m), 1456 (s), 1427 (m), 1401 (w), 1320 (w), 1255 (w), 1231 (w), 1205 (s), 1076 (m), 1046 (w), 1014 (m), 1014 (m), 861 (m), 835 (s), 726 (w), 690 (w) cm^{-1}.
HRMS (EI, 70 eV) *m/z* (%): [M]$^+$ calcd for [$C_{17}H_{14}N_4O_4$]$^+$ 338.10096; found 338.10115 (10), 92.1 (100).
Mp: 218 °C.
R*f*: 0.25 (*n*-hexane/ethyl acetate 1:1).

[4] In this report, the product was prepared by coupling of the corresponding carboxylic acid with NHS using DCC.

2,5-Dioxopyrrolidin-1-yl (*E*)-4-((4-acetamidophenyl)diazenyl)benzoate (**3f**)

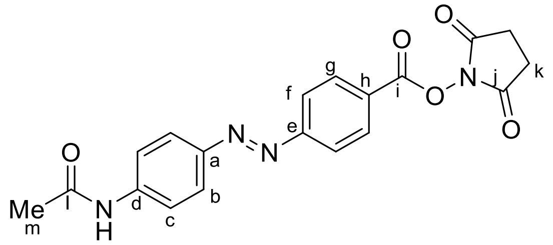

Compound **3f** was synthesized according to general procedure 1a from (*E*)*N*-(4-((4-iodophenyl)-diazenyl)phenyl)acetamide (**1f**) (64.8 mg, 200 µmol, 2.00 equiv). The solid was dissolved in ethyl acetate, washed with water (2 × 20 mL) and brine (20 mL). The combined organic phases were dried over $MgSO_4$, filtered and the solvent removed under reduced pressure. The crude product was crystalized from *n*-hexane/DCM (3:1) to obtain **3f** as an orange solid (65.6 mg, 172 µmol, 86%).

^{1}H NMR (600 MHz, DMSO- d_6) δ = 10.40 (s, 1H, N*H*-d) 8.29 (d, ^3J = 8.5 Hz, 2H, *H*-g), 8.05 (d, ^3J = 8.5 Hz, 2H, *H*-f), 7.97 (d, ^3J = 8.9 Hz, 2H, *H*-c), 7.85 (d, ^3J = 8.9 Hz, 2H, *H*-b), 2.92 (s, br, 4H, *H*-k), 2.12 (s, 3H, *H*-m) ppm.
^{13}C{^{1}H} NMR (151 MHz, DMSO- d_6) δ = 170.31 (*C*-j), 169.05 (*C*-l), 161.33 (*C*-i), 155.84 (*C*-a), 147.44 (*C*-d), 143.57 (*C*-a), 131.59 (*C*-g), 125.63 (*C*-h), 124.50 (*C*-c), 123.08 (*C*-f), 119.15 (*C*-b), 25.59 (*C*-k), 24.25 (*C*-m) ppm.
IR (ATR): $\tilde{\nu}$ = 3559 (w), 1757 (m), 1732 (m), 1688 (m), 1529 (s), 1446 (m), 1311 (m), 1300 (m), 1235 (m), 1208 (s), 998.3 (m), 861 (s), 848 (m), 739 (m) cm^{-1}.
HRMS (EI, 70 eV) *m/z* (%): [M]$^+$ calcd for $[C_{19}H_{16}N_4O_5]^+$ 380.11152; found 380.11201 (15), 134.2 (100).
Mp: 274 °C.
R*f*: 0.15 (*n*-hexane/ethyl acetate 1:1).

2,5-Dioxopyrrolidin-1-yl (*E*)-4-((4-nitrophenyl)diazenyl)benzoate (**3g**)

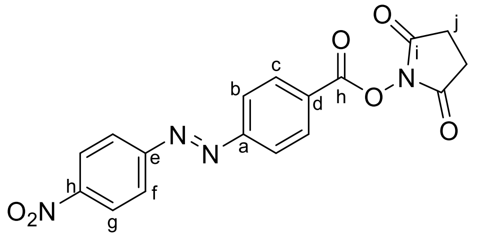

Compound **3c** was synthesized according to general procedure 2a from (*E*)-1-(4-nitrophenyl)-2-(4-iodophenyl)diazene (**1g**) (77.4 mg, 200 µmol, 2.00 equiv). The product **3g** was obtained after crystallization from *n*-hexane/DCM (3:1) at -21 °C as an orange solid (73.1 mg, 198 µmol, 99%).

^{1}H NMR (600 MHz, $CDCl_3$) δ = 8.42 (d, ^3J = 8.5 Hz, 2H, *H*-g), 8.33 (d, ^3J = 8.1 Hz, 2H, *H*-c), 8.11 (d, ^3J = 8.5 Hz, 2H, *H*-f), 8.08 (d, ^3J = 8.1 Hz, 2H, *H*-b), 2.95 (s, br, 4H, *H*-j) ppm.
^{13}C{^{1}H} NMR (151 MHz, $CDCl_3$) δ = 169.2 (*C*-i), 161.3 (*C*-h), 155.7 (*C*-a), 155.4 (*C*-e), 149.5 (*C*-h), 132.0 (*C*-c), 128.0 (*C*-d), 125.0 (*C*-g), 124.1 (*C*-f), 123.7 (*C*-b), 25.9 (*C*-j) ppm.
IR (ATR): $\tilde{\nu}$ = 3080 (w), 1767 (m), 1761 (s), 1606 (m), 1530 (s), 1431 (m), 1407(m), 1346 (s), 1322 (m), 1259 (m), 1237 (m), 1199 (s), 1110 (m), 1076 (s), 1023 (m), 1007 (s), 870 (s), 854 (m), 816 (m), 764 (m), 752 (m) cm^{-1}.
HRMS (EI, 70 eV) *m/z* (%): [M]$^+$ calcd for $[C_{17}H_{12}N_4O_6]^+$ 368.07514; found 368.07562 (4), 254.2 (100).
Mp: 245 °C.
R*f*: 0.59 (*n*-hexane/DCM 1:1).

2,5-Dioxopyrrolidin-1-yl (*E*)-4-((4-(allyloxy)phenyl)diazenyl)benzoate (3h)

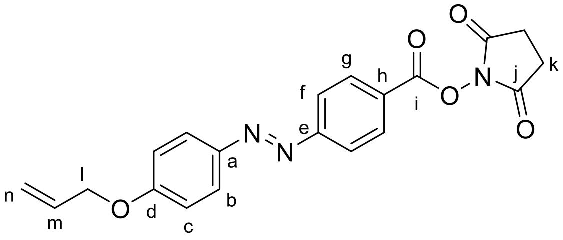

Compound **3h** was synthesized according to general procedure 1a from (*E*)-1-(4-(allyloxy)phenyl)-2-(4-iodophenyl)diazene (**1h**) (72.8 mg, 200 µmol, 2.00 equiv). The compound was purified by column chromatography on silica (eluent: gradient *n*-hexane/ethyl acetate 1:0 → 0:1) yielding **3h** as an orange powder (45.3 mg, 119 µmol, 60%).

^{1}H NMR (600 MHz, $CDCl_3$) δ = 8.27 (d, ^3J = 8.6 Hz, 2H, *H*-g), 7.97 (d, ^3J = 8.6 Hz, 4H, *H*-f/b), 7.05 (d, ^3J = 8.9 Hz, 2H, *H*-c), 6.09 (m, 1H, *H*-m), 5.46 (d, ^2J = 10.5 Hz, 1H, *H*-n), 5.35 (d, ^2J = 10.5 Hz, 1H, *H*-n), 4.65 (d, ^3J = 5.0 Hz, 2H, *H*-l), 2.93 (s, 4H, *H*-k) ppm.
^{13}C{^{1}H} NMR (151 MHz, $CDCl_3$) δ = 169.32 (*C*-j), 162.17 (*C*-d), 161.62 (*C*-i), 157.04 (*C*-e), 148.03 (*C*-a), 132.69 (*C*-m), 131.87 (*C*-g), 126.02 (*C*-h), 125.60 (*C*-f/b), 122.92 (*C*-f/b), 118.41 (*C*-n), 115.27 (*C*-c), 69.26 (*C*-l), 24.35 (*C*-k).
IR (ATR): $\tilde{\nu}$ = 2921 (w), 2851 (w), 1768 (w), 1731 (m), 1600 (m), 1499 (m), 1371 (w), 1204 (s), 1070 (s), 1016 (m), 992 (s), 861 (m), 812 (w), 760 (w), 723 (w), 687 (w) cm^{-1}.
HRMS (EI, 70 eV) *m/z* (%): $[M]^+$ calcd for $[C_{20}H_{17}N_3O_5]^+$ 379.11627; found 379.11643 (20), 133.1 (100)
Mp: - (decomposition).
R*f*: 0.35 (*n*-hexane/ethyl acetate 1:1).

2,5-Dioxopyrrolidin-1-yl (*E*)-4-((4-((9-hydroxynonyl)oxy)phenyl)diazenyl)benzoate (3i)

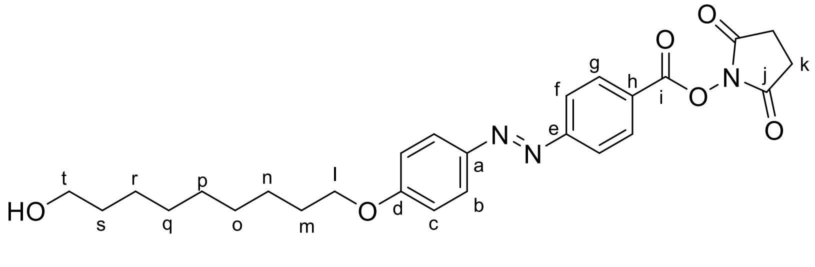

Compound **3i** was synthesized according to general procedure 1a from (*E*)-9-(4-((4-iodophenyl)diazenyl)phenoxy)nonan-1-ol (**1i**) (93.2 mg, 200 µmol, 2.00 equiv). The compound was purified by column chromatography on silica (eluent: gradient *n*-hexane/ethyl acetate 1:0 → 0:1) yielding **3i** as an yellow solid (76.3 mg, 158 µmol, 79%).

^{1}H NMR (600 MHz, $CDCl_3$) δ = 8.27 (d, ^3J = 6.6 Hz, 2H, *H*-g), 7.96 (dd, ^3J = 8.9, 6.6 Hz, 4H, *H*-f/b), 7.02 (d, ^3J = 8.9 Hz, 2H, *H*-c), 4.06 (t, ^3J = 6.5 Hz, 2H, *H*-l), 3.65 (t, ^3J = 6.6 Hz, 2H, *H*-t), 2.93 (s, 4H, *H*-k), 1.87 – 1.79 (m, 2H, *H*-m), 1.60 – 1.52 (m, 2H, *H*-s), 1.51 – 1.48 (m, 2H, *H*-n), 1.41 – 1.30 (m, 8H, *H*-o/p/r/q), 1.23 (s, 1H, O*H*-t) ppm.
^{13}C{^{1}H} NMR (151 MHz, $CDCl_3$) δ = 169.12 (*C*-j), 162.63 (*C*-d), 161.42 (*C*-i), 156.42 (*C*-e), 146.77 (*C*-a), 131.65 (*C*-g), 125.72 (*C*-h), 125.42 (*C*-f/b), 122.68 (*C*-f/b), 115.65 (*C*-c), 68.38 (*C*-l), 63.00(*C*-t), 32.71 (*C*-s), 29.43 (*C*-o/p/r/q), 29.28 (*C*-o/p/r/q), 29.22 (*C*-o/p/r/q), 29.07 (*C*-m), 25.92 (*C*-o/p/r/q), 25.66 (*C*-n), 25.64 (*C*-k) ppm.
IR (ATR): $\tilde{\nu}$ = 3523 (w), 2921 (w), 2850 (w), 1797 (m), 1773 (s), 1715 (s), 1600 (w), 1500 (m), 1381 (w), 1296 (m), 1255 (m), 1210 (s), 1138 (m), 1107 (m), 1075 (s), 1050 (s), 1016(s), 988 (s), 863 (m), 843 (s), 814 (m), 723 (m), 688 (w) cm^{-1}.

HRMS (EI, 70 eV) *m/z* (%): $[M]^+$ calcd for $[C_{26}H_{31}N_3O_6]^+$ 481.22074; found 481.22050 (5), 55.1 (100).
Mp: 156 °C.
R*f*: 0.15 (*n*-hexane/ethyl acetate 1:1).

2,5-Dioxopyrrolidin-1-yl (*E*)-4-((2,6-dimethyl-4-(nonyloxy)phenyl)diazenyl)-3,5-dimethylbenzoate (3j)

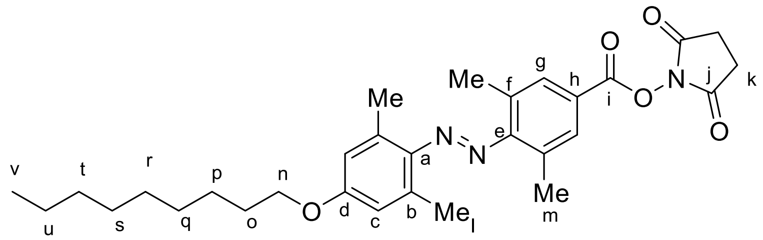

Compound **3j** was synthesized according to general procedure 2a from (*E*)-1-(2,6-dimethyl-4-(nonyloxy)phenyl)-2-(4-iodo-2,6-dimethylphenyl)diazene (**1j**) (92.8 mg, 200 µmol, 2.00 equiv). The compound was purified by column chromatography on silica (eluent: gradient *n*-hexane/ethyl acetate 1:0 → 0:1) yielding **3j** as a red powder (97.0 mg, 186 µmol, 94%).

The NMR yield was obtained according to general procedure 1a with the difference that **1j** (46.4 mg, 100 µmol, 1.00 equiv), $Pd(OAc)_2$ (675 µg, 3.00 µmol, 3 mol%), Xantphos (1.45 mg, 2.50 µmol, 2.5 mol%), 2,5-dioxopyrrolidin-1-yl formate (**2**) (28.6 mg, 200 µmol, 2.00 equiv), 1,3,5-trimethoxybenzene (16.8 mg, 100 µmol) and triethyl amine (22.2 mg, 220 µmol, 1.10 equiv) were used.

^{1}H NMR (600 MHz, $CDCl_3$) δ = 7.91 (s, 2H, *H*-g), 6.70 (s, 2H, *H*-c), 4.03 (t, ^3J = 6.5 Hz, 2H, *H*-n), 2.92 (s, 4H, *H*-k), 2.55 (s, 6H, *H*-l), 2.34 (s, 6H, *H*-m), 1.85 – 1.77 (m, 2H, *H*-o), 1.53 – 1.43 (m, 2H, *H*-p), 1.40 – 1.25 (m, 10H, *H*-q,r,s,t,u), 0.92 – 0.86 (m, 3H, *H*-v).
^{13}C{^{1}H} NMR (151 MHz, $CDCl_3$) δ = 169.32 (*C*-j), 161.72 (*C*-i), 160.34 (*C*-d), 157.55 (*C*-e), 143.83 (*C*-a), 136.72 (*C*-b), 131.32 (*C*-g), 130.41 (*C*-f), 122.77 (*C*-h), 115.31 (*C*-c), 68.13 (*C*-n), 31.90 (*C*-q,r,s,t,u), 29.55 (*C*-q,r,s,t,u), 29.38 (*C*-q,r,s,t,u), 29.28 (*C*-q,r,s,t,u), 29.21 (*C*-o), 26.02 (*C*-p), 25.71 (*C*-k), 22.69 (*C*-q,r,s,t,u), 21.38 (*C*-m), 19.09 (*C*-l), 14.13 (*C*-v).
IR (ATR): $\tilde{\nu}$ = 2922 (m), 2848 (m), 1761 (s), 1732(s), 1599 (m), 1489 (w), 1465 (w), 1431 (w), 1356 (w), 1313 (m), 1297 (m), 1248 (m), 1187 (s), 1161 (s), 1139 (s), 1077 (s), 1058 (s), 1044 (m), 994 (w), 911 (s), 903(s), 868 (m), 854 (m), 827 (w), 806 (w), 689 (w) cm^{-1}.
HRMS (EI, 70 eV) *m/z* (%): $[M]^+$ calcd for $[C_{30}H_{39}N_3O_5]^+$ 521.28842; found 521.28660 (10), 69.1 (100).
Mp: 78 °C.
R*f*: 0.45 (*n*-hexane/ethyl acetate 1:1).

2,5-Dioxopyrrolidin-1-yl (*E*)-4-((2,7-bis(4-(hexyloxy)phenoxy)naphthalen-1-yl)diazenyl)-3,5-dimethylbenzoate (**3k**)

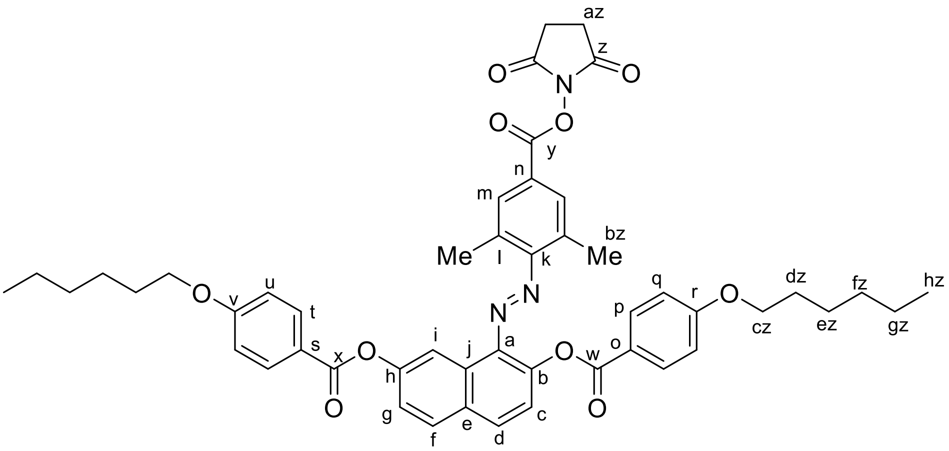

Compound **3k** was synthesized according to general procedure 1a from (*E*)-1-(2,7-bis(4-(hexyloxy)phenoxy)naphthalen-1-yl)-2-(4-iodo-2,6-dimethylphenyl)diazene (**1k**) (154 mg, 200 µmol, 2.00 equiv). The compound was purified by column chromatography on silica (eluent: gradient *n*-hexane/ethyl acetate 1:0 → 0:1) yielding **3k** as a red powder (125 mg, 148 µmol, 74%).

^{1}H NMR (600 MHz, $CDCl_3$) δ = 8.53 (d, ^4J = 2.3 Hz, 1H, *H*-i), 8.16 (d, ^3J = 8.8 Hz, 2H, *H*-t), 8.06 – 8.03 (m, 3H, *H*-p/c), 8.01 (d, ^3J = 8.9 Hz, 1H, *H*-g), 7.81 (s, 2H, *H*-m), 7.49 (dd, ^3J = 8.9, ^4J = 2.3 Hz, 1H, *H*-f), 7.44 (d, ^3J = 8.8 Hz, 1H, *H*-d), 6.97 (d, ^3J = 8.8 Hz, 2H, *H*-u), 6.91 (d, ^3J = 8.8 Hz, 2H, *H*-q), 4.03 (dt, ^2J = 13.3, ^3J = 6.7 Hz, 4H, *H*-cz), 2.90 (s, 4H, *H*-az), 2.24 (s, 6H, *H*-bz), 1.80 (dt, ^2J = 13.3, ^3J = 6.7 Hz, 4H, *H*-dz), 1.55 – 1.44 (m, 4H, *H*-ez), 1.38 - 1.32 (m, 8H, *H*-fz/gz), 0.93 – 0.90 (m, 6H, *H*-hz) ppm.
^{13}C{^{1}H} NMR (151 MHz, $CDCl_3$) δ = 169.35 (C-z), 165.11 (C-x), 165.08 (C-w), 163.81 (C-s), 163.75 (C-o), 161.68 (*C*-y), 157.03 (*C*-k), 151.06 (C-b), 140.03 (C-e), 137.81 (C-j), 132.53 (*C*-t), 132.45 (*C*-p), 131.33 (*C*-m), 131.20 (*C*-f), 131.01 (C-a), 130.53 (C-h), 129.67 (*C*-g), 123.74 (C-c), 123.03 (*C*-d), 122.40 (*C*-n), 121.40 (C-v), 121.17 (C-r), 115.84 (*C*-i), 114.47 (*C*-u/q), 68.48 (*C*-cz), 68.45 (*C*-cz), 31.71 (*C-fz/gz)*, 31.69 (*C-fz/gz)*, 29.22 (*C*-dz), 29.20 (*C*-dz), 25.80 (*C*-az, *C*ez), 22.73(*C-fz/gz)*, 18.74 (*C*-bz), 14.17 (*C*-hz) ppm.
IR (ATR): $\tilde{\nu}$ = 2929 (w), 1774 (w), 1735 (s), 1604 (m), 1577 (w), 1511 (w), 1456 (w), 1420 (w), 1367 (w), 1293 (w), 1247 (s), 1202 (s), 1161 (s), 1063 (s), 1017 (s), 1007 (m), 995 (w), 919 (w), 890 (m), 874 (w), 846 (m), 759 (w), 749 (w), 689 (w) cm^{-1}.
HRMS (ESI): *m/z* = $[M + Na]^+$ calcd for $[C_{49}H_{51}IN_3O_{10} + Na]^+$ 864.34619, found 864.34667.
Mp: 80 °C.
R*f*: 0.45 (*n*-hexane/ethyl acetate 1:1).

2,5-Dioxopyrrolidin-1-yl (*E*)-4-((4-bromophenyl)diazenyl)benzoate (**3l**)

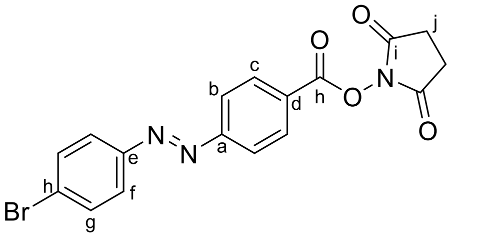

Compound **3l** was synthesized according to general procedure 2a from (*E*)-1-(4-bromophenyl)-2-(4-iodophenyl)diazene (**1l**) (77.4 mg, 200 µmol, 2.00 equiv). The product was obtained after crystallization from *n*-hexane/DCM (3:1) at -21 °C as an orange solid (76.3 mg, 190 µmol, 95%).

^{1}H NMR (600 MHz, $CDCl_3$) δ = 8.30 (d, ^3J = 8.3 Hz, 2H, *H*-c), 8.01 (d, ^3J = 8.3 Hz, 2H, *H*-b), 7.85 (d, ^3J = 8.8 Hz, 2H, *H*-f), 7.69 (d, ^3J = 8.8 Hz, 2H, *H*-g), 2.94 (s, br, 4H, *H*-j) ppm.
^{13}C{^{1}H} NMR (151 MHz, $CDCl_3$) δ = 169.3 (*C*-i), 161.5 (*C*-h), 156.1 (*C*-a), 151.3 (*C*-e), 132.7 (*C*-g), 131.9 (*C*-c), 127.0 (*C*-d), 126.9 (*C*-h), 124.9 (*C*-f), 123.3 (*C*-b), 25.9 (*C*-j) ppm.
IR (ATR): $\tilde{\nu}$ = 2987 (w), 2359 (w), 1790 (w), 1770 (m), 1722 (s), 1601 (m), 1571 (m), 1478 (m), 1426 (m), 1426 (m), 1368 (m), 1260 (m), 1231 (m), 1206 (s), 1144 (m), 1074 (s), 1016 (m), 995 (s), 859 (m), 838 (m), 819 (m), 759 (m), 706 (m) cm^{-1}.
HRMS (EI, 70 eV) *m/z* (%): $[M]^+$ calcd for $[C_{17}H_{12}BrN_3O_4]^+$ 401.00057; found 401.00131 (7), 76.1 (100).
Mp: 285 °C.
R$_f$: 0.65 (*n*-hexane/DCM 1:1).

2,5-Dioxopyrrolidin-1-yl (*E*)-4-((3-bromophenyl)diazenyl)benzoate (3m)

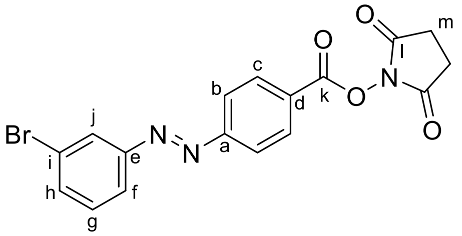

Compound **3m** was synthesized according to general procedure 2a from (*E*)-1-(3-bromophenyl)-2-(4-iodophenyl)diazene (**1m**) (77.4 mg, 200 µmol, 2.00 equiv). The product was obtained after crystallization from *n*-hexane/DCM (3:1) at -21 °C as an orange solid (76.9 mg, 198 µmol, 99%).

^{1}H NMR (600 MHz, $CDCl_3$) δ = 8.31 (d, ^3J = 8.5 Hz, 2H, *H*-c), 8.11 (at, ^4J = 1.9 Hz, 1H, *H*-j), 8.02 (d, ^3J = 8.5 Hz, 2H, *H*-b), 7.94 (ddt, ^3J = 7.9 Hz, ^4J = 1.9 Hz, ^4J = 0.9 Hz, 1H, *H*-f/h), 7.66 (ddt, ^3J = 7.9 Hz, ^4J = 1.9 Hz, ^4J = 0.9 Hz, 1H, *H*-f/h), 7.44 (t, ^3J = 7.9 Hz, 1H, *H*-g), 2.94 (s, br, 4H, *H*-m) ppm.
^{13}C{^{1}H} NMR (151 MHz, $CDCl_3$) δ = 169.2 (*C*-l), 161.5 (*C*-k), 155.9 (*C*-a), 153.4 (*C*-e), 134.8 (*C*-h), 131.9 (*C*-c), 130.8 (*C*-g), 127.2 (*C*-d), 125.1 (*C*-j), 123.7 (*C*-f), 123.4 (*C*-i), 123.4 (*C*-b), 25.9 (*C*-m) ppm.
IR (ATR): $\tilde{\nu}$ = 2937 (w), 1769 (m), 1729 (s), 1598 (m), 1568 (m), 1455 (w), 1435 (m), 1409 (m), 1353 (m), 1257 (m), 1237 (m), 1196 (s), 1143 (m), 1071 (s), 1017 (m), 994 (s), 892 (m), 862 (s), 853 (s), 808 (m), 796 (s), 760 (m), 742 (m) cm^{-1}.
HRMS (EI, 70 eV) *m/z* (%): $[M]^+$ calcd for $[C_{17}H_{12}BrN_3O_4]^+$ 401.00057; found 401.00120 (7), 76.1 (100).
Mp: 192 °C.
R$_f$: 0.65 (*n*-hexane/DCM 1:1).

2,5-Dioxopyrrolidin-1-yl (*E*)-4-((2-bromophenyl)diazenyl)benzoate (3n)

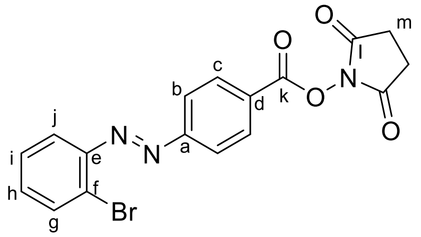

Compound **3n** was synthesized according to general procedure 2a from (*E*)-1-(2-bromophenyl)-2-(4-iodophenyl)diazene (**1n**) (77.4 mg, 200 µmol, 2.00 equiv). The product was obtained after crystallization from *n*-hexane/DCM (3:1) at -21 °C as a red solid (71.6 mg, 178 µmol, 89%).

^{1}H NMR (600 MHz, $CDCl_3$) δ = 8.31 (d, ^3J = 8.5 Hz, 2H, *H*-c), 8.07 (d, ^3J = 8.5 Hz, 2H, *H*-b), 7.80 (dd, ^3J = 7.9 Hz, ^4J = 1.7 Hz, 1H, *H*-g), 7.73 (dd, ^3J = 7.9 Hz, ^4J = 1.7 Hz, 1H, *H*-j), 7.42 (td, ^3J = 7.9 Hz, ^4J = 1.7 Hz, 1H, *H*-i), 7.38 (td, ^3J = 7.9 Hz, ^4J = 1.7 Hz, 1H, *H*-h), 2.94 (s, br, 4H, *H*-m) ppm.

^{13}C{^{1}H} NMR (151 MHz, $CDCl_3$) δ = 169.2 (*C*-l), 161.5 (*C*-k), 156.1 (*C*-a), 149.6 (*C*-e), 134.2 (*C*-g), 133.2 (*C*-h), 131.9 (*C*-c), 128.2 (*C*-i), 127.2 (*C*-d), 127.1 (*C*-f), 123.7 (*C*-b), 117.8 (*C*-j), 25.9 (*C*-m) ppm.
IR (ATR): $\tilde{\nu}$ = 2957 (W), 2359 (w), 1769 (m), 1731 (s), 1600 (m), 1458 (w), 1422 (m), 1407 (m), 1359 (m), 1255 (m), 1235 (m), 1199 (s), 1066 (s), 1045 (m), 997 (s), 859 (m), 849 (m), 807 (m), 780 (m), 761 (m), 716 (m) cm^{-1}.
HRMS (EI, 70 eV) *m/z* (%): $[M]^+$ calcd for $[C_{17}H_{12}BrN_3O_4]^+$ 401.00057; found 401.00125 (9), 76.1 (100).
Mp: 173 °C**.**
R*f*: 0.65 (*n*-hexane/DCM 1:1).

Bis(2,5-dioxopyrrolidin-1-yl)4,4´-(diazene-1,2-diyl)(*E*)-dibenzoate[5](**5a**)

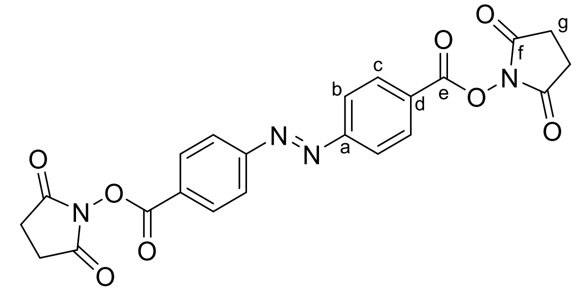

Compound **5a** was synthesized according to general procedure 2b from (*E*)-1,2-bis(4-iodophenyl)diazene (**4a**) (43.4 mg, 200 µmol). The product was obtained after crystallization from *n*-hexane/DCM (3:1) as a red solid (42.0 mg, 194 µmol, 97%, Lit.:[4] 94%[4]).

^{1}H NMR (600 MHz, $CDCl_3$) δ = 8.33 (d, ^3J = 8.5 Hz, 2H, *H*-c), 8.08 (d, ^3J = 8.1 Hz, 2H, *H*-b), 2.94 (s, br, 4H, *H*-g) ppm.
^{13}C{^{1}H} NMR (151 MHz, $CDCl_3$) δ = 169.2 (*C*-f), 161.4 (*C*-e), 155.9 (*C*-a), 132.0 (*C*-c), 127.7 (*C*-d), 123.6 (*C*- b), 25.9 (*C*-g) ppm.
IR (ATR): $\tilde{\nu}$ = 2923 (w), 1770 (m), 1732 (s), 1600 (m), 1426 (w), 1414 (m), 1365 (m), 1310 (m), 1256 (m), 1236 (m), 1201 (s), 1066 (s), 1046 (m), 1015 (m), 987 (s), 977 (s), 873 (s), 843 (m), 809 (m), 767 (m), 727 (m) cm^{-1}.
HRMS (EI, 70 eV) *m/z* (%): $[M]^+$ calcd for $[C_{22}H_{16}N_4O_8]^+$ 464.09626; found 464.09599 (9), 349.9 (100).
Mp: 276 °C Lit.:[4] >250 °C).
R*f* = 0.65 (*n*-hexane/DCM 1:1).

Bis(2,5-dioxopyrrolidin-1-yl) 4,4'-(diazene-1,2-diyl)(*E*)-bis(3,5-dimethoxybenzoate) (**5b**)

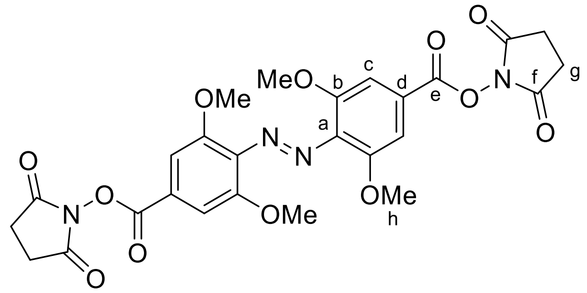

Compound **5b** was synthesized according to general procedure 1b from (*E*)-1,2-bis(4-iodo-2,6-dimethoxyphenyl)diazene[5] (**4b**) (54.4 mg, 100 µmol, 1.00 equiv). The compound was purified by column chromatography on silica (eluent: gradient *n*-hexane/ethyl acetate 1:0 → 0:1) yielding **5b** as a red powder (47.1 mg, 90.5 µmol, 91%).

[4] In this report, the product was prepared by coupling of the corresponding carboxylic acid with NHS using EDC and DMAP.
[5] 90% purity

^{1}H NMR (600 MHz, DMSO-d_6) δ = 7.45 (s, 4H, *H*-c), 3.93 (s, 12H, *H*-h), 2.94 (s, 4H, *H*-g) ppm.
^{13}C{^{1}H} NMR (151 MHz, DMSO-d_6) δ = 169.2 (*C*-f), 161.5 (*C*-e), 151.9 (*C*-a), 138.3 (*C*-b), 125.7 (*C*-d), 107.4 (*C*-c), 57.0 (*C*-h), 25.9 (*C*-g) ppm.
IR (ATR): $\tilde{\nu}$ = 2956 (w), 1732 (s), 1574 (m), 1455 (m), 1408 (m), 1360 (m), 1325 (m), 1231 (m), 1195 (m), 1120 (s), 1071 (s), 1016 (m), 993 (w), 894 (m), 853 (m), 833 (w), 808 (w), 771 (w), 741 (w) cm^{-1}.
HRMS (EI, 70 eV) *m/z* (%): [M]$^+$ calcd for $[C_{26}H_{24}N_4O_{12}]^+$ 584.13852; found 584.13941 (13), 149.1 (100).
Mp: 212 °C.
R$_f$: 0.50 (ethyl acetate).

Bis(2,5-dioxopyrrolidin-1-yl) 4,4'-(diazene-1,2-diyl)(*E*)-bis(3,5-dimethylbenzoate) (**5c**)

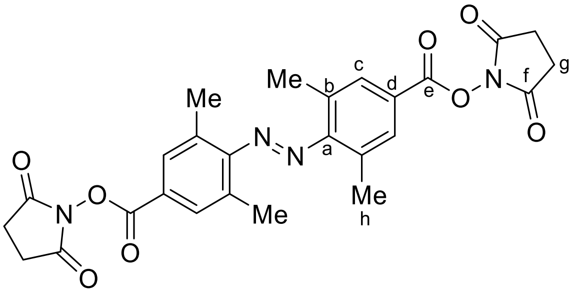

Compound **5c** was synthesized according to general procedure 1b from (*E*)-1,2-bis(4-iodo-2,6-dimethylphenyl)diazene (**4c**) (49.0 mg, 100 µmol, 1.00 equiv). The compound was purified by column chromatography on silica (eluent: gradient *n*-hexane/ethyl acetate 1:0 → 0:1) yielding **5c** as a red powder (47.1 mg, 90.5 µmol, 91%).

^{1}H NMR (600 MHz, $CDCl_3$) δ = 7.98 (s, 4 H, *H*-c), 2.95 (s, 8 H, *H*-g), 2.46 (s, 12 H, *H*-h) ppm.
^{13}C{^{1}H} NMR (151 MHz, $CDCl_3$) δ = 169.3 (*C*-f), 161.6 (*C*-e), 155.7 (*C*-a), 131.8 (*C*-c), 131.7 (*C*-b), 124.8 (*C*-d), 25.7 (*C*-g), 19.4 (*C*-h) ppm.
IR (ATR): $\tilde{\nu}$ = 3429 (m), 3351 (m), 3078 (w), 2926 (w), 2863 (w), 1614 (s), 1567 (m), 1481 (s), 1404 (s), 1309 (s), 1290 (s), 1270 (s), 1191 (m), 1163 (m), 1121 (m), 1069 (m), 933 (m), 884 (m), 862 (m), 812 (s), 780 (m), 748 (m), 733 (m) cm^{-1}.
HRMS (APCI): *m/z* = [M+H]$^+$ calcd for $[C_{26}H_{24}N_4O_8 + H]^+$ 521.166669; found 521.16629.
Mp: 175 °C.
R$_f$: 0.30 (DCM/ethyl acetate 1:1).

Bis(2,5-dioxopyrrolidin-1-yl) 4,4'-(diazene-1,2-diyl)(*E*)-bis(3,5-difluorobenzoate) (**5d**)

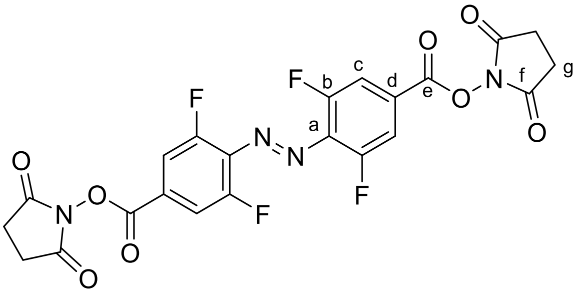

(*E*)-1,2-Bis(2,6-difluoro-4-iodophenyl)diazene (**4d**) (171 mg, 0.500 mmol, 1.00 equiv), *N*-hydroxy-succinimide (**7**) (253 mg, 2.20 mmol, 4.40 equiv) and 4-dimethylaminopyridine (DMAP) (12.5 mg, 100 µmol, 0.20 equiv) were dissolved in DMF (10 mL) at 21 °C. After 10 min of stirring, 1-ethyl-3-(3-dimethylaminopropyl)carbodiimide (EDCI) (310 mg, 11.1 mmol, 3.00 equiv) was added and the solution was stirred at 21 °C for 16 h under a nitrogen atmosphere. The mixture was diluted with DCM (100 mL) and washed with hydrochloric acid (0.1 M, 100 mL). The aqueous phase was extracted DCM (2 × 50 mL). The combined organic phases were dried over sodium sulphate and purified by column

chromatography on silica (eluent: gradient *n*-hexane/ethyl acetate 0:1 → 1:1). **5d** was obtained as an orange solid after crystallisation from THF/*n*-hexane (110 mg, 205 µmol, 41%).

^{1}H NMR (600 MHz, DMSO-d_6) δ = 8.11 (d, ^3J = 8.6 Hz, 4H, *H*-c), 2.93 (s, 8H, *H*-g) ppm.
^{13}C{^{1}H} NMR (151 MHz, DMSO-d_6) δ = 172.25 (*C*-e), 170.4 (*C*-f), 155.00 (d, ^1J = 264.3 Hz, *C*-b), 134.9 (*C*-a), 128.6 (*C*-d), 115.9 (d, ^2J = 24 Hz, *C*-c), 25.59 (*C*-g) ppm.
^{19}F NMR (565 MHz, DMSO-d_6) δ = -118.20 (s, *F*-b)
IR (ATR): $\tilde{\nu}$ = 3092 (w), 1804 (w), 1774 (w), 1731 (s), 1580 (m), 1431 (m), 1327 (m), 1200 (s), 1165 (s), 1072 (s), 1038 (s), 904 (m), 826 (w), 764 (w), 741 (s) cm^{-1}.
HRMS (EI, 70 eV) *m/z* (%): [M]$^+$ calcd for $[C_{22}H_{12}F_4N_4O_8]^+$ 536.05858; found 536.05884 (16), 140.1 (100).
Mp: 296 °C.
R*f*: 0.60 (ethyl acetate).

2,5-Dioxopyrrolidin-1-yl (Z)-11,12-dihydrodibenzo[c,g][1,2]diazocine-2-carboxylate (8a)

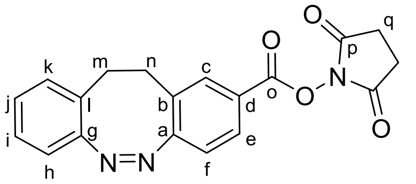

Compound **8a** was synthesized according to general procedure 2a from (*Z*)-2-iodo-11,12-dihydrodibenzo[c,g][1,2]-diazocine (**7a**) (43.4 mg, 200 µmol) in dry DMSO. The product was obtained after crystallization from *n*-hexane/DCM (3:1) as a yellow solid (69.9 mg, 198 µmol, 99%).

^{1}H NMR (600 MHz, $CDCl_3$) δ = 7.89 (dd, ^3J = 8.2 Hz, ^4J = 1.8 Hz, 1H, *H*-e), 7.78 (d, ^4J = 1.8 Hz, 1H, *H*-c), 7.17 (t, ^3J = 7.5 Hz, 1H, *H*-i), 7.06 (t, ^3J = 7.5 Hz, 1H, *H*-j), 6.99 (d, ^3J = 7.5 Hz, 1H, *H*-k), 6.93 (d, ^3J = 8.2 Hz, 1H, *H*-f), 6.85 (d, ^3J = 7.5 Hz, 1H, *H*-h), 3.07 – 2.77 (m, 8H, *H*-m/n/q) ppm.
^{13}C{^{1}H} NMR (151 MHz, $CDCl_3$) δ = 169.3 (*C*-p), 161.2 (*C*-o), 160.8 (*C*-a), 155.5 (*C*-g), 132.4 (*C*-c), 130.2 (*C*-k), 129.7 (*C*-b), 129.5 (*C*-e), 128.0 (*C*-j), 127.3 (*C*-l), 127.2 (*C*-i), 123.6 (*C*-d), 119.3 (*C*-f), 118.7 (*C*-h), 31.6 (*C*-n), 31.5 (*C*-m), 25.8 (*C*-q) ppm.
IR (ATR): $\tilde{\nu}$ = 2921 (w), 2851 (w), 2359 (w), 1769 (m), 1733 (s), 1601 (w), 1417 (m), 1358 (m), 1282 (m), 1236 (m), 1196 (s), 1064 (s), 1036 (m), 914 (m), 874 (m), 765 (m), 742 (s) cm^{-1}.
HRMS (EI, 70 eV) *m/z* (%): [M]$^+$ calcd for $[C_{19}H_{15}N_3O_4]^+$ 349.10571; found 349.10536 (7), 178.1 (100).
Mp: 207 °C.
R*f*: 0.46 (*n*-hexane/DCM 1:1).

Bis(2,5-dioxopyrrolidin-1-yl) (Z)-11,12-dihydrodibenzo[c,g][1,2]diazocine-2,9-dicarboxylate (8b)

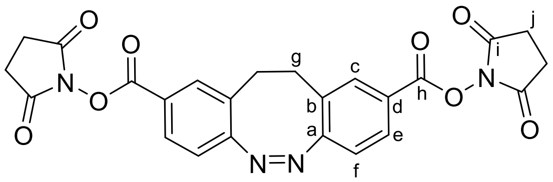

Compound **8b** was synthesized according to general procedure 2b from (*Z*)-2,9-diiodo-11,12-dihydrodibenzo[c,g][1,2]-diazocine (**7b**) (43.4 mg, 200 µmol) in dry DMSO. The product was obtained after crystallization from *n*-hexane/DCM (3:1) as a yellow solid (47.8 mg, 196 µmol, 98%).

^{1}H NMR (600 MHz, $CDCl_3$) δ = 7.95 (d, ^3J = 8.2 Hz, 2H, *H*-e), 7.82 (s, 2H, *H*-c), 7.00 (d, ^3J = 8.2 Hz, 2H, *H*-f), 3.12 – 2.91 (m, 4H, *H*-g), 2.88 (s, br, 8H, *H*-j) ppm.
^{13}C{^{1}H} NMR (151 MHz, $CDCl_3$) δ = 169.1 (*C*-i), 161.0 (*C*-h), 160.1 (*C*-a), 132.8 (*C*-c), 129.9 (*C*-e), 128.5 (*C*-b), 124.5 (*C*-d), 119.4 (*C*-f), 31.4 (*C*-g), 25.8 (*C*-j) ppm.
IR (ATR): $\tilde{\nu}$ = 2990 (w), 2359 (w), 1769 (m), 1732 (s), 1602 (m), 1482 (w), 1425 (m), 1366 (m), 1202 (s), 1069 (s), 1029 (m), 986 (m), 911 (m), 813 (m), 740 (s) cm^{-1}.
HRMS (ESI) *m/z*: [M + Na]$^+$ calcd for [$C_{24}H_{18}N_4O_8$ + Na]$^+$ 513.10168; found 513.10105.
Mp: 187 °C.
R*f*: 0.61 (ethyl acetate).

Di-*tert*-butyl 2,2'-((4,4'-(diazene-1,2-diyl)bis(benzoyl))bis(azanediyl))(*E*)-dipropionate (10a)

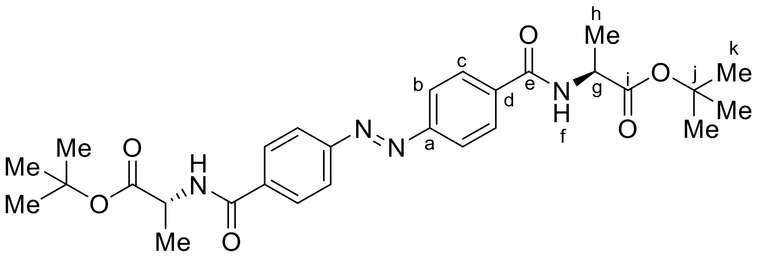

Triethyl amine (153 µL, 1.10 mmol, 2.20 equiv) was added to a solution of L-alanine *tert*-butyl ester hydrochloride (**9**) (200 mg, 1.10 mmol, 2.20 equiv) in dry DMF (10 mL). A solution of bis(2,5-dioxopyrrolidin-1-yl) 4,4'-(diazene-1,2-diyl)(*E*)-dibenzoate (**5a**) (232 mg, 500 µmol, 1.00 equiv) in dry DMF (7 mL) was added and the resulting mixture was stirred at 21 °C for 16 h. Water (100 mL) and ethyl acetate (15 mL) were added and the mixture was washed with brine (3 × 50 mL). The combined organic phases were dried over $MgSO_4$, filtered and the solvent removed under reduced pressure yielding the product as an orange-red solid (257 mg, 490 mmol, 98%).

^{1}H NMR (600 MHz, $CDCl_3$) δ = 8.01 – 7.95 (m, 8H, *H*-b/c), 6.89 (d, ^3J = 7.1 Hz, 2H, *H*-f), 4.70 (p, ^3J = 7.1 Hz, 2H, *H*-g), 1.52 (d, ^3J = 7.1 Hz, 6H, *H*-h), 1.51 (s, 18H, *H*-k) ppm.
^{13}C{^{1}H} NMR (151 MHz, $CDCl_3$) δ = 172.6 (*C*-i), 166.0 (*C*-e), 154.3 (*C*-a), 136.5 (*C*-d), 128.2 (*C*-c), 123.3 (*C*-b), 82.6 (*C*-j), 49.4 (*C*-g), 28.2 (*C*-k), 19.0 (*C*-h) ppm.
IR (ATR): $\tilde{\nu}$ = 3320 (br), 2979 (w), 2359 (w), 1731 (m), 1634 (s), 1529 (s), 1490 (m), 1454 (m), 1368 (m), 1343 (m), 1306 (m), 1271 (m), 1335 (m), 1153 (s), 1046 (m), 1012 (m), 942 (w), 893 (w), 861 (s), 848 (m), 779 (m), 737 (m), 708 (m) cm^{-1}.
HRMS (ESI) *m/z*: [M + Na]$^+$ calcd for [$C_{28}H_{36}N_4O_6$ + Na]$^+$ 547.25271; found 547.25271.
Mp: 187 °C.
R*f* = 0.70 (*n*-hexane/DCM 1:1).

Di-*tert*-butyl 2,2'-((11,12-dihydrodibenzo[c,g][1,2]diazocine-2,9-dicarbonyl)bis(azane-diyl))(*Z*)-dipropionate (**10b**)

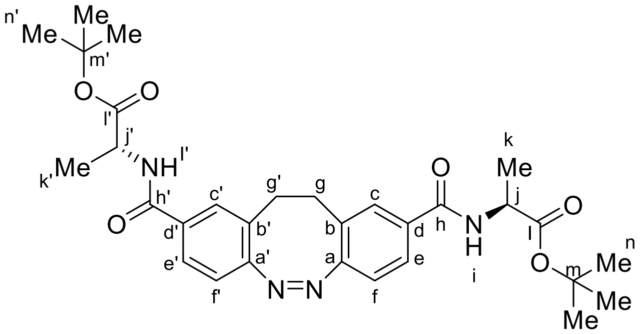

Triethyl amine (22.9 µL, 164 µmol, 2.20 equiv) was added to a solution of L-alanine *tert*-butyl ester hydrochloride (**9**) (29.8 mg, 164 µmol, 2.20 equiv) in dry DMF (2 mL). A solution of bis(2,5-dioxopyrrolidin-1-yl) 4,4'-(diazene-1,2-diyl)(*E*)-dibenzoate (**8b**) (36.5 mg, 74.4 µmol, 1.00 equiv) in dry DMF (1 mL) was added and the resulting mixture was stirred at 21 °C for 16 h. Water (30 mL) and ethyl acetate (15 mL) were added and the mixture was washed with brine (3 × 50 mL). The combined organic phases were dried over $MgSO_4$, filtered and the solvent removed under reduced pressure yielding the product as a yellow solid (33.2 mg, 60.2 µmol, 81%).

^{1}H NMR (600 MHz, $CDCl_3$) δ = 7.55 (dd, ^3J = 8.2 Hz, ^4J = 1.8 Hz, 1H, *H*-e), 7.48 (dd, ^3J = 8.2 Hz, ^4J = 1.8 Hz, 1H, *H*-e'), 7.44 (ad, ^4J = 1.8 Hz, 1H, *H*-c), 7.38 (ad, ^4J = 1.8 Hz, 1H, *H*-c'), 6.87 (d, ^3J = 8.2 Hz, 1H, *H*-f), 6.84 (d, ^3J = 8.2 Hz, 1H, *H*-f'), 4.57 (dp, ^3J = 19.0 Hz, 7.1 Hz, 2H, *H*-j/j'), 3.04 – 2.73 (m, 4H, *H*-g/g'), 1.48 (s, 9H, *H*-n), 1.47 (s, 9H, *H*-n') 1.43 (d, ^3J = 7.1 Hz, 3H, *H*-k), 1.38 (d, ^3J = 7.1 Hz, 3H, *H*-k') ppm.
3.04 – 2.73 (m, 4H, *H*-g/g'), 3.63 (s, br, 4H, *H*-h), 2.69 (s, 4H, *H*-g) ppm.
^{13}C{^{1}H} NMR (151 MHz, $CDCl_3$) δ = 172.8 (*C*-l), 172.6 (*C*-l'), 165.8 (*C*-h), 165.8 (*C*-h'), 157.8 (*C*-a), 157.8 (*C*-a'), 133.3 (*C*-b), 133.0 (*C*-b'), 129.2 (*C*-c), 129.2 (*C*-c'), 128.4 (*C*-d), 128.4 (*C*-d'), 126.1 (*C*-e), 125.8 (*C*-e'), 119.0 (*C*-f), 118.9 (*C*-f'), 82.5 (*C*-m), 82.4 (*C*-m'), 49.2 (*C*-j), 49.2 (*C*-j'), 31.6 (*C*-g), 31.4 (*C*-g'), 28.1 (*C*-n), 28.0 (*C*-n'), 18.8 (*C*-k), 18.7 (*C*-k') ppm.
IR (ATR): $\tilde{\nu}$ = 3306 (br), 2978 (w), 2359 (w), 1722 (m), 1641 (s), 1530 (s), 1480 (m), 1451 (m), 1368 (m), 1347 (m), 1227 (m), 1147 (s), 908 (m), 847 (m), 728 (m) cm^{-1}.
HRMS (ESI) *m/z*: $[M + Na]^+$ calcd for $[C_{30}H_{38}N_4O_6 + Na]^+$ 573.26836; found 573.26779.
Mp: 115 °C .
R$_f$ = 0.49 (*n*-hexane/DCM 1:1).

Determination of the ^{1}H NMR Yields

Reaction controls by ^{1}H NMR spectroscopy were performed using 1,3,5-trimethoxybenzene as internal standard. The molar amount of the corresponding product was calculated according to equation (1).

$$n_P = n_{IS} \times r_{P/IS} = n_{IS} \times \frac{\text{integral}_P/N_P}{\text{integral}_{IS}/N_{IS}} \quad (1)$$

In each reaction, 100 µmol of the standard was used and the integral of the three aromatic protons of the internal standard at 6.09 ppm was assigned to a value of one.

If DMSO was used as a solvent, no internal standard was used as DMSO could not be removed without an aqueous workup. If an internal standard was used, this would lead to a larger error in the procedure.

Table S3. Determination of the ^{1}H NMR Yield.

**Product**	**Peak$_P$ [ppm]**	**Integral$_P$/N$_P$**	**n$_P$ [µmol]**	**Yield [%]**
2,5-Dioxopyrrolidin-1-yl (*E*)-4-(phenyldiazenyl)benzoate (**3a**)	8.29	1.33/2	200	>95
2,5-Dioxopyrrolidin-1-yl (*E*)-3-(phenyldiazenyl)benzoate (**3b**)	8.23	1.31/2	197	>95
2,5-Dioxopyrrolidin-1-yl (*E*)-2-(phenyldiazenyl)benzoate (**3c**)	No conversion visible			
2,5-Dioxopyrrolidin-1-yl (*E*)-4-((4-hydroxyphenyl)diazenyl)benzoate (**3d**)	6.99	1.26/2	189	95
2,5-Dioxopyrrolidin-1-yl (*E*)-4-((4-aminophenyl)diazenyl)benzoate (**3e**)	6.71	1.26/2	189	95
2,5-Dioxopyrrolidin-1-yl (*E*)-4-((4-acetamidophenyl)diazenyl)benzoate (**3f**)	8.31	1.27/2	191	>95
2,5-Dioxopyrrolidin-1-yl (*E*)-4-((4-(allyloxy)phenyl)diazenyl)benzoate (**3h**)	8.26	0.91/2	137	69
2,5-Dioxopyrrolidin-1-yl (*E*)-4-((4-((9-hydroxynonyl)oxy)phenyl)diazenyl)benzoate (**3i**)	8.27	1.20/2	180	90
2,5-Dioxopyrrolidin-1-yl (*E*)-4-((2,6-dimethyl-4-(nonyloxy)phenyl)diazenyl)-3,5-dimethylbenzoate (**3j**)	7.90	0.63/4	95	95
2,5-Dioxopyrrolidin-1-yl (*E*)-4-((2,7-bis(4-(hexyloxy)phenoxy)naphthalen-1-yl)diazenyl)-3,5-dimethylbenzoate (**3k**)	7.80	1.26/4	189	95
2,5-Dioxopyrrolidin-1-yl (*E*)-4-((4-bromophenyl)diazenyl)benzoate (**3l**)	8.30	1.32/2	198	>95
2,5-Dioxopyrrolidin-1-yl (*E*)-4-((3-bromophenyl)diazenyl)benzoate (**3m**)	8.31	1.32/2	198	>95
2,5-Dioxopyrrolidin-1-yl (*E*)-4-((2-bromophenyl)diazenyl)benzoate (**3n**)	8.31	1.20/2	180	90
Bis(2,5-dioxopyrrolidin-1-yl) 4,4'-(diazene-1,2-diyl)(*E*)-dibenzoate (**5a**)	8.07	1.30/4	97.5	>95
Bis(2,5-dioxopyrrolidin-1-yl) 4,4'-(diazene-1,2-diyl)(*E*)-bis(3,5-dimethylbenzoate) (**5b**)	8.00	1.35/4	101	>95

The influence of the ligand was investigated using bis(2,5-dioxopyrrolidin-1-yl) 4,4'-(diazene-1,2-diyl)(*E*)-dibenzoate (**5a**) as model reaction.

Table S4: Screening of ligands.

**Ligand**	**Peak [ppm]**	**integral$_P$/N$_P$**	**n$_P$ [µmol]**	**Yield [%]**
1,1'-Bis(diphenylphosphino)ferrocene	8.07	0.06/4	4.50	5
2,2'-Bis(diphenylphosphino)-1,1'-binaphthyl	8.07	0.02/4	1.50	2
Di-(1-adamantyl)-*n*-butylphosphine	8.07	0.10/4	7.5	8
Tri-*tert*-butylphosphine	8.07	0.03/4	2.25	2
Xantphos	8.07	1.30/4	97.5	>95

N-Hydroxysuccinimidyl formate[5] (2)

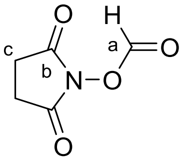

This reaction was not performed under inert conditions.

Acetic anhydride (77.0 mL, 800 µmol, 8.00 equiv) was cooled to 0 °C. Formic acid (38.0 mL, 1.00 mol, 10.0 equiv) was added over the course of 10 min and the solution was stirred at 23 °C for 2 h. 1-Hydroxypyrrolidine-2,5-dione (11.7 g, 100 µmol, 1.00 equiv) was added and the reaction mixture was stirred for further 14 h. Afterwards, the solvent was removed *in vacuo* and the resulting solid dried under reduced pressure at 8×10^{-3} mbar several hours to afford **2**[6] as a colorless solid (14.4 g, 1.00 mol, 100%, Lit.:[5] 99%).

^{1}H NMR (600 MHz, $CDCl_3$) δ = 8.18 (s, 1H, *H*-a), 2.86 (s, 4H, *H*-c) ppm.
^{13}C{^{1}H} NMR (151 MHz, $CDCl_3$) δ = 168.6 (*C*-a), 155.1 (*C*-b), 25.7 (*C*-c) ppm.
HRMS (ESI) *m/z*: $[M + Na]^+$ calcd for $[C_5H_5NO_4 + Na]^+$ 166.01108; found 166.01100.
IR (ATR): $\tilde{\nu}$ = 3002 (m), 2956 (w), 1698 (s), 1209 (m), 646 (m) cm^{-1}.
Mp: 177 °C.

(*E*)-1-(4-Iodophenyl)-2-phenyldiazene[6] (1a)

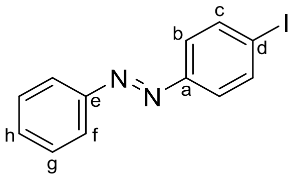

This reaction was not performed under inert conditions.

A solution of 4-iodoaniline (1.10 g, 5.00 mmol, 1.00 equiv) and nitrosobenzene (696 mg, 6.50 mmol, 1.30 equiv) in acetic acid (25 mL) was stirred for 16 h at 25 °C. The solvent was evaporated yielding **1a** as an orange solid (1.54 g, 5.00 mmol, >99%, Lit.:[6] 91%).

^{1}H NMR (600 MHz, $CDCl_3$) δ = 7.92 (d, ^{3}J = 7.1 Hz, 2H, *H*-f), 7.87 (d,^{3}J = 8.4 Hz, 2H, *H*-c), 7.66 (d, ^{3}J = 8.4 Hz, 2H, *H*-b), 7.54 – 7.48 (m, 3H, *H*-g/h) ppm.
^{13}C{^{1}H} NMR (151 MHz, $CDCl_3$) δ = 152.6 (*C*-e), 152.1 (*C*-a), 138.5 (*C*-c), 131.5 (*C*-h), 129.3 (*C*-g), 124.6 (*C*-b), 123.1 (*C*-f), 97.8 (*C*-d) ppm.
IR (ATR): $\tilde{\nu}$ = 3038 (br), 2357 (w), 2107 (w), 1809 (br), 1668 (w), 1563 (m), 1473 (s), 1438 (m), 1391 (m), 1293 (m), 1100 (m), 1049 (m), 998 (s), 915 (m), 838 (s), 768 (s), 701 (m) cm^{-1}.
HRMS (EI, 70 eV) *m/z* (%): $[M]^+$ calcd for $[C_{12}H_9N_2I]^+$ 307.98050; found 307.98043 (56), 77.0 (100).
Mp: 105 °C (Lit.:[6] 105 °C).
R_f = 0.75 (*n*-hexane/DCM 3:1).

---

6 The product was stored under dry conditions and a nitrogen atmosphere.

(*E*)-1-(3-Iodophenyl)-2-phenyldiazene[6] (**1b**)

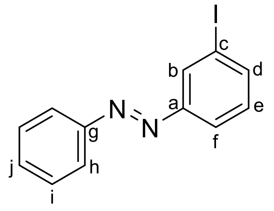

This reaction was not performed under inert conditions.

A solution of 3-iodoaniline (5.48 g, 25.0 mmol, 1.00 equiv) and nitrosobenzene (3.48 g, 32.5 mmol, 1.30 equiv) in acetic acid (150 mL) was stirred for 16 h at 25 °C. The solvent was evaporated yielding **1b** as an orange solid (7.47 g, 24.2 mmol, 97%, Lit.:[6] 96%).

^{1}H NMR (600 MHz, $CDCl_3$) δ = 8.25 (at, ^4J = 1.8 Hz, 1H, *H*-b), 7.94 – 7.90 (m, 3H, *H*-f/h), 7.80 (ddd, ^3J = 7.9 Hz, ^4J = 1.4 Hz, 1.0 Hz, 1H, *H*-d), 7.55 – 7.48 (m, 3H, *H*-i/j), 7.27 (t, ^3J = 7.9 Hz, 1H, *H*-e) ppm.
^{13}C{^{1}H} NMR (151 MHz, $CDCl_3$) δ = 153.5 (*C*-a), 152.5 (*C*-g), 140.0 (*C*-d), 131.6 (*C*-j), 130.8 (*C*-e), 130.7 (*C*-b), 129.3 (*C*-i), 123.7 (*C*-f), 123.2 (*C*-h), 94.7 (*C*-c) ppm.
IR (ATR): $\tilde{\nu}$ = 3056 (w), 1828 (br), 1560 (w), 1451 (m), 1403 (m), 1302 (w), 1203 (m), 1148 (m), 1086 (w), 1070 (w), 1051 (w), 1018 (m), 992 (w), 914 (m), 880 (m), 833 (w), 792 (s), 761 (s) cm^{-1}.
HRMS (EI, 70 eV) *m/z* (%): $[M]^+$ calcd for $[C_{12}H_9N_2I]^+$ 307.98050; found 307.98052 (57), 77.0 (100).
Mp: 71 °C (Lit.:[6] 67 °C).
R*f*: 0.75 (*n*-hexane/DCM 3:1).

(*E*)-1-(2-Iodophenyl)-2-phenyldiazene[6] (**1c**)

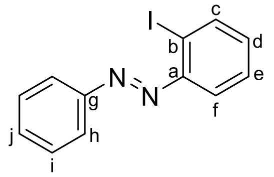

This reaction was not performed under inert conditions.

A solution of 2-iodoaniline (14.2 g, 65.0 mmol, 1.00 equiv) and nitrosobenzene (9.05 g, 84.5 mmol, 1.30 equiv) in acetic acid (400 mL) was stirred for 16 h at 40 °C. The solvent was removed under reduced pressure and the residue crystallized from methanol at -21 °C yielding **1c** as an orange solid (7.47 g, 24.2 mmol, 28%, Lit.:[6] 25%).

^{1}H NMR (600 MHz, $CDCl_3$) δ = 8.04 (dd, ^3J = 7.9 Hz, ^4J = 1.3 Hz, 1H, *H*-c), 8.01 (d, ^3J = 8.2 Hz, 2H, *H*-h), 7.64 (dd, ^3J = 8.0 Hz, ^4J = 1.6 Hz, 1H, *H*-f), 7.57 – 7.49 (m, 3H, *H*-i/j), 7.43 (ddd, ^3J = 7.9 Hz, 7.2 Hz, ^4J = 1.3 Hz, 1H, *H*-e), 7.17 (ddd, ^3J = 7.9 Hz, 7.2 Hz, ^4J = 1.6 Hz, 1H, *H*-d) ppm.
^{13}C{^{1}H} NMR (151 MHz, $CDCl_3$) δ = 152.5 (*C*-g), 151.5 (*C*-a), 140.0 (*C*-c), 132.3 (*C*-d), 131.7 (*C*-j), 129.3 (*C*-i), 129.1 (*C*-e), 123.7 (*C*-h), 117.5 (*C*-f), 102.6 (*C*-b) ppm.
IR (ATR): $\tilde{\nu}$ = 3055 (w), 1887 (br), 1572 (w), 1562 (m), 1489 (w), 1446 (m), 1419 (w), 1303 (w), 1249 (w), 1220 (w), 1142 (m), 1015 (s), 999 (w), 948 (w), 920 (w), 771 (s), 708 (s) cm^{-1}.
HRMS (EI, 70 eV) *m/z* (%): $[M]^+$ calcd for $[C_{12}H_9N_2I]^+$ 307.98050; found 307.98052 (72), 77.0 (100).
Mp: 61 °C (Lit.:[6] 59 °C).
R*f*: 0.70 (*n*-hexane/DCM 3:1).

(*E*)-4-((4-Iodophenyl)diazenyl)phenol[7] (**1d**)

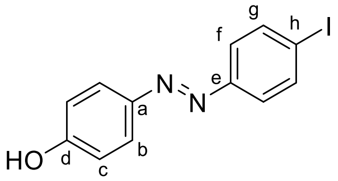

This reaction was not performed under inert conditions.

4-Iodoaniline (2.00 g, 9.13 mmol, 1.20 equiv) was dissolved in a mixture of acetone:water (1:1, 25 mL) and cooled to 0 °C. Subsequently, conc. HCl (1.15 mL, 1.50 equiv) was slowly added to the solution over the course of 10 min and left to stir for another 10 min. Sodium nitrite (735 mg, 10.6 mmol, 1.40 equiv) dissolved in water (6 mL) was added slowly over the course of 3 min and the reaction mixture was stirred for 30 min. Meanwhile, a second mixture with phenol (716 mg, 7.61 mmol, 1.00 equiv), Na_2CO_3 (1.61 g, 15.2 mmol, 2.00 equiv) and NaOH (1.22 g, 30.4 mmol, 4.00 equiv) dissolved in acetone/water (1:1, 25 mL) was cooled to 0 °C. The first prepared solution was slowly added to the solution containing the phenol over the course of 5 min. The resulting mixture was stirred for 12 h while slowly warmed to 21 °C. Subsequently, the mixture was diluted with a HCl solution (1 M, 30 mL). The precipitate that formed was removed by filtration and washed with water (2 × 10 mL). The precipitate was crystallized from MeOH at -21 °C to give **1d** as a yellow solid (2.39 g, 7.38 mmol, 97%, Lit.:[7]81%).

^{1}H NMR (600 MHz, $CDCl_3$) δ = 7.87 (d, ^3J = 8.9 Hz, 2H, *H*-g), 7.84 (d, ^3J = 8.7 Hz, 2H, *H*-c), 7.61 (d, ^3J = 8.7 Hz, 2H, *H*-b), 6.94 (d, ^3J = 8.9 Hz, 2H, *H*-f), 5.11 (s, 1H, O*H*-d) ppm.
^{13}C{^{1}H} NMR (125 MHz, $CDCl_3$) δ = 158.41 (*C*-d), 151.99 (*C*-e), 147.06 (*C*-a), 138.27 (*C*-g), 125.16 (*C*-c), 124.24 (*C*-b), 115.86 (*C*-f), 96.82 (*C*-h) ppm.
HRMS (EI, 70 eV) *m/z* (%): $[M]^+$ calcd for $[C_{12}H_9IN_2O]^+$ 323.97541; found 323.97570 (45), 93.0 (100).
IR: $\tilde{\nu}$ = 3024 (b), 1590 (s), 1562 (w), 1466 (s), 1438 (m), 1298 (s), 1240 (m), 1152 (s), 1140 (s), 1101 (m), 1051 (w), 1002 (w), 829 (s), 724 (s), 704 (m) cm^{-1}.
Mp: 168 °C.
R*f*: 0.50 (*n*-hexane/ethyl acetate 4:1).

(*E*)-4-((4-Iodophenyl)diazenyl)aniline (**1e**)

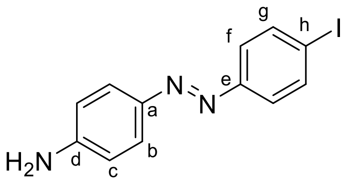

To (*E*)-*N*-(4-((4-Iodophenyl)diazenyl)phenyl)acetamide (365 mg, 1.00 mmol, 1.00 equiv.) was added MeOH (15 mL) and HCl (3 M, 75 mL). The suspension was stirred for 18 h at 85 °C, resulting in a clear red solution. The mixture was neutralized with a saturated aqueous $NaHCO_3$ solution and the precipitate was filtered. The precipitate was washed with water (2 × 20 mL) and the product was crystalized from EtOH at -21 °C to obtain **1e** as a red solid (280 mg, 0.866 mmol, 87%).

^{1}H NMR (600 MHz, DMSO-*d*$_6$) δ = 7.87 (d, ^3J = 8.2 Hz, 2H, *H*-g), 7.67 (d, ^3J = 8.4 Hz, 2H, *H*-c), 7.53 (d, ^3J = 8.2 Hz, 2H. *H*-f), 6.67 (d, ^3J = 8.4 Hz, 2H, *H*-b), 6.20 (s, 2H, N*H*$_2$-d) ppm.
^{13}C{^{1}H} NMR (151 MHz, DMSO-*d*$_6$) δ = 153.74 (*C*-a/e), 152.32 (*C*-a/e), 143.13 (*C*-d), 138.54 (*C*-g), 125.94 (*C*-c), 124.15 (*C*-f), 113.8(*C*-b), 96.20 (*C*-h) ppm.

IR (ATR): $\tilde{\nu}$ = 3446 (m), 3319 (m), 3205 (m), 2667 (w), 1893 (w), 1628 (m), 1593 (s), 1504 (m), 1473 (w), 1416 (m), 1385 (s), 1304 (s), 1230 (w), 1186 (m), 1153 (m), 1138 (s), 1119 (s), 1094 (w), 1004 (w), 943 (w), 829 (s), 722 (s), 699 (w) cm^{-1}.
HRMS (EI, 70 eV) *m/z* (%): $[M]^+$ calcd for $[C_{12}H_{10}IN_3]^+$ 322.99140; found 322.99152 (10), 92.1 (100).
Mp: 162 °C.
R*f*: 0.40 (*n*-hexane/ethyl acetate 1:1).

(*E*)-*N*-(4-((4-Iodophenyl)diazenyl)phenyl)acetamide (**1f**)

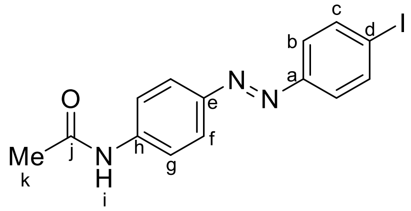

4-Aminoacetanilide (2.40 g, 16.0 mmol, 1.00 equiv) was dissolved in water (200 mL). Oxone® (19.7 g, 32 mmol, 2.00 equiv) was dissolved in water (100 mL) and added to the solution. The mixture was stirred for five minutes, while a green solid precipitated. The precipitate was filtered and the filter cake was extracted with ethyl acetate. The organic phase was dried over $MgSO_4$ and filtered. The solvent was removed and NMR analysis revealed that the nitroso compound was obtained in a high purity and a yield of 850 mg (5.18 mmol, 32%).

This product was used without further purification. 4-Nitrosoacetaniliden (820 mg, 5.00 mmol, 1.00 equiv) was dissolved in acetic acid (100 mL). To this solution, 4-iodoaniline (1.10 g, 5.00 mmol, 1.00 equiv) was added and the mixture was stirred for 1 d at 22 °C. The solvent was removed *in vacuo* and the crude product filtered through a short plug of silica (eluent: DCM). The solvent was removed and the crude product was crystallized in MeOH at -21 °C to yield **1f** as an orange solid (1.25 g, 3.42 mmol, 68%).

^{1}H NMR (600 MHz, DMSO-*d*$_6$) δ = 10.32 (s, 1H, N*H*-j), 7.95 (d, ^3J = 8.4 Hz, 2H, *H*-g), 7.88 (d, ^3J = 8.4 Hz, 2H, *H*-f), 7.80 (d, ^3J = 8.7 Hz, 2H, *H*-b), 7.63 (d, ^3J = 8.7 Hz, 2H, *H*-c), 2.10 (s, 3H, *H*-k) ppm.
^{13}C{^{1}H} NMR (151 MHz, DMSO-*d*$_6$) δ = 168.9 (*C*-j), 151.4 (*C*-h), 147.3 (*C-e*), 142.8 (*C*-a), 138.4 (*C*-g), 124.2 (*C*-f), 123.9 (*C*-b), 119.1 (*C*-c), 98.2 (*C*-d), 24.2 (*C*-k) ppm.
IR (ATR): $\tilde{\nu}$ = 3247 (w), 1658 (m), 1589 (m), 1520 (m), 1495 (m), 1473 (m), 1403 (w), 1367 (m), 1298 (m), 1264 (m), 1156 (w), 1142 (w), 1097 (w), 1037 (w), 1013 (w), 1001 (m), 835 (s), 728 (s), 701 (w) cm^{-1}.
HRMS (EI, 70 eV) *m/z* (%): $[M]^+$ calcd for $[C_{14}H_{12}IN_3O]^+$ 365.00196; found 365.00248 (25), 134.2 (100).
Mp: 230 °C.
R*f*: 0.15 (*n*-hexane/ethyl acetate 1:1).

(*E*)-1-(4-Iodophenyl)-2-(4-nitrophenyl)diazene[8] (**1g**)

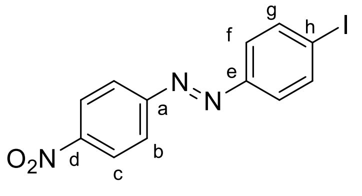

This reaction was not performed under inert conditions.

4-Amino-nitrophenol (20.0 g, 145 mmol, 1.00 equiv) was dissolved in DCM (60 mL). Oxone® (178 g, 190 mmol, 2.00 equiv) was dissolved in water (750 mL) and added to the solution. The biphasic mixture was stirred vigorously for 12 h. The phases were separated and the organic phase was dried over

$MgSO_4$ and filtered to yield 16.8 g crude product with a 60% conversion to the desired nitroso compound. This product was used without further purification.

4-Nitro-nitrosophenol (telescoped from the above reaction, 10.1 g, 66.1 mmol, 1.00 equiv) was dissolved in acetic acid (200 mL). To this solution, 4-iodoaniline (15.9 g, 72.7 mmol, 1.10 equiv) was added and the mixture was stirred for 3 d at 85 °C. The solvent was removed *in vacuo* and the crude product was crystallized two times from MeOH at -21 °C to yield **1g** 15.8 g (44.7 mmol, 68%, Lit.:[8] 51%).

^{1}H NMR (600 MHz, $CDCl_3$) δ = 8.39 (d, ^3J = 9.1 Hz, 2H, *H*-g), 8.04 (d, ^3J = 9.1 Hz, 2H, *H*-f), 7.92 (d, ^3J = 8.7 Hz, 2H, *H*-c), 7.70 (d, ^3J = 8.7 Hz, 2H, *H*-b) ppm.
^{13}C{^{1}H} NMR (125 MHz, $CDCl_3$) δ = 155.46 (*C*-h), 151.63 (*C*-e), 148.89 (*C*-a), 138.65 (*C*-c), 124.85 (*C*-b), 124.78 (*C*-g), 123.55 (*C*-h), 99.68 (*C*-d) ppm.
IR: $\tilde{\nu}$ = 1604 (m), 1514 (s), 1451 (m), 1338 (s), 1324 (m), 1139 (m), 1104 (w), 1048 (m), 1004 (m), 856 (s), 830 (s), 818 (m), 751 (s), 704 (s), 682 (m) cm^{-1}.
HRMS (EI, 70 eV) *m/z* (%): $[M]^+$ calcd for $[C_{12}H_8IN_3O_2]^+$ 352.96558; found 352.96538 (40, 76.0 (100).
Mp: 240 °C.
R$_f$: 0.40 (*n*-hexane/ethyl acetate 1:1).

(*E*)-1-(4-(Allyloxy)phenyl)-2-(4-iodophenyl)diazene[9] (1h)

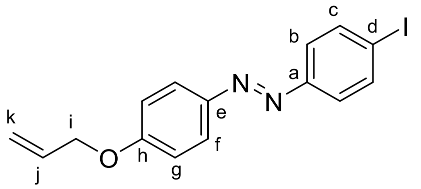

1d (1.62 g, 5.00 mmol, 1.00 equiv) and potassium carbonate (829 mg, 6.00 mmol, 1.10 equiv) were dissolved in anhydrous DMF (20 mL) under nitrogen. The solution was heated to 50 °C. Allyl bromide (1.21 g, 10.0 mmol, 2.00 equiv) in 20 mL DMF were added dropwise. The mixture was stirred at 50 °C for 17 h. After adding ethyl acetate (100 mL), the solution was washed with water (100 mL), brine (100 mL) and dried over $MgSO_4$. After filtration, the solvent was removed by evaporation under reduced pressure. The crude product was filtered through a short plug of silica and further purified by crystallization in methanol to give **1h** as a yellow solid (1.29 g, 3.55 mmol, 71% Lit.:[9] 88%).

^{1}H NMR (600 MHz, $CDCl_3$) δ = 7.90 (d, *J* = 8.9 Hz, 2H, *H*-g), 7.84 (d, *J* = 8.7 Hz, 2H, *H*-b), 7.61 (d, *J* = 8.7 Hz, 2H, *H*-c), 7.02 (d, *J* = 8.9 Hz, 2H, *H*-f), 6.12 – 6.04 (m, 1H, *H*-j), 5.45 (d, *J* = 17.3 Hz, 1H, *H*-k), 5.33 (d, *J* = 10.5 Hz, 1H, *H*-k), 4.63 (d, *J* = 6.6 Hz, 2H, *H*-i).
^{13}C{^{1}H} NMR (151 MHz, $CDCl_3$) δ 161.50 (C-h), 152.23 (C-a), 147.04 (*C*-e), 138.41 (*C*-b), 132.81 (*C*-j), 125.05 (*C*-g), 124.39 (*C*-c), 118.30 (*C*-k), 115.17 (*C*-f), 96.87 (*C*-d), 69.21 (*C*-i).
IR: $\tilde{\nu}$ = 1600 (s), 1579 (m), 1563 (m), 1492 (m), 1473 (w), 1426 (w), 1391 (w), 1362 (w), 1296 (w), 1239 (m), 1152 (m), 1141 (m), 1016 (m), 1016 (m), 993 (s), 930 (m), 838 (s), 826 (s), 727 (m), 705 (m) cm^{-1}.
HRMS (EI, 70 eV) *m/z* (%): $[M]^+$ calcd for $[C_{15}H_{13}N_2OI]^+$ 364.00671; found 364.00660 (60), 133.1 (100).
Mp: 148 °C.
R$_f$: 0.65 (*n*-hexane/ethyl acetate 1:1).

(*E*)-9-(4-((4-Iodophenyl)diazenyl)phenoxy)nonan-1-ol (**1i**)

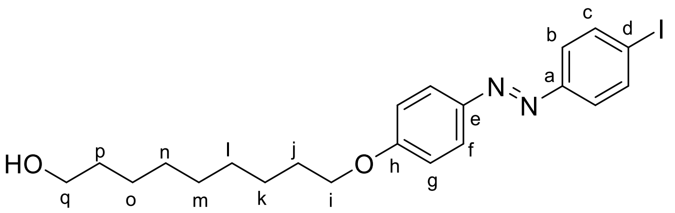

This reaction was not performed under inert conditions.

A mixture of **1d** (6.00 g, 18.5 mmol, 1.00 equiv) and 9-bromo-1-nonanol (4.95 g, 22.2 mmol, 1.20 equiv) was dissolved in acetone (100 mL). Potassium iodide (100 mg, 602 µmol, 3 mol%) and potassium carbonate (5.11 g, 37.0 mmol, 2.00 equiv) was added to the solution. The resulting solution was heated to reflux at 60 °C for 5 h. After the reaction mixture was cooled to 24 °C, water (300 mL) was added to the mixture. A precipitate was formed, which was collected by filtration. The aqueous solution was extracted with DCM (3 × 20 mL). Both the precipitate and the extract were combined and dried *in vacuo*. The resulting crude product was purified by column chromatography (gradient *n*-pentane → DCM) to give **1i** as a yellow solid (3.40 g, 7.29 mmol, 57%).

^{1}**H NMR** (600 MHz, $CDCl_3$) δ = 7.90 (d, ^3J = 9.0 Hz, 2H, *H*-g), 7.83 (d, ^3J = 8.7 Hz, 2H, *H*-c), 7.61 (d, ^3J = 8.7 Hz, 2H, *H*-b), 6.99 (d, ^3J = 9.0 Hz, 2H, *H*-f), 4.04 (t, ^3J = 6.5 Hz, 2H, *H*-i), 3.65 (t, ^3J = 6.6 Hz, 2H, *H*-q), 1.86-1.77 (m, 2H, *H*-j), 1.58 (p, ^3J = 6.6 Hz, 2H, *H*-p), 1.48 (m, 2H, C2-*H*-k), 1.35 (m, 8H, *H*-l,m,n,o) ppm.
^{13}C{^{1}H} NMR (125 MHz, $CDCl_3$) δ = 162.00 (*C*-h), 152.10 (*C*-e), 146.67 (*C*-d)), 138.23 (*C*-c), 124.92 (*C*-g), 124.20 (*C*-b), 114.76 (*C*-f)), 96.59 (*C*-d), 68.37 (*C*-i), 63.07 (*C*-q), 32.78 (*C*-p), 29.50 (*C*-m), 29.34 (*C*-n), 29.29 (*C*-j), 29.16 (*C*-l), 25.99 (*C*-k), 25.72 (*C*-o) ppm.
IR: $\tilde{\nu}$ = 3298 (b), 2917 (m), 2849 (m), 1602 (s), 1582 (m), 1500 (w), 1471 (w), 1297 (s), 1251 (m), 1143 (s), 1107 (m), 1020 (m), 843 (s), 829 (s), 721 (m), 705 (w) cm^{-1}.
HRMS (EI, 70 eV) *m/z* (%): $[M]^+$ calcd for $[C_{21}H_{27}IN_2O_2]^+$ 466.11118; found 466.11064 (10), 55.0 (100).
Mp: 133 °C.
R*f*: 0.50 (*n*-pentane: DCM 1:1).

(*E*)-4-((4-Iodo-2,6-dimethylphenyl)diazenyl)-3,5-dimethylphenol (**S1**)

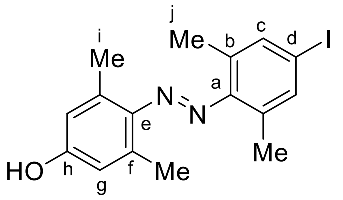

This reaction was not performed under inert conditions.

2,6-Dimethyl-4-nitroaniline (500 mg, 3.01 mmol, 1.00 equiv) and K_2CO_3 (410 mg, 3.01 mmol, 1.00 equiv) were dissolved in water (10 mL). $NaNO_2$ (205 mg, 3.01 mmol, 1.00 equiv) dissolved in water (10 mL) was added dropwise over the course of 1 min. The mixture was cooled to 0 °C and HCl (4 M, 10 mL) was slowly added dropwise over the course of 10 min forming white crystals. The dispersion was stirred for 1 h at 0 °C. The mixture was slowly added dropwise over the course of 10 min to a second solution of 3,5-dimethylphenol (400 mg, 3.27 mmol, 1.08 eq) and NaOH (5 wt%, 10 mL), which was cooled to 0 °C in beforehand. The resulting dispersion was stirred for another 21 h while warming up to 23 °C. The mixture was diluted with HCl (1 M, 20 mL). The precipitate was removed by filtration and washed with water (2 x 10 mL). The residue was crystallized in MeOH at -20 °C to yield **S1** as a dark red solid. (410 mg, 1.34 mmol, 45%).

^{1}**H NMR** (600 MHz, $CDCl_3$) *δ* = 7.52 (s, 2 H, *H*-c), 6.65 (s, 2 H, O*H*-g), 2.50 (s, 6 H, *H*-i), 2.35 (s, 6 H, *H*-j) ppm.
^{13}**C{^{1}H} NMR** (151 MHz, $CDCl_3$) *δ* = 156.2 (*C*-h), 151.5 (*C*-a), 144.6 (*C*-e), 137.9 (*C*-c), 136.0 (*C*-b), 132.9 (*C*-f), 116.1 (*C*-g), 93.1 (*C*-d), 21.0 (*C*-i), 19.4 (*C*-j) ppm.
IR: $\tilde{\nu}$ = 3418 (m) 2956 (w), 1609 (m), 1507 (s), 1466 (m), 1376 (s), 1337 (s), 1302 (s), 1217 (m), 1148 (s), 1099 (w), 941 (m), 895 (m), 800 (w), 746 (s) cm^{-1}.
HRMS (ESI): *m/z* = $[M + Na]^+$ calcd for $[C_{16}H_{17}N_3O_3 + Na]^+$ 322.11621, found 322.11633.
Mp: 190 °C.
R*f*: 0.70 (*n*-hexane/ethyl acetate 4:1).

(*E*)-1-(2,6-Dimethyl-4-(nonyloxy)phenyl)-2-(4-iodo-2,6-dimethylphenyl)diazene (**1j**)

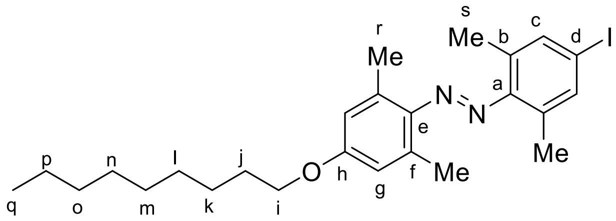

This reaction was not performed under inert conditions.

A mixture of **S1** (1.30 g, 3.42 mmol, 1.00 equiv), K_2CO_3 (886 mg, 6.40 mmol) and 1-bromohexane (2.30 mL, 4.65 mmol, 1.36 equiv) in acetonitrile (20 mL) was stirred at 80 °C. After stirring for 24 h, the solvent was removed. DCM was added and washed with HCl (1 M, 20 mL), water (20 mL), sat. $NaHCO_3$ (20 mL) and brine (20 mL). The organic phase was dried over $MgSO_4$ and filtered. The solvent was removed in vacuo and the residue was purified by column chromatography to afford **1j** as a red oil, which solidified after several hours. (1.02 g, 2.13 mmol, 62%).

^{1}**H NMR** (600 MHz, $CDCl_3$) *δ* = 7.49 (s, 2H, *H*-c), 6.68 (s, 2H, *H*-g), 4.01 (t, ^{3}J = 6.6 Hz, 2H *H*-i), 2.50 (s, 6H, *H*-r), 2.33 (s, 6H, *H*-s), 1.83 – 1.78 (m, 2H, *H*-j), 1.47 (p, ^{3}J = 7.5, 7.1 Hz, 2H, *H*-k), 1.39 – 1.25 (m, 12H, *H*-l-p), 0.90 (d, ^{3}J = 7.2 Hz, 3H, *H*-q) ppm.
^{13}**C{^{1}H} NMR** (151 MHz, $CDCl_3$) *δ* = 159.79 (*C*-h), 151.73 (*C*-a), 144.41 (*C*-e), 138.00 (*C*-c), 135.88 (*C*-f), 133.02 (*C-b*), 115.33 (*C*-g), 93.11 (*C*-d), 68.21 (*C*-i), 32.03 (*C*-l-p), 29.69 (*C*-l-p), 29.53 (*C*-l-p), 29.42 (*C*-l-p), 29.38 (*C*-j), 26.17(*C*-k), 22.83 (*C*-l-p), 21.39 (*C*-r), 19.47 (*C*-s), 14.27 (*C*-q) ppm
IR (ATR): $\tilde{\nu}$ = 2966 (s), 2935 (s), 2921 (m), 2850 (w), 1599 (m), 1588 (w), 1487 (w), 1487 (m), 1462 (m), 1454 (s), 1375 (w), 1311 (s), 1245 (w), 1168 (m), 1147 (s), 1127 (m), 1064 (s), 1036 (m), 877 (m), 863 (s), 854 (s), 825 (m), 779 (w), 747 (m), 730 (w) cm^{-1}.
HRMS (EI, 70 eV) *m/z* (%): $[M]^+$ calcd for $[C_{25}H_{35}N_2OI]^+$ 506.17886; found 506.17900 (10), 43.1 (100).
Mp: 66 °C.
R*f*: 0.70 (*n*-hexane/ethyl acetate 1:1).

(*E*)-1-((4-Iodo-2,6-dimethylphenyl)diazenyl)naphthalene-2,7-diol (**S2**)

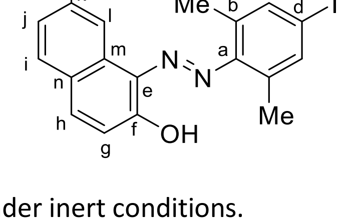

This reaction was not performed under inert conditions.

4-Iodo-2,5-dimethylanilin (2.47 g, 10 mmol, 1.00 equiv) and K_2CO_3 (1.26 g, 10.00 mmol, 1.00 equiv) were dissolved in water (10 mL). $NaNO_2$ (680 mg, 10.0 mmol, 1.00 equiv) dissolved in water (10 mL)

was added dropwise. The mixture was cooled to 0 °C under constant stirring and HCl (4 M, 10 mL) was added dropwise. The mixture was stirred for 1h and was then added dropwise to a mixture of 2,7-dihydroxynaphthalene (1.60 g, 10.0 mmol, 1.00 equiv) and 15 wt% NaOH solution (10 mL). The reaction was stirred for a further 3 h while warming up to 21 °C. The mixture was diluted with HCl (1 M, 50 mL). The precipitate was removed by filtration and washed with water (2 × 10 mL). The residue was crystallized from ethanol/water (5:1) at -21 °C to yield **S2** as a dark red solid (3.49 g, 8.70 mmol, 87%).

^{1}H NMR (600 MHz, DMSO-d_6) δ = 10.14 (s, 2H, O*H*-f/k), 7.83 (d, ^3J = 9.3 Hz, 1H, *H*-j), 7.66 (s, 2H, *H*-c), 7.66 (s, 1H, H-l), 7.63 (d, 1H, ^3J = 8.5 Hz, *H*-g), 6.91 (dd, ^3J = 8.5, ^4J = 2.4 Hz, 1H, *H*-h), 6.67 (d, ^3J = 9.3 Hz, 1H, *H*-i), 2.51 (s, 9H, *H*-o) ppm.
^{13}C{^{1}H} NMR (151 MHz, DMSO-d_6) δ = 169.88 (*C*-k), 158.80 (*C*-f), 141.20 (*C*-a), 140.27 (*C*-j), 138.09 (*C*-c), 135.18 (*C*-e), 132.01 (*C*-b), 130.98 (*C*-g), 130.03 (*C*-m), 121.33 (*C*-n), 120.12 (*C*-i), 115.71 (*C*-h), 105.15 (*C*-l), 92.64 (*C*-d), 19.47 (*C*-o) ppm.
IR (ATR): $\tilde{\nu}$ = 3137 (br), 1621 (m), 1548 (m), 1506 (s), 1479 (s), 1460 (s), 1418 (m), 1356 (w), 1331 (s), 1271 (m), 1242 (m), 1216 (m), 1198 (m), 1163 (m), 1141 (w), 1019 (w), 946 (w), 888 (m), 875 (m), 833 (s), 813 (m) cm^{-1}.
HRMS (EI): *m/z* = [M]$^+$ calcd for [$C_{18}H_{15}IN_2O_2$]$^+$ 418.01728, found 418.01727 (30), 159.1 (100).
Mp: 232 °C.
R$_f$: 0.50 (*n*-hexane/ethyl acetate 1:1).

(*E*)-1-((4-Iodo-2,6-dimethylphenyl)diazenyl)naphthalene-2,7-diyl bis(4-(hexyloxy)benzoate) (**1k**)

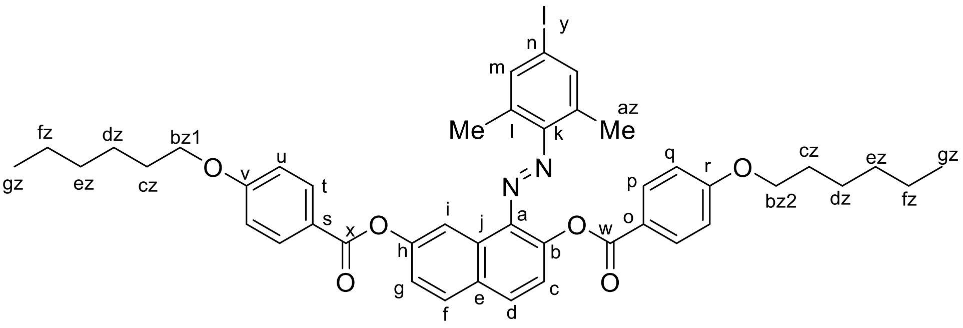

4-Hexyloxybenzoic acid (571 mg, 3.00 mmol, 3.00 equiv), **S2** (418 mg, 1.00 mmol, 1.00 equiv) and DMAP (122 mg, 1.00 mmol, 1.00 equiv) were dissolved in dry DCM (15 mL). Afterwards DCC (1.03 g, 5.00 mmol, 5.00 equiv) was added portion-wise. The mixture was stirred for 16 h. The resulting suspension was filtered, washed with a saturated $NaHCO_3$ solution (30 mL), water (30 mL), brine (30 mL). The organic phase was dried over $MgSO_4$, filtered and the solvent was removed under reduced pressure. The product was purified by column chromatography (eluent: DCM/*n*-hexane 1:1) to yield **1k** as a red solid (637 mg, 720 µmol, 72%).

^{1}H NMR (600 MHz, $CDCl_3$) δ = 8.46 (d, ^4J = 2.3 Hz, 1H, *H*-i), 8.16 (d, ^3J = 8.9 Hz, 2H, *H*-t), 8.04 (d, ^3J = 8.9 Hz, 2H, *H*-p), 7.99 (t, ^3J = 8.8 Hz, 2H, *H*-f/d), 7.46 (dd, ^3J = 8.8, 2.3 Hz, 1H, *H*-g), 7.41 (d, ^3J = 8.8 Hz, 1H, *H*-c), 7.39 (s, 2H, *H*-m), 6.07 (d, ^3J = 8.9 Hz, 2H, *H*-u), 6.90 (d, ^3J = 8.9 Hz, 2H, *H*-q), 4.05 (t, ^3J = 6.5 Hz, 2H, *H*-bz1) 4.02 (t, ^3J = 6.6 Hz, 2H, *H*-bz2), 2.22 (s, 6H, *H*-az), 1.82 (dd, ^2J = 14.8, ^3J = 6.6 Hz, 4H, *H*-cz), 1.52-1.43 (m, 4H, *H*-dz), 1.40-1.31 (m, 8H, *H*-ez/fz), 0.94 – 0.90 (m, 6H, *H*-gz) ppm.
^{13}C{^{1}H} NMR (151 MHz, $CDCl_3$) δ = 165.13 (*C*-x), 165.09 (*C*-w), 163.78 (*C*-s), 163.65 (*C*-o), 151.40 (*C*-k), 150.80 (*C*-b), 139.82 (*C*-e), 138.49 (*C*-j), 138.02 (*C*-m), 133.79 (*C*-l), 132.50 (*C*-p+t), 131.40 (*C*-f), 130.62 (*C*-d), 130.54 (*C*-h), 129.58 (*C*-a), 123.01 (*C*-c), 122.19 (*C*-g), 121.48 (*C*-v), 121.38 (*C*-r), 115.97 (*C*-i), 114.47 (*C*-u), 114.39 (*C*-q), 94.85 (*C*-n), 68.49 (*C*-bz1), 68.45 (*C*-bz2), 31.70 (*C*-fz/ez), 29.21 (*C*-cz), 25.81 (*C*-dz), 24.85 (*C*-ez), 22.74 (*C*-fz/ez), 19.10 (*C*-az), 14.18 (*C*-gz) ppm.

IR (ATR): $\tilde{\nu}$ = 2924 (m), 1721 (s), 1604 (s), 1563 (m), 1512 (m), 1469 (m), 1421 (w), 1249 (s), 1201 (s), 1217 (s), 1201 (m), 1162 (s), 1067 (s), 1006 (m), 934 (m), 916 (m), 899 (w), 875 (m), 848 (w), 759 (s), 690 (m) cm^{-1}.
HRMS (ESI): *m/z* = $[M+Na]^+$ calcd for $[C_{44}H_{47}IN_2O_6+Na]^+$ 849.23669, found 849.23711.
Mp: 232 °C.
R*f*: 0.50 (*n*-hexane/ethyl acetate 1:1).

(*E*)-1-(4-Bromophenyl)-2-(4-iodophenyl)diazene[10] (1l)

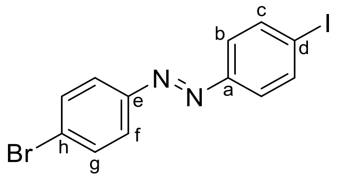

This reaction was not performed under inert conditions.

4-Bromoaniline (3.70 g, 21.5 mmol, 1.00 equiv) was dissolved in DCM (75 mL). To this solution, Oxone® (26.4 g, 43.0 mmol, 2.00 equiv) dissolved in water (300 mL) was added. The solution was stirred under a nitrogen atmosphere at 25 °C for 2 h. The color of the solution turned to green as the corresponding nitrosoarene formed. The layers were separated and the aqueous layer was extracted with DCM (3 × 100 mL). The combined organic layers were washed with HCl (1 M, 150 mL), saturated sodium hydrogen carbonate solution (150 mL), water (150 mL) and brine (150 mL). The organic phase was dried over $MgSO_4$ and filtered. The solvent was removed under reduced pressure. The obtained crude 1-bromo-4-nitrosobenzene (4.00 g, 21.5 mmol, 1.00 equiv) was dissolved in acetic acid (100 mL) and 4-iodoaniline was added (4.71 g, 21.5 mmol, 1.00 equiv). The resulting mixture was stirred at 25 °C for 14 h. The precipitate was separated by filtration and washed with acetic acid (100 mL). The residue was crystallized from methanol at -21 °C yielding **1l** as an orange solid (6.63 g, 17.1 mmol, 80%, Lit.:[10] 86%).

^{1}H NMR (600 MHz, $CDCl_3$) δ = 7.87 (d, ^3J = 8.6 Hz, 2H, *H*-c), 7.79 (d, ^3J = 8.7 Hz, 2H, *H*-f), 7.65 (d, ^3J = 8.7 Hz, 2H, *H*-g), 7.64 (d, ^3J = 8.6 Hz, 2H, *H*-b) ppm.
^{13}C{^{1}H} NMR (151 MHz, $CDCl_3$) δ = 151.9 (*C*-a), 151.3 (*C*-e), 138.6 (*C*-c), 132.6 (*C*-b), 126.0 (*C*-h), 124.7 (*C*-g), 124.6 (*C*-f), 98.3 (*C*-d) ppm.
IR (ATR): $\tilde{\nu}$ = 2355 (w), 2093 (br), 1904 (w), 1652 (w), 1563 (m), 1470 (m), 1395 (m), 1280 (w), 1222 (w), 1143 (w), 1095 (m), 1061 (m), 1001 (s), 831 (s), 773 (s), 709 (s) cm^{-1}.
HRMS (EI, 70 eV) *m/z* (%): $[M]^+$ calcd for $[C_{12}H_8BrIN_2]^+$ 385.89101; found 385.89136 (45), 76.0 (100).
Mp: 205 °C (Lit.:[6] 201 °C).
R*f*: 0.75 (*n*-hexane/DCM 3:1).

(*E*)-1-(3-Bromophenyl)-2-(4-iodophenyl)diazene (1m)

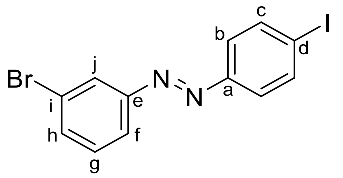

This reaction was not performed under inert conditions.

3-Bromoaniline (6.00 g, 35.0 mmol, 1.00 equiv) was dissolved in DCM (75 mL). To this solution, Oxone® (43.1 g, 75.0 mmol, 2.00 equiv) dissolved in water (300 mL) was added. The solution was stirred under a nitrogen atmosphere at 25 °C for 6 h. The color of the solution turned to green as the corresponding nitrosoarene formed. The layers were separated and the aqueous layer was extracted with DCM (3 ×

100 mL). The combined organic layers were washed with HCl (1 M, 150 mL), saturated sodium hydrogen carbonate solution (150 mL), water (150 mL) and brine (150 mL). The organic phase was dried over $MgSO_4$ and filtered. The solvent was removed under reduced pressure. The obtained crude 1-bromo-3-nitrosobenzene (6.51 g, 35.0 mmol, 1.00 equiv) was dissolved in acetic acid (100 mL) and 4-iodoaniline was added (7.67 g, 35.0 mmol, 1.00 equiv). The resulting mixture was stirred at 25 °C for 16 h. The precipitate was separated by filtration and washed with acetic acid (100 mL). The residue was crystallized from methanol at -21 °C yielding **1m** as an orange solid (9.65 g, 24.9 mmol, 72%).

^{1}H NMR (600 MHz, $CDCl_3$) δ = 8.05 (at, ^4J = 1.9 Hz, 1H, *H*-j), 7.88 – 7.86 (m, 3H, *H*-c/h), 7.65 (d, ^3J = 8.6 Hz, 2H, *H*-b), 7.61 (ddd, ^3J = 7.9 Hz, ^4J = 1.9 Hz, 1.0 Hz, 1H, *H*-f), 7.41 (t, ^3J = 7.9 Hz, 1H, *H*-g) ppm.
^{13}C{^{1}H} NMR (151 MHz, $CDCl_3$) δ = 153.5 (*C*-e), 151.8 (*C*-a), 138.6 (*C*-c), 134.1 (*C*-f), 130.7 (*C*-g), 124.8 (*C*-j), 124.8 (*C*-b), 123.3 (*C*-h), 123.3 (*C*-i), 98.6 (*C*-d) ppm.
IR (ATR): $\tilde{\nu}$ = 2990 (w), 2356 (w), 2120 (w), 1893 (w), 1652 (w), 1563 (m), 1465 (m), 1446 (m), 1409 (m), 1387 (m), 1297 (m), 1209 (w), 1170 (m), 1147 (m), 1089 (m), 1050 (m), 1000 (s), 921 (m), 879 (m), 833 (s), 819 (s), 790 (s), 775 (m), 710 (s), 703 (s) cm^{-1}.
HRMS (EI, 70 eV) *m/z* (%): $[M]^+$ calcd for $[C_{12}H_8BrIN_2]^+$ 385.89101; found 385.89122 (14), 76.0 (100).
Mp: 129 °C.
R$_f$: 0.75 (*n*-hexane/DCM 3:1).

(*E*)-1-(2-Bromophenyl)-2-(4-iodophenyl)diazene (1n)

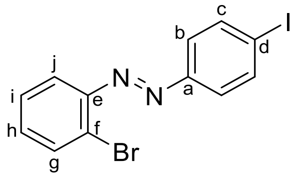

This reaction was not performed under inert conditions.

2-Bromoaniline (12.0 g, 70.0 mmol, 1.00 equiv) was dissolved in DCM (150 mL). To this solution, Oxone® (86.1 g, 140 mmol, 2.00 equiv) dissolved in water (300 mL) was added. The solution was stirred under a nitrogen atmosphere at 25 °C for 24 h. The color of the solution turned to green as the corresponding nitrosoarene formed. The layers were separated and the aqueous layer was extracted with DCM (3 × 100 mL). The combined organic layers were washed with HCl (1 M, 150 mL), saturated sodium hydrogen carbonate solution (150 mL), water (150 mL) and brine (150 mL). The organic phase was dried over $MgSO_4$ and filtered. The solvent was removed under reduced pressure. The obtained crude 1-bromo-2-nitrosobenzene (13.0 g, 70.0 mmol, 1.00 equiv) was dissolved in acetic acid (200 mL) and 4-iodoaniline was added (15.3 g, 70.0 mmol, 1.00 equiv). The resulting mixture was stirred at 25 °C for 16 h. The precipitate was separated by filtration and washed with acetic acid (100 mL). The residue was crystallized from methanol at -21 °C yielding **1n** as an orange solid (19.6 g, 50.6 mmol, 72%).

^{1}H NMR (600 MHz, $CDCl_3$) δ = 7.88 (d, ^3J = 8.6 Hz, 2H, *H*-c), 7.76 (dd, ^3J = 7.9 Hz, ^4J = 1.4 Hz, 1H, *H*-g), 7.70 (d, ^3J = 8.6 Hz, 2H, *H*-b), 7.68 (dd, ^3J = 7.9 Hz, ^4J = 1.7 Hz, 1H, *H*-j), 7.39 (td,^3J = 7.2 Hz, ^4J = 1.4 Hz, 1H, *H*-i), 7.33 (td, ^3J = 7.2 Hz, ^4J = 1.7 Hz, 1H, *H*-h) ppm.
^{13}C{^{1}H} NMR (151 MHz, $CDCl_3$) δ = 152.1 (*C*-a), 149.6 (*C*-e), 138.6 (*C*-c), 134.0 (*C*-g), 132.4 (*C*-h), 128.2 (*C*-i), 126.2 (*C*-f), 125.1 (*C*-b), 117.9 (*C*-j), 98.7 (*C*-d) ppm.
IR (ATR): $\tilde{\nu}$ = 2356 (w), 2121 (w), 1821 (w), 1660 (w), 1576 (w), 1564 (m), 1475 (w), 1456 (m), 1422 (m), 1392 (w), 1225 (w), 1044 (m), 1024 (s), 1003 (s), 953 (m), 831 (s), 759 (s), 717 (s), 702 (m) cm^{-1}.
HRMS (EI, 70 eV) *m/z* (%): $[M]^+$ calcd for $[C_{12}H_8BrIN_2]^+$ 385.89101; found 385.89146 (36), 76.0 (100).
Mp: 93 °C.
R$_f$: 0.75 (*n*-hexane/DCM 3:1).

(E)-1,2-Bis(4-iodophenyl)diazene[11] (4a)

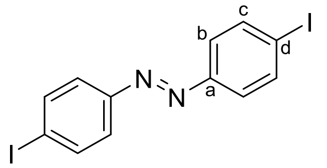

This reaction was not performed under inert conditions.

A solution of 4-iodoaniline (10.0 g, 45.7 mmol, 1.00 equiv) and manganese dioxide (70.0 g, 805 mmol, 17.6 equiv) in toluene (700 mL) was stirred for 3 h at 120 °C. The warm reaction mixture was filtered through a short plug of silica and washed with toluene (400 mL). The solvent was removed under reduced pressure yielding **4a** as a red solid (9.43 g, 21.7 mmol, %, Lit.:[11] 61%[7]).

^{1}H NMR (600 MHz, $CDCl_3$) δ = 7.87 (d, ^3J = 8.7 Hz, 4H, *H*-c), 7.64 (d,^3J = 8.7 Hz, 4H, *H*-b) ppm.
^{13}C{^{1}H} NMR (151 MHz, $CDCl_3$) δ = 151.8 (*C*-a), 138.6 (*C*-c), 124.7 (*C*-b), 98.3 (*C*-d) ppm.
IR (ATR): $\tilde{\nu}$ = 3067 (w), 2359 (w), 2111 (w), 1640 (w), 1574 (m), 1560 (m), 1470 (m), 1393 (m), 1296 (m), 1279 (m), 1096 (m), 1051 (m), 1002 (m), 834 (s), 810 (s), 774 (m), 713 (s) cm^{-1}.
HRMS (APCI) *m/z*: $[M + H]^+$ calcd for $[C_{12}H_8N_2I_2 + H]^+$ 434.88497; found 434.88462.
Mp: 244 °C (Lit.:[11] 210 °C).
R_f = 0.75 (*n*-hexane/DCM 3:1).

4-Iodo-2,6-dimethylaniline[12] (S3)

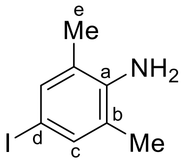

This reaction was not performed under inert conditions.

2,6-Dimethylaniline (31.5 mL, 31.0 g, 212 mmol, 1.00 equiv) was added to diethyl ether (200 mL). Iodine (59.2 g, 233 mmol, 1.10 equiv) and a sat. $NaHCO_3$ solution (600 mL) were added. The resulting biphasic mixture was stirred vigorously. After 5 h, the reaction mixture was diluted with a saturated sodium thiosulfate solution (300 mL). The phases were separated and the aqueous phase was extracted with diethyl ether (2 × 100 mL). The combined organic phases were washed with water (200 mL), dried over $MgSO_4$ and filtered. The solvent was evaporated yielding **S3** as a brown oil, which solidified upon standing at 20 °C (49.7 g, 201 mmol, 95%, Lit.:[12] 99%).

^{1}H NMR (600 MHz, CDCl3) δ = 7.24 (s, 2 H, *H*-c), 3.58 (s, 2 H, NH_2-a), 2.13 (s, 6 H, *H*-e) ppm.
^{13}C{^{1}H} NMR (151 MHz, CDCl3) δ = 142.6 (*C*-a), 136.5 (*C*-c), 124.1 (*C*-b), 79.1 (*C*-d), 17.3 (*C*-e) ppm.
IR: $\tilde{\nu}$ = 3402 (m), 3199 (m), 1625 (m), 1582 (s), 1270 (m), 1222 (w), 1034 (w), 857 (s), 730 (s) cm^{-1}.
HRMS (EI, 70 eV) *m/z* (%): $[M]^+$ calcd for $[C_8H_{10}IN]^+$ 246.98525; found 246.98532 (100).
Mp: 48°C.

[7] In this report, the product was obtained by oxidation using copper chloride.

(E)-1,2-Bis(4-iodo-2,6-dimethylphenyl)diazene[13] (4b)

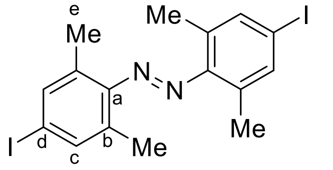

This reaction was not performed under inert conditions.

4-Iodo-2,6-dimethylaniline (**S3**) (3.00 g, 12.1 mmol, 1.00 equiv) was dissolved in toluene (300 mL). MnO_2 (26.3 g, 302 mmol, 25.0 equiv) was added and the solution was heated up to 130 °C for 3 h while vigorously stirring. After allowing the solution to cool to ambient temperature, it was filtered through a pad consisting of a layer of Celite® followed by silica. This assembly was washed with HCl (1 M, 20 mL), sat. sodium hydrogen carbonate (100 mL) and brine (100 mL). The organic phase was dried over $MgSO_4$, filtered and the solvent was evaporated. The crude product was purified by column chromatography (eluent: cyclohexane) yielding **4b** as an orange solid (593 mg, 1.21 mmol, 22%, Lit.:[13] 20%).

^{1}H NMR (500 MHz, CDCl3) δ = 7.52 (s, 4 H, *H*-c), 2.36 (s, 12 H, H-e) ppm.
^{13}C{1H} NMR (151 MHz, CDCl3) δ = 150.6 (*C*-a), 138.2 (*C*-b), 133.6 (*C*-c), 94.7 (*C*-d), 19.5 (*C*-e) ppm.
IR: $\tilde{\nu}$ = 2964 (w), 1616 (w), 1553 (m), 1435 (m), 1398 (w), 1244 (m), 1184 (w), 1033 (w), 880 (s), 864 (s), 843 (s) cm^{-1}.
HRMS (APCI): *m/z* = $[M + H]^+$ calcd for $[C_{16}H_{16}I_2N_2 + H]^+$ 490.94757, found 490.94704.
Mp: 130°C.
R$_f$: 0.40 (cyclohexane).

4-Bromo-2,6-dimethoxyaniline[14] (S4)

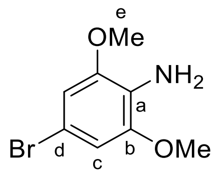

This reaction was not performed under inert conditions.

A solution of bromine (1.00 mL, 3.12 g, 1.95 mmol, 1.00 equiv) in chloroform (50 mL) was added to a solution of 2,6-dimethoxyaniline (3.06 g, 2.00 mmol, 1.03 equiv) in chloroform (50 mL) at 0°C over the course of 1 h. Afterwards, the solution was warmed up to 21 °C and stirred for additional 14 h. Then, NaOH (2 M) was added to adjust the pH to 11. The water phase was extracted with ethyl acetate (3 × 100 mL). The combined organic phases were washed with water (100 mL), brine (100 mL), dried over $MgSO_4$ and filtered. The crude product was purified by column chromatography (eluent: *n*-hexane/DCM 95:5) to yield **S4** as a colorless solid (3.66 g, 1.58 mmol, 79%, Lit.:[14] 77%)

^{1}H NMR (600 MHz, $CDCl_3$) δ = 6.65 (s, 2H, *H*-c), 3.83 (s, 6H, *H*-e), 3.77 (s, 2H, N*H$_2$*-a) ppm.
^{z13}C{^{1}H} NMR (151 MHz, $CDCl_3$) δ = 147.8 (*C*-a), 124.7 (*C*-b), 108.8 (*C*-d), 107.8 (*C*-c), 56.2 (*C*-e) ppm.
IR (ATR): $\tilde{\nu}$ = 3369 (w), 2926 (w), 1732 (w), 1604 (m), 1503 (m), 1450 (s), 1375 (m), 1297 (s), 1202 (m), 1184 (m), 1155 (s), 1054 (s), 848 (s), 811 (m), 797 (w) cm^{-1}.
HRMS (EI, 70 eV) *m/z* (%): $[M]^+$ calcd for $[C_8H_{10}NO_2Br]^+$ 230.98894; found 230.98907 (95), 233.0 (100).
Mp: 74°C (Lit: [14] 75-76°C).
R$_f$ = 0.45 (*n*-hexane: ethyl acetate 1:1).

(E)-1,2-Bis(4-bromo-2,6-dimethoxyphenyl)diazene[15] (**S5**)

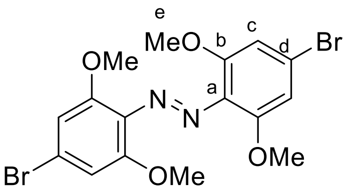

This reaction was not performed under inert conditions.

4-Bromo-2,6-dimethoxylaniline (**S4**) (2.32 g, 10.0 mmol, 1.00 equiv) was dissolved in toluene (100 mL). MnO_2 (10.9 g, 125 mmol, 12.0 equiv) was added and the solution was heated up to 130 °C for 3 h while vigorously stirring. After allowing the solution to cool to ambient temperature, it was filtered through a pad consisting of a layer of Celite® followed by silica. This assembly was washed with HCl (1 M, 20 mL), sat. sodium hydrogen carbonate (20 mL) and brine (20 mL). The organic phase was dried over $MgSO_4$, filtered and the solvent was evaporated. The crude product was purified by column chromatography (eluent: DCM) to obtain **S5** as an orange solid (1.20 g, 2.61 mmol, 52%, Lit.:[15] 20%).

^{1}H NMR (600 MHz, $CDCl_3$) δ = 6.82 (s, 4H, *H*-c), 3.84 (s, 12H, *H*-e); *Z*-Isomer: 6.60 (s, 4H, *H*-c), 3.66 (s, 12H, *H*-e) ppm.
^{13}C{^{1}H} NMR (151 MHz, $CDCl_3$) δ = 152.90 (*C*-a), 133.22 (*C*-b), 123.05 (*C*-c), 109.00 (*C*-c), 56.94 (*C*-e) ppm.
IR (ATR): $\tilde{\nu}$ = 2924 (w), 1731 (w), 1603 (s), 1564 (m), 1514 (w), 1471 (m), 1450 (m), 1398 (m), 1230 (s), 1157 (w), 1117 (s), 1015 (w), 848 (m), 830 (m), 801 (m), 757 (m). cm^{-1}.
HRMS (EI, 70 eV) *m/z* (%): $[M]^+$ calcd for $[C_{16}H_{16}N_2O_4Br_2]^+$ 457.94713; found 457.94716 (20), 243.0 (100).
Mp: 211°C.
R$_f$ = 0.50 (*n*-hexane/ethyl acetate 1:1).

(E)-1,2-Bis(4-iodo-2,6-dimethoxyphenyl)diazene (**4c**)

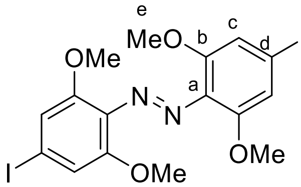

A vial was charged with 4,4´-dibromo-2,2´,6,6´-tetrakis(methoxy)-azobenzene (**S5**) (230 mg, 500 µmol, 2.00 equiv), copper iodide (9.52 mg, 50.0 µmol, 5.00 mol%) and sodium iodide (300 mg, 2.00 mmol, 4.00 equiv). *N,N'*-dimethylethylenediamine (10.8 µL, 0.100 mmol, 10.0 mol%) and dry dioxane (6 mL) were added. The reaction mixture was stirred at 110 °C for 48 h. After cooling down to 21 °C, the suspension was diluted with an aqueous sodium hydrogen carbonate solution (2 M, 50 mL), poured into water (200 mL), and extracted with DCM (3 × 150 mL). The combined organic phases were washed with hydrochloric acid (1 M, 100 mL) and brine (100 mL). After drying over $MgSO_4$ and filtration, the solvent was removed under reduced pressure. The product **4c** was obtained in 90% purity with 10% mono-brominated species left. (260 mg, 469 mmol, 94%).

^{1}H NMR (600 MHz, $CDCl_3$) δ = 7.00 (s, 4H, *H*-c), 3.83 (s, 12H, *H*-e) ppm.
^{13}C{^{1}H} NMR (151 MHz, $CDCl_3$) δ = 152.61 (*C*-a), 134.02 (*C*-b), 115.08 (*C*-c), 94.05 (*C*-d), 56.97 (*C*-e) ppm.
IR (ATR): $\tilde{\nu}$ = 2933 (w), 1558 (s), 1449 (m), 1395 (s), 1230 (s), 1158 (w), 1115 (s), 1018 (w), 828 (s), 803 (m), 742 (w) cm^{-1}.

HRMS (EI, 70 eV) *m/z* (%): $[M]^+$ calcd for $[C_{16}H_{16}I_2N_2O_4]^+$ 553.91940; found 553.91926 (25), 248.0 (100).
Mp: 265°C.
$\mathbf{R}_f$ = 0.65 (*n*-hexane/ethyl acetate 1:1).

4-Iodo-2,6-difluoro-aniline[16] (S6)

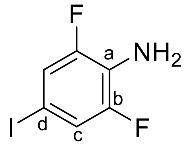

This reaction was not performed under inert conditions.

A solution of *N*-iodosuccinimide (4.95 g, 22.0 mmol, 1.10 equiv) in DMF (25 mL) was added dropwise to a solution of 2,6-difluoroaniline (2.15 g, 20.0 mmol, 1.00 equiv) and *p*-toluenesulfonic acid (760 mg, 4.00 mmol, 20 mol%) in DMF (35 mL) at 5 °C. The mixture was stirred for 2.5 h at 21 °C, diluted with ethyl acetate (250 mL), and thoroughly washed with brine (4 × 150 mL). The phases were separated. The organic phase was dried over $MgSO_4$, filtered, and concentrated under reduced pressure to give **S6** as a light brown solid (5.10 g, 19.8 mmol, 99%, Lit.:[16] 99%).

^{1}H NMR (600 MHz, $CDCl_3$) δ = 7.46 (d, ^3J = 8.0 Hz, 2H, *H*-c), 3.76 (s, 2H, NH_2-a) ppm.
^{13}C{^{1}H} NMR (151 MHz, $CDCl_3$) δ = 151.92 (dd, ^1J = 245.4 Hz, ^4J = 8.1 Hz, *C*-b), 124.3 (t, ^2J = 16.2 Hz, *C*-a), 120.5 – 120.0 (m, *C*-c), 74.28 (t, ^3J = 10.0 Hz, *C*-d) ppm.
^{19}F NMR (565 MHz, $CDCl_3$) δ -119.4 (d, ^3J = 9.2 Hz, *F*-b) ppm.
IR (ATR): $\tilde{\nu}$ = 3458 (m), 3373 (m), 1631 (s), 1593 (m), 1578 (w), 1497 (s), 1416 (s), 1288 (s), 1202 (w), 1152 (s), 1123 (w), 984 (w), 951 (s), 860 (m), 836 (s), 746 (m) cm^{-1}.
HRMS (EI, 70 eV) *m/z* (%): $[M]^+$ calcd for $[C_6H_4INF_4]^+$ 254.93498; found 254.93498 (100), 128.1 (95).
Mp: 81°C.
$\mathbf{R}_f$ = 0.70 (*n*-hexane/ethyl acetate 1:1).

(*E*)-1,2-bis(2,6-difluoro-4-iodophenyl)diazene[17] (4d)

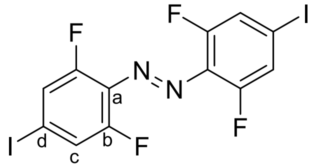

This reaction was not performed under inert conditions.

To a solution of 4-iodo-2,6-difluoroaniline (**S6**) (637 mg, 2.50 mmol, 1.00 equiv) in DCM (40 mL) was added 1,8-diazabicyclo[5.4.0]undec-7-ene (761 mg, 5.00 mmol, 2.00 equiv). The solution was stirred at 21 °C for 5 min before being cooled down to -78 °C. *N*-Chlorosuccinimide (668 mg, 5.00 mmol, 2.00 equiv) was added to the reaction mixture. The orange solution was stirred for 30 min at -78 °C, and a saturated $NaHCO_3$ solution (50 mL) was added. The layers were separated. The organic layer was washed with water (50 mL) and HCl (1 M, 50 mL). The combined organic layers were dried over $MgSO_4$, filtered and concentrated under reduced pressure. The residue was purified by silica gel flash chromatography (eluent: gradient *n*-hexane/ethyl acetate 1:0 → 1:1). **4d** was obtained as a red solid (474 mg, 937 µmol, 75%, Lit.:[17] 63%).

^{1}H NMR (600 MHz, $CDCl_3$) δ = 7.46 (d, ^3J = 8.0 Hz, 4H, *H*-c) ppm.
^{13}C{^{1}H} NMR (151 MHz, $CDCl_3$) δ = 155.00 (dd, ^1J = 267.2 Hz, ^4J = 4.4 Hz, *C*-d), 131.5 (t, ^2J = 9.3 Hz, *C*-a), 122.7 (dd, ^2J = 22.6, ^4J = 3.9 Hz, *C*-c), 94.9 (t, ^3J = 10.4 Hz, *C*-d) ppm.
^{19}F NMR (565 MHz, $CDCl_3$) δ = -119.44 (d, ^4J = 9.2 Hz, *F*-b) ppm.

IR (ATR): $\tilde{\nu}$ = 1595 (s), 1566 (s), 1445 (w), 1412 (s), 1289 (w), 1203 (m), 1046 (s), 866 (m), 855 (m), 838 (s), 747 (m) cm^{-1}.
HRMS (EI, 70 eV) *m/z* (%): $[M]^+$ calcd for $[C_{12}H_4F_4I_2N_2]^+$ 505.83946; found 505.83960 (12), 112.1 (100).
Mp: 201°C.
R*f* = 0.50 (*n*-hexane/ethyl acetate 1:1).

4-Bromo-2,6-difluoroaniline[18] (S7)

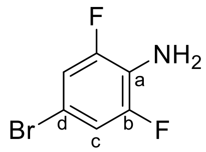

This reaction was not performed under inert conditions.

To a solution of 2,6-difluoroaniline (12.9 g, 100 mmol, 1.00 equiv) in acetonitrile (200 mL) was added a solution of *N*-bromosuccinimide (17.8 g, 100 mmol, 1.00 equiv) in acetonitrile (100 mL) at 0°C dropwise over a duration of 1h. The mixture was stirred overnight while warming up to 21 °C, and then diluted with water (800 mL). The product was extracted with ethyl acetate (3 × 100 mL). The combined organic phases were dried over $MgSO_4$, filtered, and concentrated under reduced pressure. The crude residue was purified by column chromatography over silica gel (eluent: *n*-hexane/DCM, 4:1) to yield **S7** as a colorless solid (17.2 g, 99.0 mmol, 99%, Lit.:[18] 99%).

^{1}H NMR (600 MHz, $CDCl_3$) δ = 7.02 (d, ^3J = 7.8 Hz, 2H, *H*-c), 3.73 (s, 2H, NH_2-a) ppm.
^{13}C{^{1}H} NMR (151 MHz, $CDCl_3$) *δ* = 151.76 (dd, ^1J = 244.2, ^4J = 8.7 Hz, *C*-b), 123.52 (t, ^2J = 16.1 Hz, *C*-a), 115.17 – 114.21 (m, *C*-c), 107.09 (t, ^3J = 11.6 Hz, *C*-d) ppm.
^{19}F NMR (565 MHz, $CDCl_3$) δ -130.69 (d, ^4J = 6.3 Hz, *F*-b) ppm.
Mp: 66 °C.
R*f*: 0.42 (*n*-hexane/DCM 4:1).

4-Amino-3,5-difluorobenzonitrile[19] (S8)

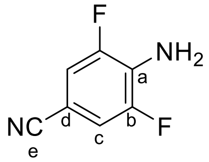

A mixture of copper(I) cyanide (29.3 g, 325 mmol, 1.70 equiv) and 4-bromo-2,6-difluoroaniline (**S7**) (39.5 g, 190 mmol, 1.00 equiv) in *N*-methyl-2-pyrrolidone (120 mL) was stirred under a nitrogen atmosphere at 204 °C for 4 h. After cooling to 30 °C, the resulting mixture was poured into aqueous ammonia until a white precipitate was formed. The suspension was extracted with toluene (5 × 100 mL). The combined organic phases were washed with water (100 mL), brine (100 mL), dried over $MgSO_4$ and filtered. The solvent was removed *in vacuo*. The crude product was dissolved in DCM and purified by column chromatography (eluent: DCM/hexane 1:1). **S8** was obtained as a colorless solid (20.5 g, 133 mmol, 70%, Lit.:[19] 87%).

^{1}H NMR (600 MHz, $CDCl_3$) *δ* =7.14 (dd, ^3J = 6.0, ^4J = 2.3 Hz, 2H, *H*-c), 4.27 (s, 2H, NH_2-a) ppm.
^{13}C{^{1}H} NMR (151 MHz, $CDCl_3$) δ 150.66 (dd, ^1J = 243.5 Hz, ^4J = 9.1 Hz, *C*-b), 129.75 (t, ^2J = 15.7 Hz, *C*-a), 118.05 (t, ^5J = 3.4 Hz, *C*-e), 115.60 (dd, ^2J = 17.5, ^4J = 7.1 Hz, *C*-c), 98.47 (t, ^3J = 11.1 Hz, *C*-d) ppm.
^{19}F NMR (565 MHz, $CDCl_3$) *δ* = -130.76 (dd, ^4J = 6.1 Hz, ^6J = 2.2 Hz, *F*-b) ppm.
IR (ATR): $\tilde{\nu}$ = 3376 (w), 3313 (w), 2224 (m), 1650 (w), 1608 (w), 1587 (m), 1523 (m), 1443 (s), 1338 (m), 1163 (s), 977 (s), 875 (s), 862 (m), 727 (s) cm^{-1}.
HRMS (EI, 70 eV) *m/z* (%): $[M]^+$ calcd for $[C_7H_4N_2F_2]^+$ 154.03371; found 154.03383 (100).

Mp: 112 °C.
R$_f$: 0.40 (*n*-hexane/DCM 1:2).

4-Amino-3,5-difluorobenzoic acid[20] (S9)

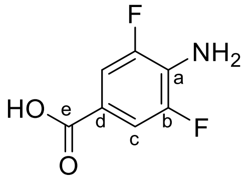

This reaction was not performed under inert conditions.

A mixture of 4-amino-3,5-difluorobenzonitrile (**S8**) (5.00 g, 32,4 mmol, 1.00 equiv) was dissolved in a 25 wt% solution of KOH in water (100 mL). The mixture was heated up to 110 °C for 19 h. After cooling down to 21 °C, the aqueous phase was acidified to pH = 2 with HCl (2 M) and extracted with ethyl acetate (2 × 100 mL). The combined organic extracts were washed with water (75 mL), brine (75 mL), dried over $MgSO_4$ and filtered. The solvent was removed under reduced pressure to obtain **S9** as a slightly yellowish solid (4.99 g, 28.8 mmol, 89%, Lit.:[20] 87%).

^{1}H NMR (600 MHz, DMSO-*d*$_6$) *δ* = δ 7.39 (dd, ^3J = 7.2 Hz, ^4J = 2.4 Hz, 2H, *H*-c), 6.04 (s, 2H, NH_2-a) ppm.
^{13}C{^{1}H} NMR (151 MHz, DMSO-*d*$_6$) *δ* = 166.12 (*C*-e), 149.80 (dd, ^1J = 239.6, ^4J = 9.5 Hz, *C*-b), 130.61 (t, ^2J = 16.7 Hz, *C*-a), 112.31 (dd, ^2J = 15.8 Hz, ^4J = 5.9 Hz, *C*-c), 59.83 (*C*-d) ppm.
^{19}F NMR (565 MHz, DMSO-*d*$_6$) δ -131.62 (d, ^4J = 7.2 Hz, *F*-b) ppm.
IR (ATR): $\tilde{\nu}$ = 3397 (w), 1683 (m), 1628 (s), 1587 (m), 1538 (w), 1454 (m), 1422 (s), 1341 (s), 1277 (s), 1243 (m), 1145 (m), 1082 (w), 953 (m), 889 (m), 765 (s), 719 (s) cm^{-1}.
HRMS (EI, 70 eV) *m/z* (%): $[M]^+$ calcd for $[C_7H_5NO_2F_2]^+$ 173.02829; found 173.02830 (75), 156.1 (100).
Mp: 171 °C.
R$_f$: 0.90 (ethyl acetate).

Ethyl 4-amino-3,5-difluorobenzoate[20] (S10)

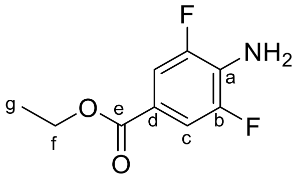

This reaction was not performed under inert conditions.

S9 (2.42 g, 14.0 mmol) was dissolved in EtOH (50 mL) and H_2SO_4 (1 mL), and refluxed for 5 h. The mixture was neutralized (pH = 7) with a saturated $NaHCO_3$ solution and extracted with DCM (3 × 50 mL). The combined organic phases were dried over $MgSO_4$, filtered, and concentrated under reduced pressure to yield **S10** as a pale brown solid (2.74 g, 13.6 mmol, 97%, Lit.:[20] 94%.)

^{1}H NMR (600 MHz, $CDCl_3$) *δ* = 7.53 (dd, *J* = 7.1, 2.2 Hz, 2H, *H*-c), 4.33 (q, ^3J = 7.1 Hz, 2H, *H*-f), 4.13 (s, 2H, NH_2-a), 1.36 (t, ^3J = 7.1 Hz, 3H, *H*-g) ppm.
^{13}C{^{1}H} NMR (151 MHz, $CDCl_3$) *δ* = 165.30 (t, ^5J = 3.5 Hz, *C*-e), 150.80 (dd, ^1J = 240.8 Hz, ^4J = 7.9 Hz, *C*-b), 128.92 (t, ^3J = 16.1 Hz, *C*-a), 118.72 (t, ^4J = 8.2 Hz, *C*-d), 113.13 – 112.08 (m, *C*-c), 61.20 (*C*-f), 14.45 (*C*-g) ppm.
^{19}F NMR (565 MHz, $CDCl_3$) δ -132.93 (dd, ^4J = 7.2 Hz, ^6J = 2.3 Hz, *F*-b) ppm.
IR (ATR): $\tilde{\nu}$ = 3505 (w), 3368 (m), 1697 (s), 1629 (s), 1585 (m), 1531 (m), 1477 (w), 1444 (m), 1397 (m), 1370 (m), 1339 (s), 1270 (s), 1222 (s), 1133 (m), 1089 (s), 1027 (s), 946 (s), 881 (m), 760 (m) cm^{-1}.
HRMS (EI, 70 eV) *m/z* (%): $[M]^+$ calcd for $[C_9H_9NO_2F_2]^+$ 201.0599; found 201.05932 (15), 156.1 (100).
R$_f$: 0.30 (*n*-hexane/DCM 1:1).

(E)-4,4'-(Diazene-1,2-diyl)bis(3,5-difluorobenzoic acid)[17] (**S11**)

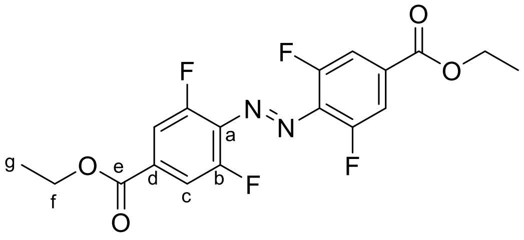

This reaction was not performed under inert conditions.

To a solution of 4-iodo-2,6-difluoroaniline (**S10**) (637 mg, 2.50 mmol, 1.00 equiv) in DCM (40 mL) was added 1,8-diazabicyclo[5.4.0]undec-7-ene (761 mg, 5.00 mmol, 2.00 equiv). The solution was stirred at 21 °C for 5 min before being cooled down to -78 °C. *N*-Chlorosuccinimide (668 mg, 5.00 mmol, 2.00 equiv) was added to the reaction mixture. The orange solution was stirred for 30 min at -78 °C, and a saturated $NaHCO_3$ solution (50 mL) was added. The layers were separated. The organic layer was washed with water (50 mL) and HCl (1 M, 50 mL). The combined organic layers were dried over $MgSO_4$, filtered and concentrated under reduced pressure. The residue was purified by silica gel flash chromatography (eluent: gradient *n*-hexane/DCM 1:0 → 1:1) to obtain **S11** as a red solid (402 mg, 1.01 mmol, 81%, Lit.:[17] 62%).

^{1}H NMR (600 MHz, $CDCl_3$) δ = 7.75 (d, ^3J = 8.9 Hz, 4H, *H*-c), 4.43 (q, ^3J = 7.1 Hz, 4H, *H*-f), 1.43 (t, ^3J = 7.1 Hz, 6H, *H*-g) ppm.
^{13}C{^{1}H} NMR (151 MHz, $CDCl_3$) δ = 163.84 (*C*-e), 155.17 (dd, ^1J = 262.6 Hz, ^4J = 3.8 Hz, *C*-b) ,134.31 (t, ^3J = 10.1 Hz, *C*-a), 133.89 (t, ^4J = 9.2 Hz, *C*-d), 114.07 (dd, ^2J = 21.7, ^4J = 3.9 Hz, *C*-c), 62.34 (*C*-f), 14.36 (*C*-g) ppm.
^{19}F NMR (565 MHz, $CDCl_3$) δ = -120.06 (d, ^4J = 10.4 Hz, *F*-b) ppm.
IR (ATR): $\tilde{\nu}$ = 1721 (m), 1573 (m), 1434 (m), 1369 (s), 1330 (m), 1238 (m), 1192 (m), 1089 (w), 1052 (s), 1017 (s), 886 (s), 767 (s), 752 (s) cm^{-1}.
HRMS (EI, 70 eV) *m/z* (%): [M]$^+$ calcd for $[C_{18}H_{14}N_2O_4F_2]^+$ 398.08842; found 398.08876 (15), 101.1 (100).
Mp: 147°C.
R*f*: 0.35 (*n*-hexane/DCM 1:1).

(E)-4,4'-(Diazene-1,2-diyl)bis(3,5-difluorobenzoic acid)[20] (**6**)

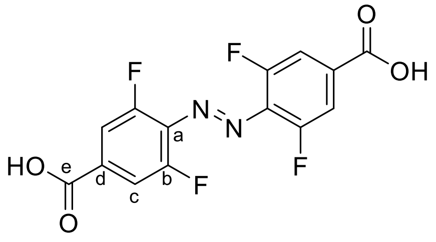

This reaction was not performed under inert conditions.

Diethyl 4,4'-(diazene-1,2-diyl)-bis(3,5-difluorobenzoate) (**S11**) (1.19 g, 3.00 mmol, 1.00 equiv) was dissolved in THF (40 mL) and KOH (673 mg, 12.0 mmol, 4.00 equiv) dissolved in H_2O (40 mL) was added. The reaction mixture was stirred for 1 h at 21 °C and acidified with HCl (1 M) until a precipitate formed. The precipitate was filtered and dried in a vacuum oven at 50°C to yield the desired product **6** (924 mg, 2.70 mmol, 90 %, Lit.:[20] 94%) as a pink solid.

^{1}H NMR (600 MHz, DMSO-d_6) δ = 7.80 (d, ^3J = 9.3 Hz, 4H, *H*-c) ppm.
^{13}C{^{1}H} NMR (151 MHz, DMSO-d_6) δ = 164.64 (*C*-e), 154.35 (dd, ^1J = 260.9 Hz, ^4J = 3.8 Hz, *C*-b), 135.46 (t, ^3J = 9.3 HZ, *C*-d), 133.00 (t, ^2J = 10.3 Hz, *C*-a), 113.97 (dd, ^2J = 21.3 Hz, ^4J = 3.6 Hz, *C*-c) ppm.

^{19}F NMR (565 MHz, DMSO-d_6) δ = -120.06 (d, ^4J = 10.4 Hz, *F*-b) ppm.
IR (ATR): $\tilde{\nu}$ = 2871 (br), 1693 (s), 1577 (s), 1481 (m), 1436 (m), 1414 (m), 1259 (s), 1195 (m), 1054 (s), 893 (s), 879 (m), 773 (s), 729 (m) cm^{-1}.
HRMS (EI, 70 eV) *m/z* (%): [M]$^+$ calcd for $[C_{14}H_6N_2O_4F_4]^+$ 342.02582; found 342.02604 (5), 101.1 (100).
Mp: >300 °C.

2-(2-(2-Aminophenethyl)phenyl)isoindoline-1,3-dione[21] (S12)

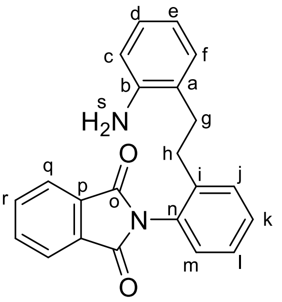

This reaction was not performed under inert conditions.

Phthalic anhydride (2.22 g, 15.0 mmol, 1.00 equiv) and 2,2'-ethylenedianiline (3.18 g, 15.0 mmol, 1.00 equiv) were dissolved in acetic acid (60 mL). The mixture was vigorously stirred for 3 h at 118 °C. After cooling down to 25 °C, water (40 mL) was added and the formed precipitate was filtered. The precipitate was recrystallized from ethanol at 78 °C. The solids were discarded and the filtrate was concentrated. The residue was re-dissolved in DCM (20 mL) and washed with water (20 mL). The aqueous phase was extracted with DCM (3 × 20 mL). Hydrogen chloride (1 M, 20 mL) was added to the combined organic layers. The obtained thick slurry was stirred for 1 h at 0 °C. The precipitate was filtered, washed with water (20 mL) and suspended in DCM (20 mL). An aqueous solution of sodium carbonate was added (2 M, 20 mL). The mixture was stirred for 1 h at 25 °C, until everything was fully dissolved. An aqueous solution of sodium hydroxide (2 M, 20 mL) was added and the phases were separated. The organic phase was dried over $MgSO_4$, filtered and concentrated yielding the product **S12** as yellow solid (1.40 g, 4.09 mmol, 41%, Lit.:[21] 52%).

^{1}H NMR (600 MHz, $CDCl_3$) δ = 7.99 – 7.92 (m, 2H, *H*-q), 7.84 – 7.78 (m, 2H, *H*-r), 7.46 – 7.33 (m, 3H, *H*-k/l/m), 7.21 (dd, ^3J = 7.5 Hz, ^4J = 1.7 Hz, 1H, *H*-j), 6.98 (ddt, ^3J = 8.2 Hz, ^4J = 4.2 Hz, 1.7 Hz, 2H, *H*-d/f), 6.66 (td (^3J = 7.5 Hz, ^4J = 1.2 Hz, 1H, *H*-e), 6.56 (dd, ^3J = 8.2 Hz, ^4J = 1.2 Hz, 1H, *H*-c), 3.54 (s, br, 2H, *H*-s), 2.84 – 2.72 (m, 4H, *H*-g/h) ppm.
^{13}C{^{1}H} NMR (151 MHz, $CDCl_3$) δ = 168.1 (*C*-o), 144.5 (*C*-b), 140.4 (*C*-n), 134.6 (*C*-r), 132.1 (*C*-p), 130.5 (*C*-l), 129.8 (*C*-i), 129.7 (*C*-k), 129.4 (*C*-f), 127.5 (*C*-m), 127.4 (*C*-d), 125.5 (*C*-l), 124.1 (*C*-a), 124.0 (*C*-q), 118.8 (*C*-e), 115.7 (*C*-c), 32.3 (*C*-g), 31.2 (*C*-h) ppm.
IR (ATR): $\tilde{\nu}$ = 3346 (w, br), 2937 (w), 2359 (w), 1708 (s), 1605 (m), 1492 (m), 1466 (m), 1454 (m), 1378 (s), 1220 (m), 1161 (m), 1109 (m), 1082 (m), 1003 (m), 888 (m), 765 (s), 724 (s) cm^{-1}.
HRMS (EI, 70 eV) *m/z* (%): [M]$^+$ calcd for $[C_{22}H_{18}N_2O_2]^+$ 342.13628; found 342.13649 (6), 106.1 (100).
Mp: 134 °C.
R$_f$ = 0.70 (*n*-hexane/DCM 1:1).

2-(2-Aminophenethyl)-4-iodoaniline[21] (S13)

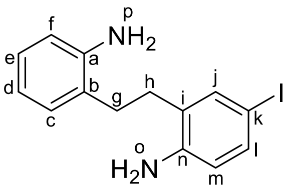

This reaction was not performed under inert conditions.

N-Iodosuccinimide (1.11 g, 7.46 mmol, 1.00 equiv) was added in one portion to a solution of 2-(2-(2-aminophenethyl)phenyl)isoindoline-1,3-dione (**S12**) (2.55 g, 7.46 mmol, 1.00 equiv) in DMSO (50 mL). After stirring the mixture for 3 h at 23 °C, brine (50 mL) was added and the mixture was extracted with DCM (3 × 30 mL). The combined organic layers were washed with water (50 mL) and brine (3 × 50 mL). The solution was filtered over a short plug of silica and the solvent removed under reduced pressure. The crude iodination product (2.61 g, 5.57 mmol) was dissolved in THF (30 mL) and hydrazine monohydrate (1.67 mL, 33.4 mmol, 6.00 equiv) was added. The mixture was stirred at 64 °C for 2 h. After cooling to 23 °C, the mixture was filtered. The solids were washed with THF (60 mL) and discarded. Water (30 mL) was added to the filtrate. The THF was removed under reduced pressure and the residue was extracted with diethyl ether (3 × 30 mL). The combined organic layers were washed with brine (30 mL), dried over $MgSO_4$ and filtered. The solvent was removed under reduced pressure. The residue was dissolved in DCM (5 mL) and precipitated with *n*-hexane (5 mL) yielding **S13** as a brownish-yellow solid (1.51 g, 4.47 mmol, 60%, Lit.:[21] 93%).

^{1}H NMR (600 MHz, $CDCl_3$) δ = 7.36 (ad, ^4J = 2.1 Hz, 1H, *H*-j), 7.30 (dd, ^3J = 8.3 Hz, ^4J = 2.1 Hz, 1H, *H*-l), 7.10 – 7.01 (m, 2H, *H*-c/e), 6.75 (td, ^3J = 7.4 Hz, ^4J = 1.2 Hz, 1H, *H*-d), 6.69 (dd, ^3J = 7.9 Hz, ^4J = 1.2 Hz, 1H, *H*-f), 6.44 (d,^3J = 8.3 Hz, 1H, *H*-m), 3.60 (s, br, 4H, *H*-o/p), 2.80 – 2.70 (m, 4H, *H*-g/h) ppm.
^{13}C{^{1}H} NMR (151 MHz, $CDCl_3$) δ = 144.3 (*C*-a), 144.3 (*C*-n), 138.0 (*C*-j), 136.0 (*C*-l), 129.7 (*C*-c), 129.1 (*C*-i), 127.6 (*C*-e), 126.0 (*C*-b), 119.3 (*C*-d), 118.0 (*C*-m), 116.1 (*C*-f), 80.2 (*C*-k), 31.1 (*C*-g), 30.7 (*C*-h) ppm.
IR (ATR): $\tilde{\nu}$ = 3353 (w, br), 3021 (w), 2927 (w), 2867 (w), 1715 (m), 1616 (s), 1494 (s), 1448 (s), 1368 (m), 1263 (s), 1159 (m), 1055 (m), 858 (m), 749 (s) cm^{-1}.
HRMS (ESI) *m/z*: $[M + H]^+$ calcd for $[C_{14}H_{15}IN_2 + H]^+$ 339.03527; found 339.03462.
Mp: 131 °C.
R$_f$ = 0.57 (*n*-hexane/DCM 1:1).

(*Z*)-2-Iodo-11,12-dihydrodibenzo[c,g][1,2]diazocine[21] (7a)

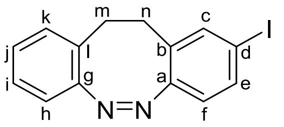

This reaction was not performed under inert conditions.

A freshly prepared solution of *meta*-chloroperoxybenzoic acid (1.44 g, 8.34 mmol, 70%, 2.00 equiv) in acetic acid (50 mL) was added over the course of 2 h under rapid stirring to a solution of 2,2'-(ethane-1,2-diyl)bis(4-iodoaniline) (**S13**) (988 mg, 2.92 mmol, 1.00 equiv) in acetic acid (100 mL) and DCM (300 mL). After the complete addition, the mixture was stirred for a further 1 h at 25 °C. The solvent was removed under reduced pressure. The crude product was purified by column chromatography on silica gel (eluent: cyclohexane) to afford **7a** as a yellow solid (680 mg, 2.04 mmol, 70%, Lit.: [21] 75%).

^{1}H NMR (600 MHz, $CDCl_3$) δ = 7.44 (dd, ^3J = 8.3 Hz, ^4J = 1.8 Hz, 1H, *H*-e), 7.34 (d, ^4J = 1.8 Hz, 1H, *H*-c), 7.16 (dd, ^3J = 7.6 Hz, ^4J = 1.4 Hz, 1H, *H*-i), 7.05 (dd, ^3J = 7.6 Hz, ^4J = 1.4 Hz, 1H, *H*-j), 7.00 (dd, ^3J = 7.6 Hz,

^4J = 1.4 Hz, 1H, *H*-k), 6.83 (dd, ^3J = 7.6 Hz, ^4J = 1.4 Hz, 1H, *H*-h), 6.58 (d,^3J = 8.3 Hz, 1H, *H*-f), 3.06 – 2.62 (m, 4H, *H*-m/n) ppm.
^{13}C{^{1}H} NMR (151 MHz, $CDCl_3$) δ = 155.5 (*C*-g), 155.0 (*C*-a), 138.4 (*C*-c), 135.8 (*C*-e), 130.7 (*C*-b), 129.9 (*C*-k), 127.6 (*C*-l), 127.5 (*C*-j), 127.1 (*C*-i), 120.9 (*C*-f), 118.9 (*C*-h), 91.9 (*C*-d)z, 31.6 (*C*-m), 31.5 (*C*-n) ppm.
IR (ATR): $\tilde{\nu}$ = 2923 (w, br), 2359 (w), 1737 (s), 1605 (m), 1511 (m), 1453 (w), 1248 (s), 1200 (s), 1163 (s), 1062 (s), 891 (m), 848 (m), 760 (m), 751 (m) cm^{-1}.
HRMS (EI, 70 eV) *m/z* (%): $[M]^+$ calcd for $[C_{14}H_{11}IN_2]^+$ 333.99615; found 333.99606 (7), 178.1 (100).
Mp: 131 °C.
R*f* = 0.60 (*n*-hexane/DCM 3:1).

2,2'-(Ethane-1,2-diyl)bis(4-iodoaniline)[21] (S14)

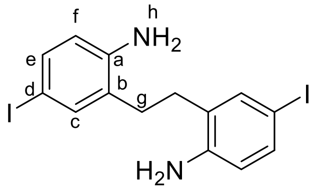

This reaction was not performed under inert conditions.

N-Iodosuccinimide (21.7 g, 96.6 mmol, 2.05 equiv) was added in three portions over 10 min to a solution of 2,2'-ethylenedianiline (10.0 g, 47.1 mmol, 1.00 equiv) in DMSO (125 mL) at 25 °C. After stirring the mixture for 23 h at 25 °C, DCM (100 mL) and water (100 mL) were added. The phases were separated, and the organic layer was washed with an aqueous solution of sodium hydrogen carbonate (2 M, 2 × 100 mL), water (100 mL) and brine (100 mL). The organic phase was dried over $MgSO_4$, filtered and the solvent removed under reduced pressure. **S14** was obtained as a red-brown solid (21.2 g, 45.7 mmol, 97%, Lit.:[21] 87%).

^{1}H NMR (600 MHz, $CDCl_3$) δ = 7.34 – 7.30 (m, 4H, *H*-e/f), 6.48 – 6.44 (m, 2H, *H*-c), 3.64 (s, br, 4H, *H*-h), 2.69 (s, 4H, *H*-g) ppm.
^{13}C{^{1}H} NMR (151 MHz, $CDCl_3$) δ = 144.2 (*C*-a), 138.0 (*C*-f), 136.2 (*C*-e), 128.6 (*C*-b), 118.1 (*C*-c), 80.3 (*C*-d), 30.6 (*C*-g) ppm.
IR (ATR): $\tilde{\nu}$ = 3397 (m, br), 3317 (m), 3219 (w), 2882 (w), 2359 (w), 1626 (m), 1562 (w), 1486 (s), 1470 (m), 1436 (m), 1398 (m), 1297 (s), 1268 (s), 1142 (m), 1096 (m), 1052 (m), 1002 (m), 931 (m), 868 (m), 834 (m), 809 (s), 709 (s) cm^{-1}.
HRMS (EI, 70 eV) *m/z* (%): $[M]^+$ calcd for $[C_{14}H_{14}I_2N_2]^+$ 463.92410; found 463.92397 (78), 232.0 (100).
Mp: 165 °C.
R*f* = 0.57 (*n*-hexane/DCM 1:1).

(*Z*)-2,9-Diiodo-11,12-dihydrodibenzo[c,g][1,2]diazocine[21] (7b)

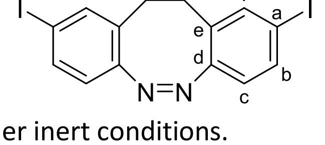

This reaction was not performed under inert conditions.

A freshly prepared solution of *meta*-chloroperoxybenzoic acid (1.75 g, 10.1 mmol, 71%, 2.00 equiv) in acetic acid (125 mL) was added over the course of 3 h under rapid stirring to a solution of 2,2'-(ethane-1,2-diyl)bis(4-iodoaniline) (**S14**) (1.67 g, 3.60 mmol, 1.00 equiv) in acetic acid (100 mL) and DCM (300 mL). After the complete addition, the mixture was stirred for a further 1 h at 25 °C. The solvent was removed under reduced pressure. The crude product was purified by column chromatography on

silica gel (eluent: cyclohexane/DCM 1:4) to afford **7b** as a yellow solid (1.23 g, 2.66 mmol, 71%, Lit.:[21] 72%).

^{1}H NMR (600 MHz, $CDCl_3$) δ = 7.48 (dd, ^{3}J = 8.3 Hz, ^{4}J = 1.8 Hz, 2H, *H*-b), 7.36 (d, ^{4}J = 1.8 Hz, 2H, *H*-f), 6.59 (d, ^{3}J = 8.3 Hz, 2H, *H*-c), 2.97 – 2.62 (m, 4H, *H*-g) ppm.
^{13}C{^{1}H} NMR (151 MHz, $CDCl_3$) δ = 154.9 (*C*-d), 138.6 (*C*-f), 136.1 (*C*-b), 130.1 (*C*-e), 120.9 (*C*-c), 92.4 (*C*-a), 31.3 (*C*-g) ppm.
IR (ATR): $\tilde{\nu}$ = 2921 (w), 2358 (w), 1575 (m), 1533 (w), 1467 (m), 1383 (w), 1280 (w), 1156 (m), 1097 (m), 1052 (w), 1002 (m), 979 (w), 959 (w), 918 (w), 891 (m), 878 (m), 801 (s), 714 (m) cm^{-1}.
HRMS (EI, 70 eV) *m/z* (%): $[M]^+$ calcd for $[C_{14}H_{10}I_2N_2]^+$ 459.89280; found 459.89287 (49), 178.1 (100).
Mp: 173 °C.
R_f = 0.60 (*n*-hexane/DCM 3:1).

References

1. *The ACS Style Guide: Effective Communication of Scientific Information*, 3rd ed.; Coghill, A.M., Garson, L.R., Eds.; American Chemical Society: Washington, DC, 2006.
2. Keiper, S.; Vyle, J.S. Reversible Photocontrol of Deoxyribozyme-Catalyzed RNA Cleavage under Multiple-Turnover Conditions. *Angew. Chem. Int. Ed.* **2006**, *45*, 3306-3309, doi:10.1002/anie.200600164.
3. Zhou, M.; Haldar, S.; Franses, J.; Kim, J.-M.; Thompson, D.H. Synthesis and Self-assembly Properties of Acylated Cyclodextrins and Nitrilotriacetic Acid (NTA)-modified Inclusion Ligands for Interfacial Protein Crystallization. *Supramol. Chem.* **2006**, *17*, 101-111, doi:10.1080/10610270412331329005.
4. Liu, D.; Xie, Y.; Shao, H.; Jiang, X. Using Azobenzene-Embedded Self-Assembled Monolayers to Photochemically Control Cell Adhesion Reversibly. *Angew. Chem. Int. Ed.* **2009**, *48*, 4406-4408, doi:10.1002/anie.200901130.
5. Barré, A.; Ţînţaş, M.-L.; Alix, F.; Gembus, V.; Papamicaël, C.; Levacher, V. Palladium-Catalyzed Carbonylation of (Hetero)Aryl, Alkenyl and Allyl Halides by Means of N-Hydroxysuccinimidyl Formate as CO Surrogate. *J. Org. Chem.* **2015**, *80*, 6537-6544, doi:10.1021/acs.joc.5b01119.
6. Strueben, J.; Lipfert, M.; Springer, J.O.; Gould, C.A.; Gates, P.J.; Sönnichsen, F.D.; Staubitz, A. High-Yield Lithiation of Azobenzenes by Tin-Lithium Exchange. *Chem. Eur. J.* **2015**, *21*, 11165-11173, doi:10.1002/chem.201500003.
7. Tiwari, V.K.; Kumar, A.; Schmidt, R.R. Disaccharide-Containing Macrocycles by Click Chemistry and Intramolecular Glycosylation. *Eur. J. Org. Chem.* **2012**, 2945-2956, doi:10.1002/ejoc.201101815.
8. Yu, B.-C.; Shirai, Y.; Tour, J.M. Syntheses of New Functionalized Azobenzenes for Potential Molecular Electronic Devices. *Tetrahedron* **2006**, *62*, 10303-10310, doi:10.1016/j.tet.2006.08.069.
9. Rasheed, O.K.; Raftery, J.; Quayle, P. A New Benzannulation Reaction of Azoaromatics. *Synlett* **2015**, *26*, 2806-2810.
10. Nguyen, T.-T.-T.; Turp, D.; Wang, D.; Nolscher, B.; Laquai, F.; Mullen, K. A Fluorescent, Shape-Persistent Dendritic Host with Photoswitchable Guest Encapsulation and Intramolecular Energy Transfer. *J. Am. Chem. Soc.* **2011**, *133*, 11194-11204, doi:10.1021/ja2022398.
11. Strueben, J.; Gates, P.J.; Staubitz, A. Tin-Functionalized Azobenzenes as Nucleophiles in Stille Cross-Coupling Reactions. *J. Org. Chem.* **2014**, *79*, 1719-1728, doi:10.1021/jo402598u.
12. Brown, D.G.; Schauer, P.A.; Borau-Garcia, J.; Fancy, B.R.; Berlinguette, C.P. Stabilization of Ruthenium Sensitizers to TiO2 Surfaces through Cooperative Anchoring Groups. *J. Am. Chem. Soc.* **2013**, *135*, 1692-1695, doi:10.1021/ja310965h.
13. Abboud, M.; Sayari, A. Novel Family of Periodic Mesoporous Organosilicas Containing Azobenzene within the Pore Walls. *Microporous Mesoporous Mater.* **2017**, *249*, 157-164, doi:10.1016/j.micromeso.2017.04.061.

14. Broser, W.; Harrer, W. Synthesen, spektroskopische Charakterisierung, Gleichgewichts- und kinetische Messungen an m-methoxylierten Parafuchsinen. *Z. Naturforsch., B: J. Chem. Sci.* **1969**, *24*, 542-547, doi:doi:10.1515/znb-1969-0514.
15. Samanta, S.; Beharry, A.A.; Sadovski, O.; McCormick, T.M.; Babalhavaeji, A.; Tropepe, V.; Woolley, G.A. Photoswitching Azo Compounds in Vivo with Red Light. *J. Am. Chem. Soc.* **2013**, *135*, 9777-9784, doi:10.1021/ja402220t.
16. Bléger, D.; Schwarz, J.; Brouwer, A.M.; Hecht, S. o-Fluoroazobenzenes as Readily Synthesized Photoswitches Offering Nearly Quantitative Two-Way Isomerization with Visible Light. *J. Am. Chem. Soc.* **2012**, *134*, 20597-20600, doi:10.1021/ja310323y.
17. Antoine John, A.; Lin, Q. Synthesis of Azobenzenes Using N-Chlorosuccinimide and 1,8-Diazabicyclo[5.4.0]undec-7-ene (DBU). *J. Org. Chem.* **2017**, *82*, 9873-9876, doi:10.1021/acs.joc.7b01530.
18. Moreno, J.; Gerecke, M.; Grubert, L.; Kovalenko, S.A.; Hecht, S. Sensitized Two-NIR-Photon *Z*→*E* Isomerization of a Visible-Light-Addressable Bistable Azobenzene Derivative. *Angew. Chem. Int. Ed.* **2016**, *55*, 1544-1547, doi:10.1002/anie.201509111.
19. Cigl, M.; Bubnov, A.; Kašpar, M.; Hampl, F.; Hamplová, V.; Pacherová, O.; Svoboda, J. Photosensitive Chiral Self-Assembling Materials: Significant Effects of Small Lateral Substituents. *J. Mater. Chem. C* **2016**, *4*, 5326-5333, doi:10.1039/C6TC01103A.
20. Heinrich, B.; Bouazoune, K.; Wojcik, M.; Bakowsky, U.; Vázquez, O. ortho-Fluoroazobenzene Derivatives as DNA Intercalators for Photocontrol of DNA and Nucleosome Binding by Visible Light. *Org. Biomol. Chem.* **2019**, *17*, 1827-1833, doi:10.1039/C8OB02343C.
21. Maier, M.S.; Hüll, K.; Reynders, M.; Matsuura, B.S.; Leippe, P.; Ko, T.; Schäffer, L.; Trauner, D. Oxidative Approach Enables Efficient Access to Cyclic Azobenzenes. *J. Am. Chem. Soc.* **2019**, *141*, 17295-17304, doi:10.1021/jacs.9b08794.

^{1}H, $^{13}C\{^{1}H\}$ and ^{19}F NMR Spectra of the Purified Compounds

2,5-Dioxopyrrolidin-1-yl (*E*)-4-(phenyldiazenyl)benzoate (**3a**)

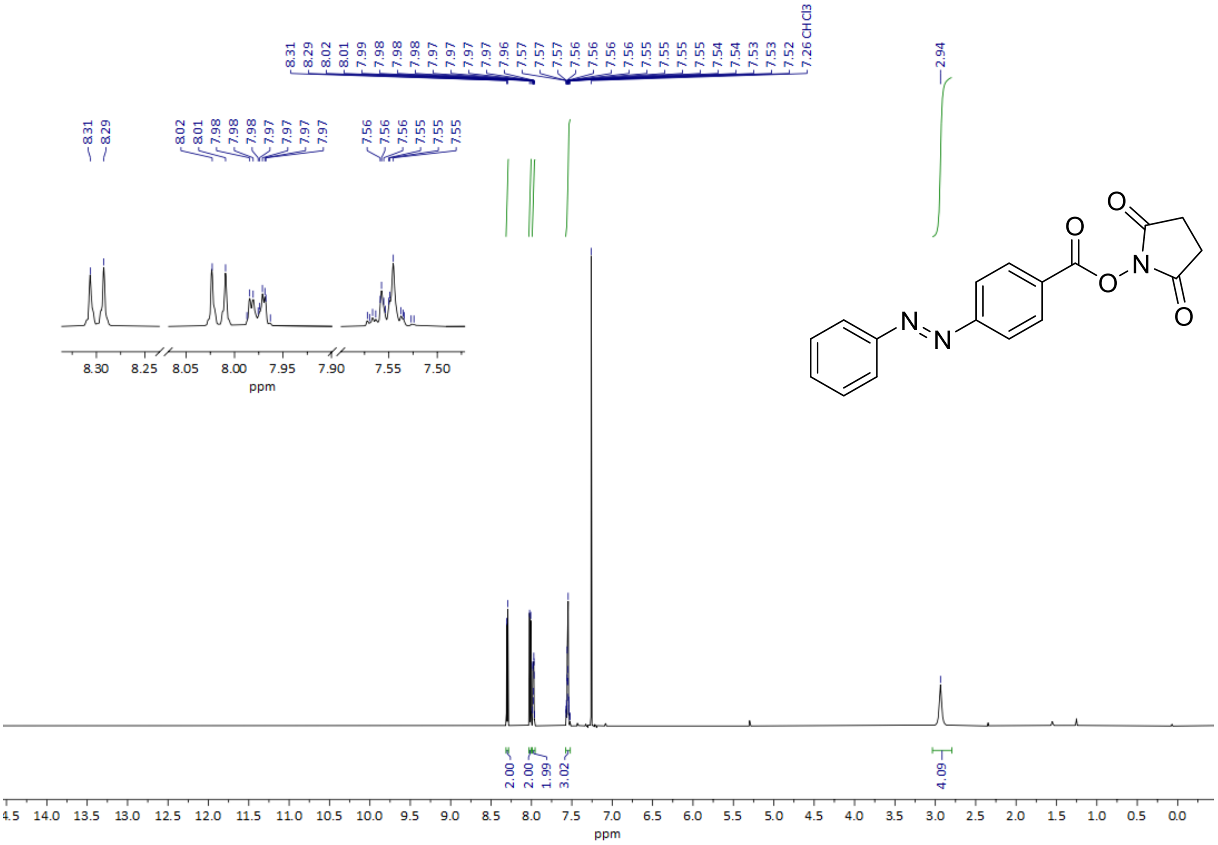

Figure S1: ^{1}H NMR spectrum of **3a** in $CDCl_3$.

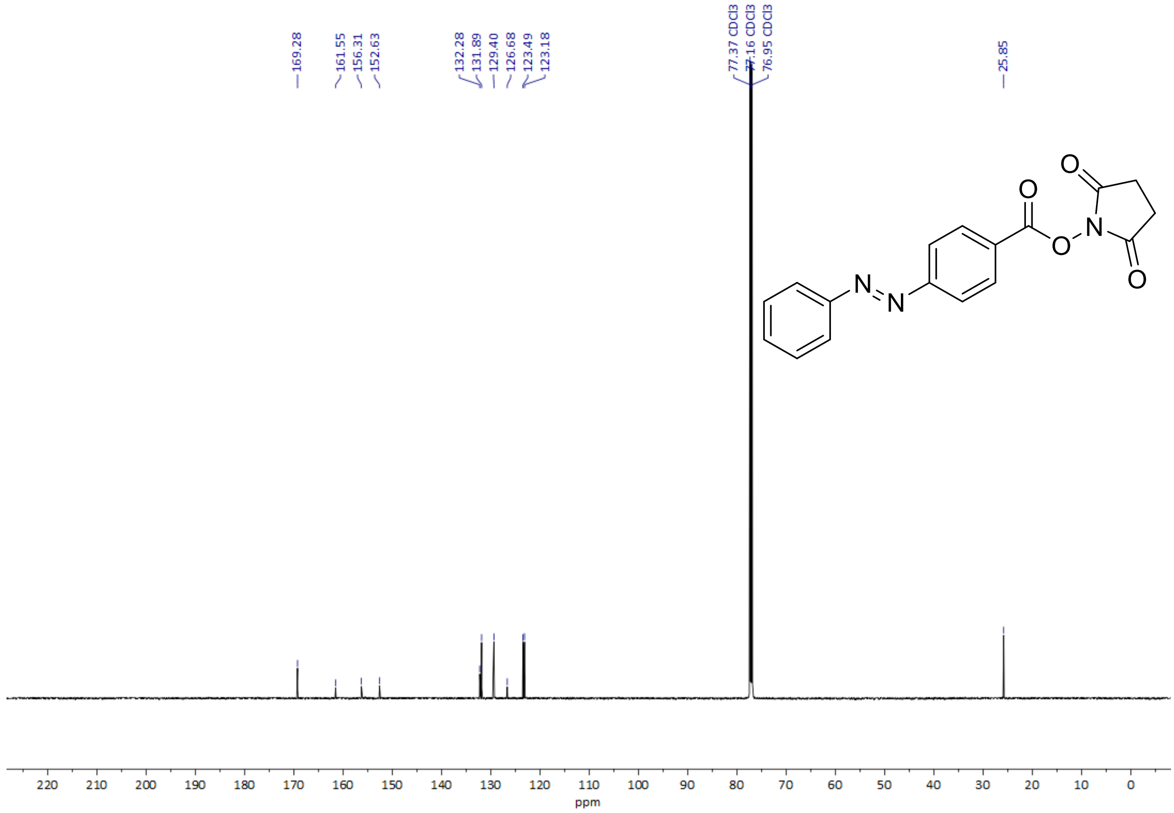

Figure S2: $^{13}C\{^{1}H\}$ NMR spectrum of **3a** in $CDCl_3$.

2,5-Dioxopyrrolidin-1-yl (*E*)-3-(phenyldiazenyl)benzoate (**3b**)

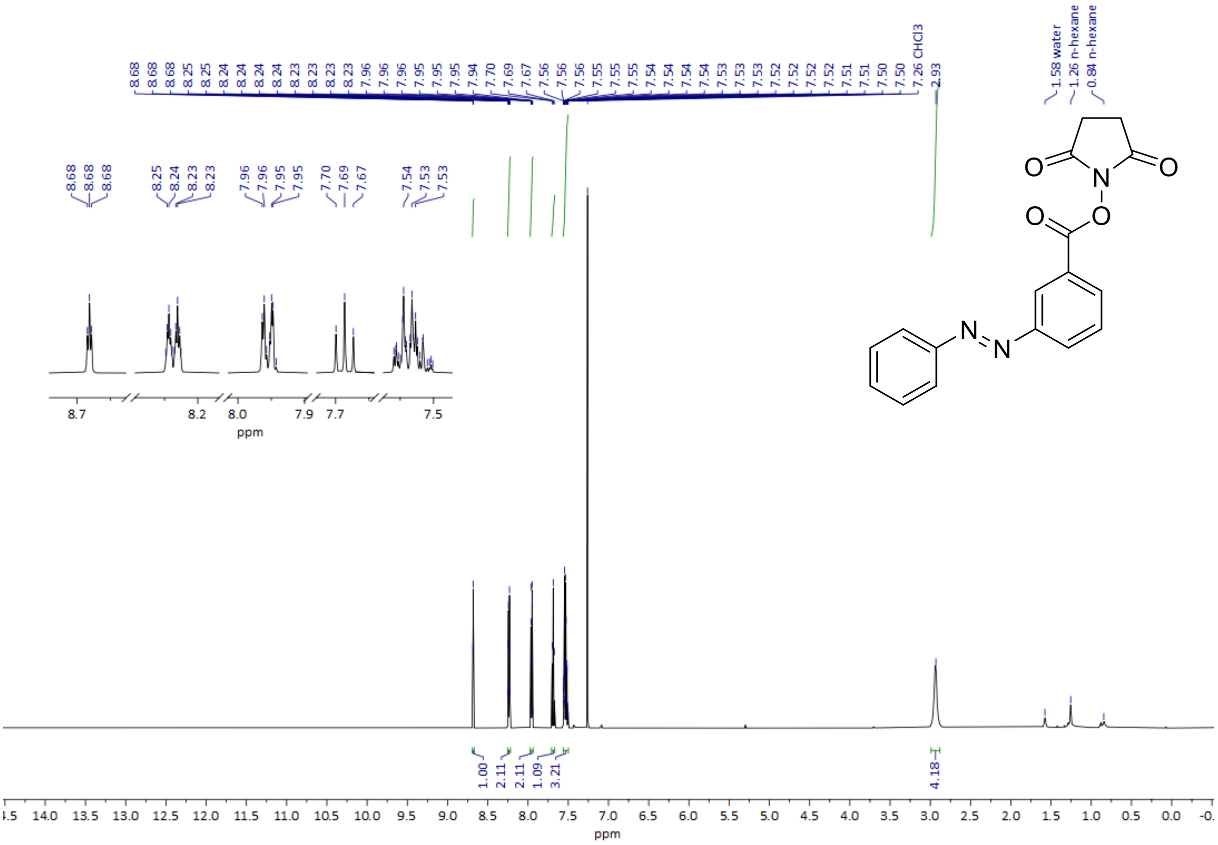

Figure S3: ^{1}H NMR spectrum of **3b** in $CDCl_3$.

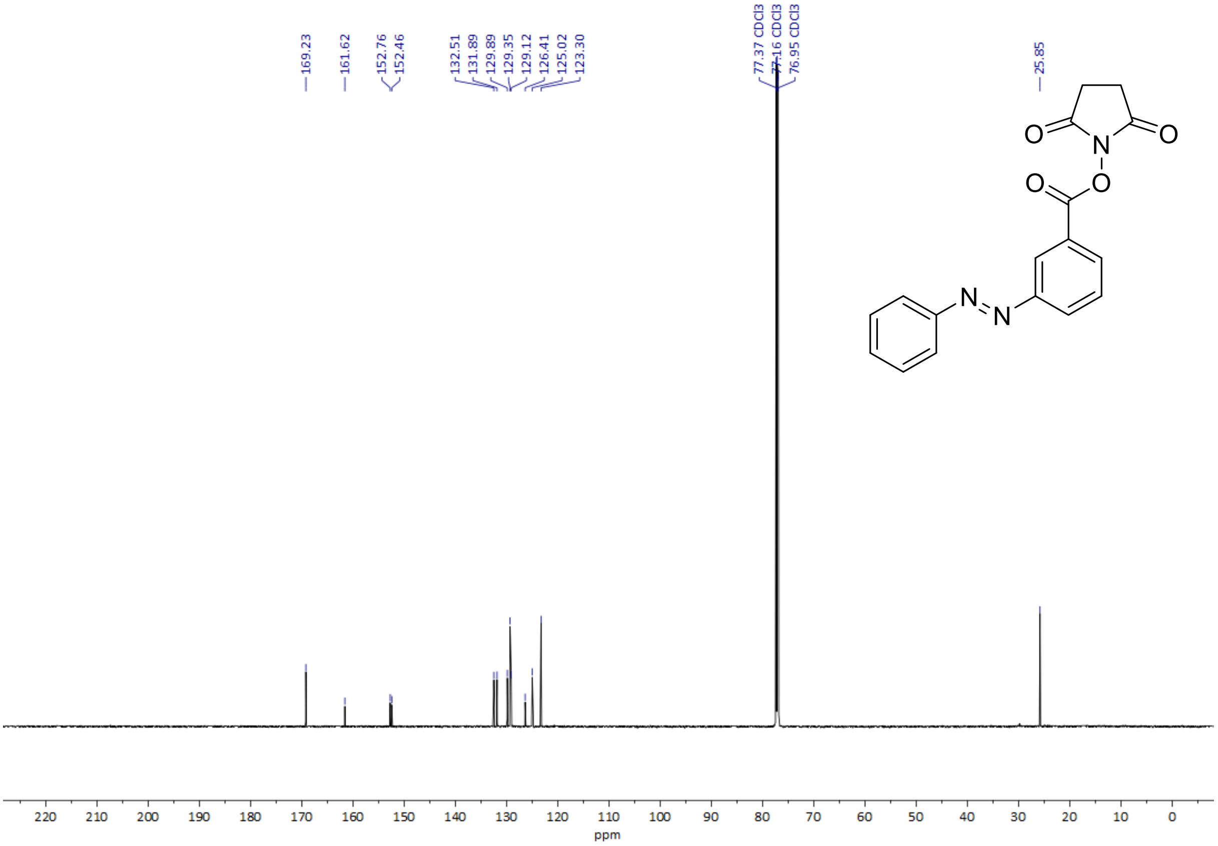

Figure S4: $^{13}C\{^{1}H\}$ NMR spectrum of **3b** in $CDCl_3$.

2,5-Dioxopyrrolidin-1-yl (*E*)-4-((4-hydroxyphenyl)diazenyl)benzoate (**3d**)

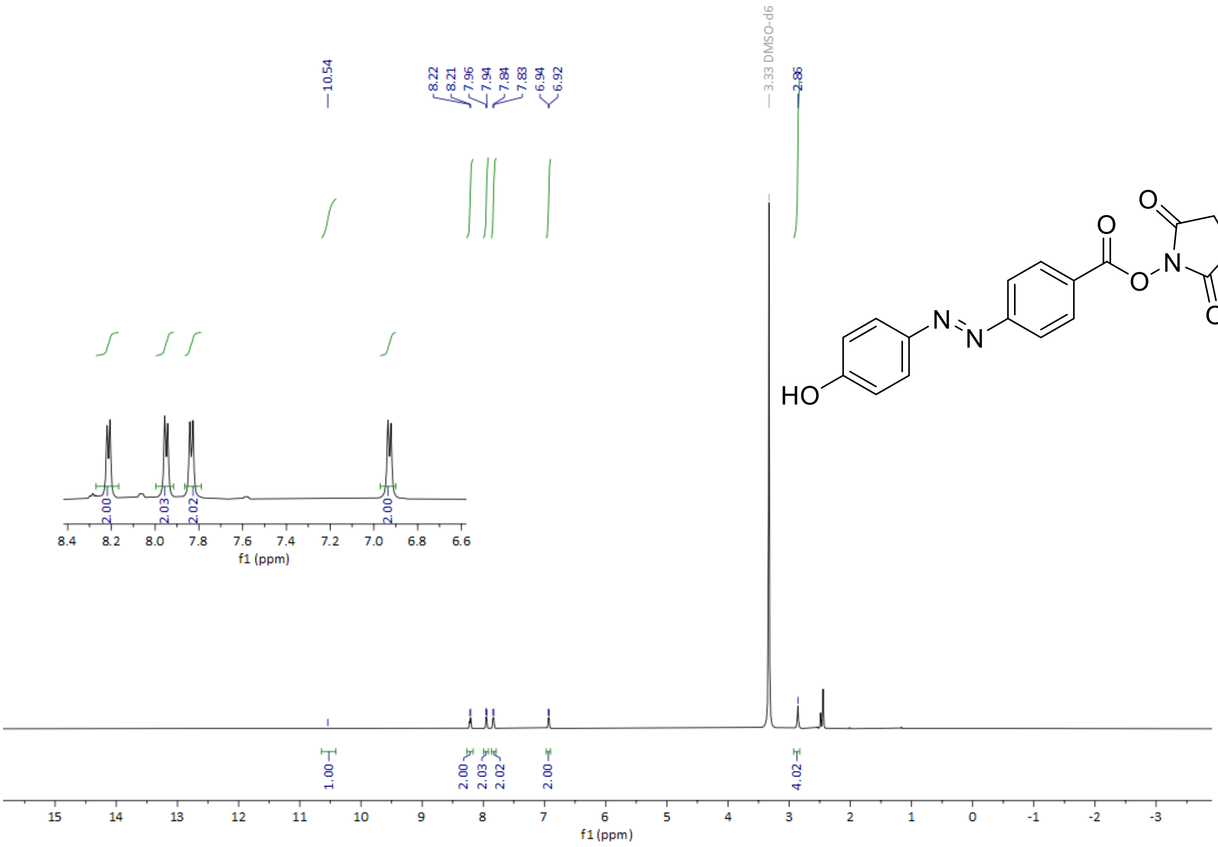

Figure S5: ^{1}H NMR spectrum of **3d** in DMSO-d_6.

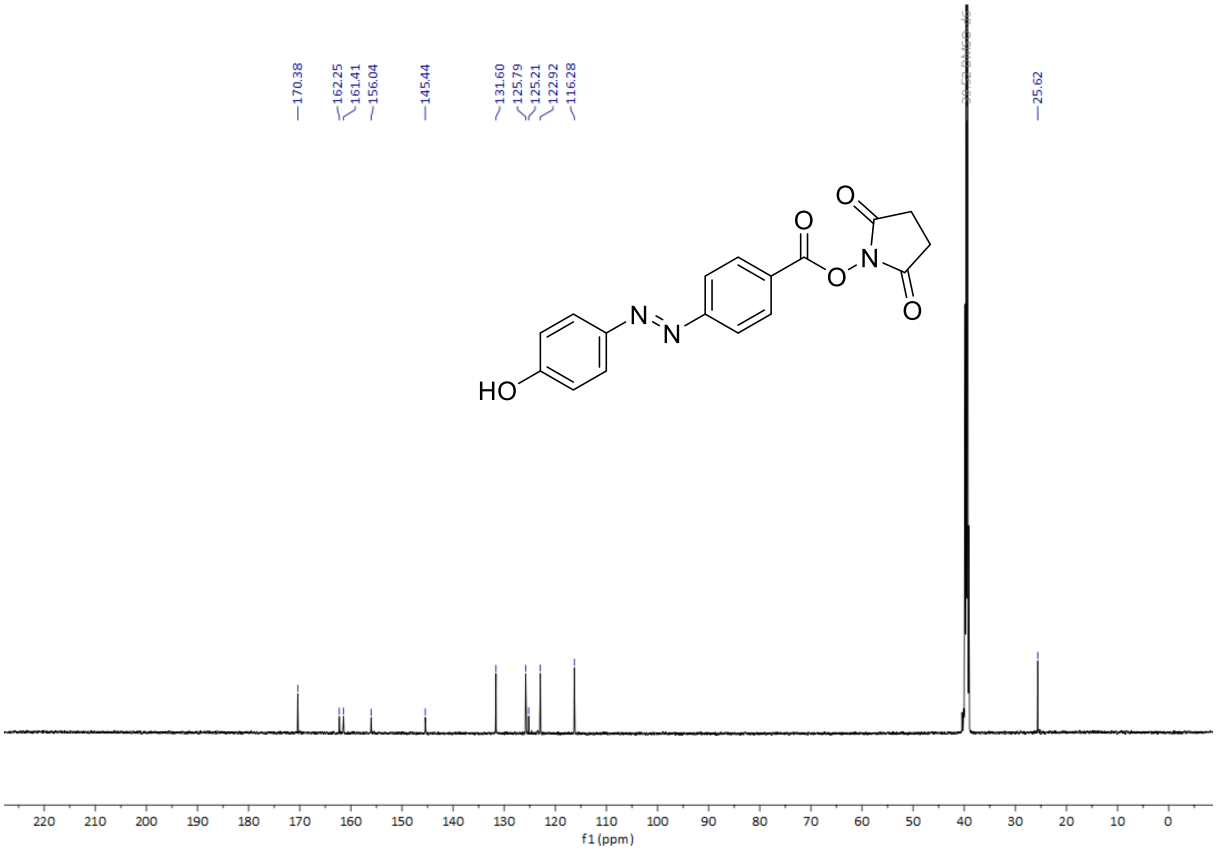

Figure S6: $^{13}C\{^{1}H\}$ NMR spectrum of **3d** in DMSO-d_6.

2,5-Dioxopyrrolidin-1-yl (*E*)-4-((4-aminophenyl)diazenyl)benzoate (**3e**)

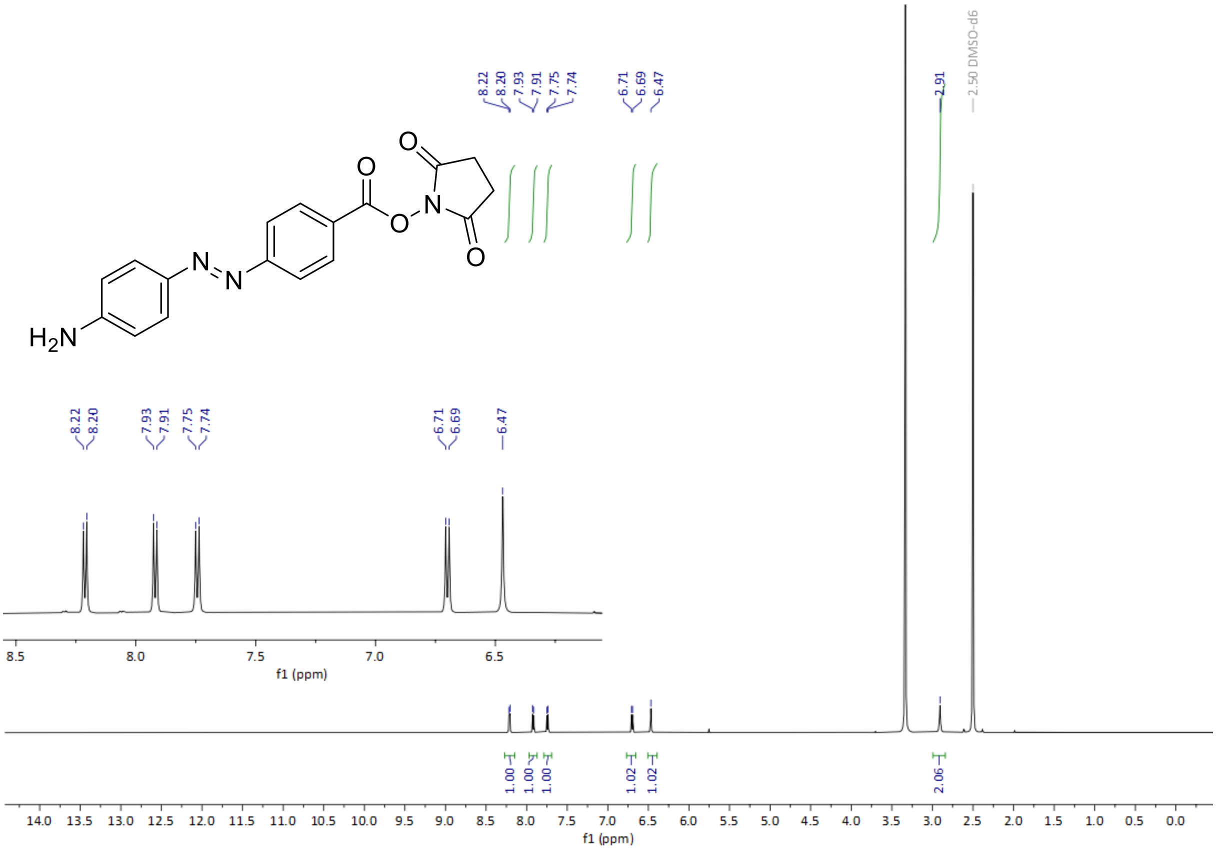

Figure S7:^{1}H NMR spectrum of **3e** in DMSO-d_6.

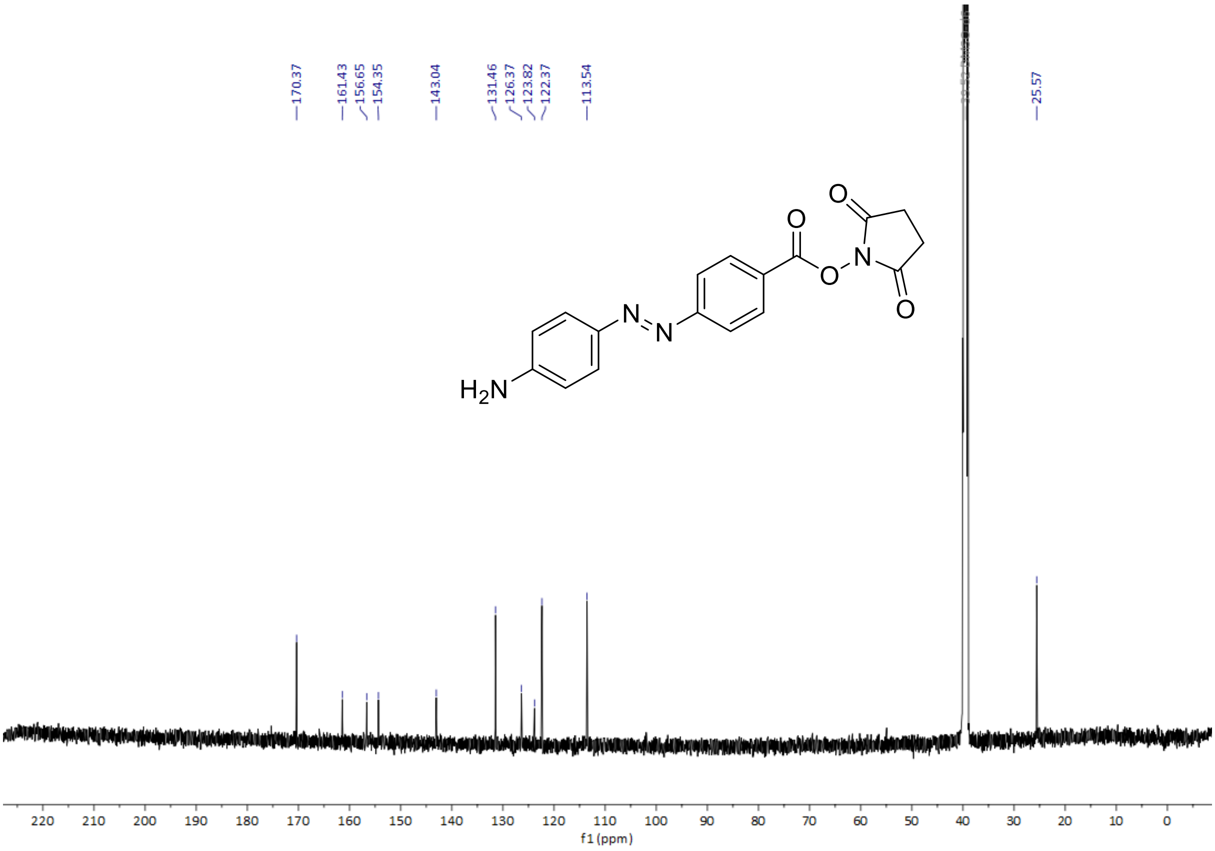

Figure S8: ^{13}C{^{1}H} NMR spectrum of **3e** in DMSO-d_6.

2,5-Dioxopyrrolidin-1-yl (*E*)-4-((4-acetamidophenyl)diazenyl)benzoate (**3f**)

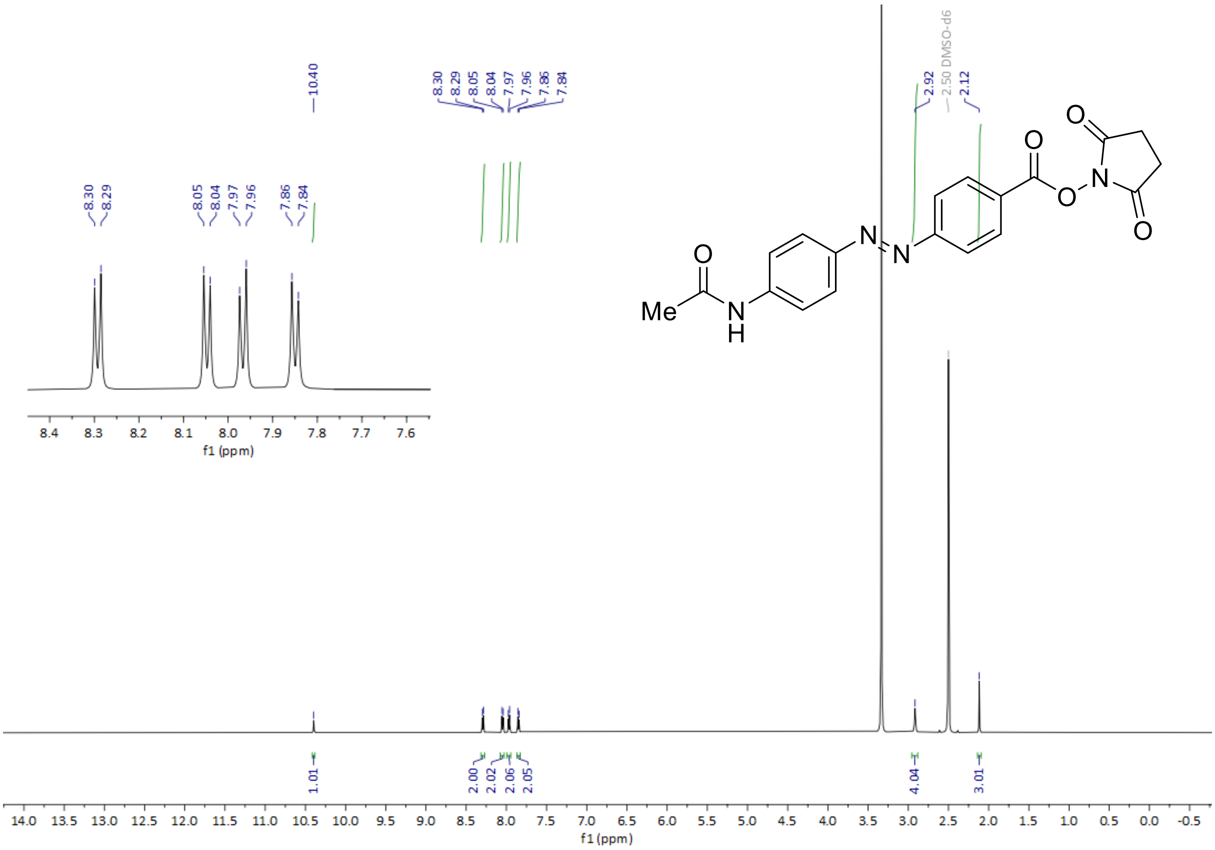

Figure S9:^{1}H NMR spectrum of **3f** in DMSO-d_6.

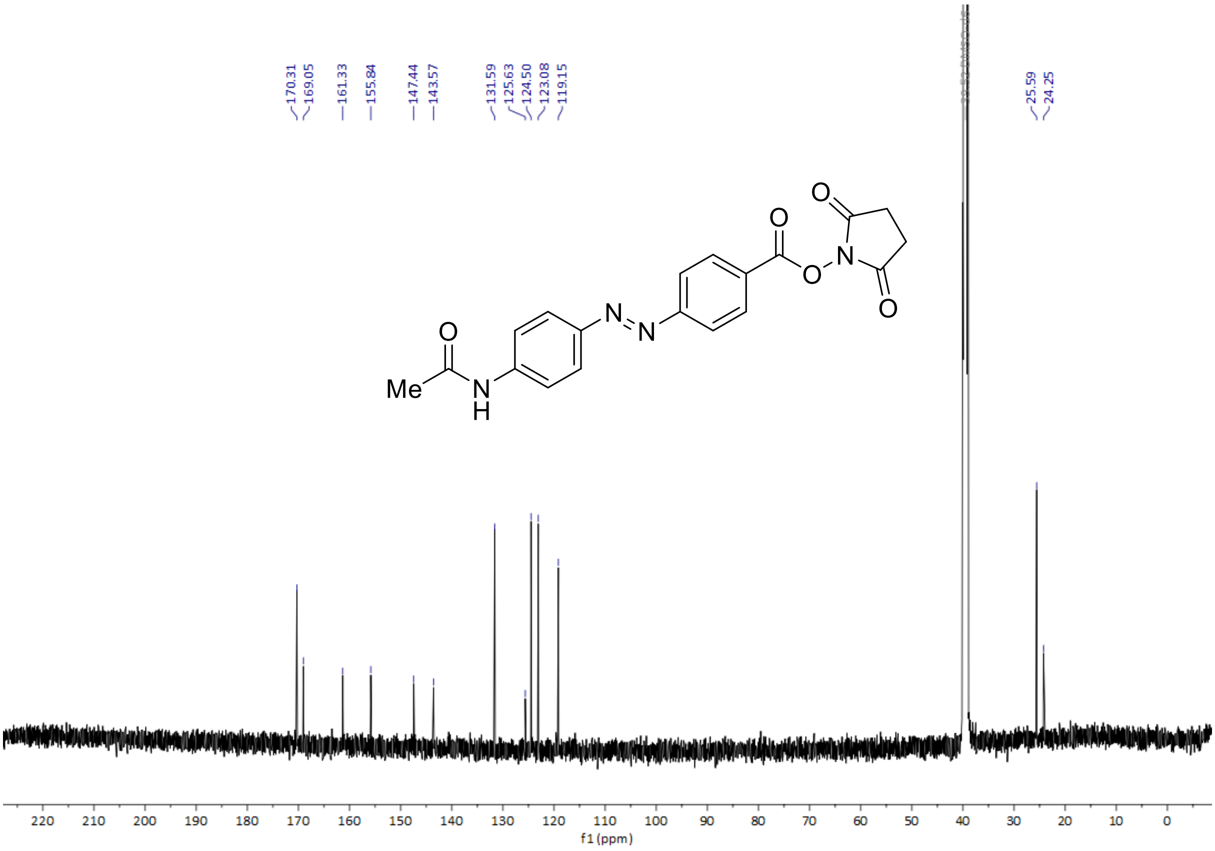

Figure S10: $^{13}C\{^1H\}$ NMR spectrum of **3f** in DMSO-d_6.

2,5-Dioxopyrrolidin-1-yl (*E*)-4-((4-nitrophenyl)diazenyl)benzoate (**3g**)

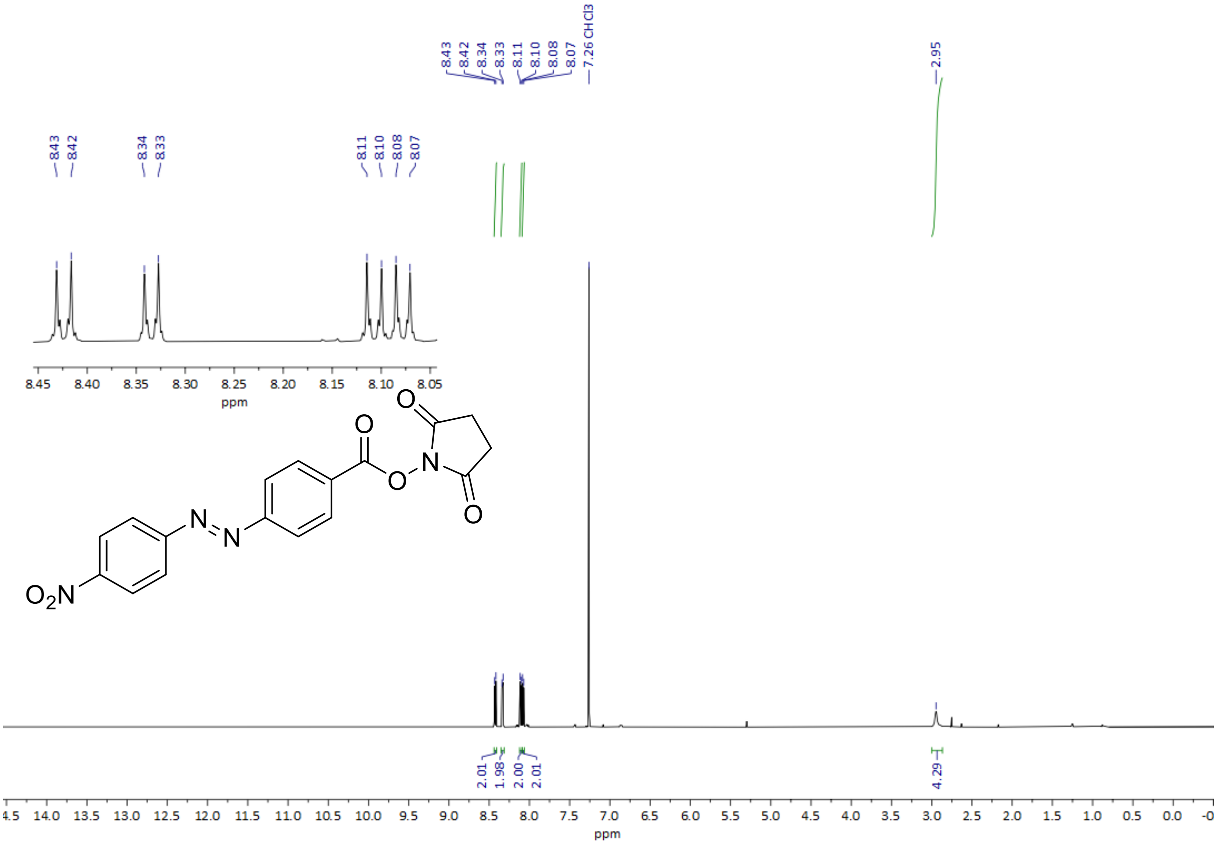

Figure S11: ^{1}H NMR spectrum of **3g** in $CDCl_3$.

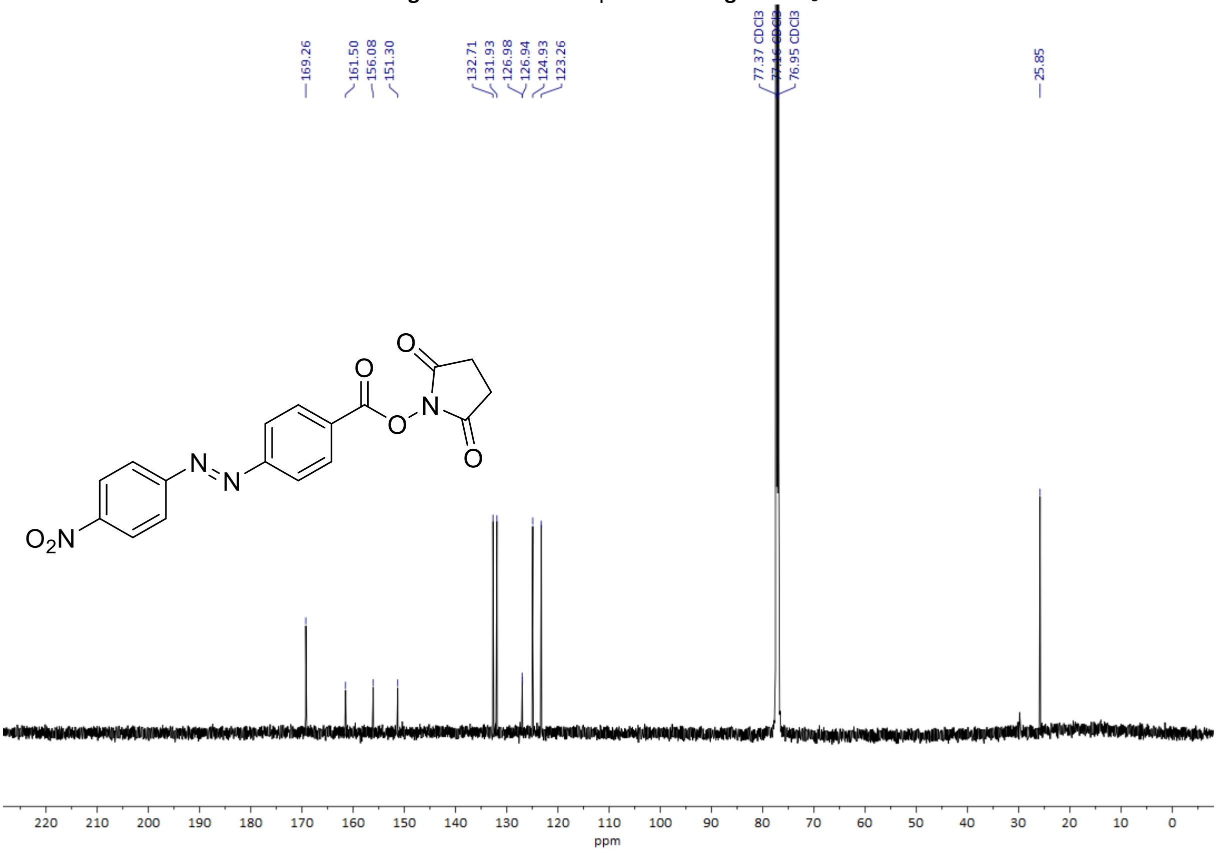

Figure S12: $^{13}C\{^{1}H\}$ NMR spectrum of **3g** in $CDCl_3$.

2,5-Dioxopyrrolidin-1-yl (*E*)-4-((4-(allyloxy)phenyl)diazenyl)benzoate (**3h**)

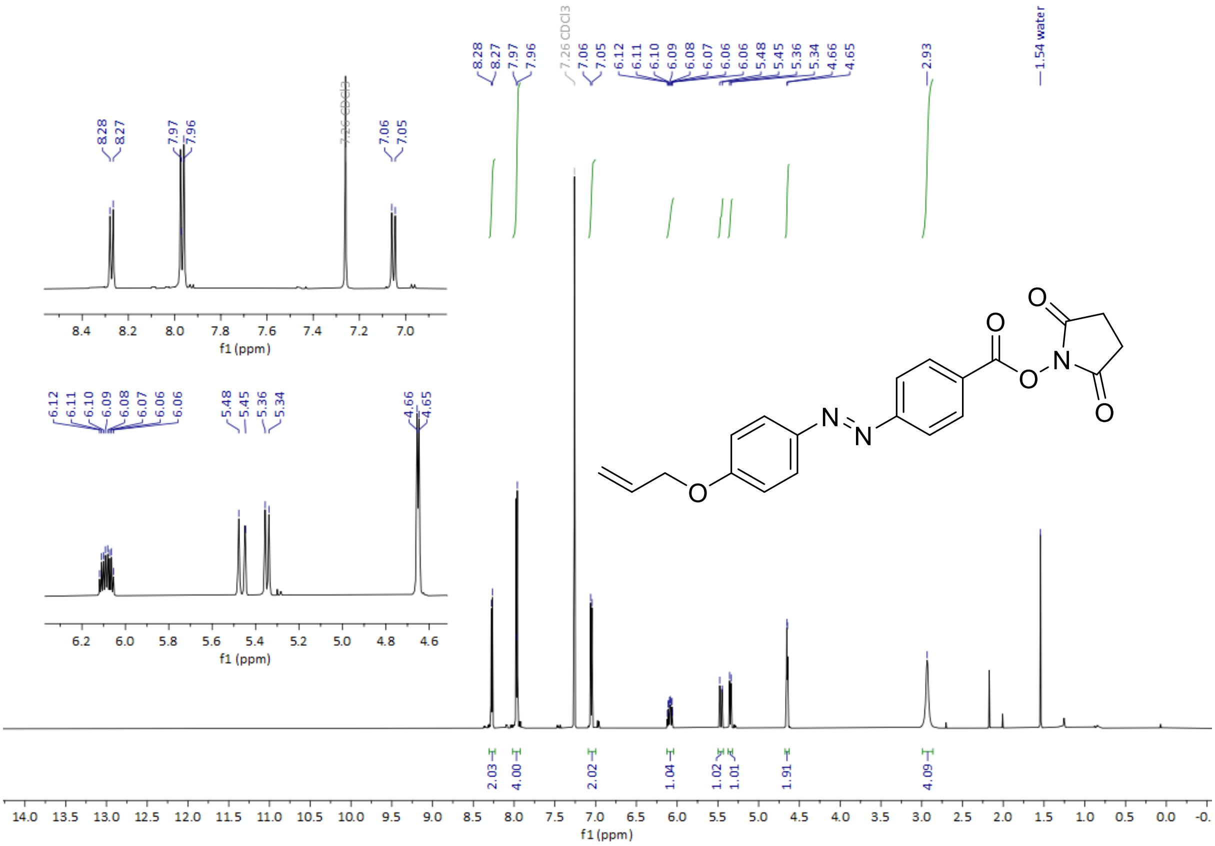

Figure S13:^{1}H NMR spectrum of **3h** in $CDCl_3$.

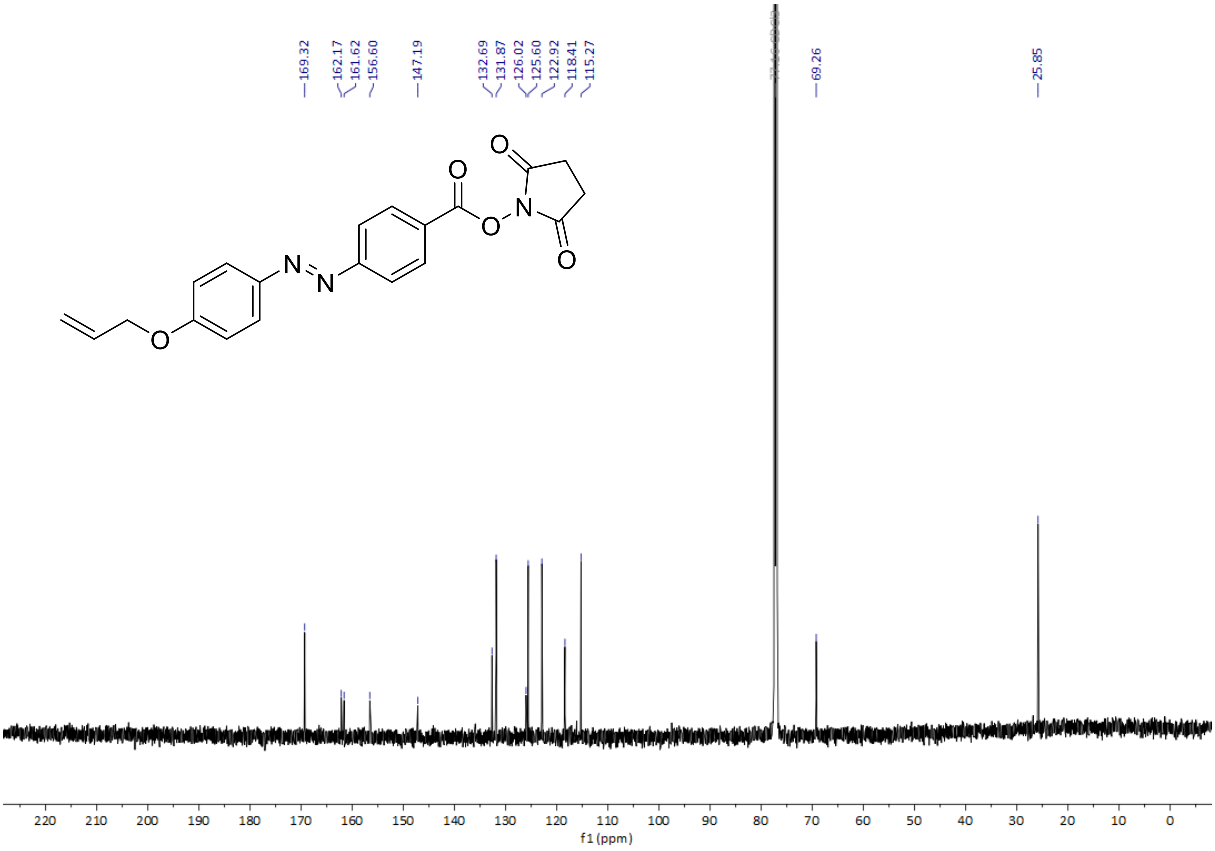

Figure S14: $^{13}C\{^1H\}$ NMR spectrum of **3h** in $CDCl_3$.

2,5-Dioxopyrrolidin-1-yl (*E*)-4-((4-((9-hydroxynonyl)oxy)phenyl)diazenyl)benzoate (**3i**)

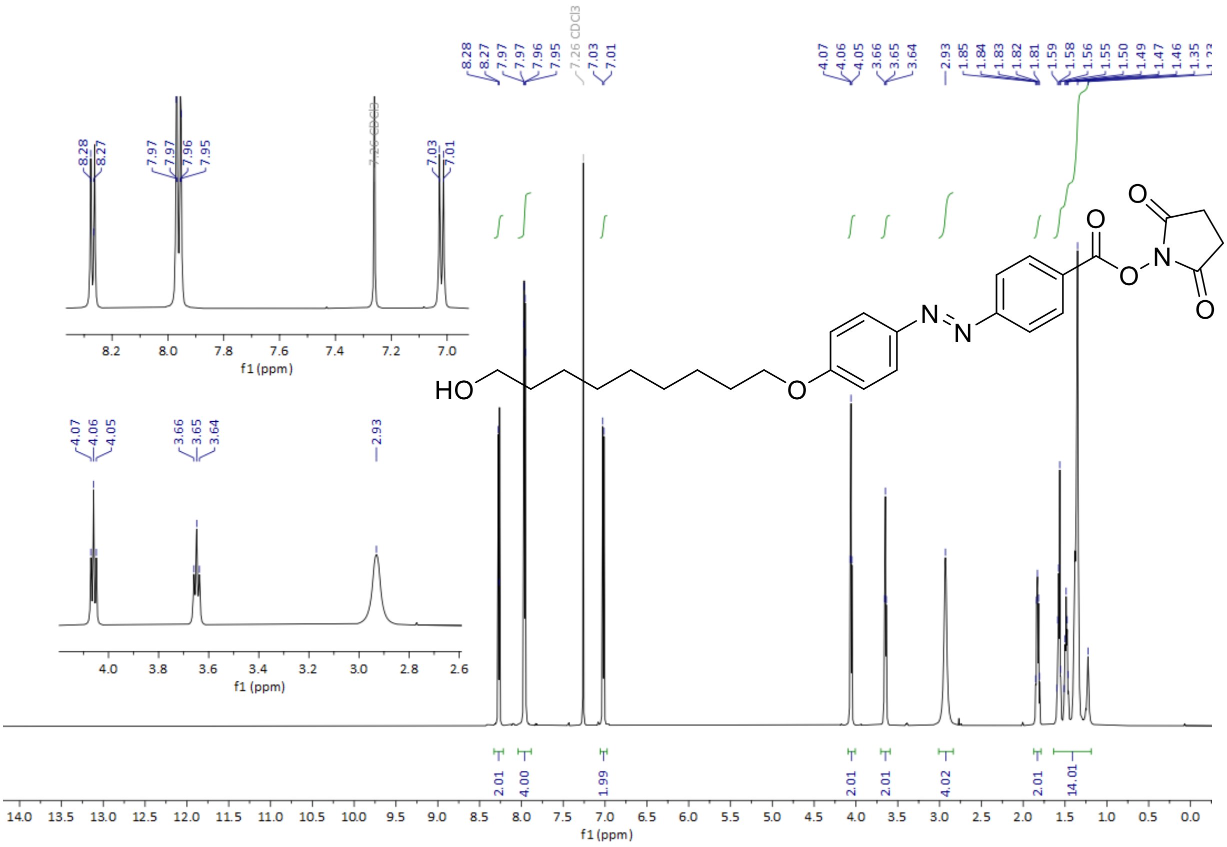

Figure S15:^{1}H NMR spectrum of **3i** in $CDCl_3$.

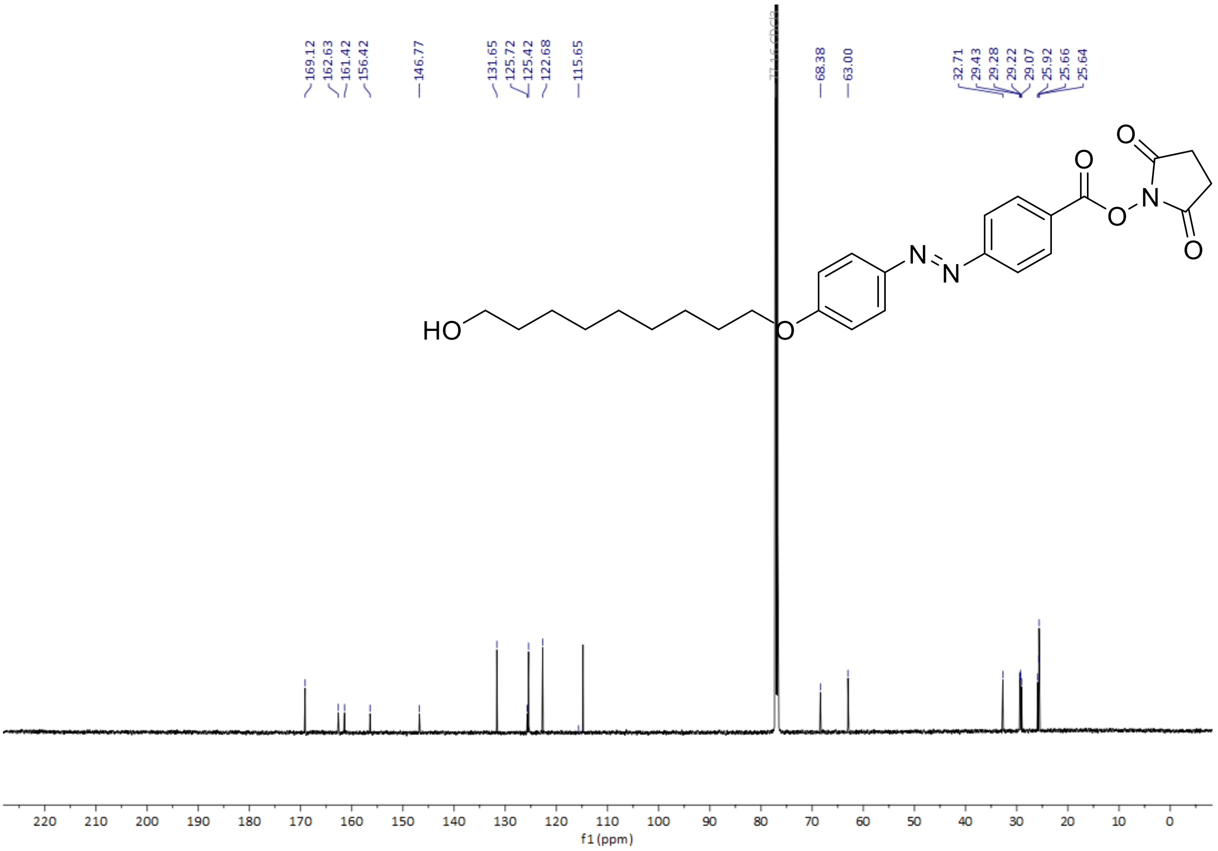

Figure S16: $^{13}C\{^{1}H\}$ NMR spectrum of **3i** in $CDCl_3$.

2,5-Dioxopyrrolidin-1-yl (E)-4-((2,6-dimethyl-4-(nonyloxy)phenyl)diazenyl)-3,5-dimethylbenzoate (**3j**)

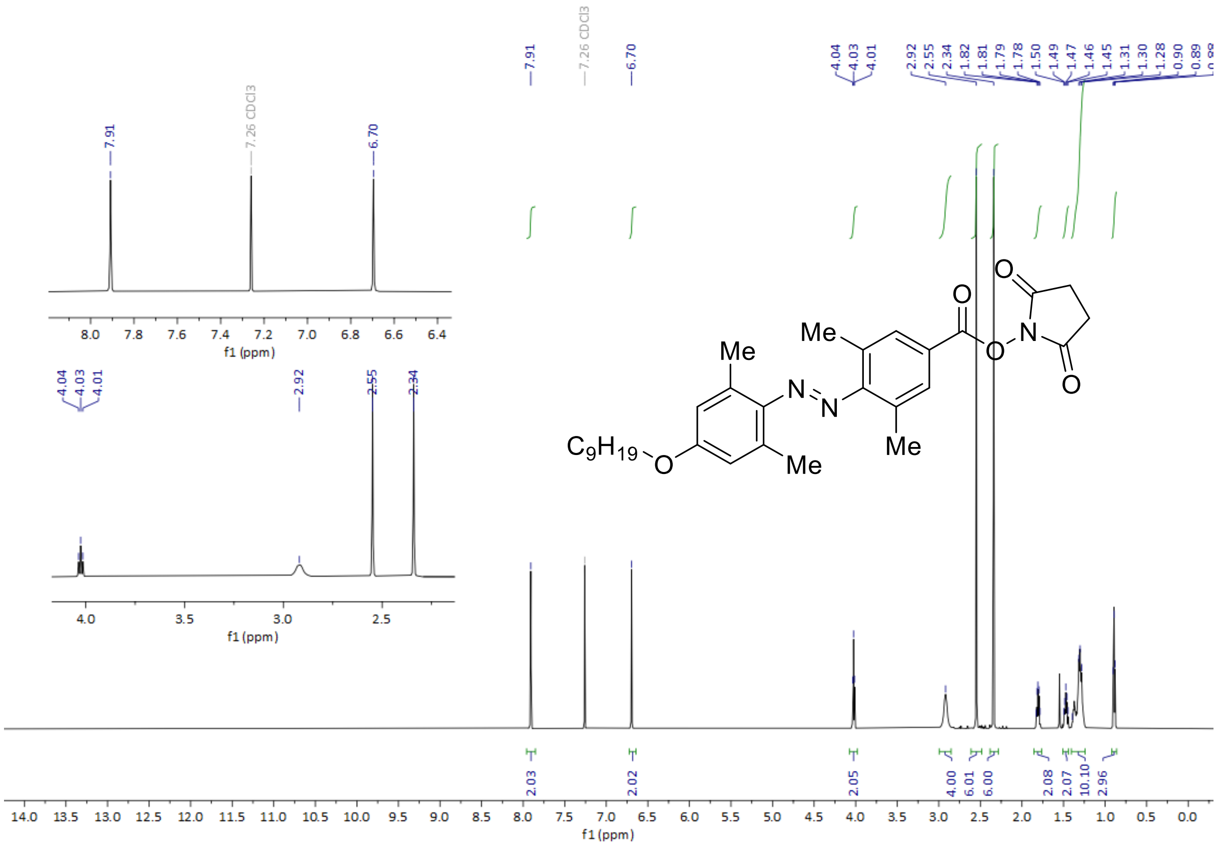

Figure S17:^{1}H NMR spectrum of **3j** in $CDCl_3$.

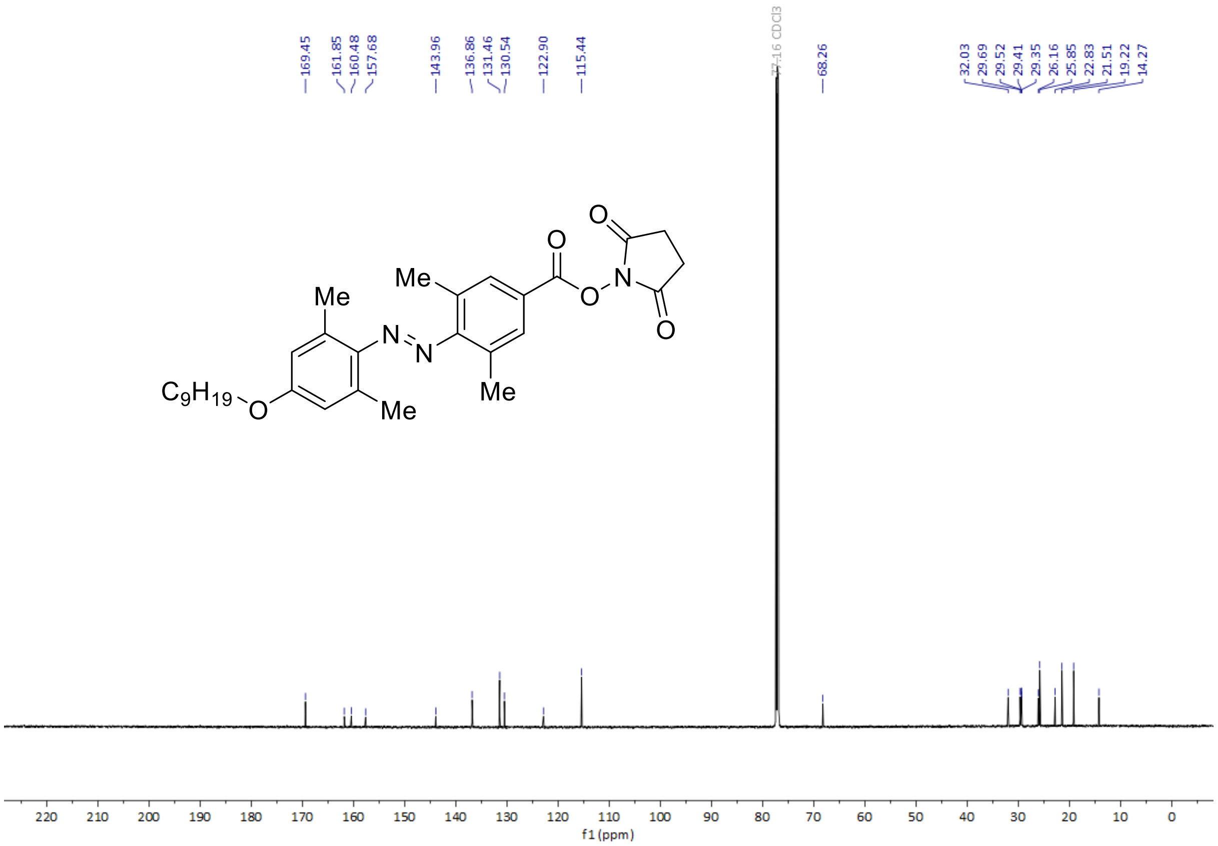

Figure S18: $^{13}C\{^{1}H\}$ NMR spectrum of **3j** in $CDCl_3$.

2,5-Dioxopyrrolidin-1-yl (*E*)-4-((2,7-bis(4-(hexyloxy)phenoxy)naphthalen-1-yl)diazenyl)-3,5-dimethylbenzoate (**3k**)

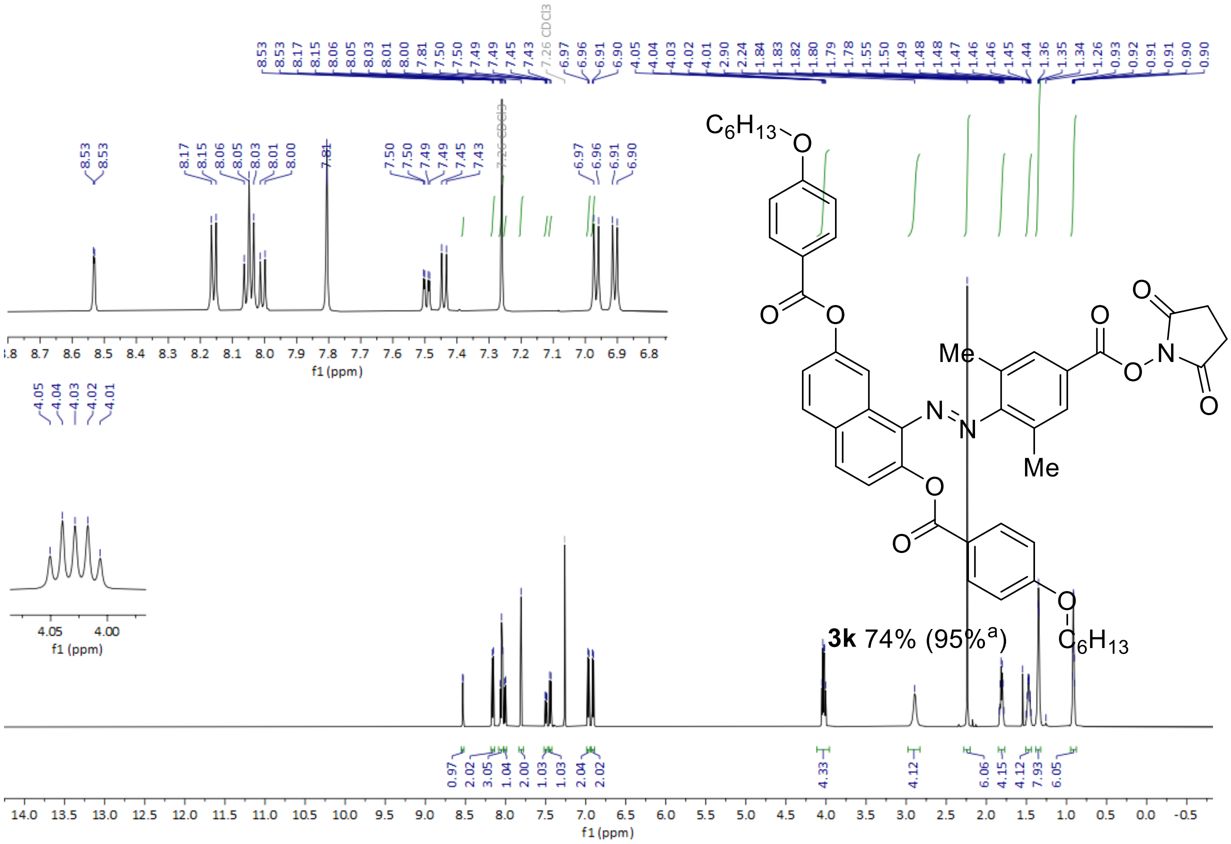

Figure S19: ^{1}H NMR spectrum of **3k** in $CDCl_3$.

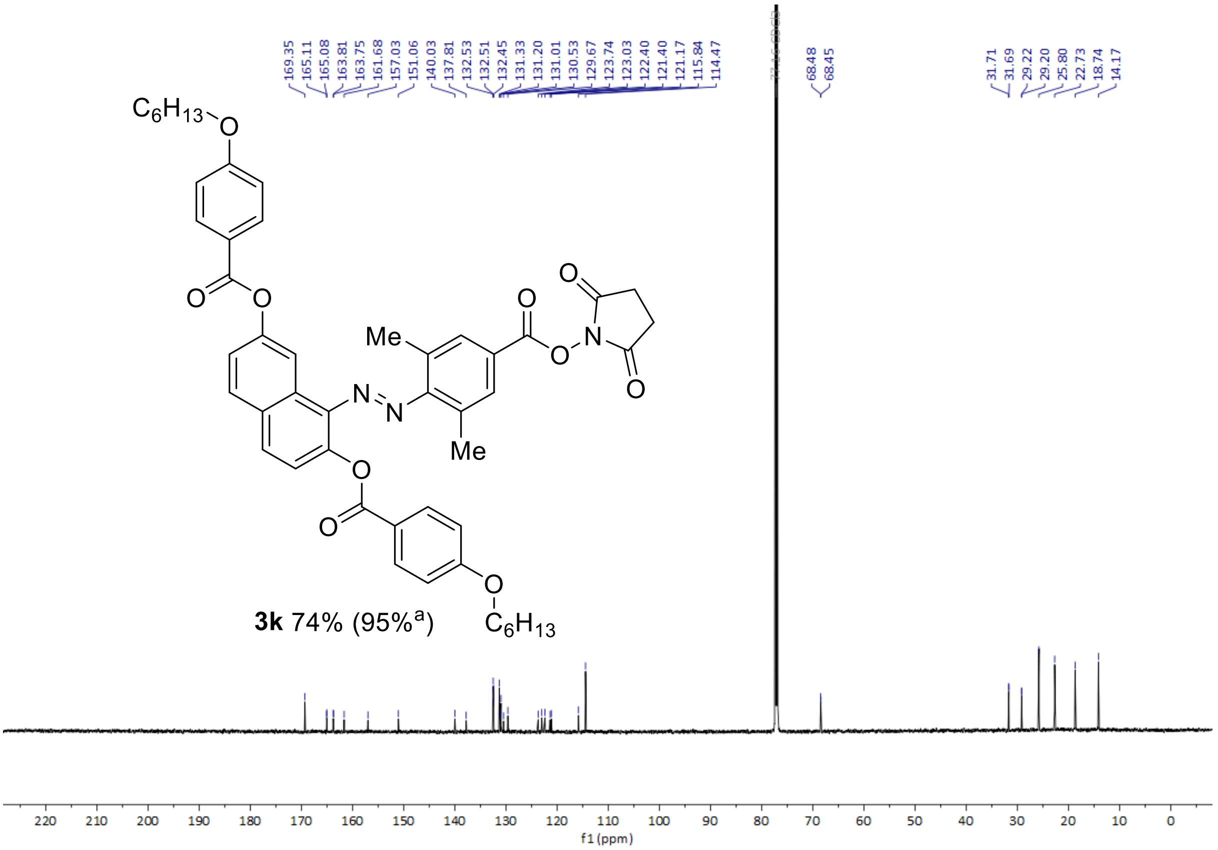

Figure S20: $^{13}C\{^{1}H\}$ NMR spectrum of **3k** in $CDCl_3$.

2,5-Dioxopyrrolidin-1-yl (*E*)-4-((4-bromophenyl)diazenyl)benzoate (**3l**)

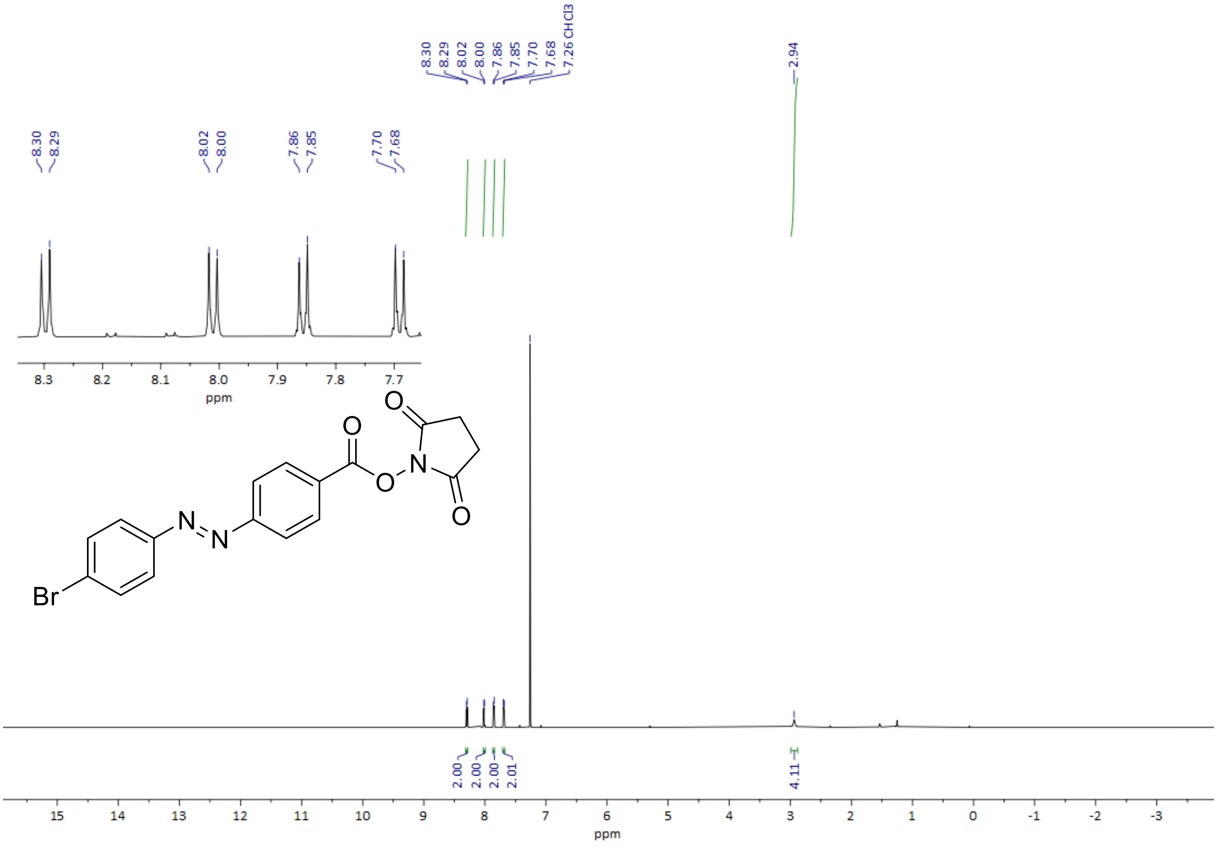

Figure S21: ^{1}H NMR spectrum of **3l** in $CDCl_3$.

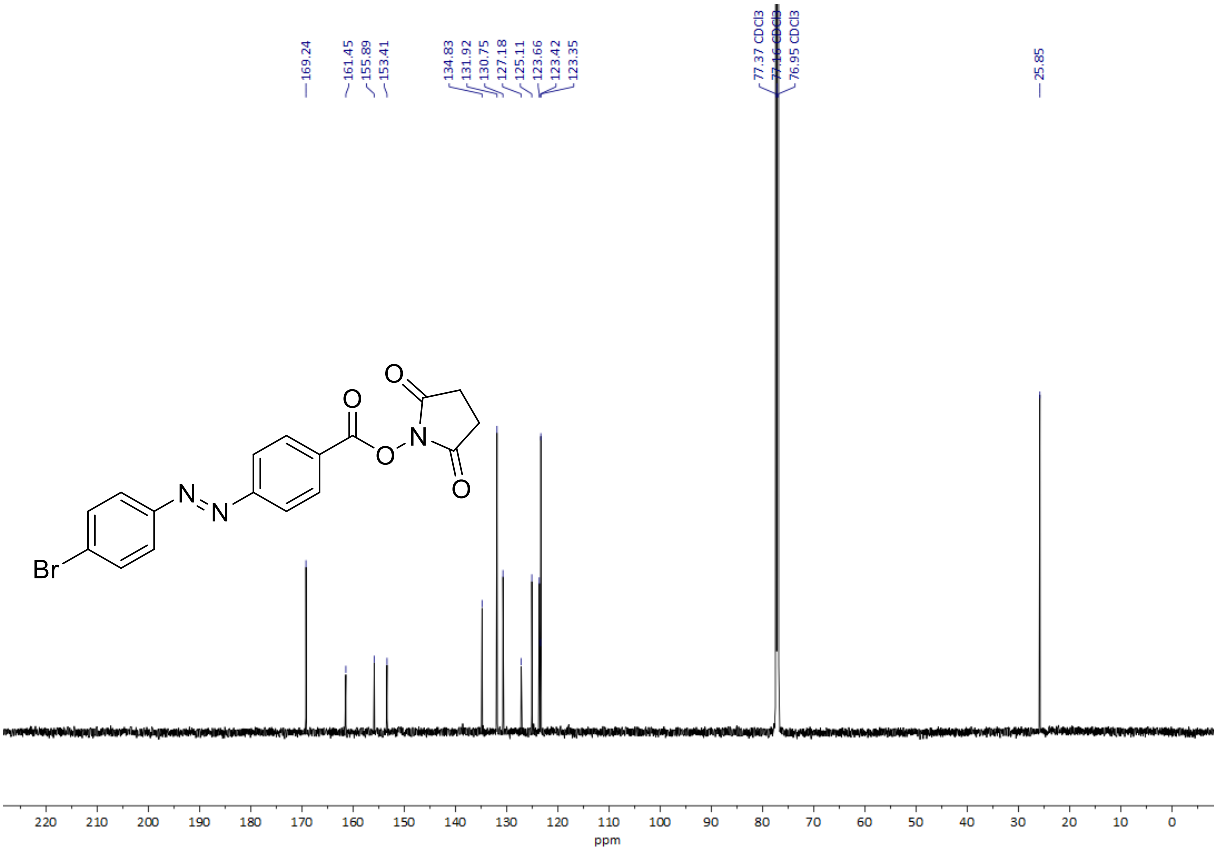

Figure S22: $^{13}C\{^{1}H\}$ NMR spectrum of **3l** in $CDCl_3$.

2,5-Dioxopyrrolidin-1-yl (*E*)-4-((3-bromophenyl)diazenyl)benzoate (**3m**)

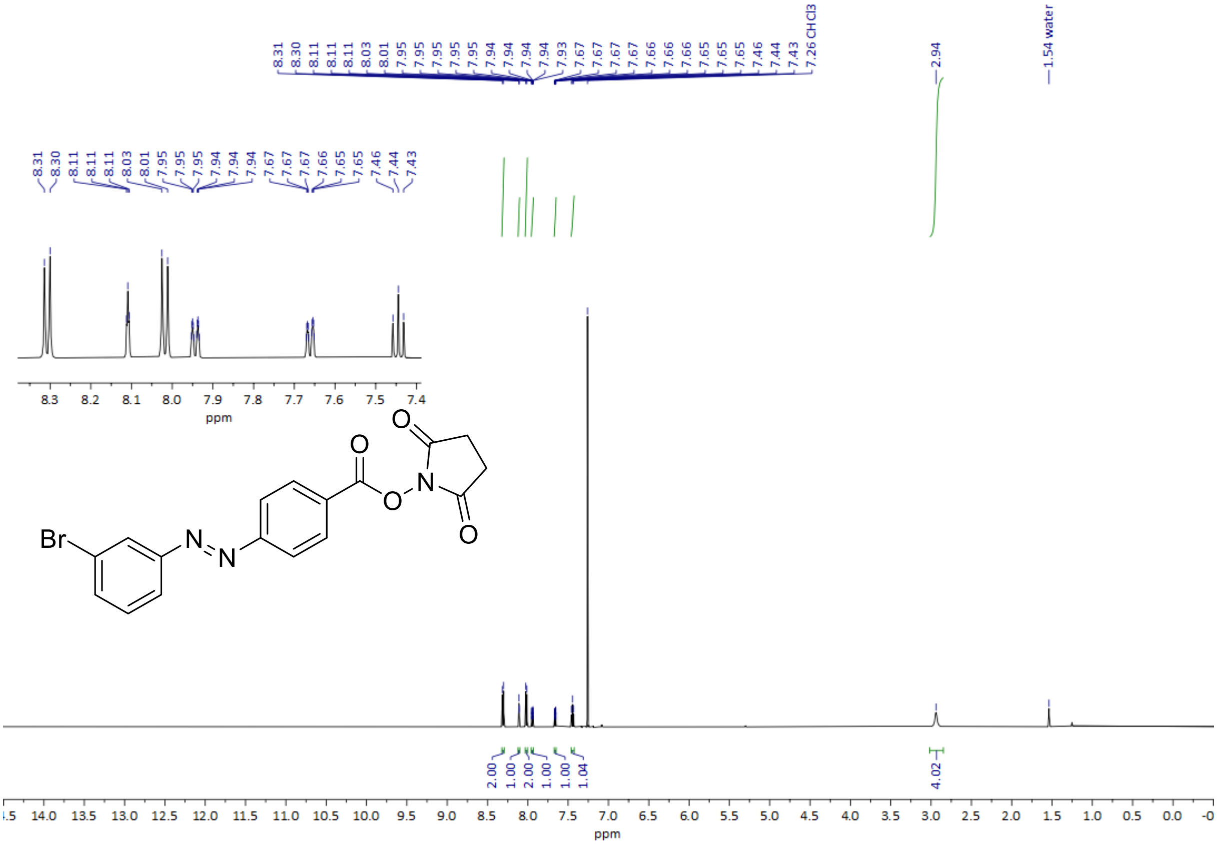

Figure S23: ^{1}H NMR spectrum of **3m** in $CDCl_3$.

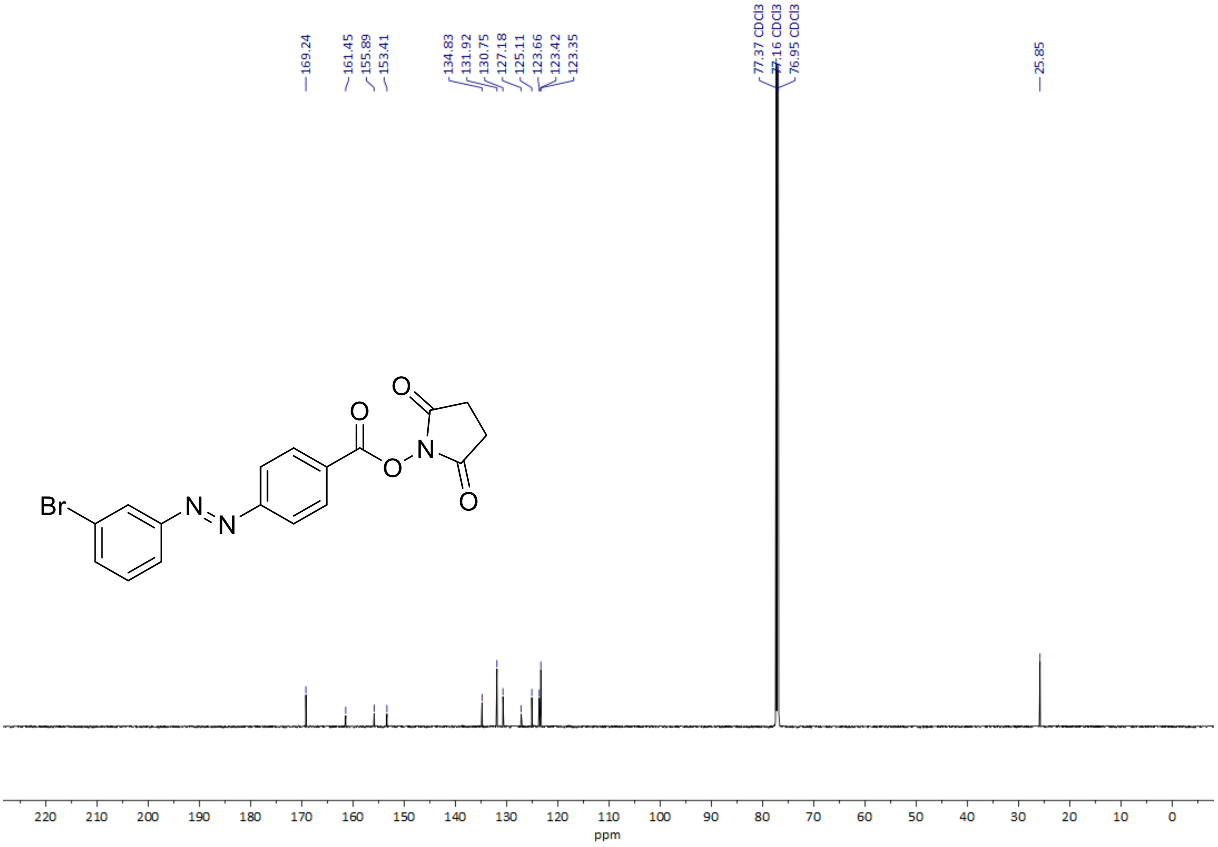

Figure S24: $^{13}C\{^{1}H\}$ NMR spectrum of **3m** in $CDCl_3$.

2,5-Dioxopyrrolidin-1-yl (*E*)-4-((2-bromophenyl)diazenyl)benzoate (**3n**)

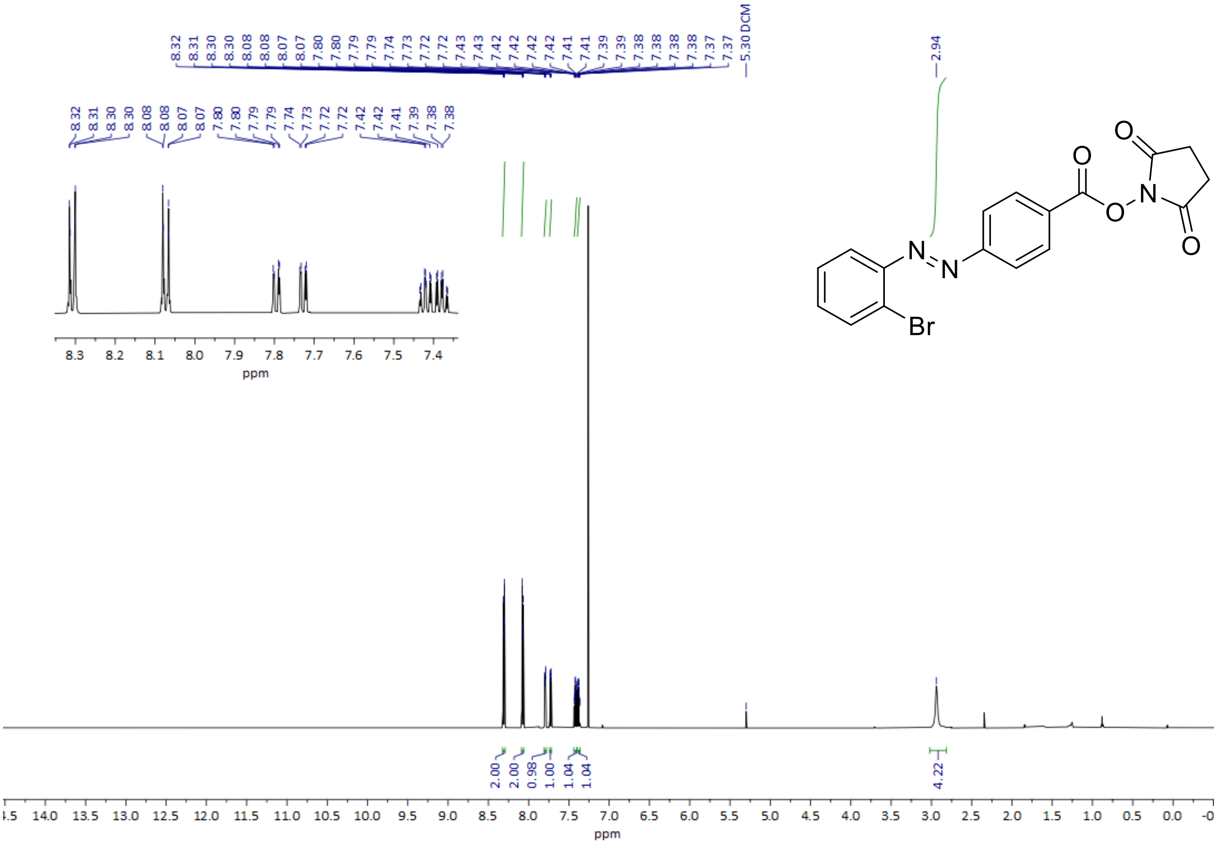

Figure S25: ^{1}H NMR spectrum of **3n** in $CDCl_3$.

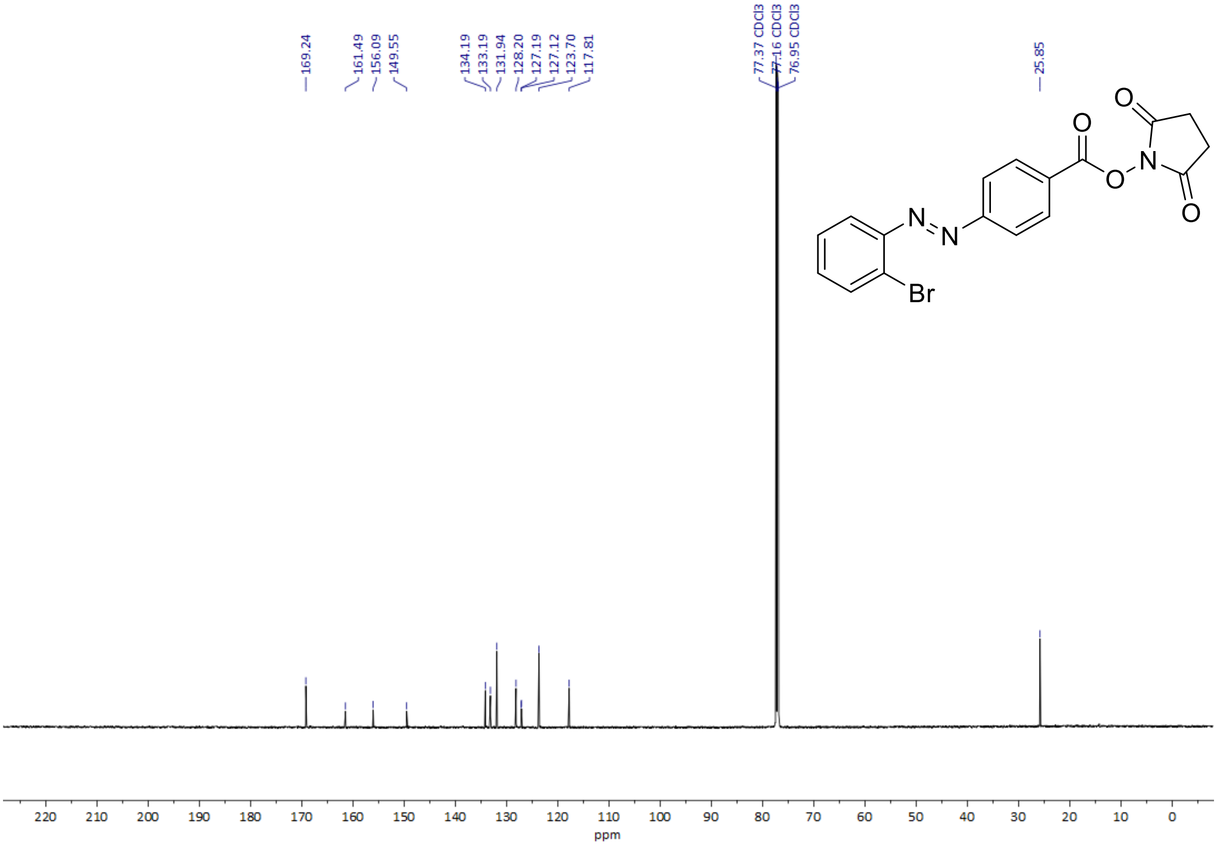

Figure S26: ^{13}C{^{1}H} NMR spectrum of **3n** in $CDCl_3$.

Bis(2,5-dioxopyrrolidin-1-yl) 4,4'-(diazene-1,2-diyl)(*E*)-dibenzoate (**5a**)

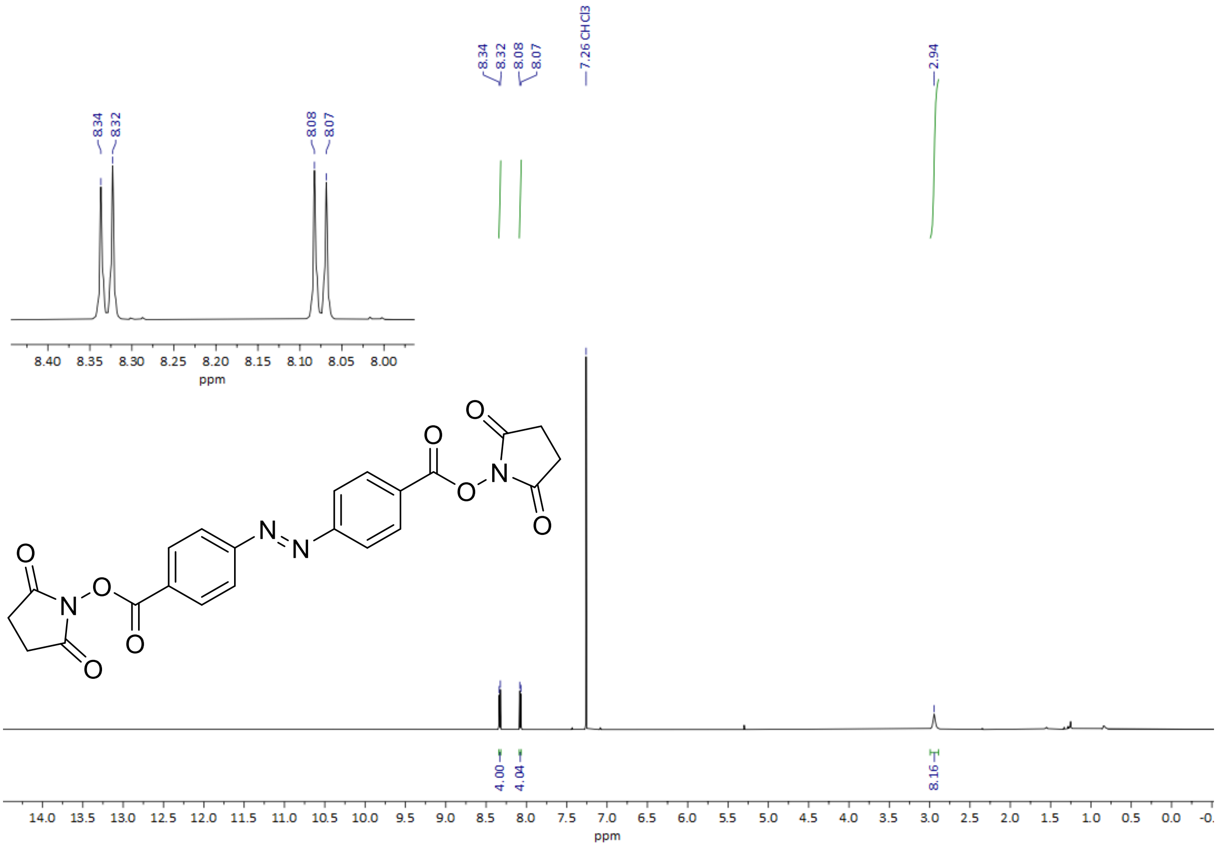

Figure S27:^{1}H NMR spectrum of **5a** in $CDCl_3$.

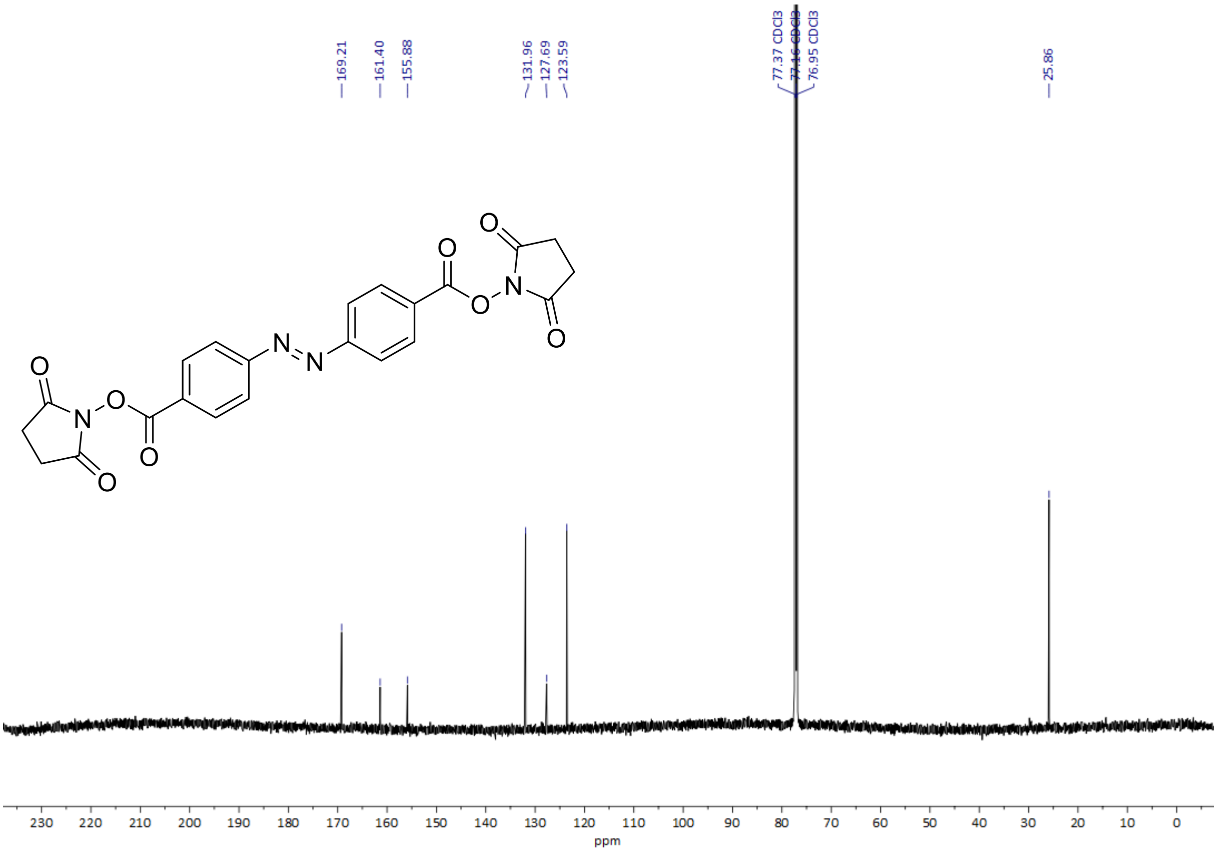

Figure S28: $^{13}C\{^{1}H\}$ NMR spectrum of **5a** in $CDCl_3$.

Bis(2,5-dioxopyrrolidin-1-yl) 4,4'-(diazene-1,2-diyl)(*E*)-bis(3,5-dimethylbenzoate) (**5b**)

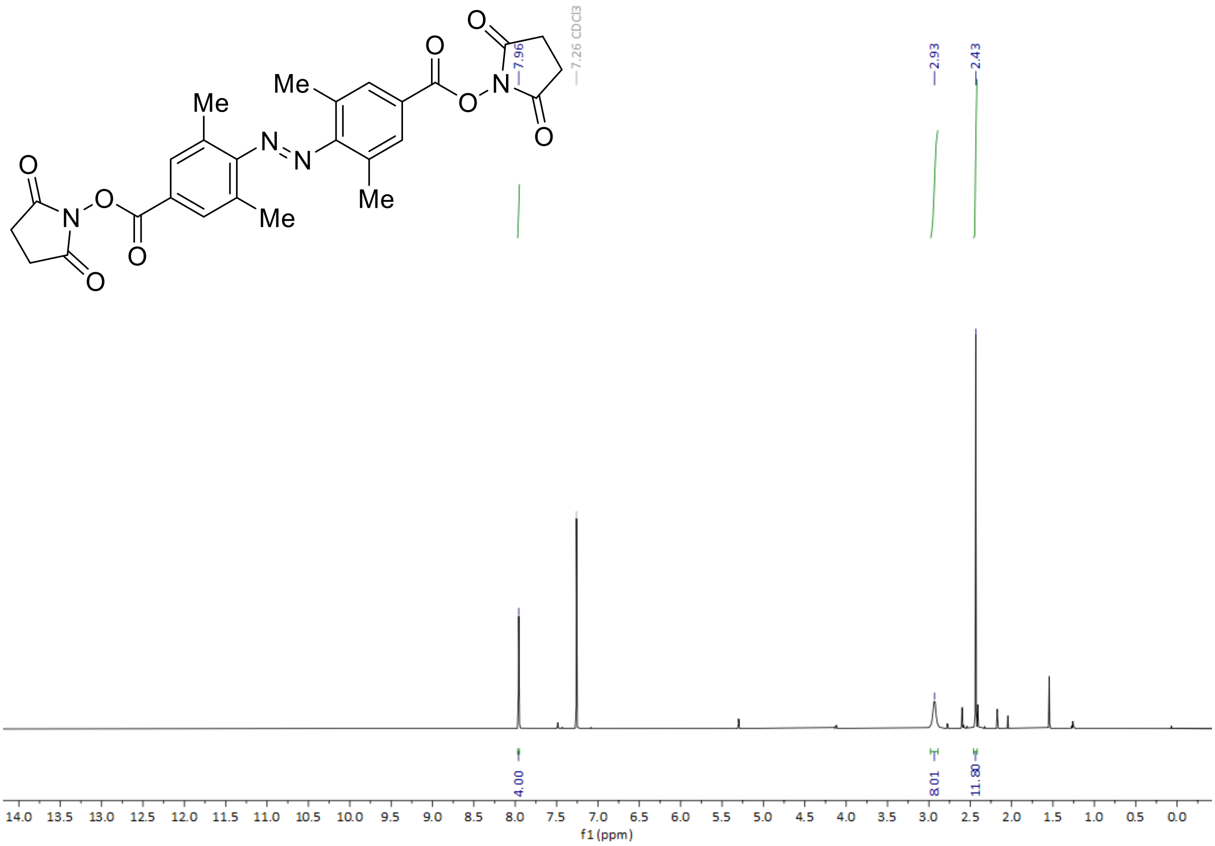

Figure S29:^{1}H NMR spectrum of **5b** in $CDCl_3$.

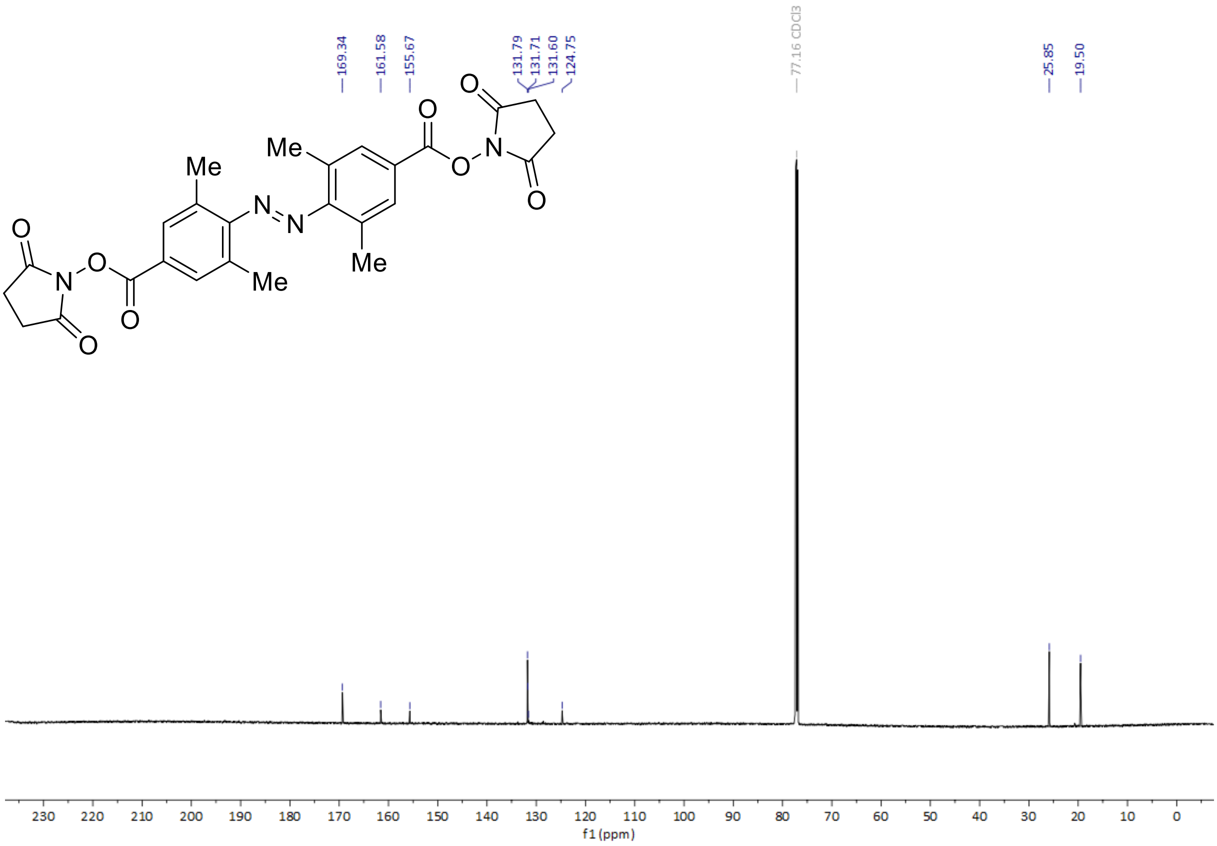

Figure S30:^{1}H NMR spectrum of **5b** in $CDCl_3$.

Bis(2,5-dioxopyrrolidin-1-yl) 4,4'-(diazene-1,2-diyl)(*E*)-bis(3,5-dimethoxybenzoate) (**5c**)

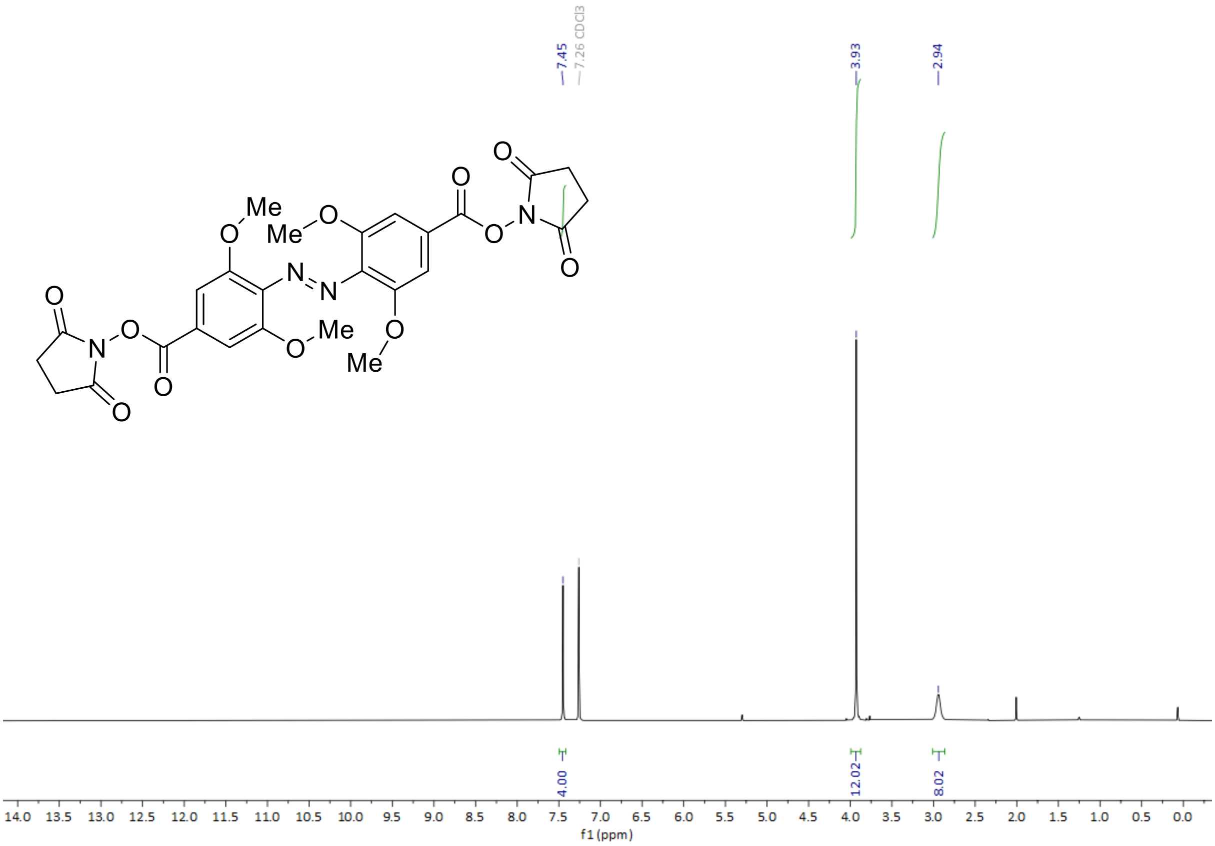

Figure S31: ^{1}H NMR spectrum of **5c** in $CDCl_3$.

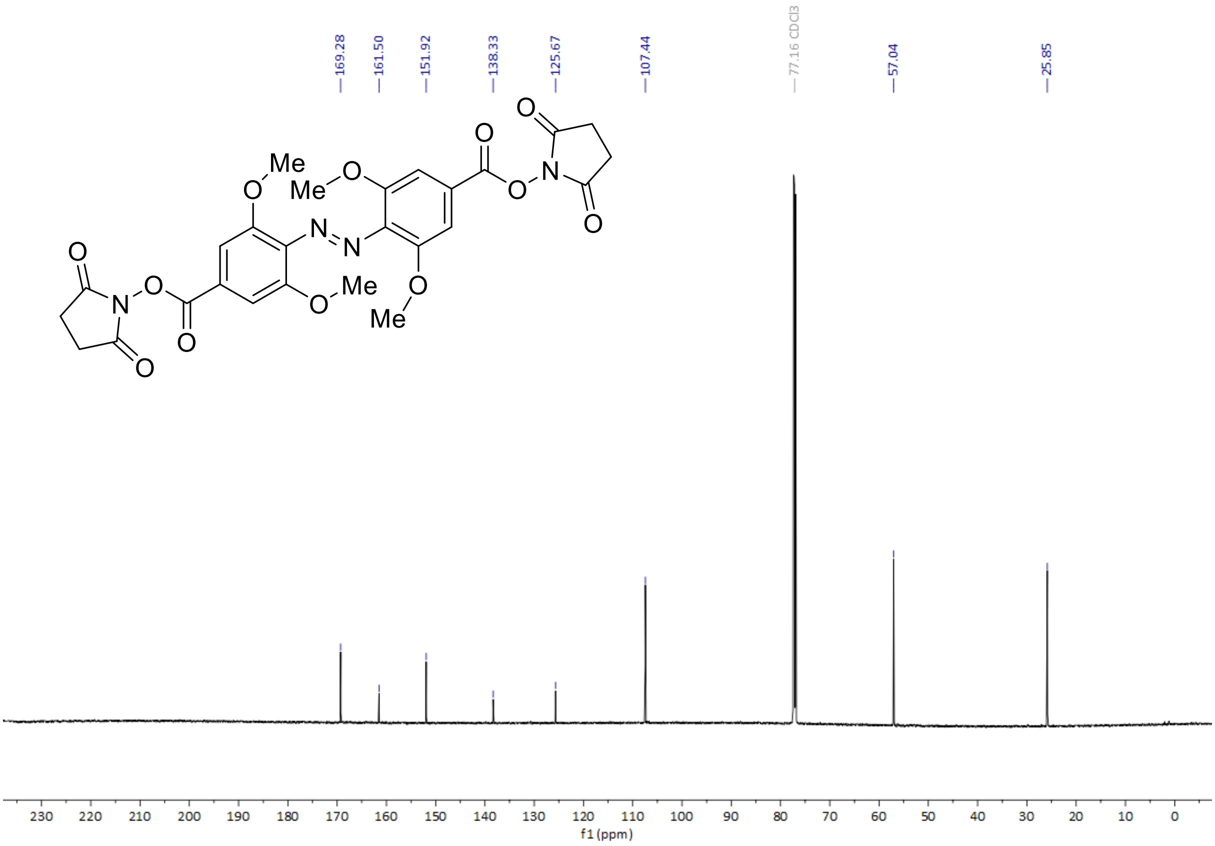

Figure S32: $^{13}C\{^{1}H\}$ NMR spectrum of **5c** in $CDCl_3$.

Bis(2,5-dioxopyrrolidin-1-yl) 4,4'-(diazene-1,2-diyl)(*E*)-bis(3,5-difluorobenzoate) (**5d**)

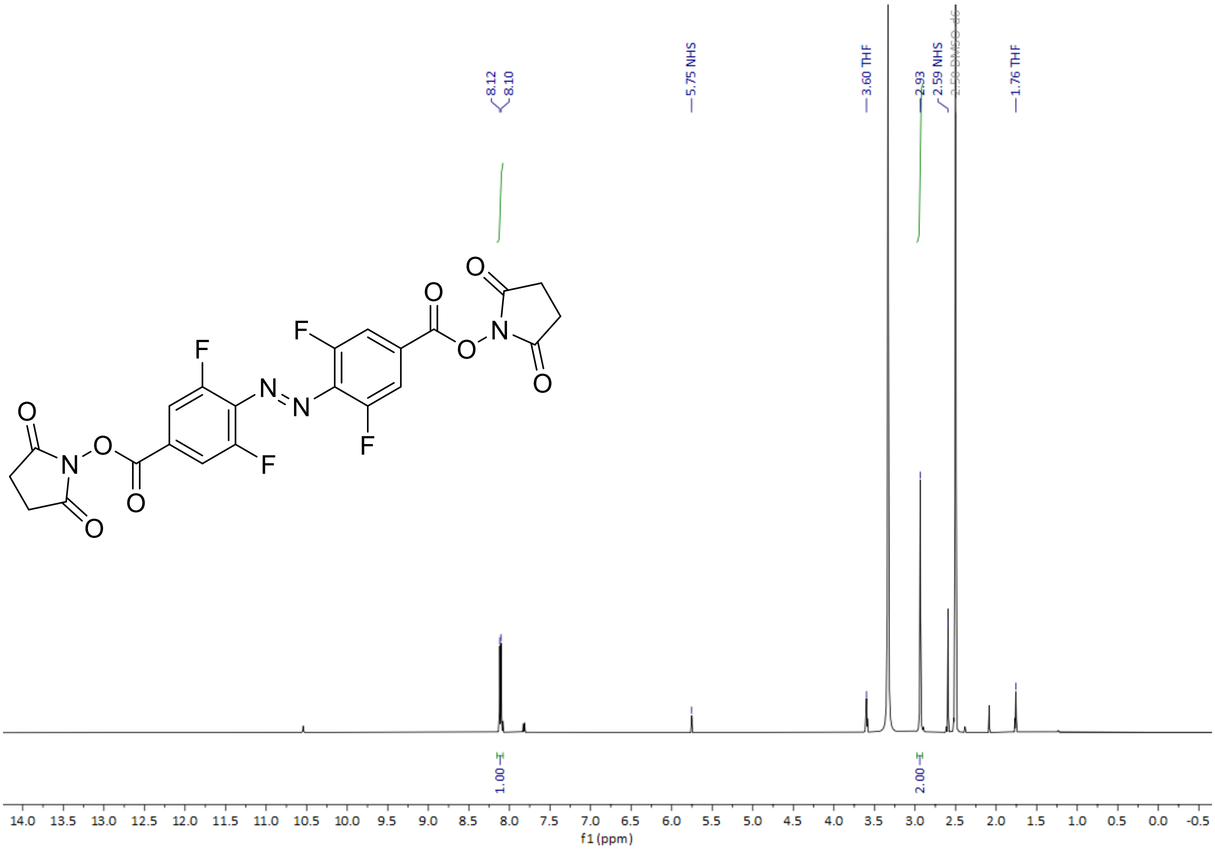

Figure S33:^{1}H NMR spectrum of **5d** in DMSO-d_6.

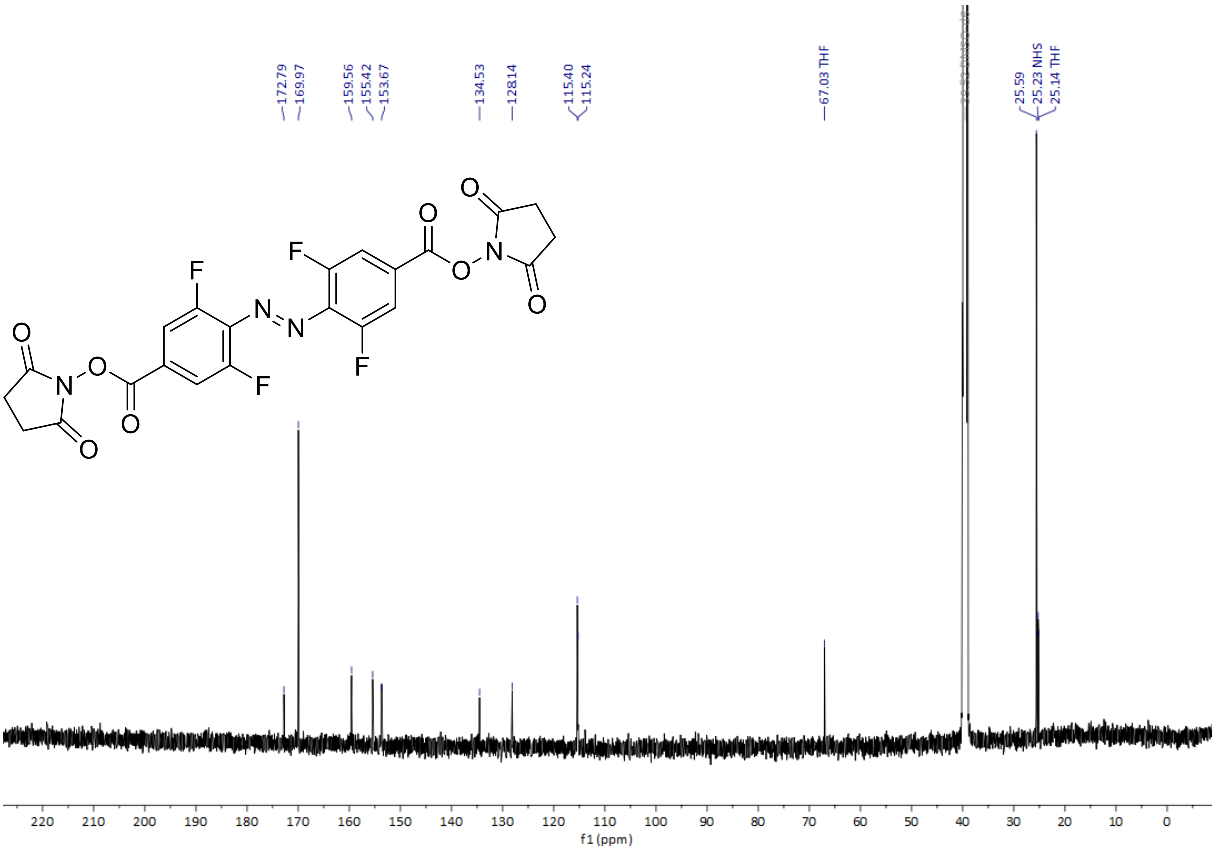

Figure S34: ^{13}C{^{1}H} NMR spectrum of **5d** in DMSO-d_6.

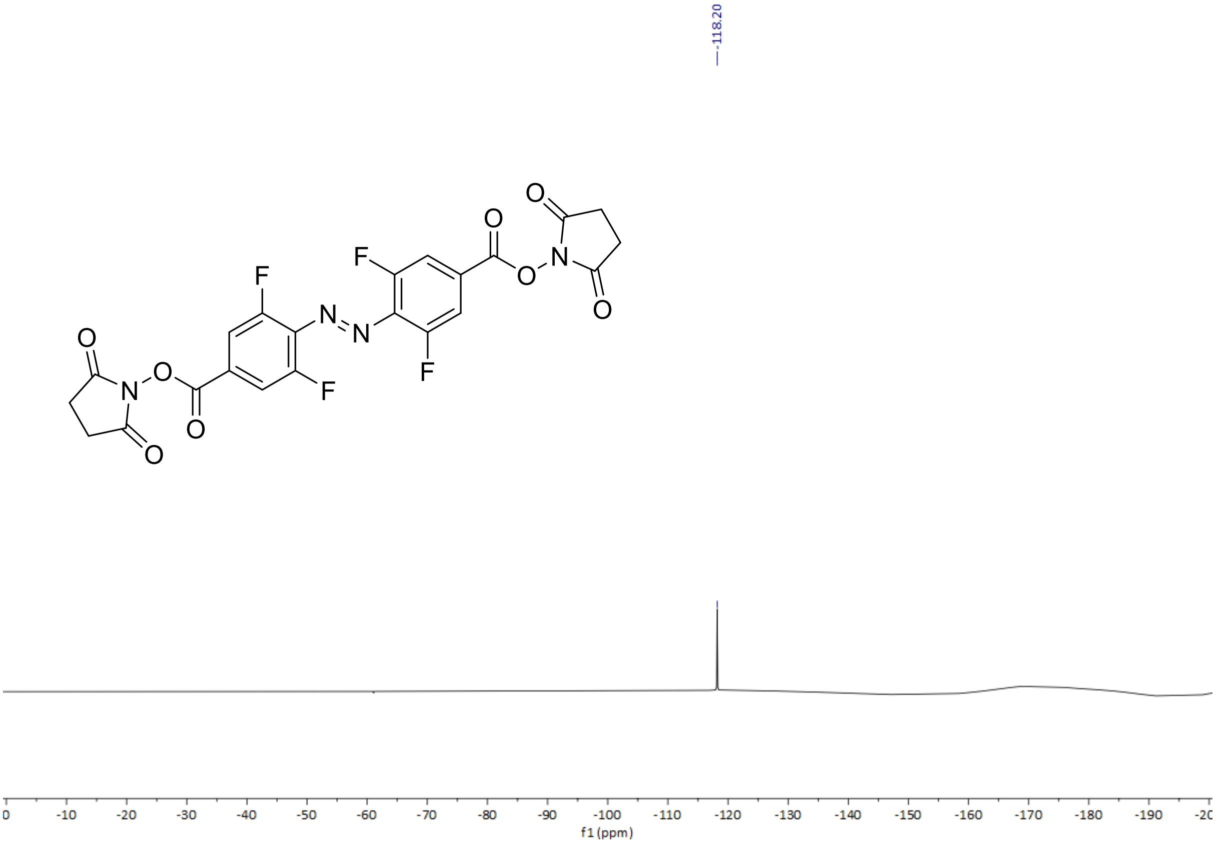

Figure S35: ^{19}F NMR spectrum of **5d** inDMSO-d_6.

2,5-Dioxopyrrolidin-1-yl (Z)-11,12-dihydrodibenzo[c,g][1,2]diazocine-2-carboxylate (**8a**)

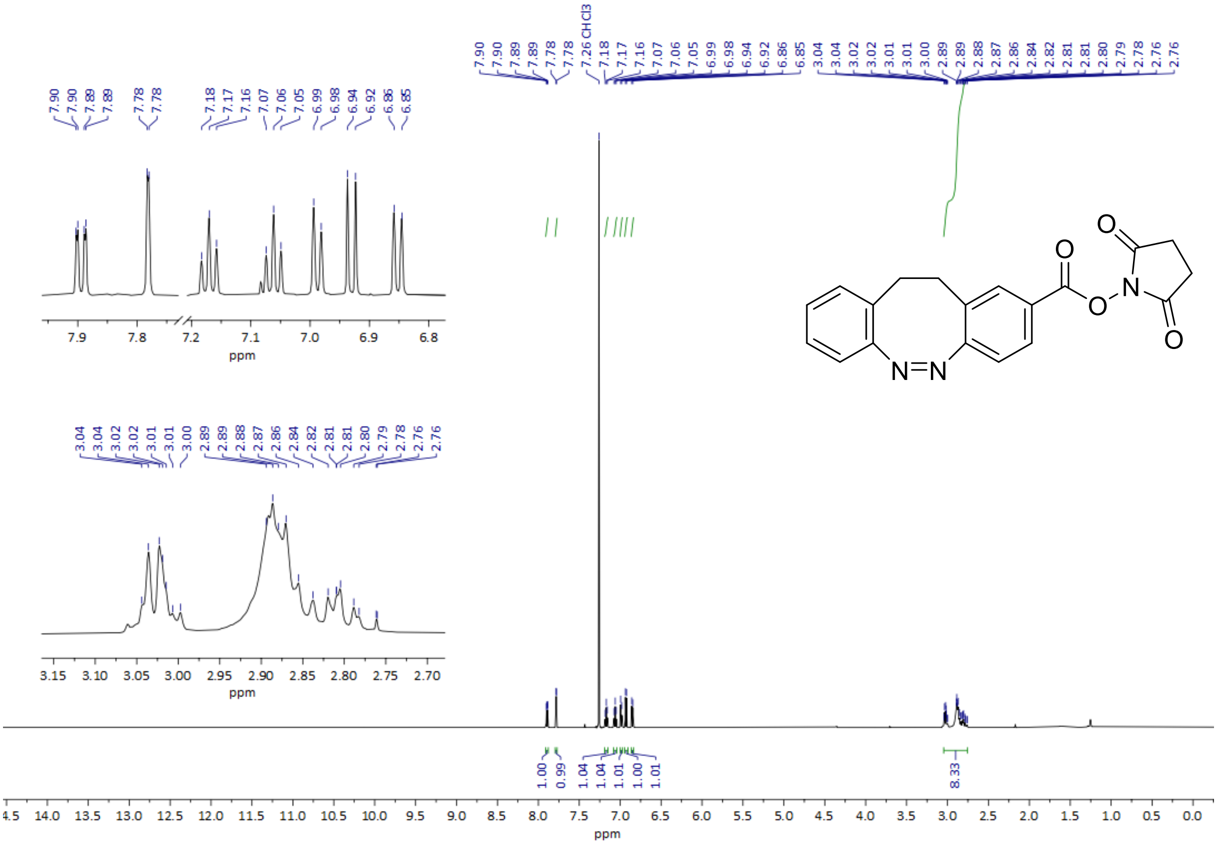

Figure S36: ^{1}H NMR spectrum of **8a** in $CDCl_3$.

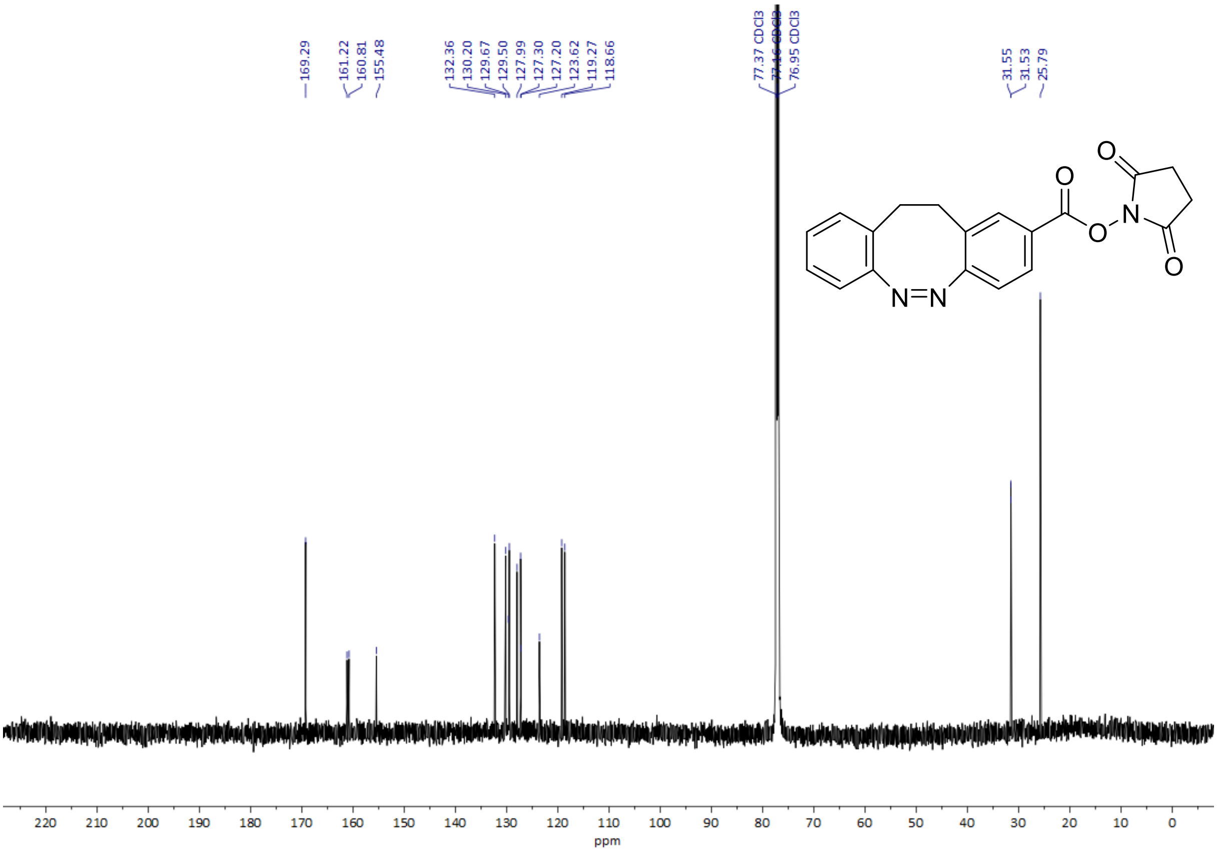

Figure S37: $^{13}C\{^{1}H\}$ NMR spectrum of **8a** in $CDCl_3$.

Bis(2,5-dioxopyrrolidin-1-yl) (Z)-11,12-dihydrodibenzo[c,g][1,2]diazocine-2,9-dicarboxylate (**8b**)

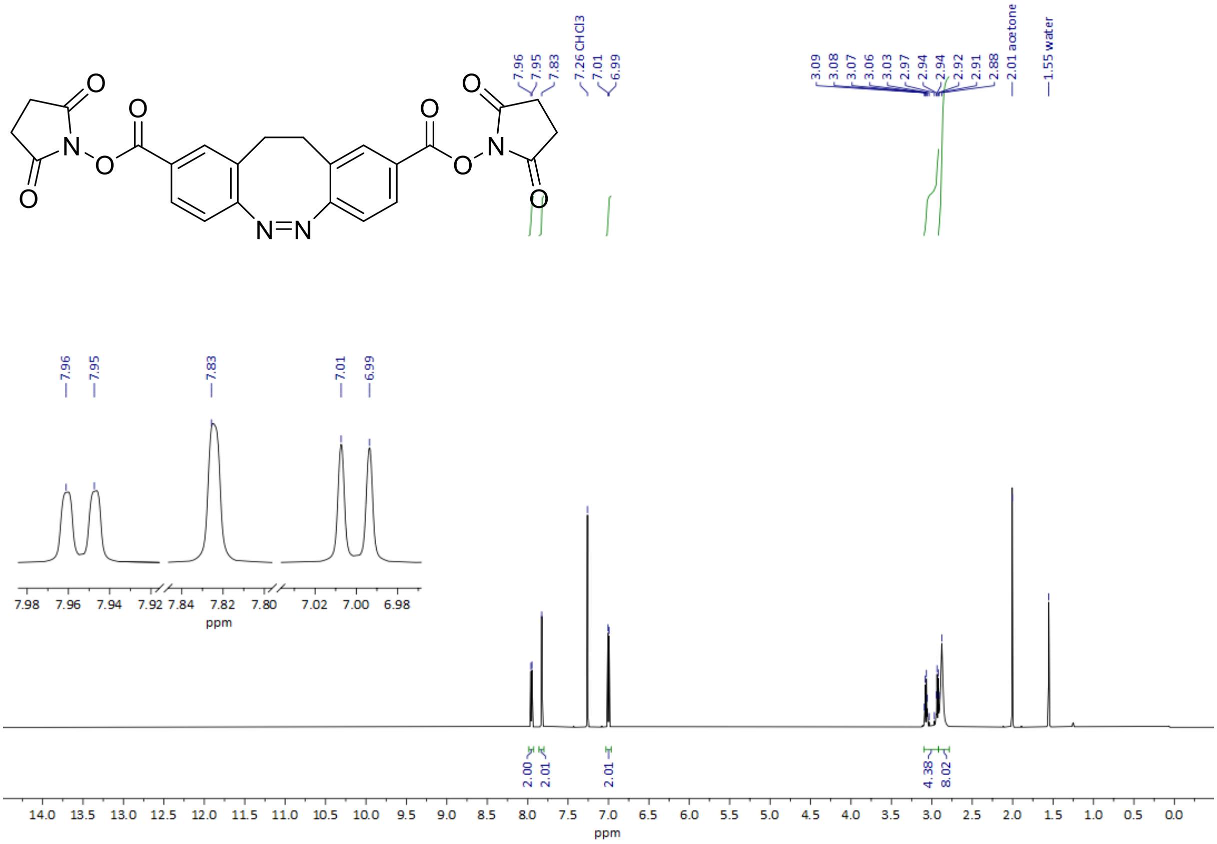

Figure S38: ^{1}H NMR spectrum of **8b** in $CDCl_3$.

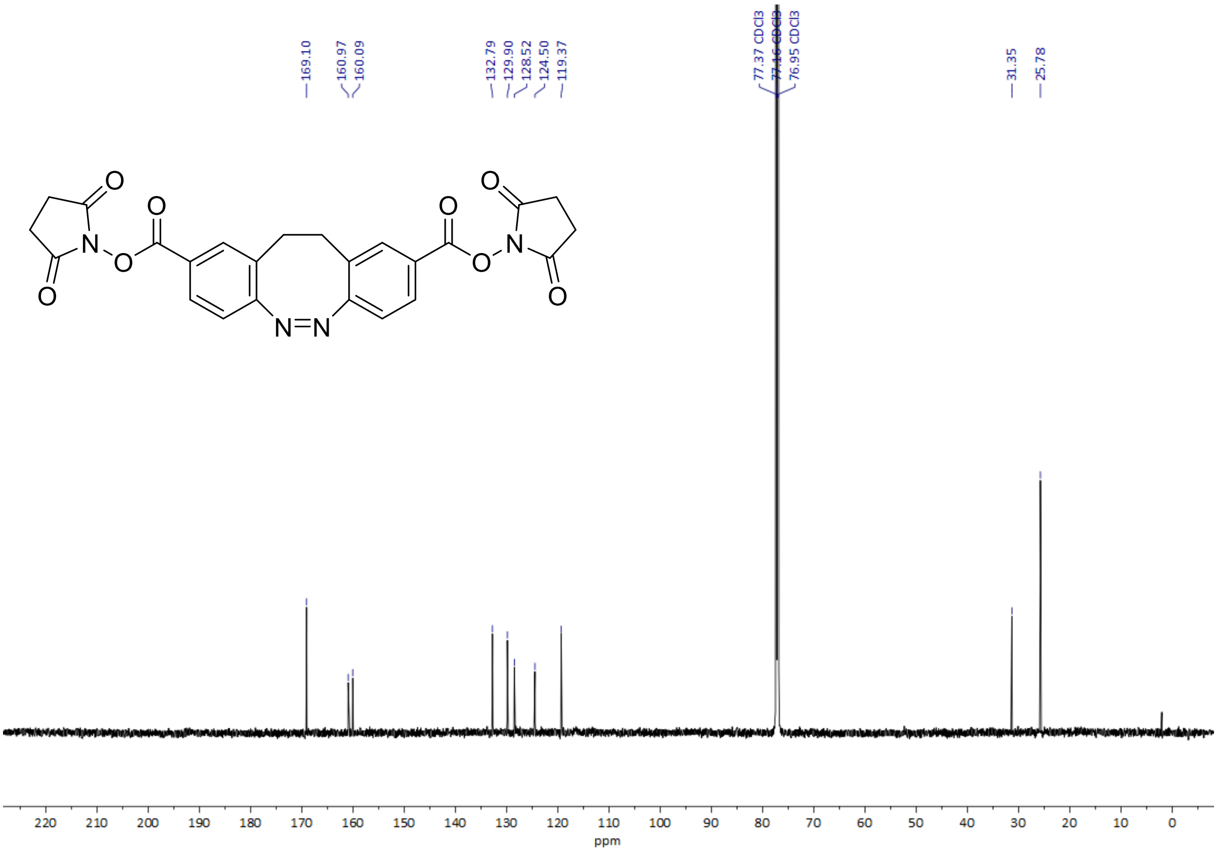

Figure S39: $^{13}C\{^{1}H\}$ NMR spectrum of **8b** in $CDCl_3$.

Di-tert-butyl 2,2'-((4,4'-(diazene-1,2-diyl)bis(benzoyl))bis(azanediyl))(*E*)-dipropionate (**10a**)

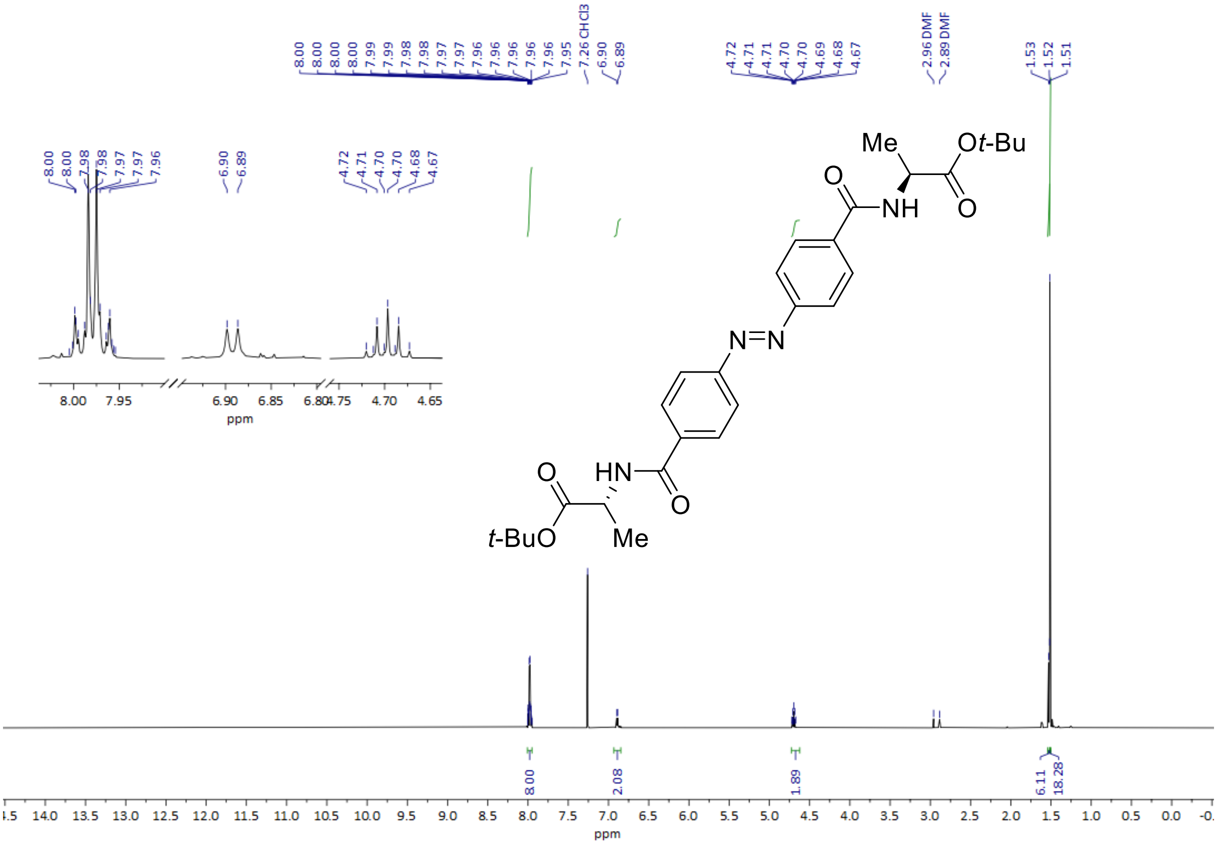

Figure S40: ^{1}H NMR spectrum of **10a** in $CDCl_3$.

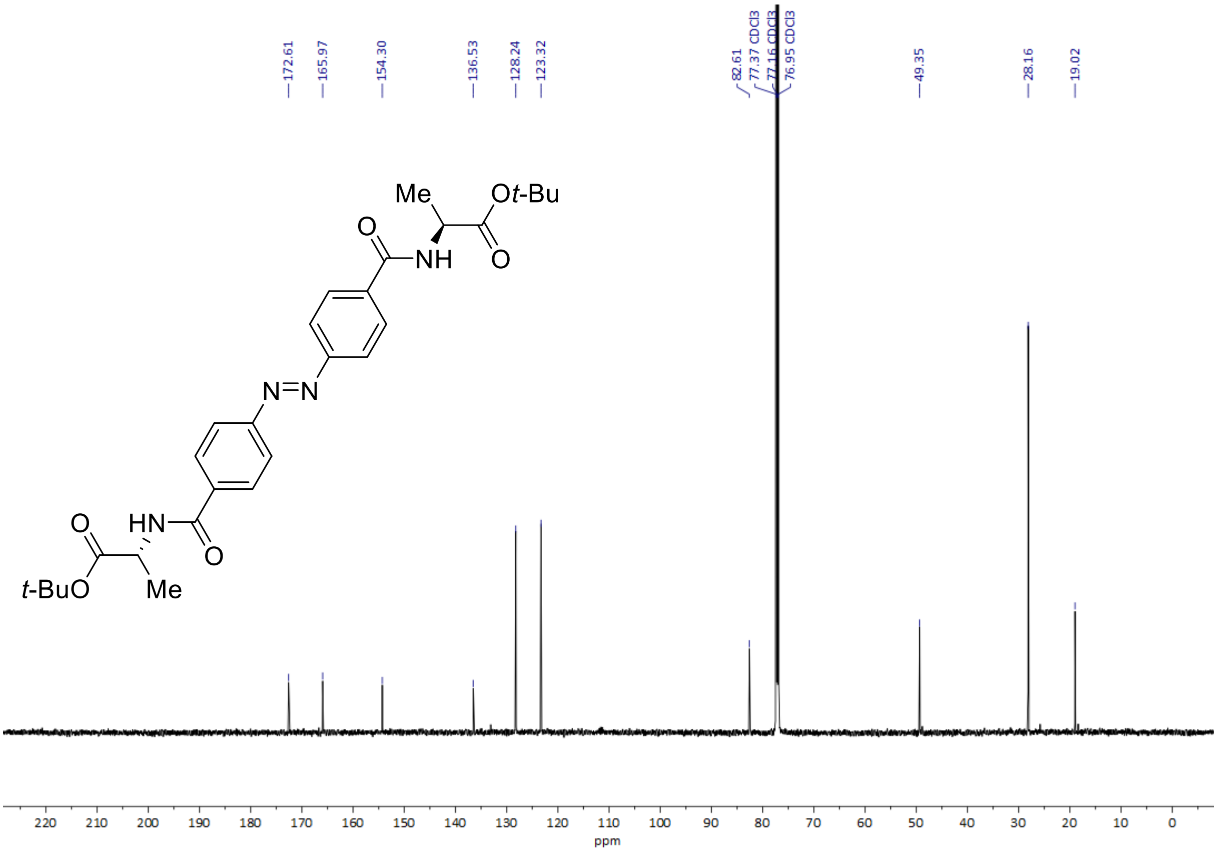

Figure S41: $^{13}C\{^{1}H\}$ NMR spectrum of **10a** in $CDCl_3$.

Di-tert-butyl 2,2'-((11,12-dihydrodibenzo[c,g][1,2]diazocine-2,9-dicarbonyl)bis(azane-diyl))(*Z*)-dipropionate (**10b**)

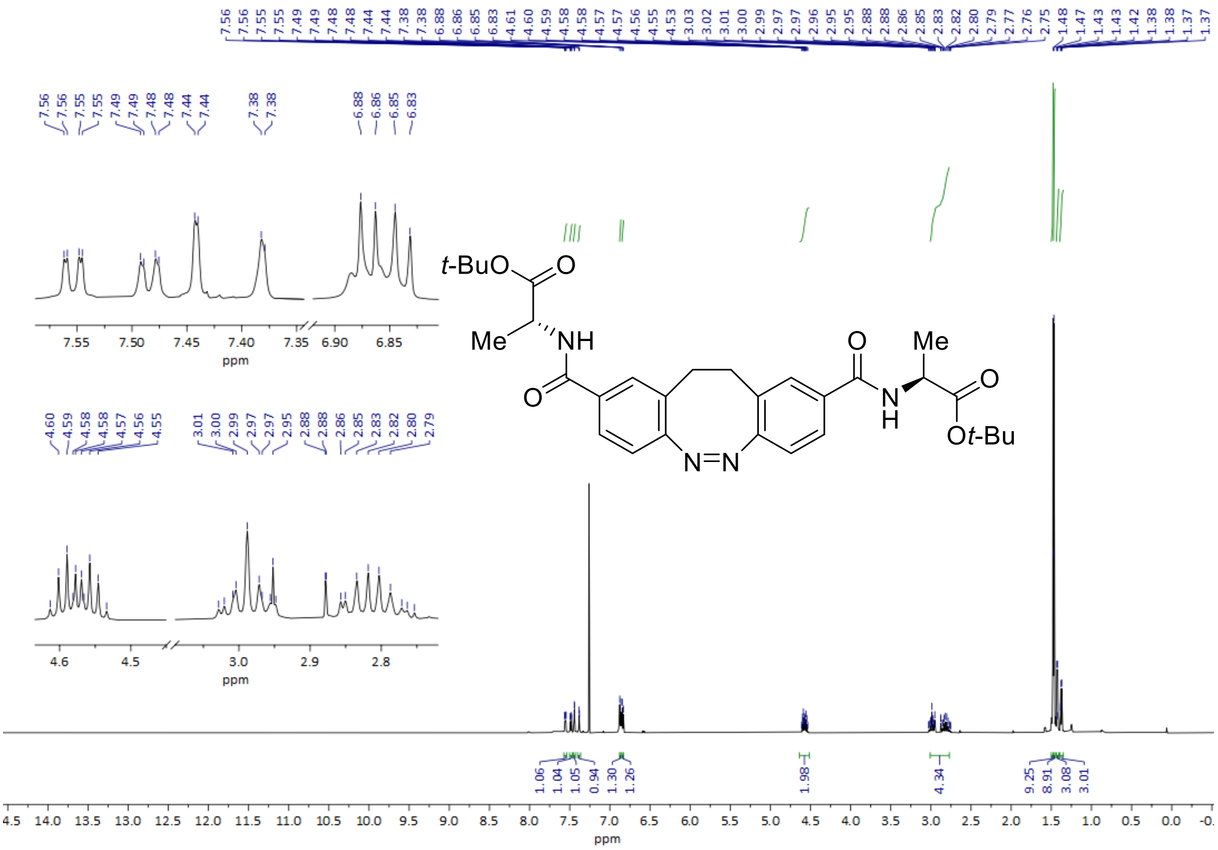

Figure S42:^{1}H NMR spectrum of **10b** in $CDCl_3$.

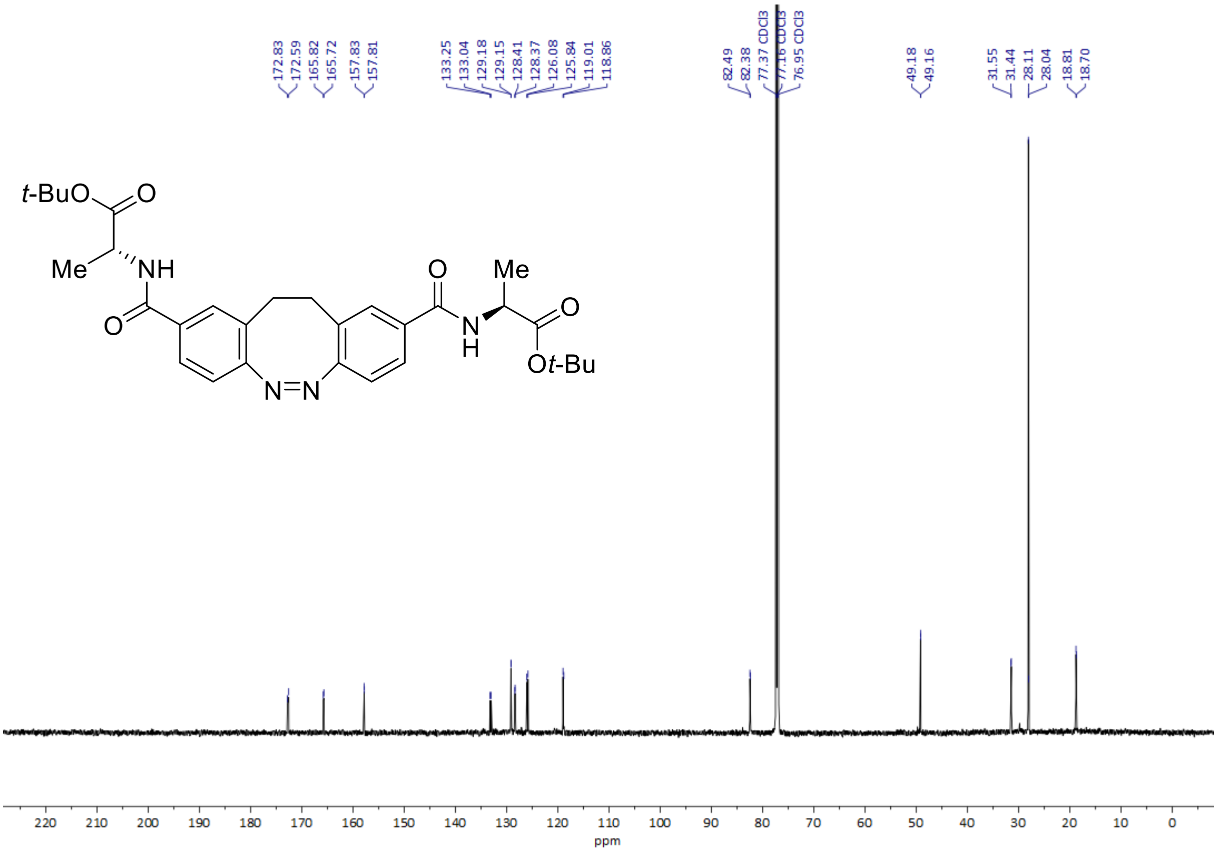

Figure S43: $^{13}C\{^1H\}$ NMR spectrum of **10b** in $CDCl_3$.

N-Hydroxysuccinimidyl formate (**2**)

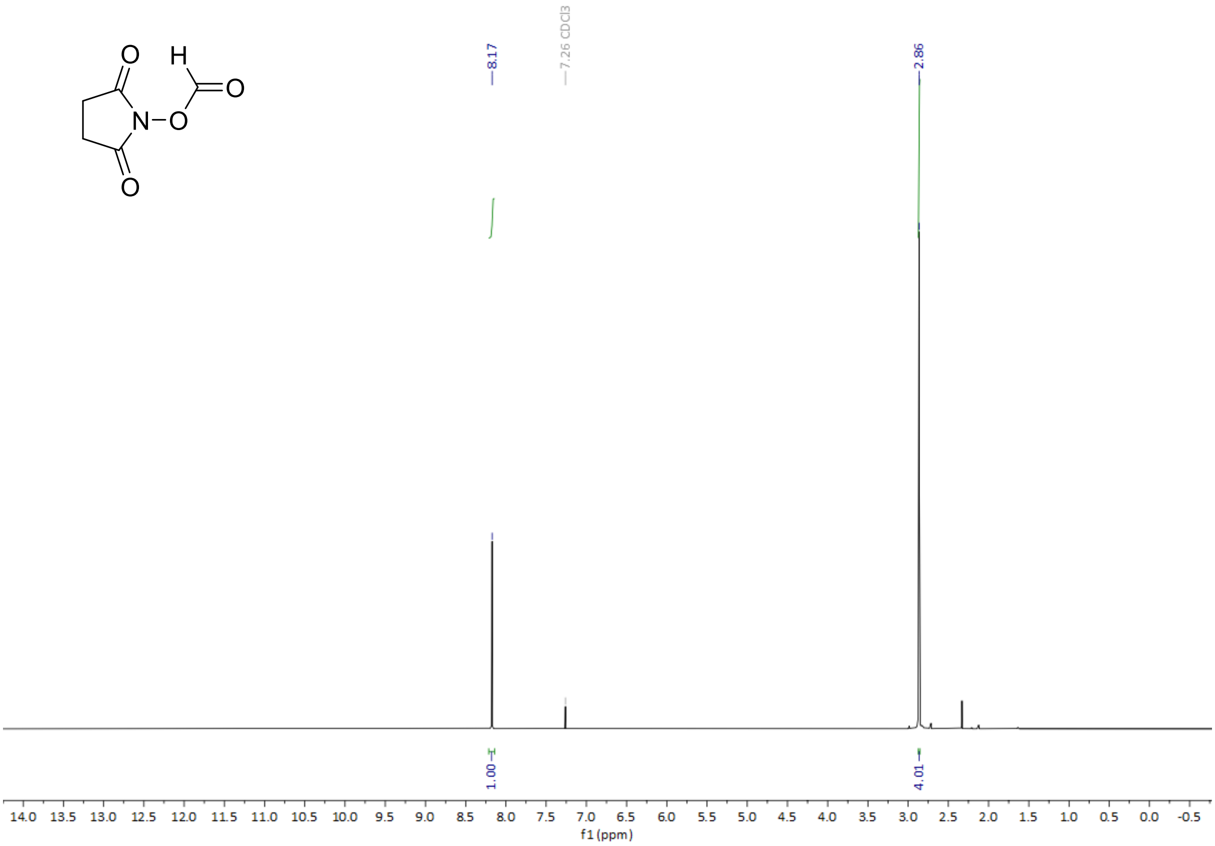

Figure S44: ^{1}H NMR spectrum of **2** in $CDCl_3$.

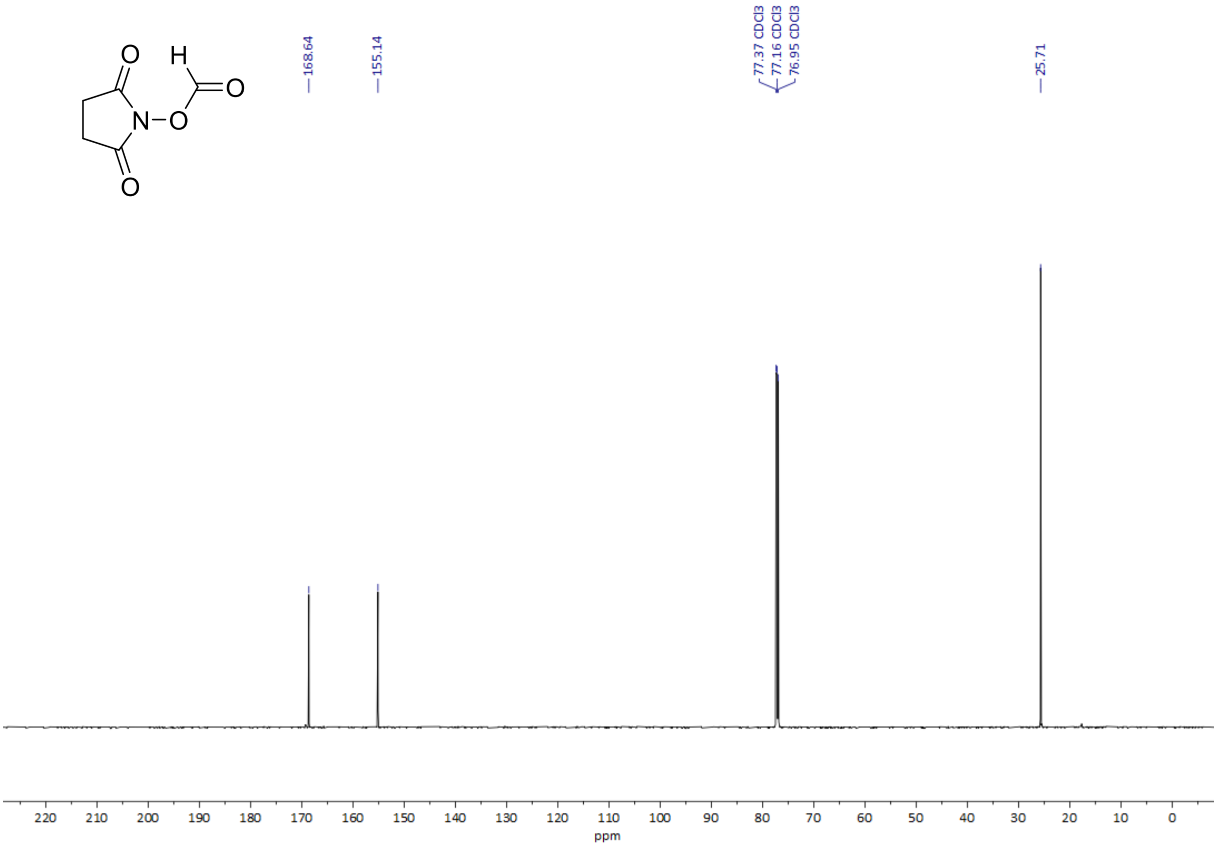

Figure S45: $^{13}C\{^{1}H\}$ NMR spectrum of **2** in $CDCl_3$.

(*E*)-1-(4-Iodophenyl)-2-phenyldiazene (**1a**)

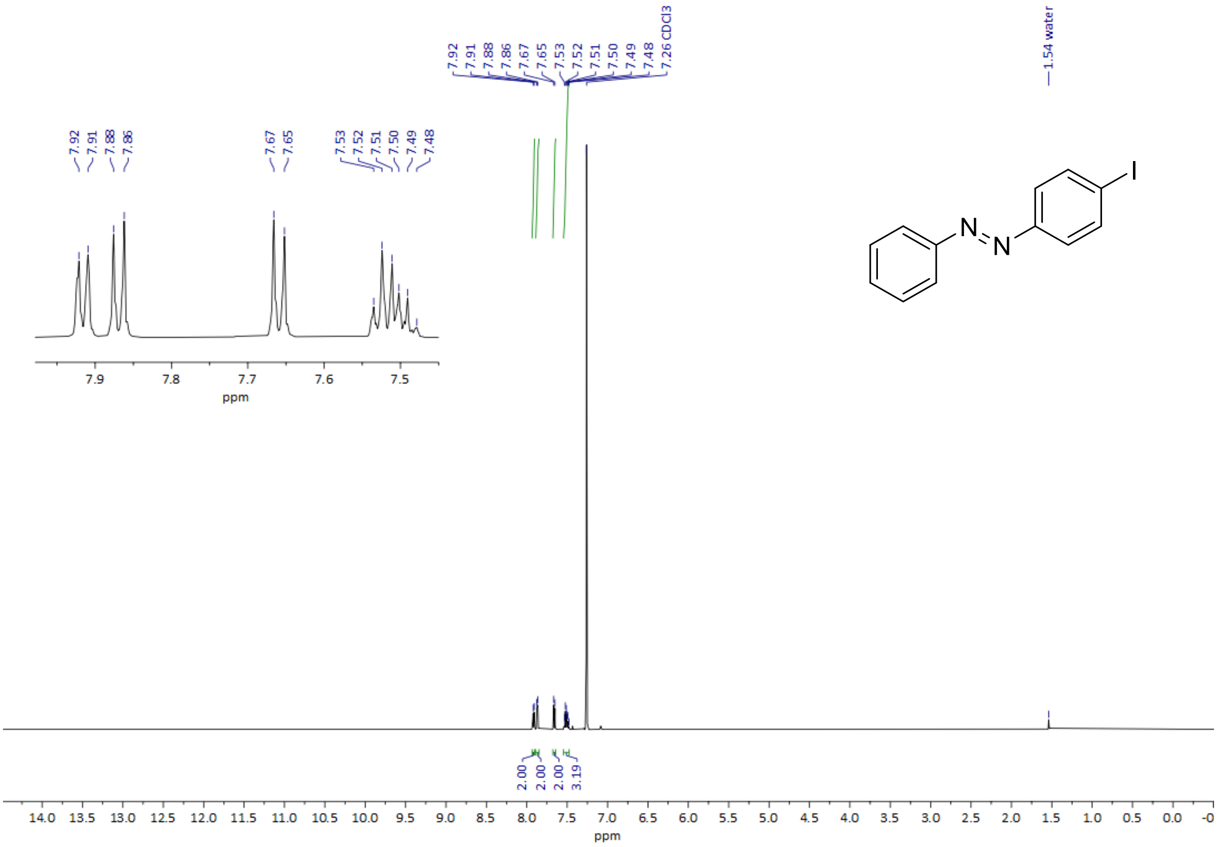

Figure S46: ^{1}H NMR spectrum of **1a** in $CDCl_3$.

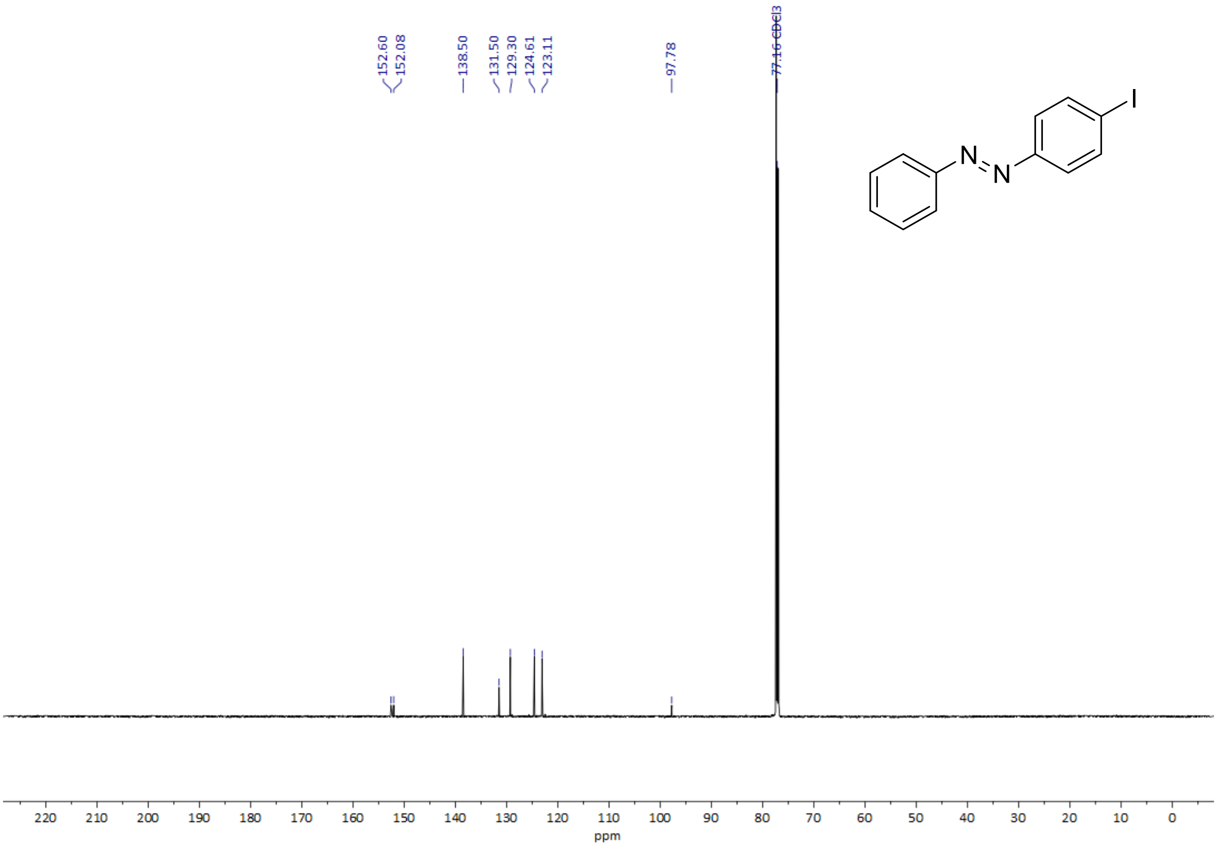

Figure S47: $^{13}C\{^{1}H\}$ NMR spectrum of **1a** in $CDCl_3$.

(*E*)-1-(3-Iodophenyl)-2-phenyldiazene (**1b**)

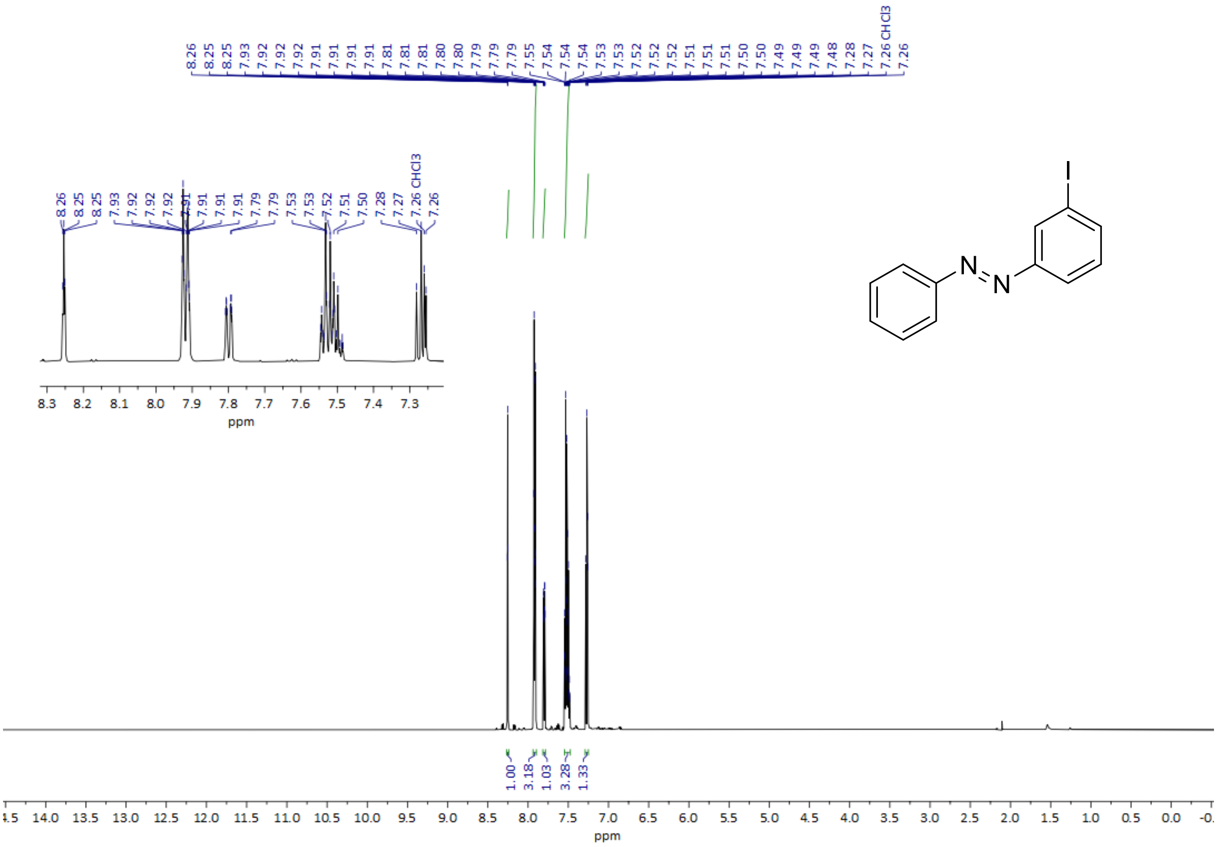

Figure S48: ^{1}H NMR spectrum of **1b** in $CDCl_3$.

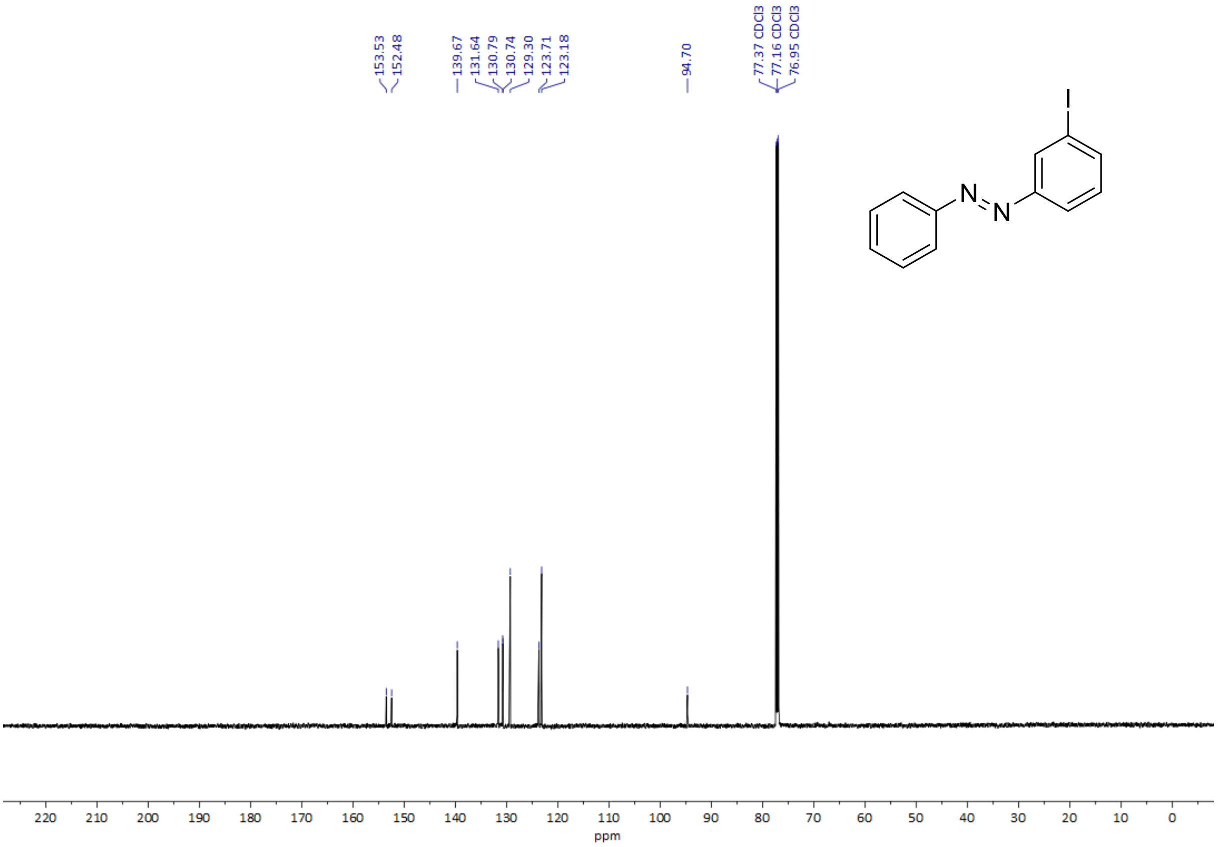

Figure S49: $^{13}C\{^{1}H\}$ NMR spectrum of **1b** in $CDCl_3$.

(*E*)-1-(2-Iodophenyl)-2-phenyldiazene (**1c**)

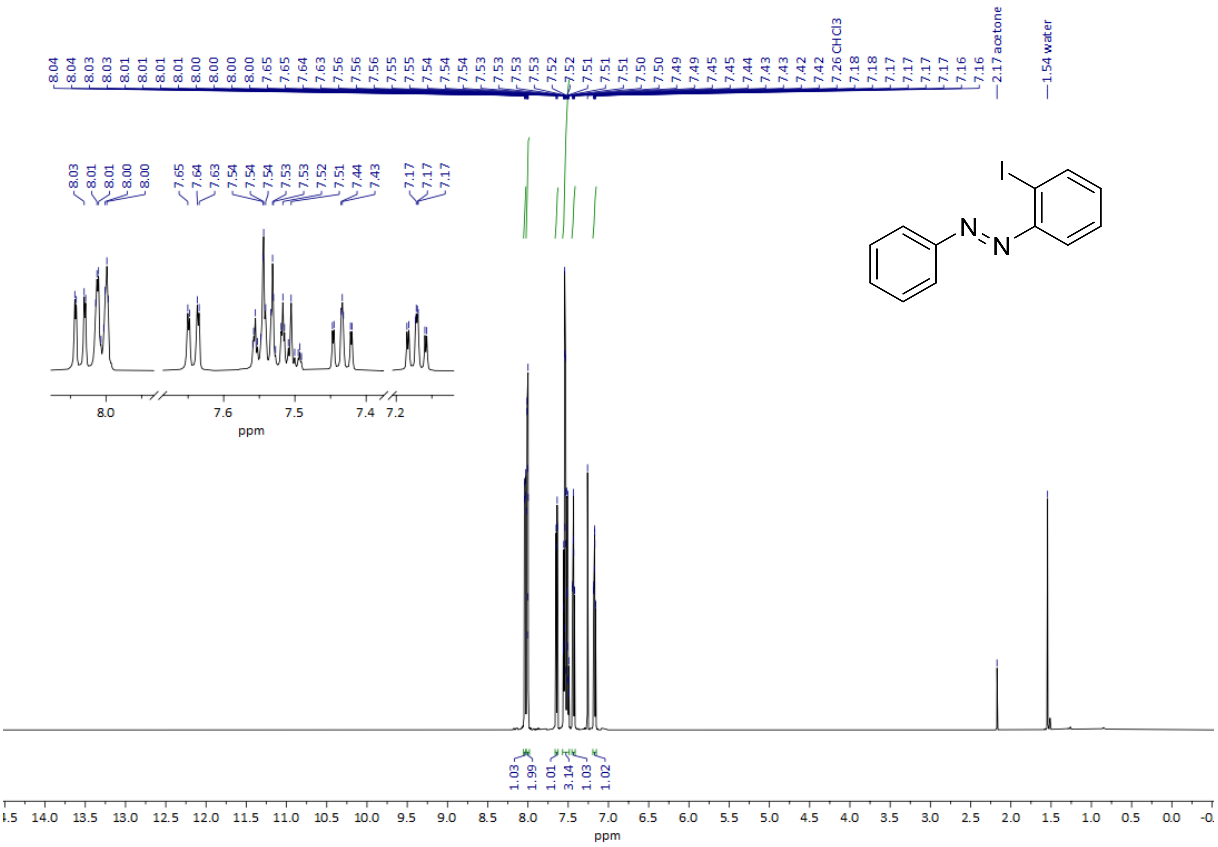

Figure S50: ^{1}H NMR spectrum of **1c** in $CDCl_3$.

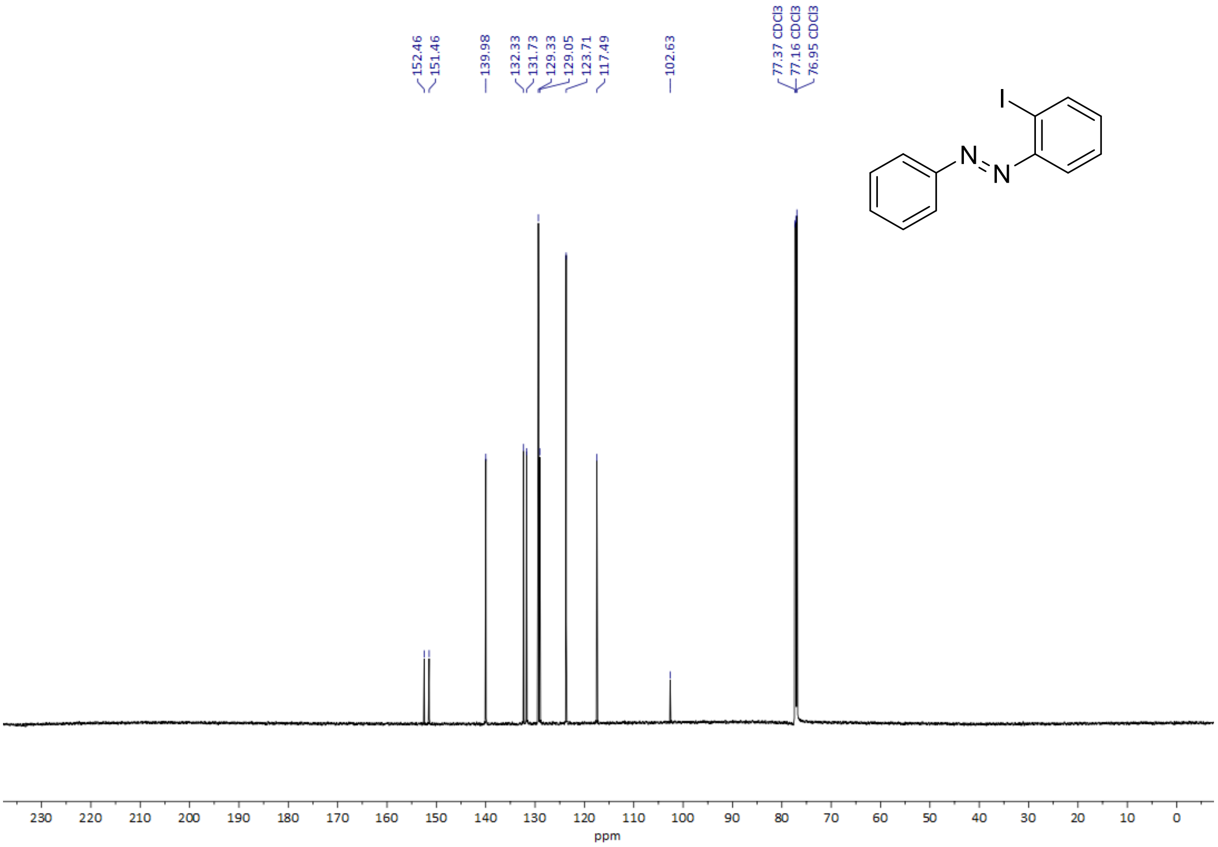

Figure S51: $^{13}C\{^{1}H\}$ NMR spectrum of **1c** in $CDCl_3$.

(*E*)-4-((4-Iodophenyl)diazenyl)phenol (**1d**)

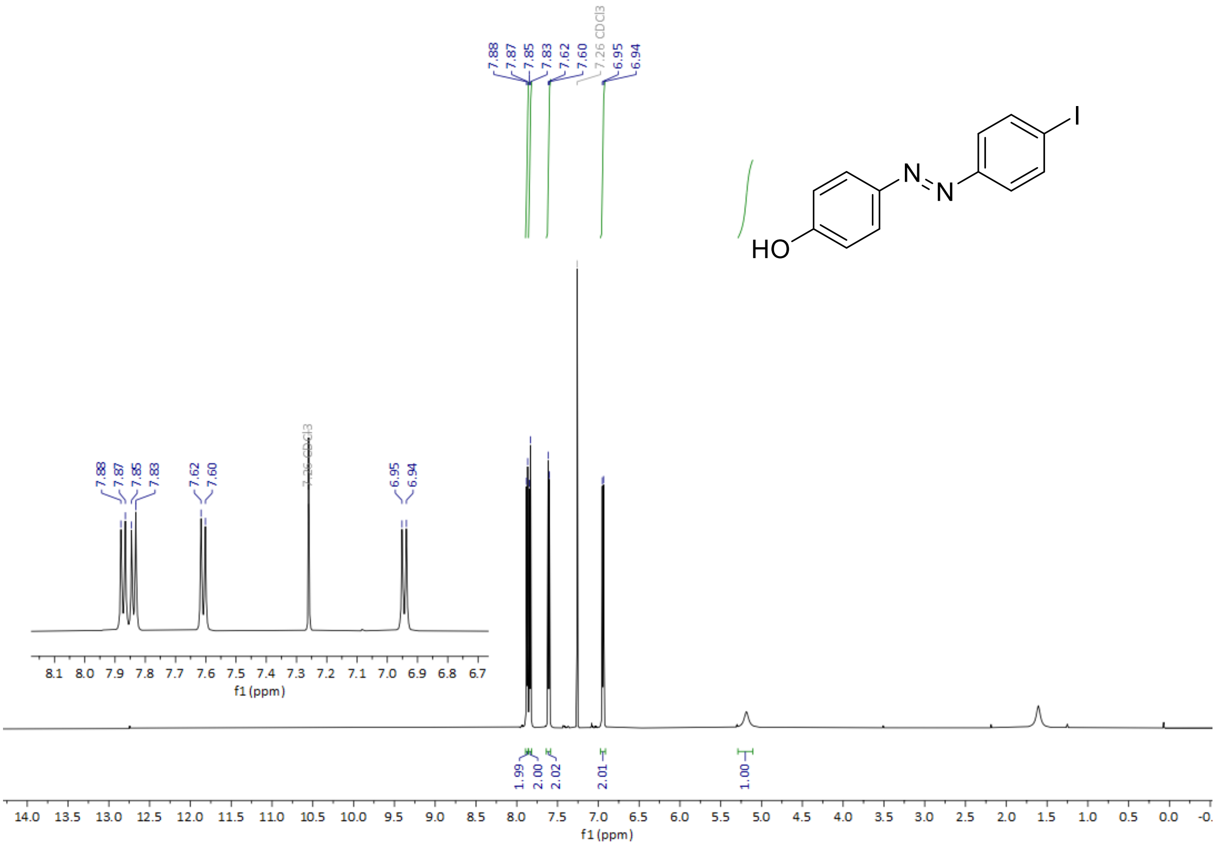

Figure S52:^{1}H NMR spectrum of **1d** in $CDCl_3$.

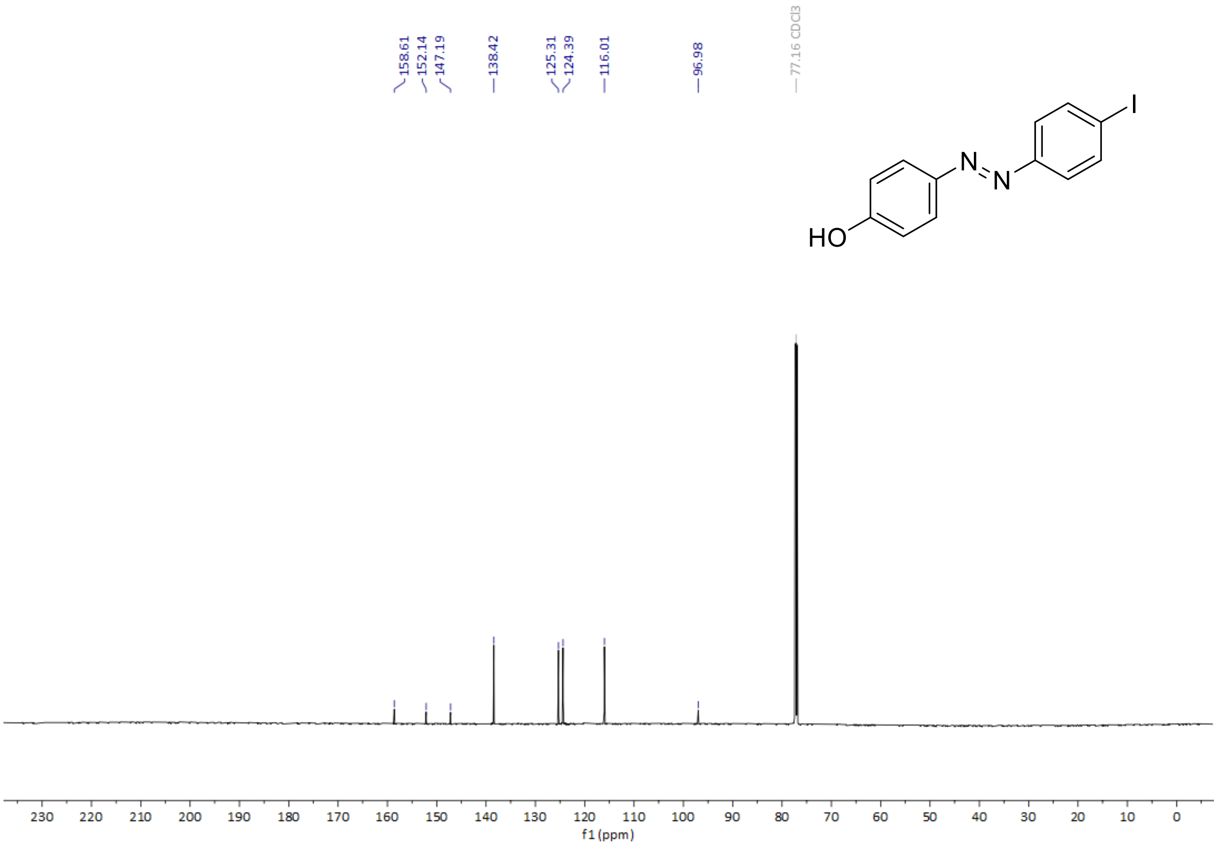

Figure S53: $^{13}C\{^1H\}$ NMR spectrum of **1d** in $CDCl_3$.

(*E*)-4-((4-Iodophenyl)diazenyl)aniline (**1e**)

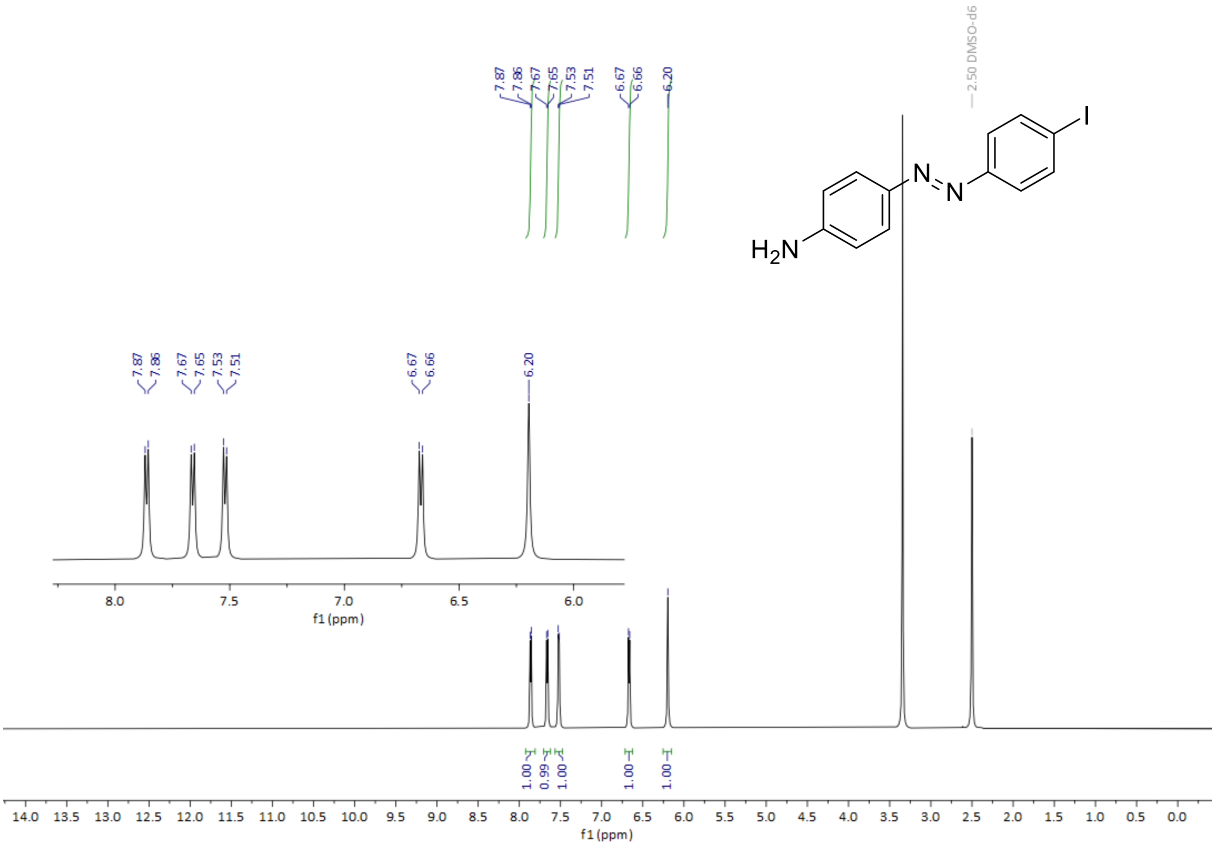

Figure S54:^{1}H NMR spectrum of **1e** in DMSO-d_6.

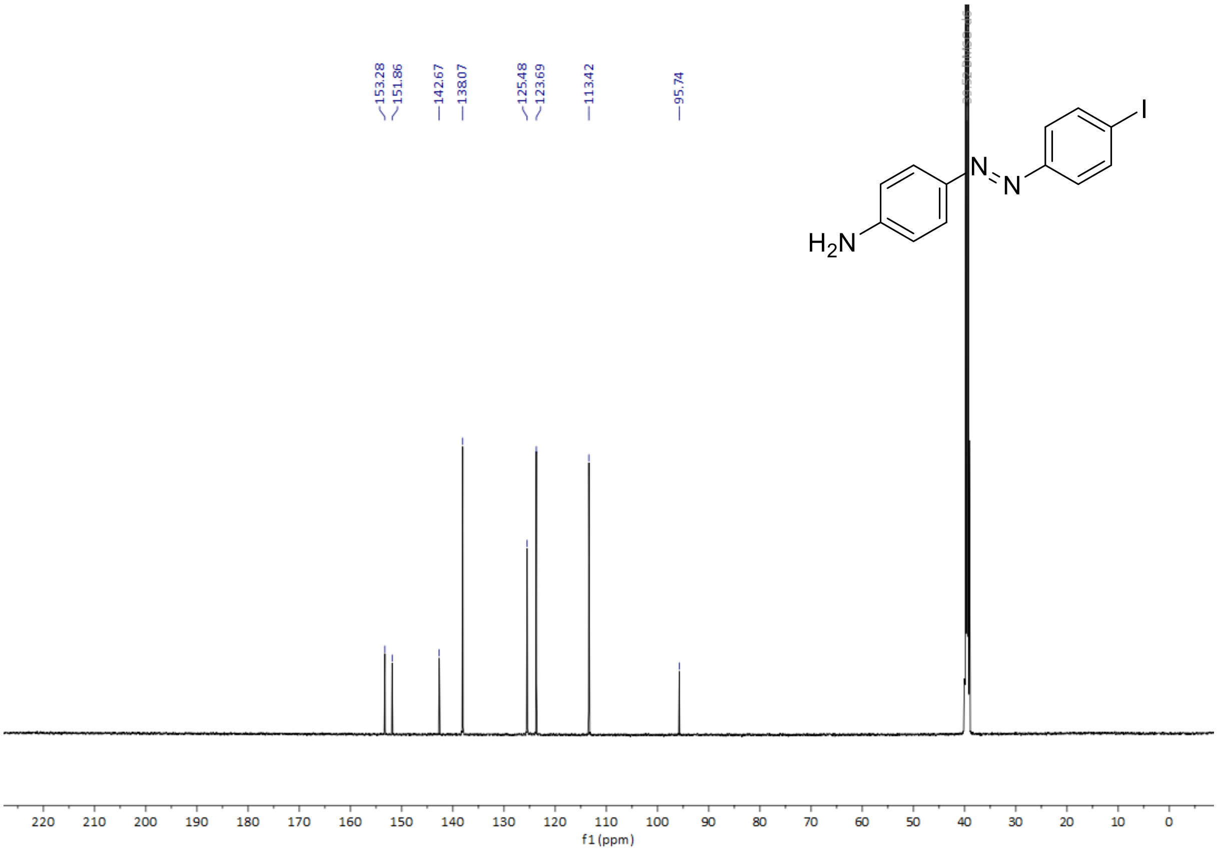

Figure S55: ^{13}C{^{1}H} NMR spectrum of **1e** in DMSO-d_6.

(*E*)-*N*-(4-((4-Iodophenyl)diazenyl)phenyl)acetamide (**1f**)

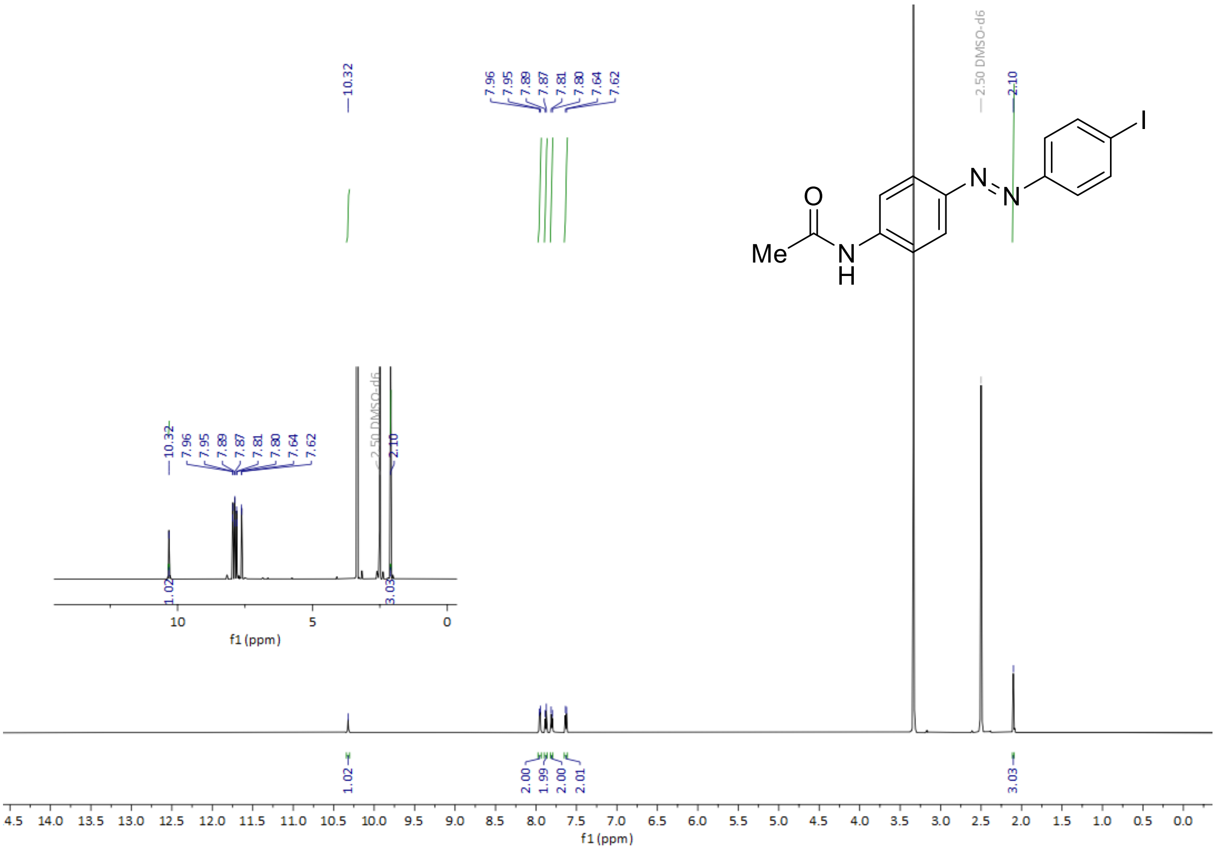

Figure S56:^{1}H NMR spectrum of **1f** in DMSO-d_6.

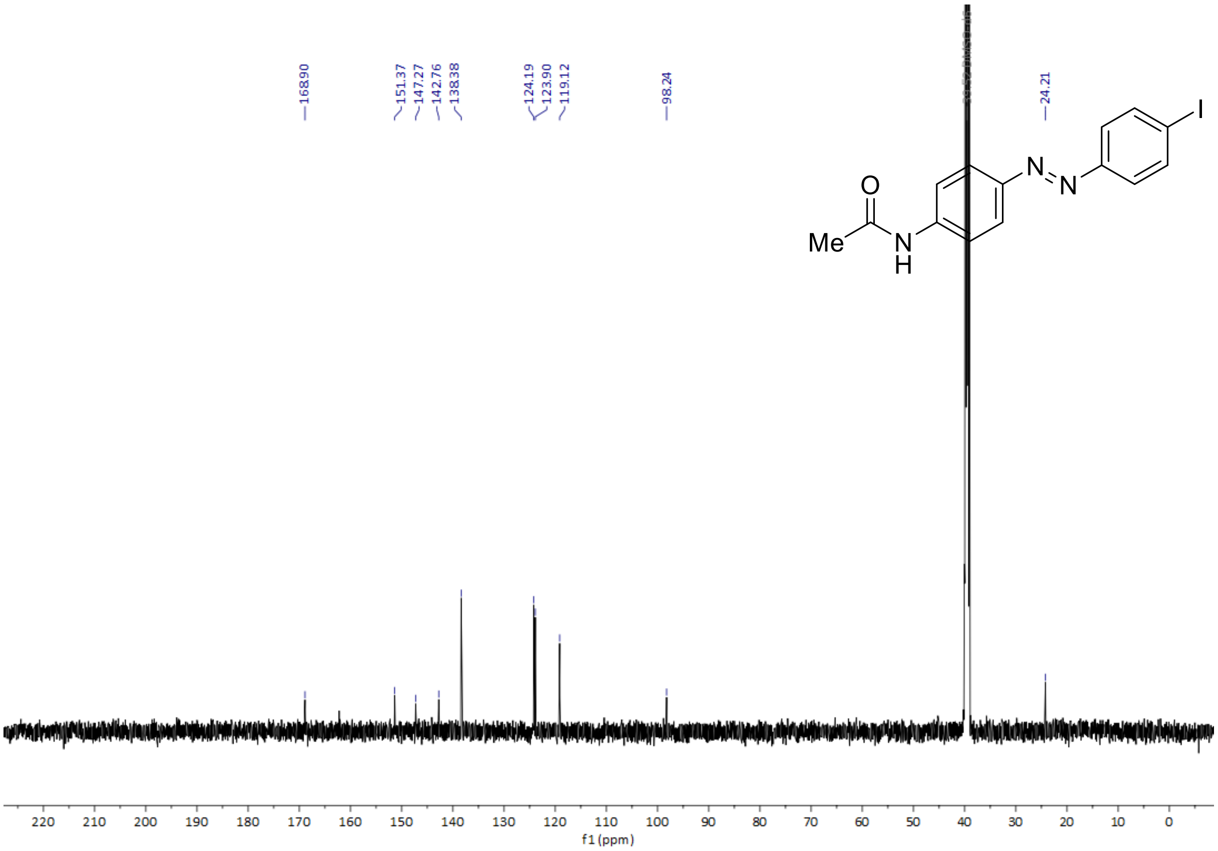

Figure S57: ^{13}C{^{1}H} NMR spectrum of **1f** in DMSO-d_6.

(*E*)-1-(4-Iodophenyl)-2-(4-nitrophenyl)diazene (**1g**)

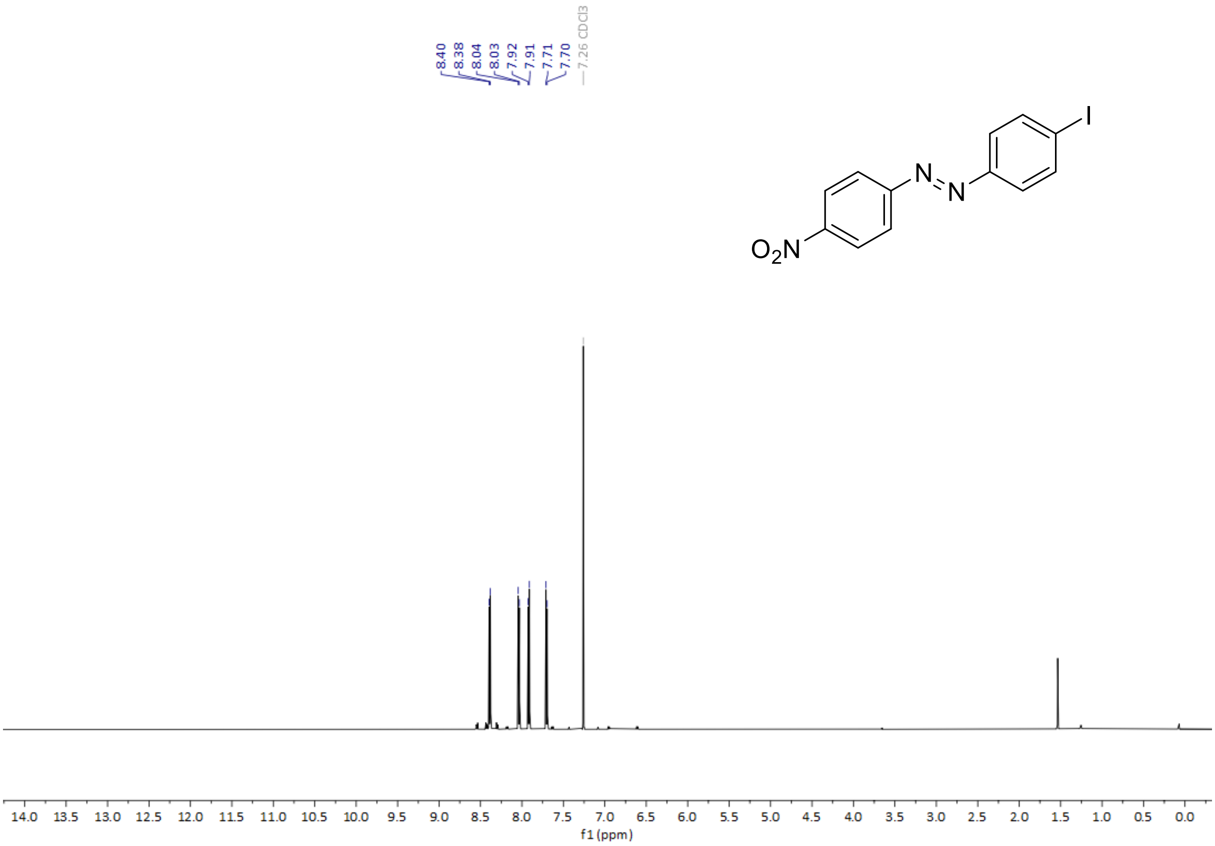

Figure S58:^{1}H NMR spectrum of **1g** in $CDCl_3$.

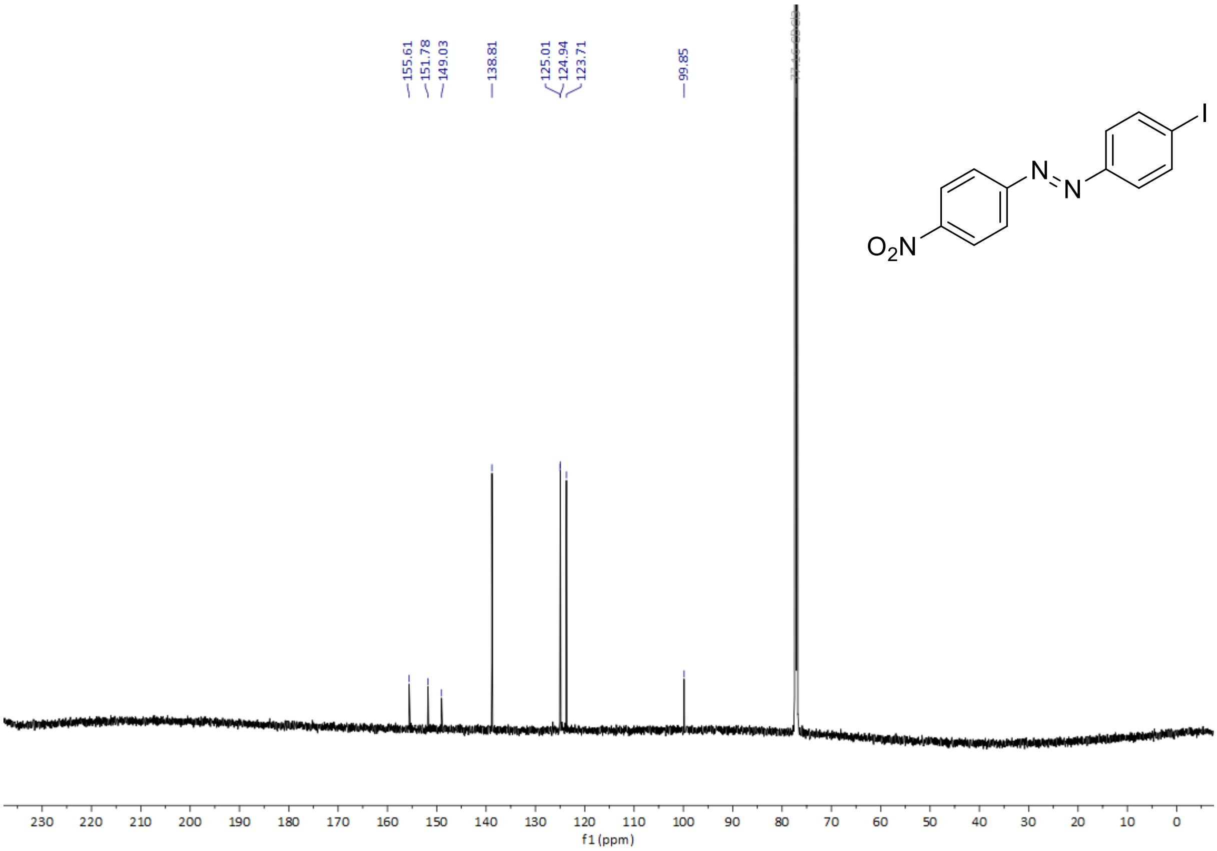

Figure S59: $^{13}C\{^{1}H\}$ NMR spectrum of **1g** in $CDCl_3$.

(*E*)-1-(4-(Allyloxy)phenyl)-2-(4-iodophenyl)diazene (**1h**)

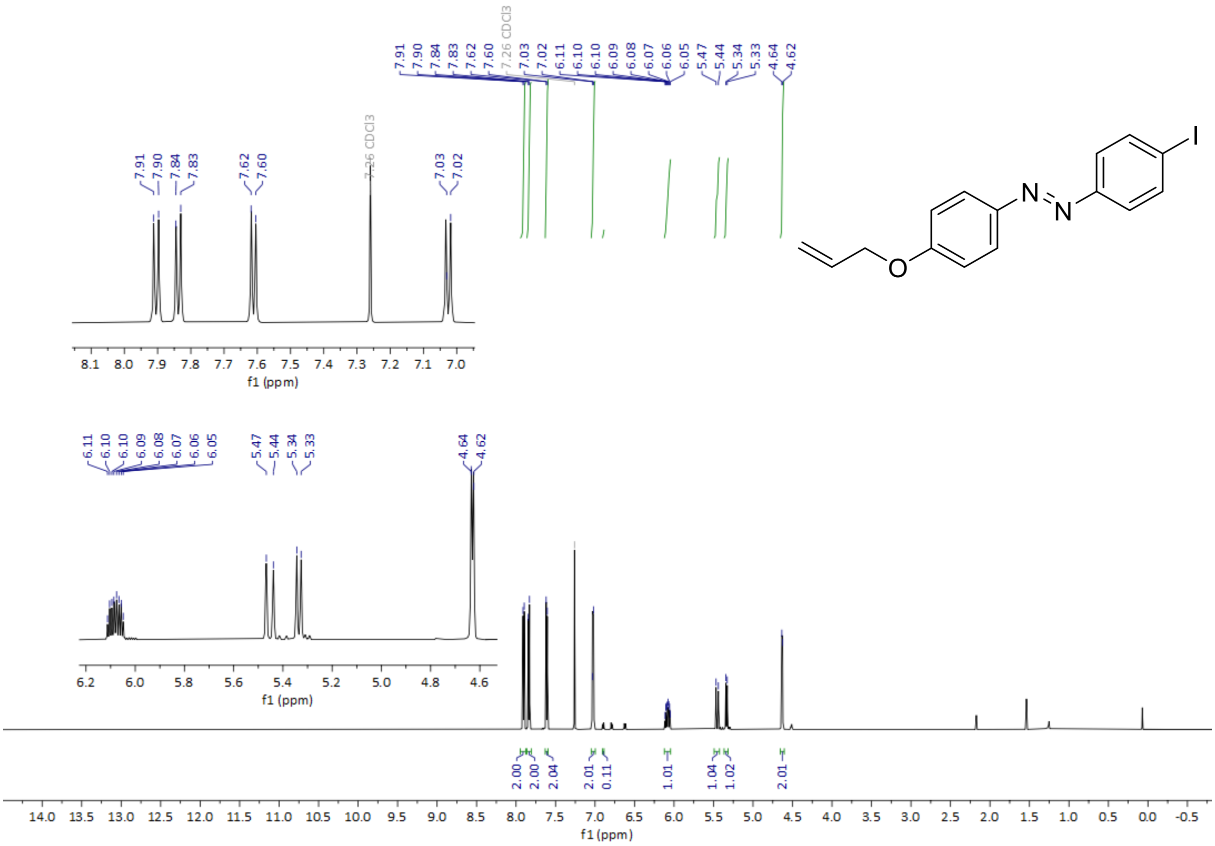

Figure S60:^{1}H NMR spectrum of **1h** in $CDCl_3$.

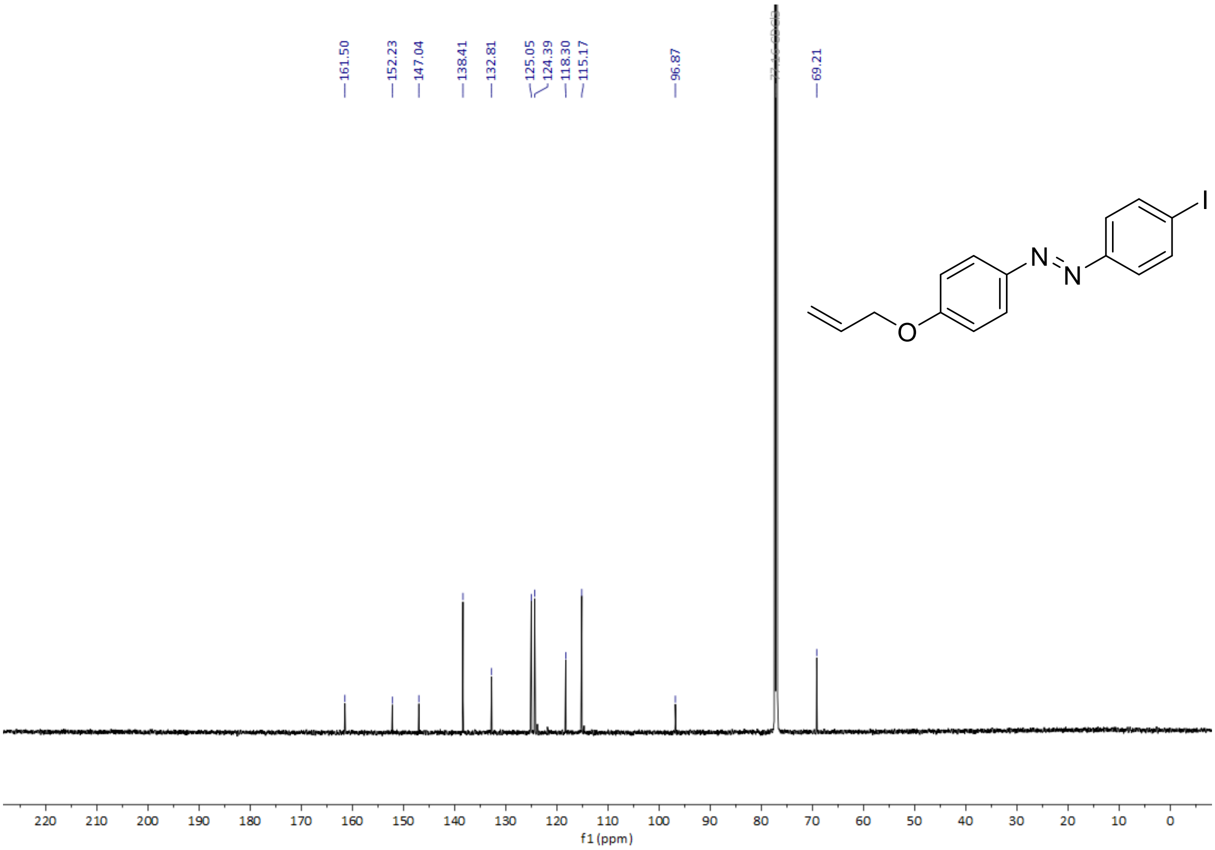

Figure S61: $^{13}C\{^{1}H\}$ NMR spectrum of **1h** in $CDCl_3$.

(*E*)-9-(4-((4-Iodophenyl)diazenyl)phenoxy)nonan-1-ol (**1i**)

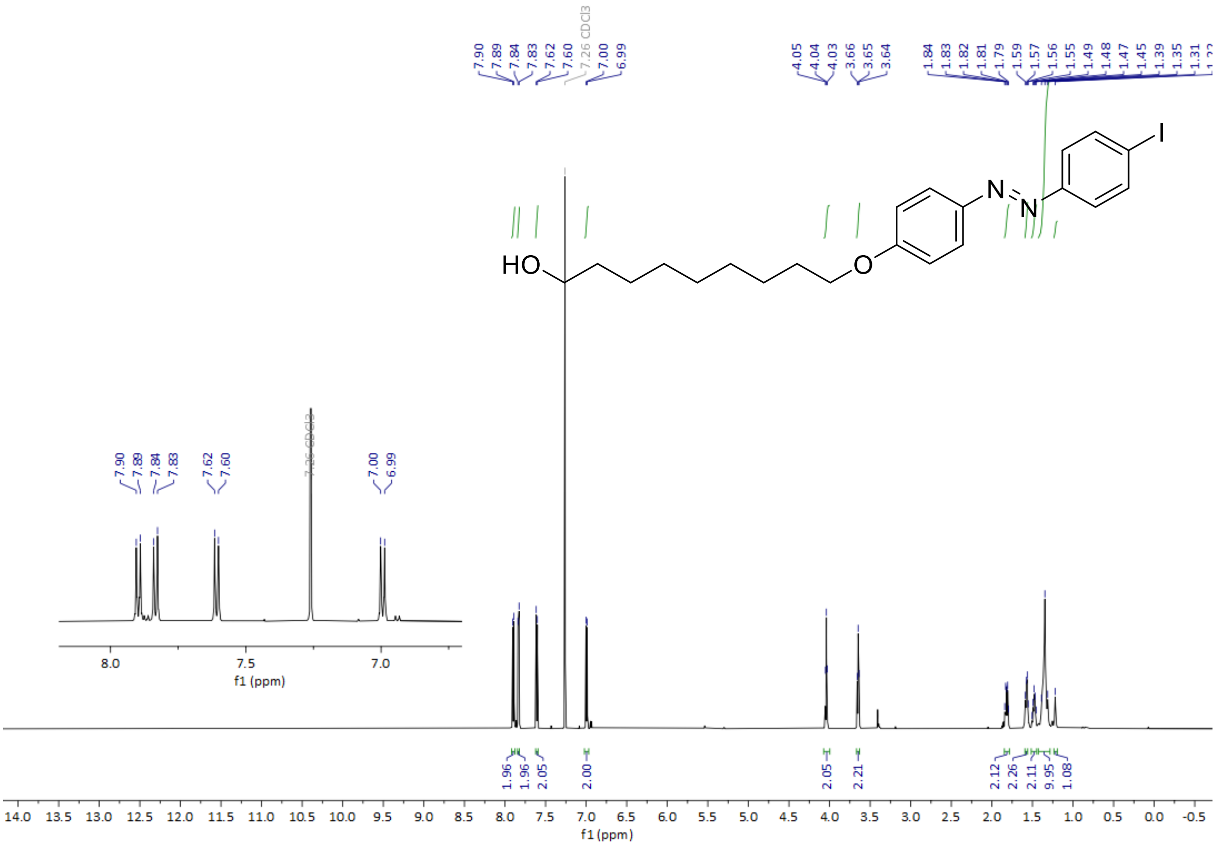

Figure S62: ^{1}H NMR spectrum of **1i** in $CDCl_3$.

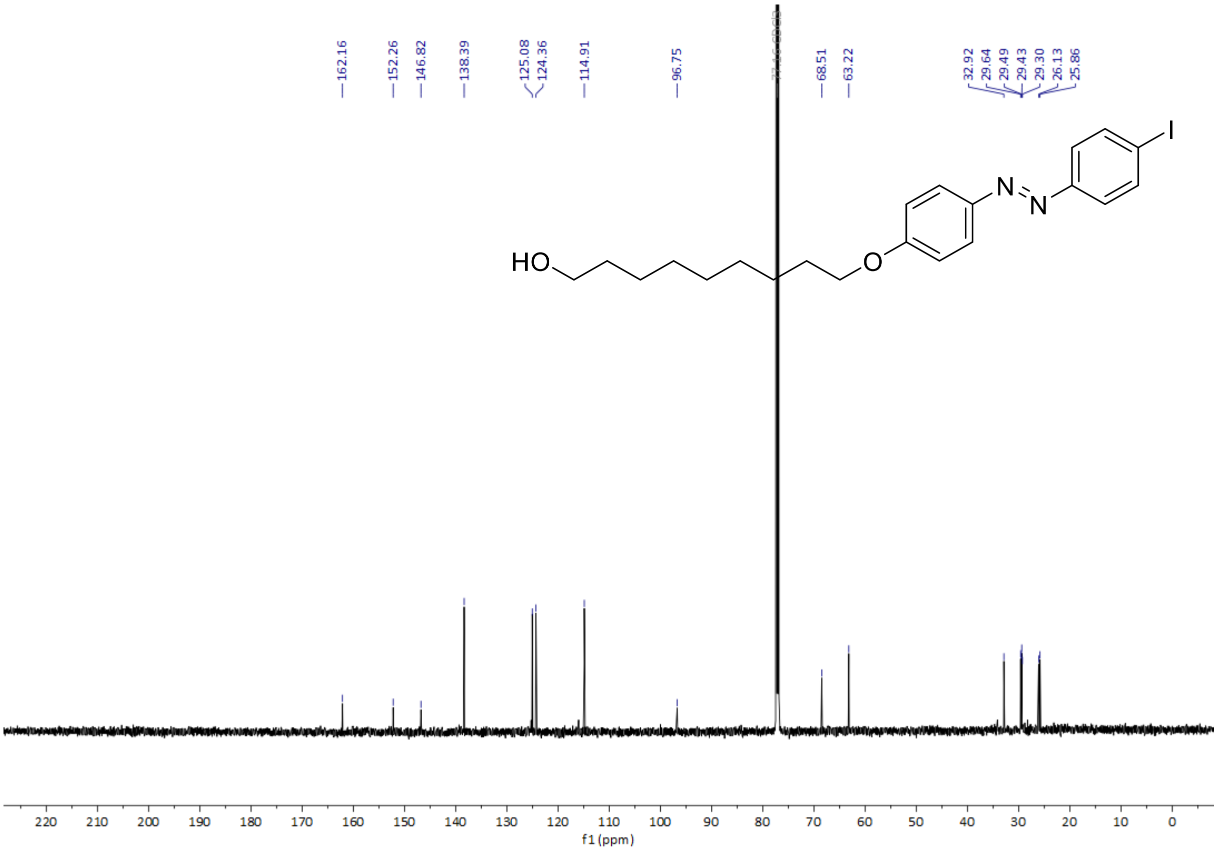

Figure S63: $^{13}C\{^{1}H\}$ NMR spectrum of **1i** in $CDCl_3$.

(*E*)-4-((4-Iodo-2,6-dimethylphenyl)diazenyl)-3,5-dimethylphenol (**S1**)

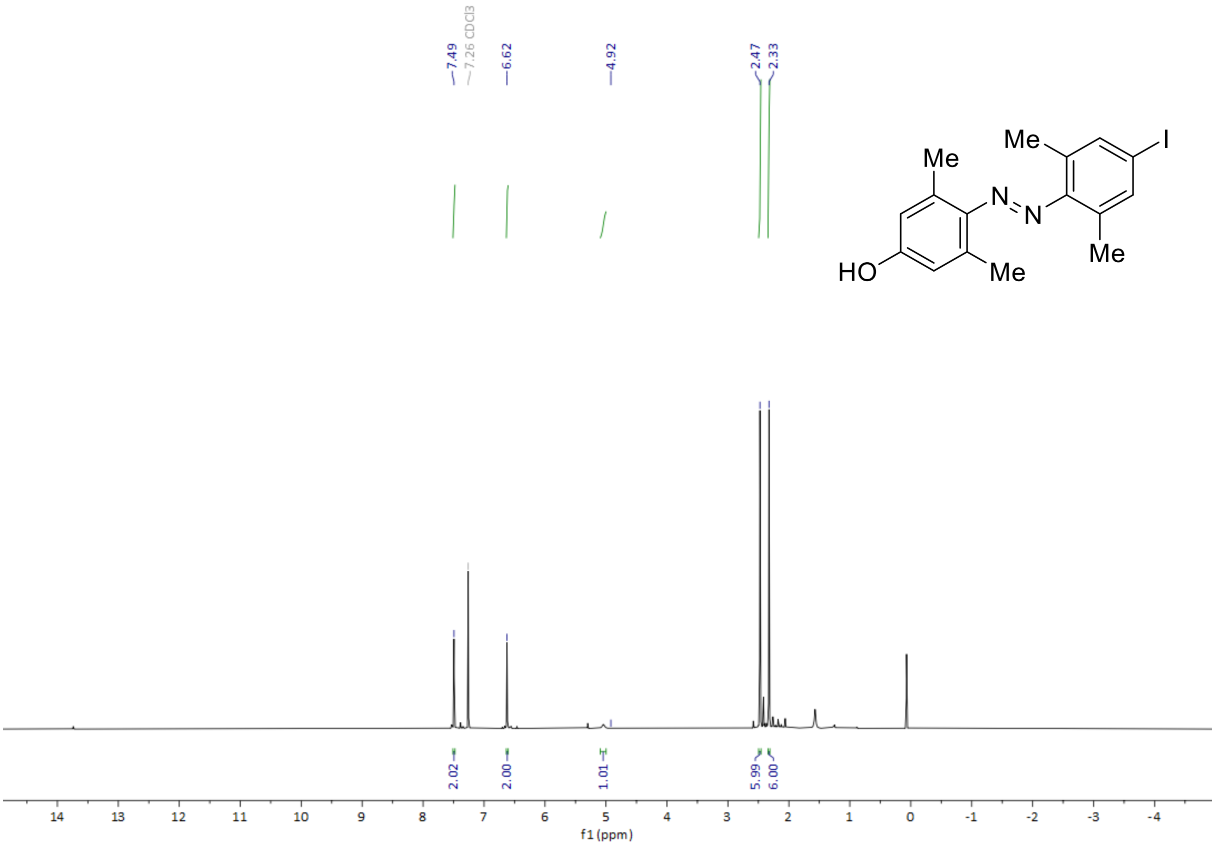

Figure S64: ^{1}H NMR spectrum of **S1** in $CDCl_3$.

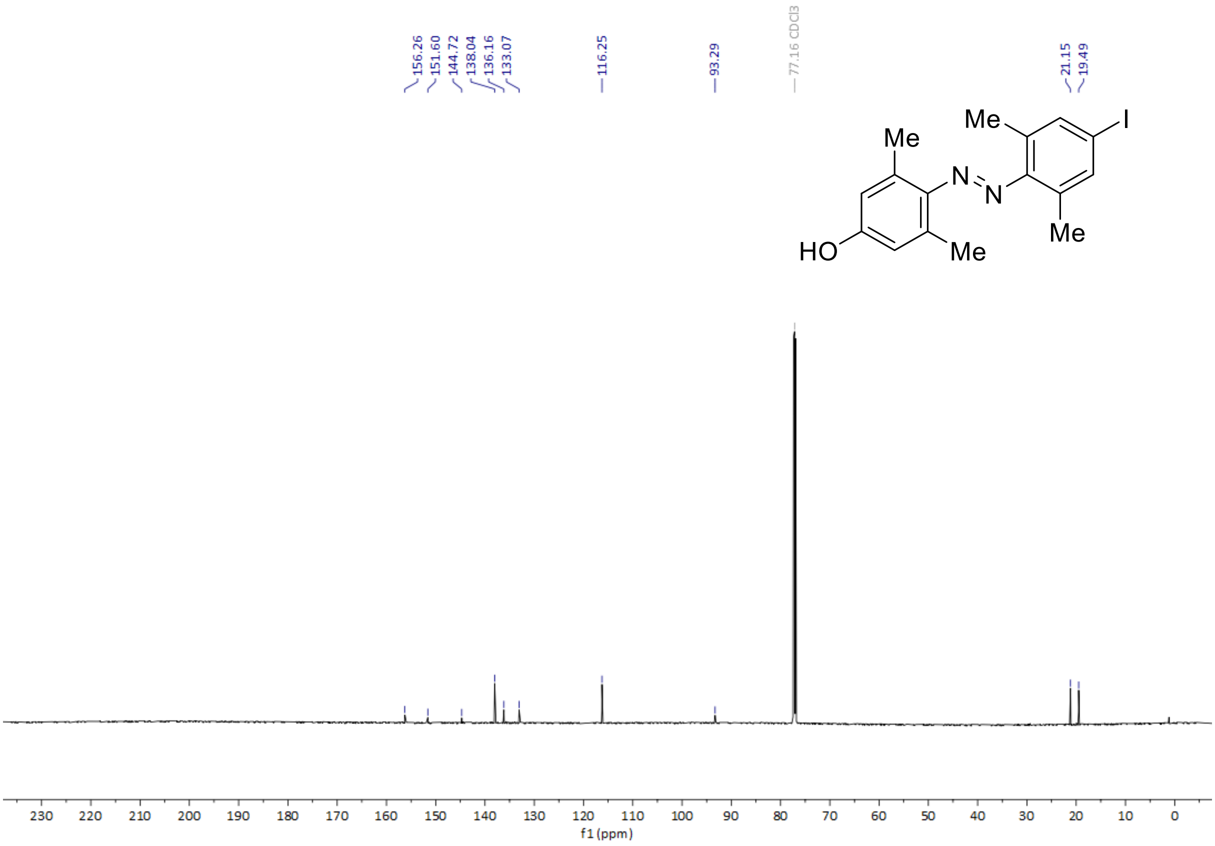

Figure S65: $^{13}C\{^{1}H\}$ NMR spectrum of **S1** in $CDCl_3$.

(*E*)-1-(2,6-Dimethyl-4-(nonyloxy)phenyl)-2-(4-iodo-2,6-dimethylphenyl)diazene (**1j**)

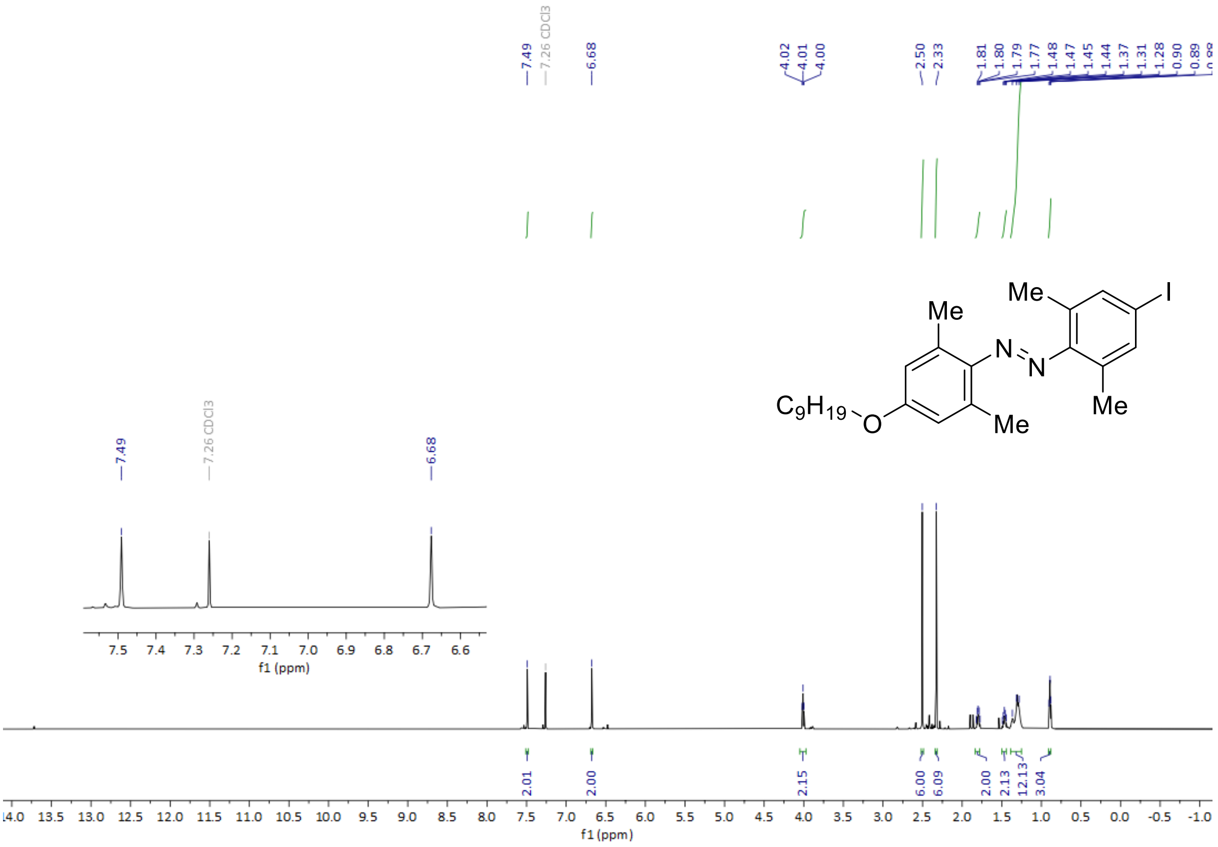

Figure S66:^{1}H NMR spectrum of **1j** in $CDCl_3$.

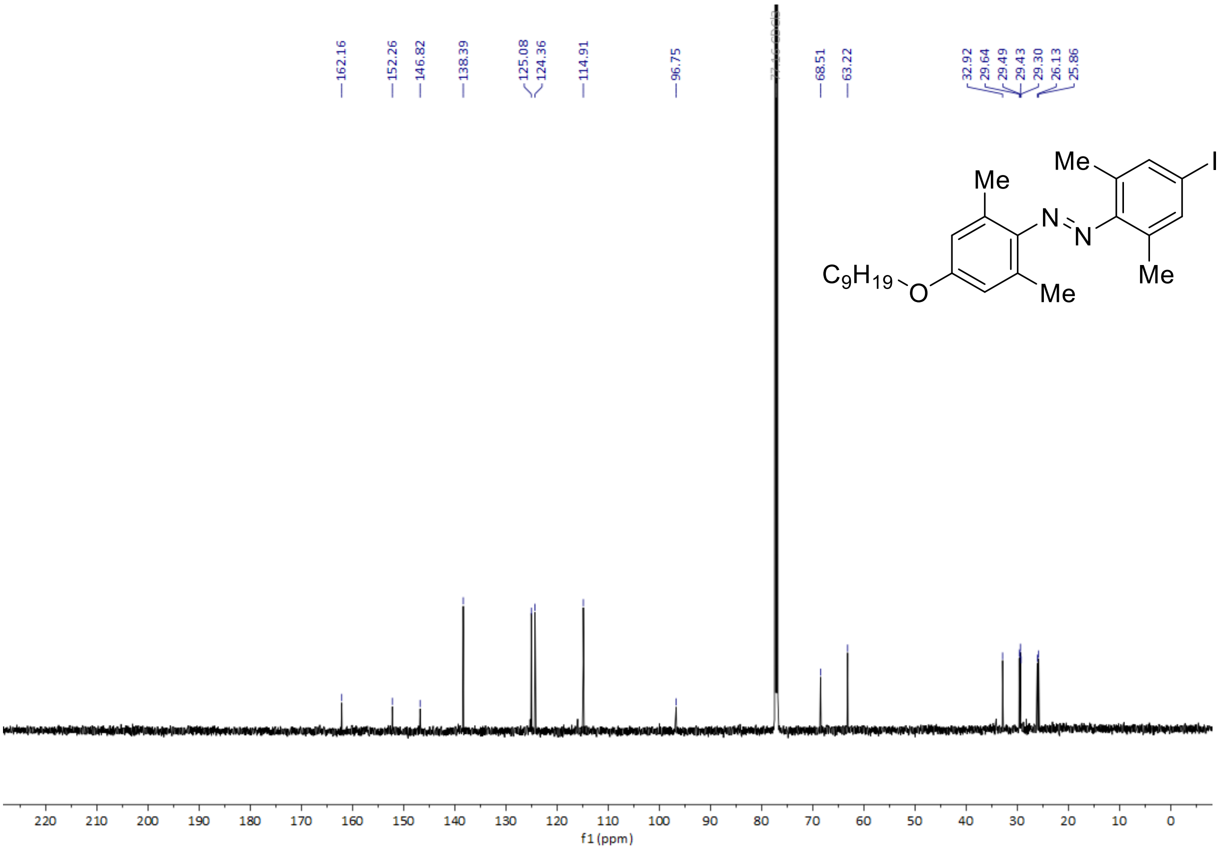

Figure S67: $^{13}C\{^1H\}$ NMR spectrum of **1j** in $CDCl_3$.

(*E*)-1-((4-Iodo-2,6-dimethylphenyl)diazenyl)naphthalene-2,7-diol (**S2**)

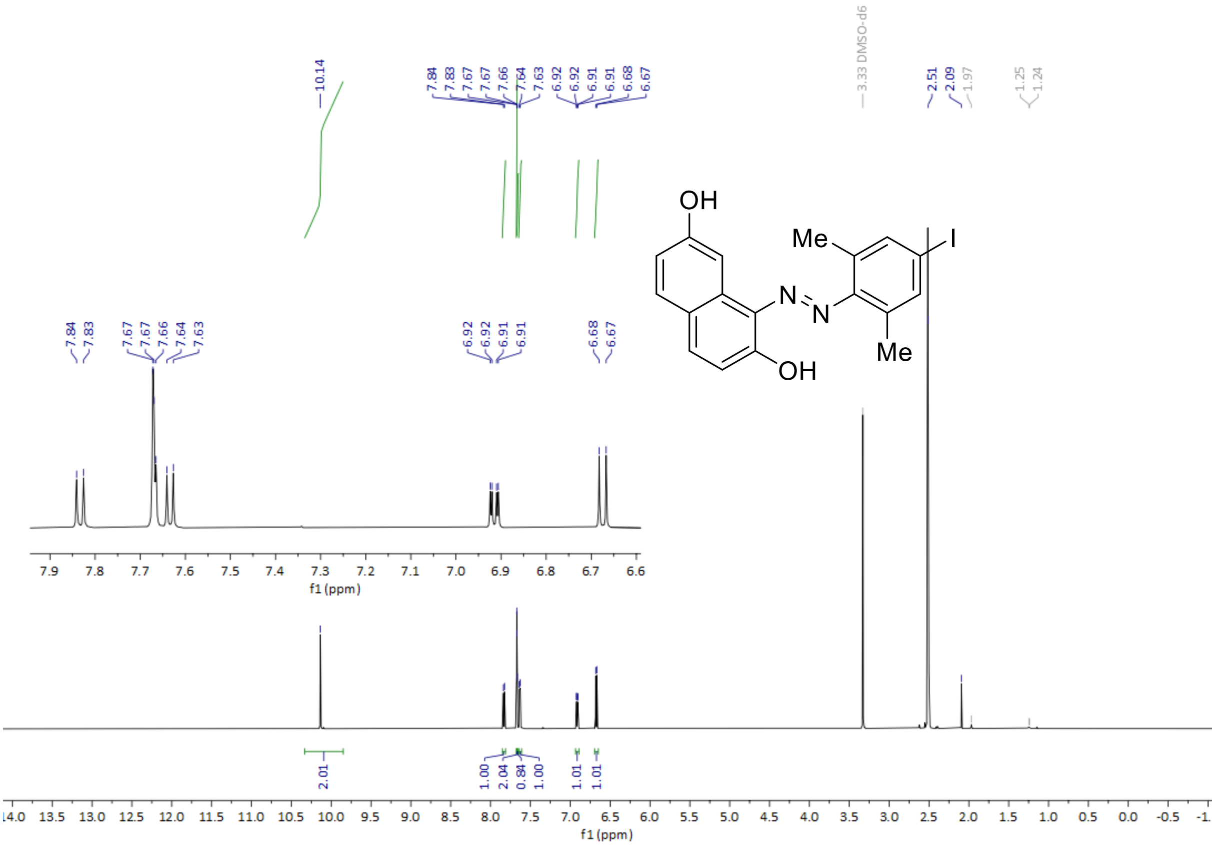

Figure S68: ^{1}H NMR spectrum of **S2** in DMSO-d_6.

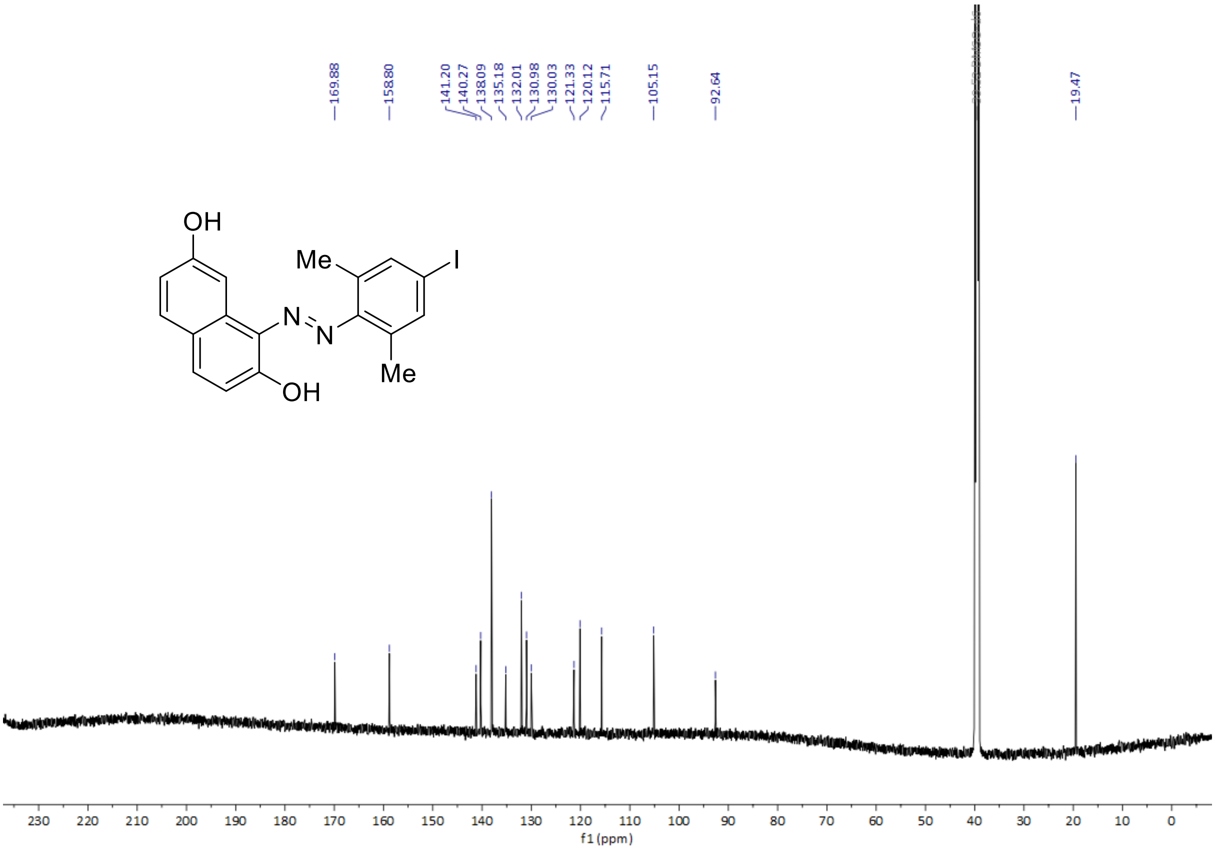

Figure S69: $^{13}C\{^{1}H\}$ NMR spectrum of **S2** in DMSO-d_6.

(*E*)-1-((4-Iodo-2,6-dimethylphenyl)diazenyl)naphthalene-2,7-diyl bis(4-(hexyloxy)benzoate) (**1k**)

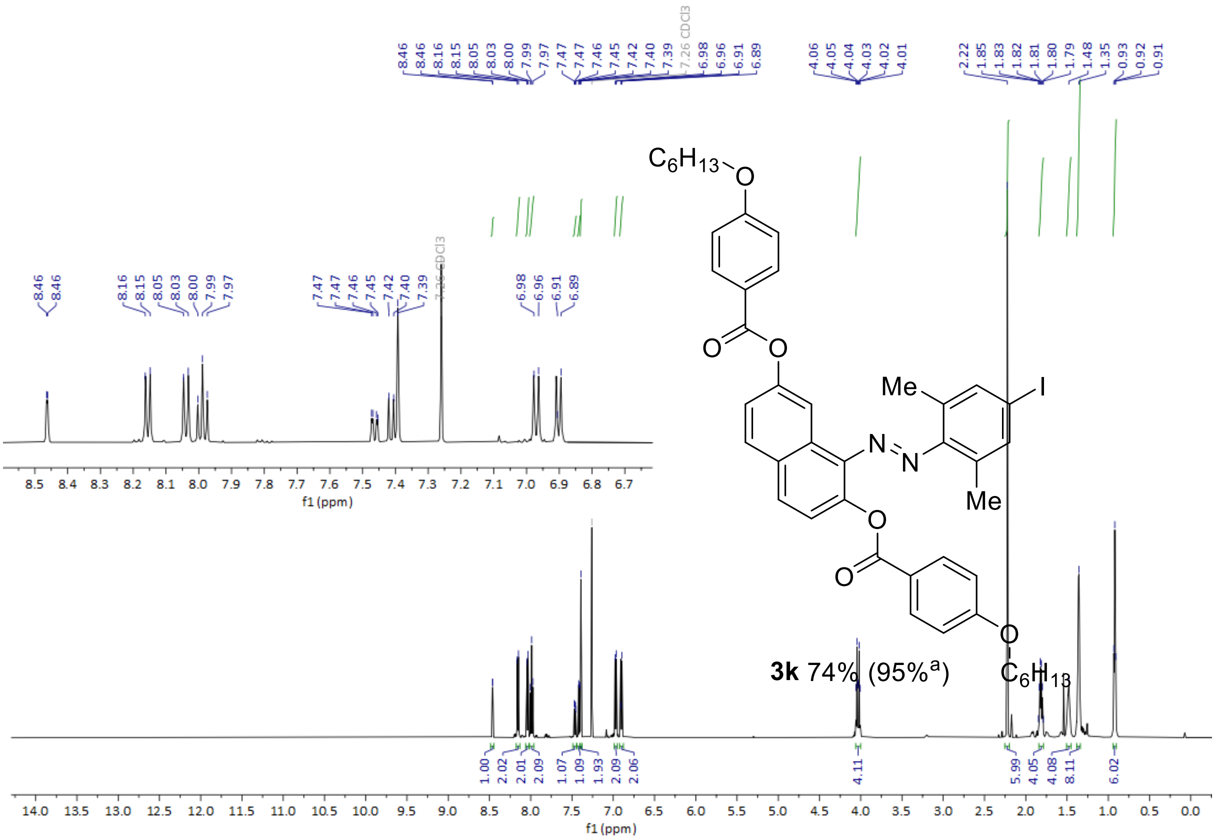

Figure S70: ^{1}H NMR spectrum of **1k** in $CDCl_3$.

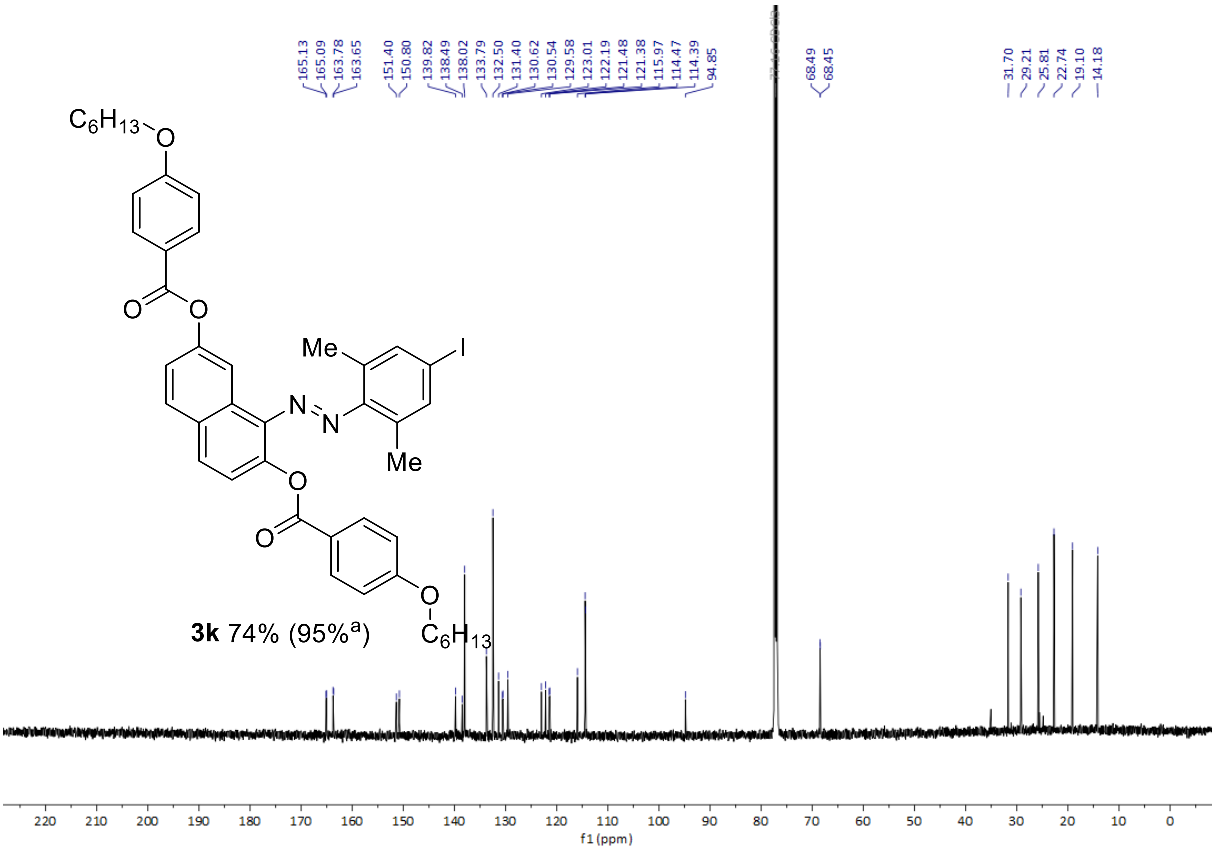

Figure S71: $^{13}C\{^1H\}$ NMR spectrum of **1k** in $CDCl_3$.

(*E*)-1-(4-Bromophenyl)-2-(4-iodophenyl)diazene (**1l**)

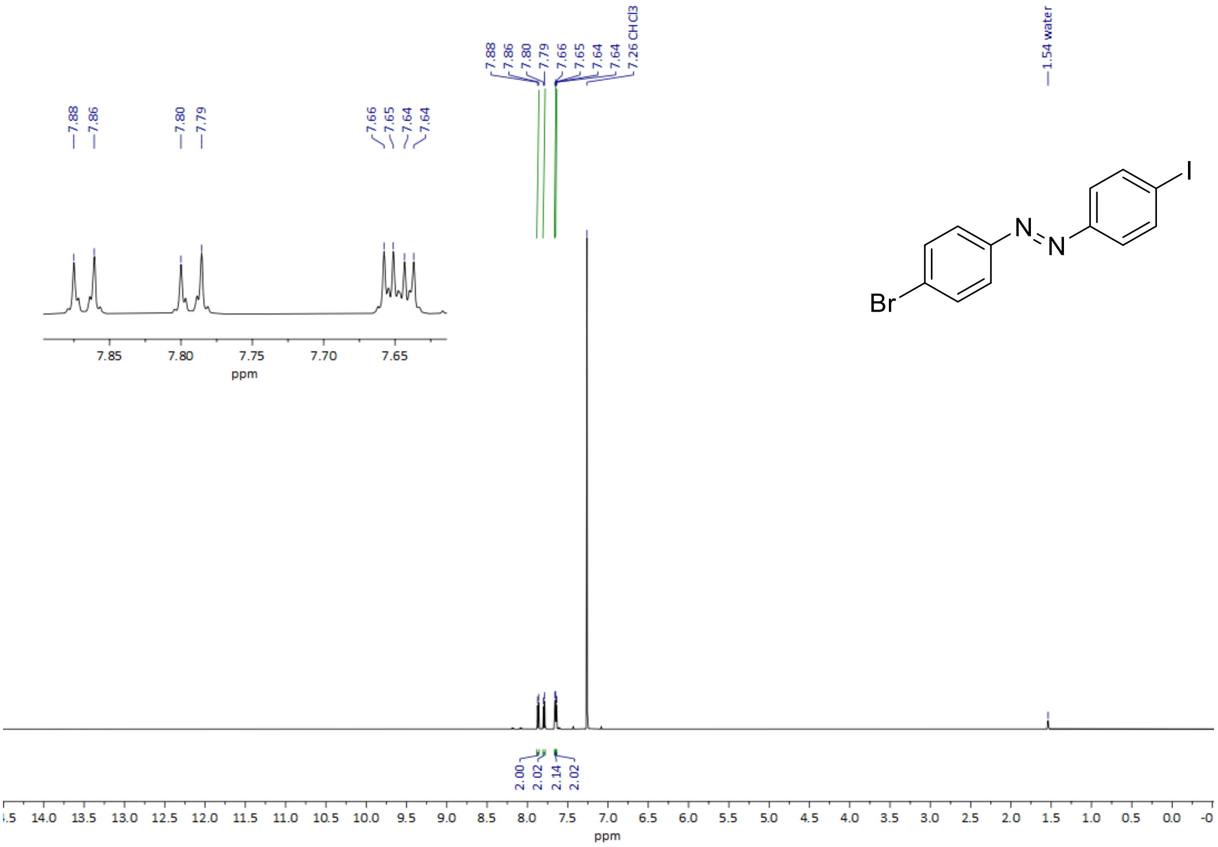

Figure S72: ^{1}H NMR spectrum of **1l** in $CDCl_3$.

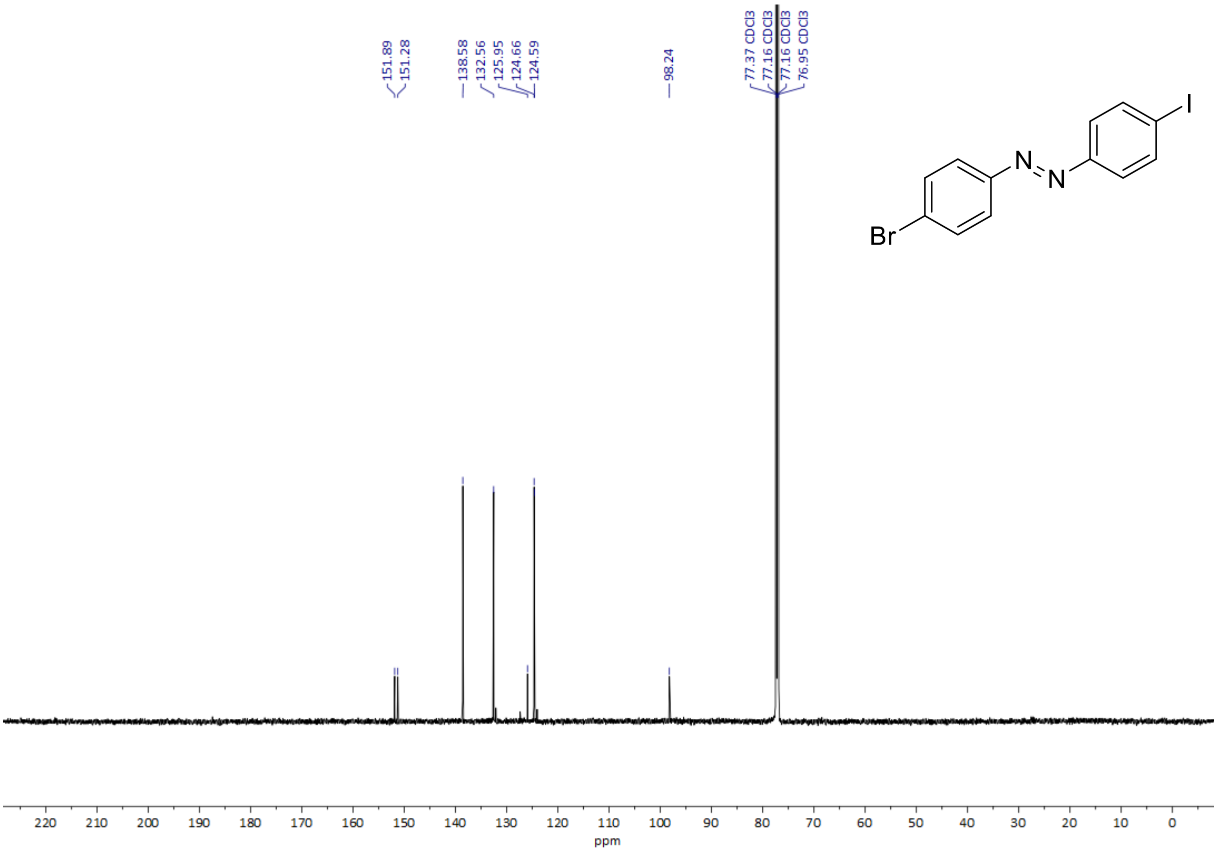

Figure S73: $^{13}C\{^{1}H\}$ NMR spectrum of **1l** in $CDCl_3$.

(*E*)-1-(3-Bromophenyl)-2-(4-iodophenyl)diazene (**1m**)

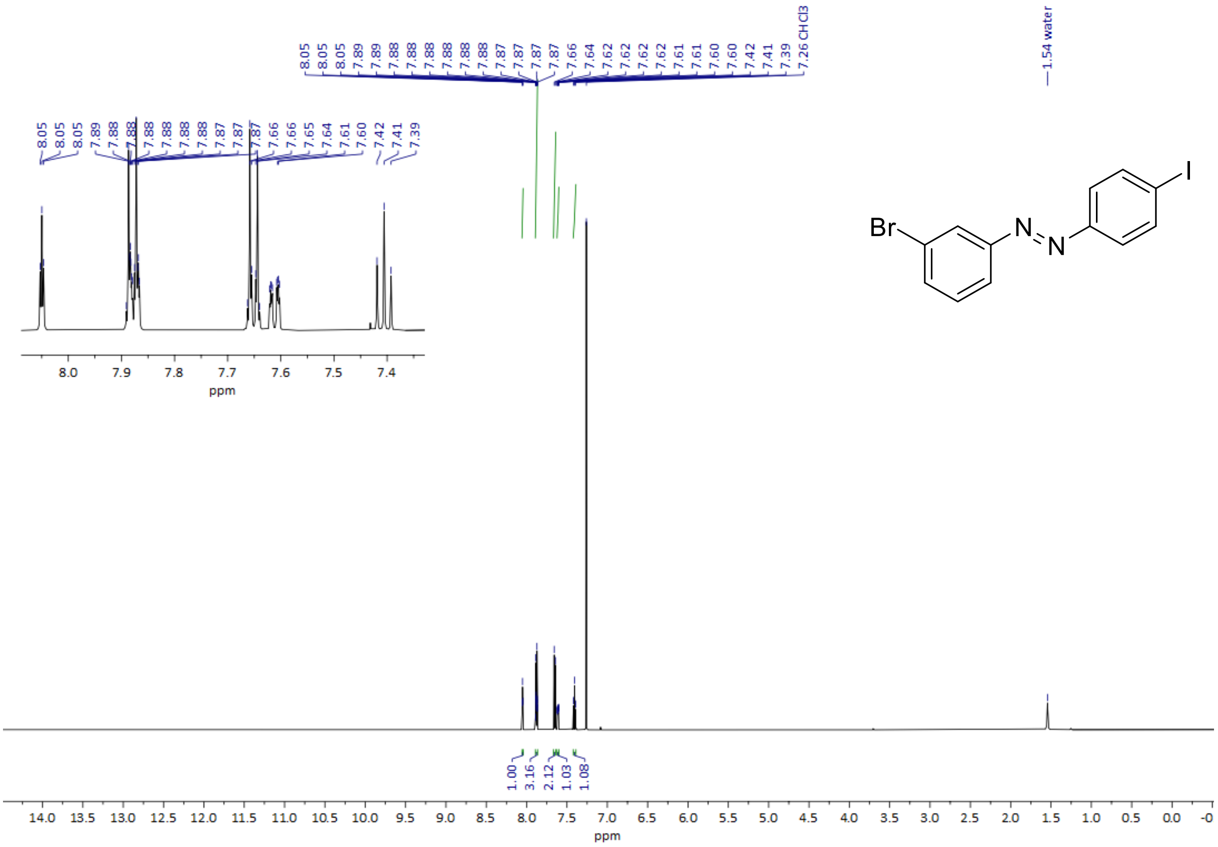

Figure S74: ^{1}H NMR spectrum of **1m** in $CDCl_3$.

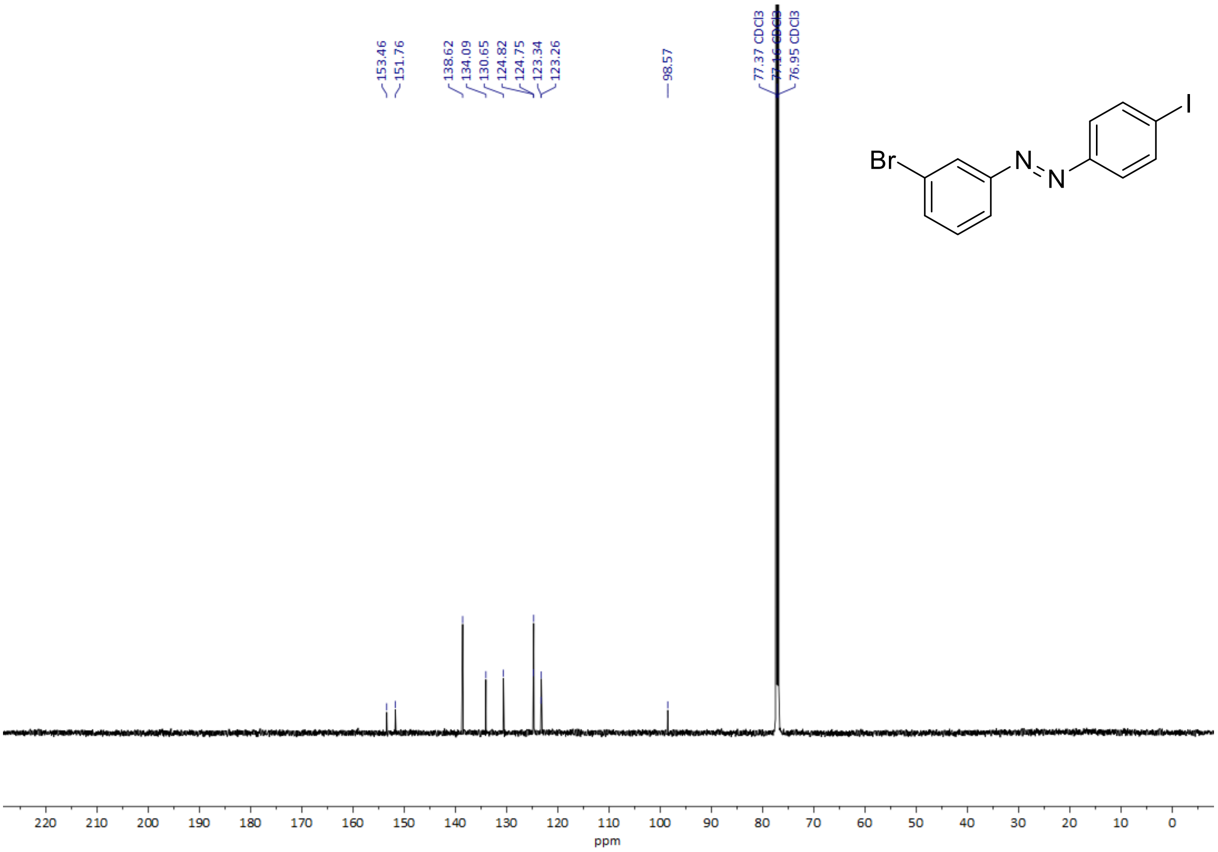

Figure S75: $^{13}C\{^{1}H\}$ NMR spectrum of **1m** in $CDCl_3$.

(*E*)-1-(2-Bromophenyl)-2-(4-iodophenyl)diazene (**1n**)

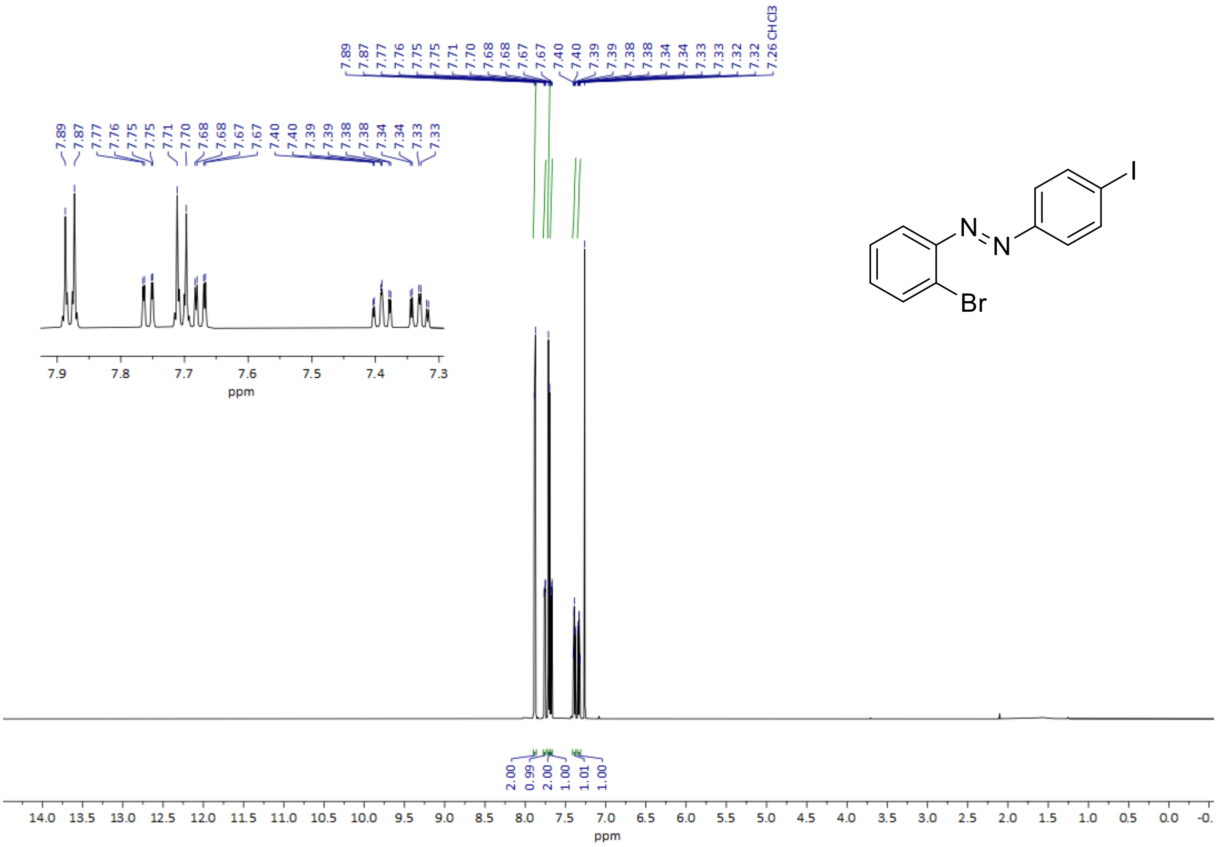

Figure S76: ^{1}H NMR spectrum of **1n** in $CDCl_3$.

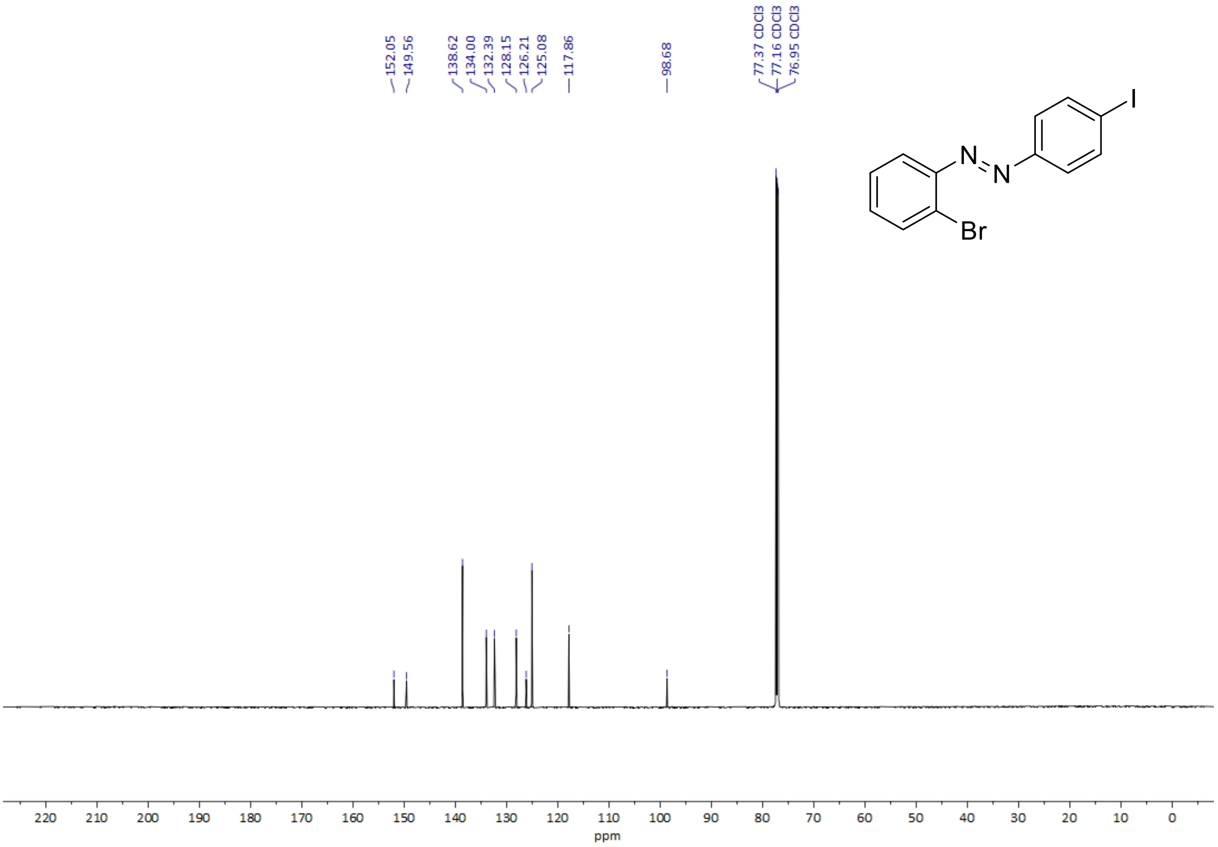

Figure S77: $^{13}C\{^{1}H\}$ NMR spectrum of **1n** in $CDCl_3$.

(*E*)-1,2-Bis(4-iodophenyl)diazene (**4a**)

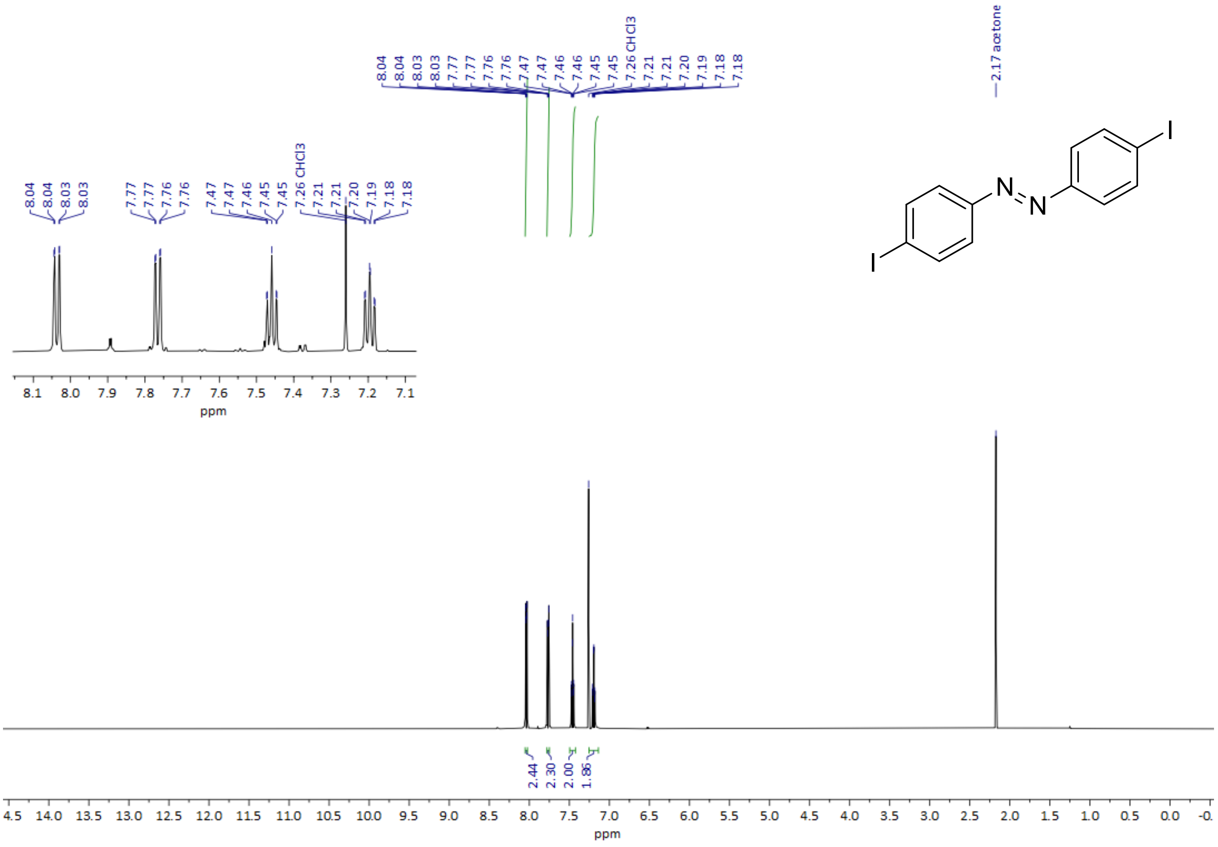

Figure S78: ^{1}H NMR spectrum of **4a** in $CDCl_3$.

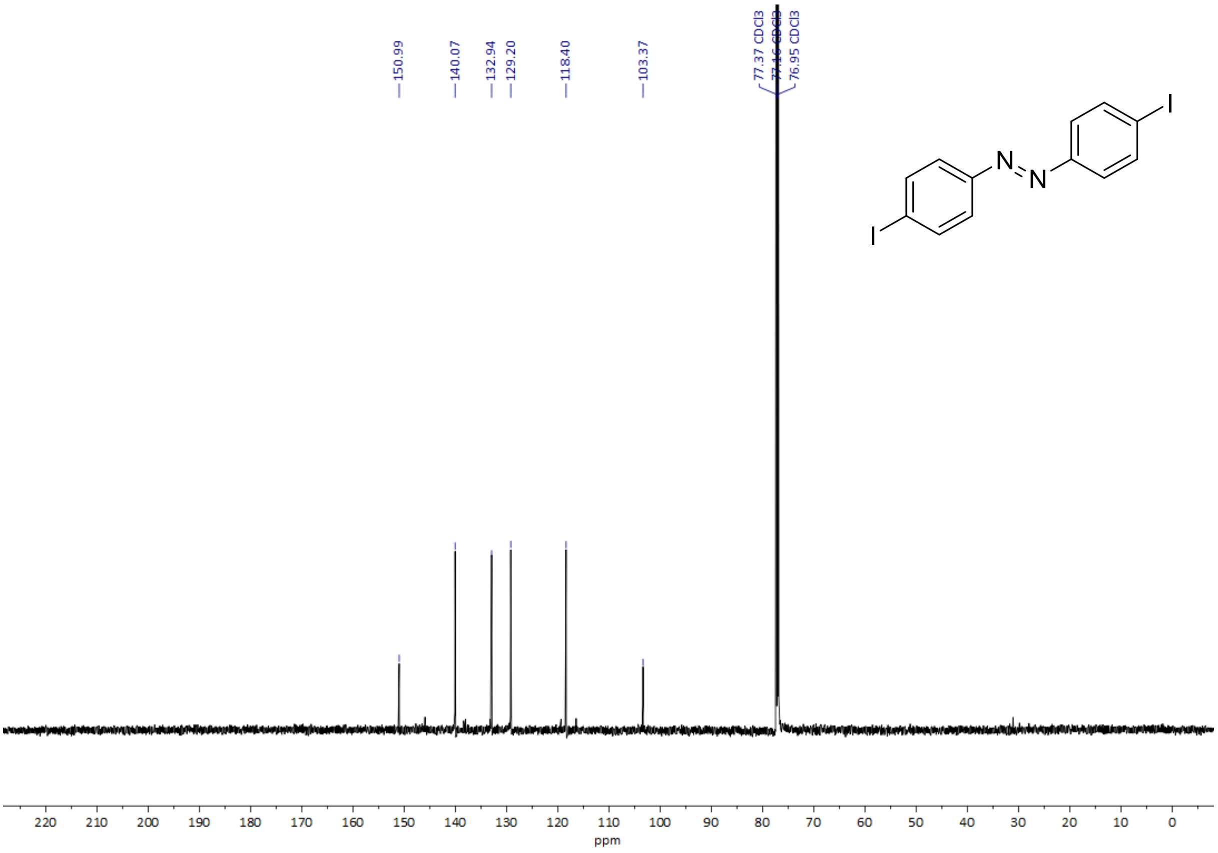

Figure S79: $^{13}C\{^{1}H\}$ NMR spectrum of **4a** in $CDCl_3$.

4-Iodo-2,6-dimethylaniline (**S3**)

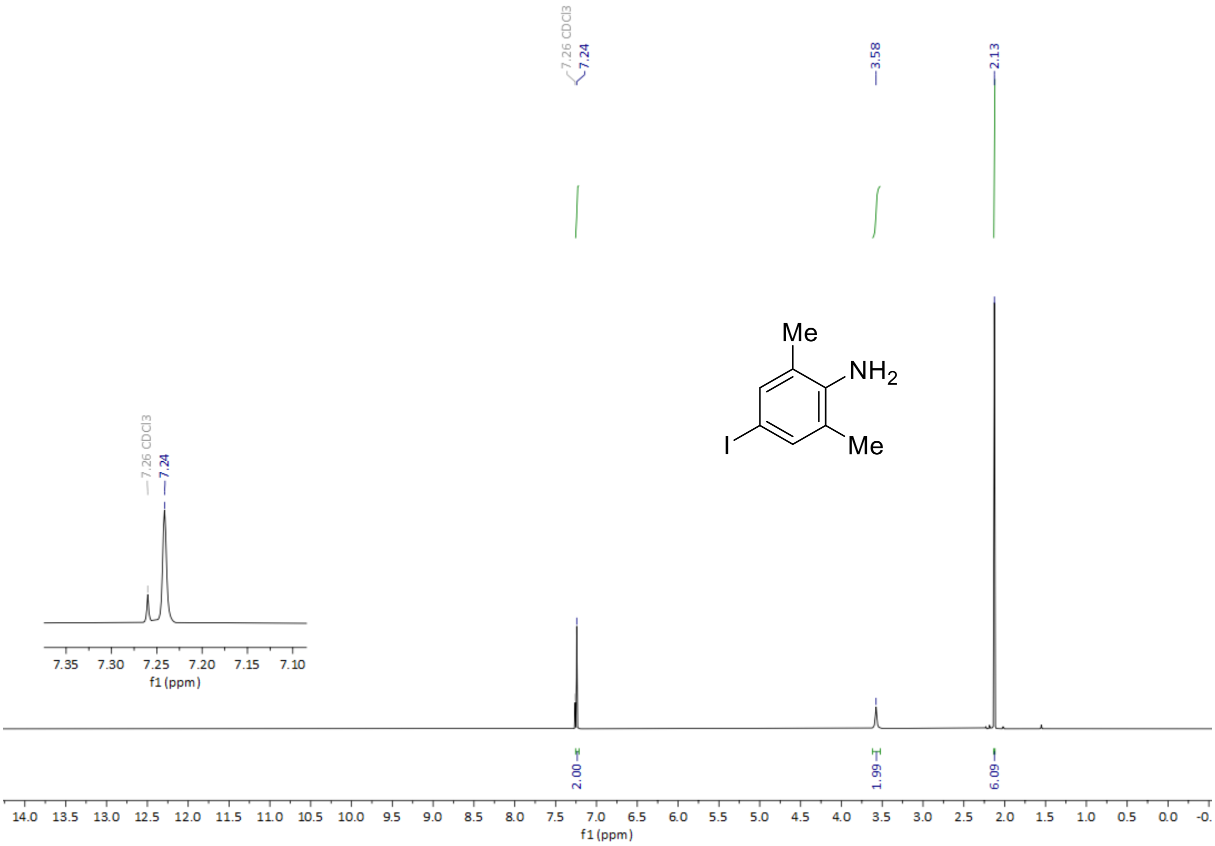

Figure S80:^{1}H NMR spectrum of **S3** in $CDCl_3$.

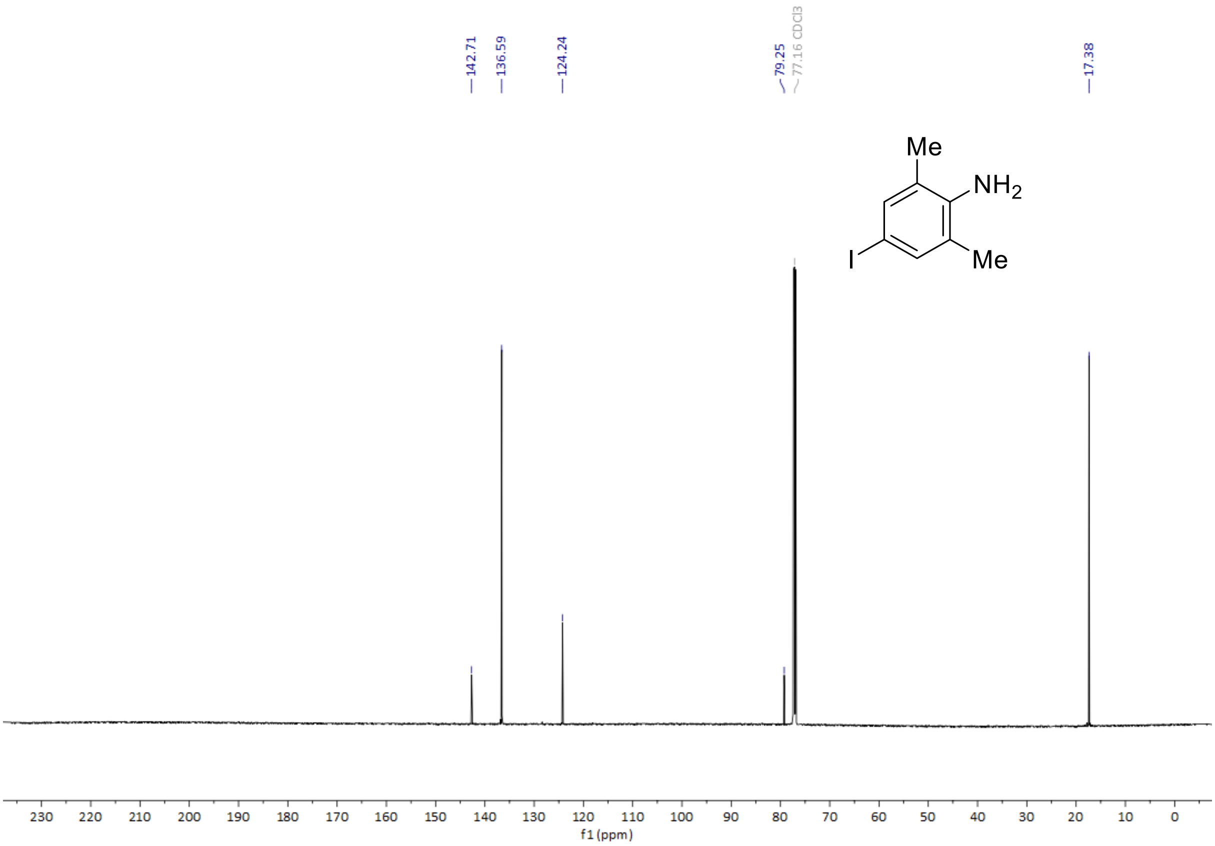

Figure S81: $^{13}C\{^{1}H\}$ NMR spectrum of **S3** in $CDCl_3$.

(*E*)-1,2-Bis(4-iodo-2,6-dimethylphenyl)diazene (**4b**)

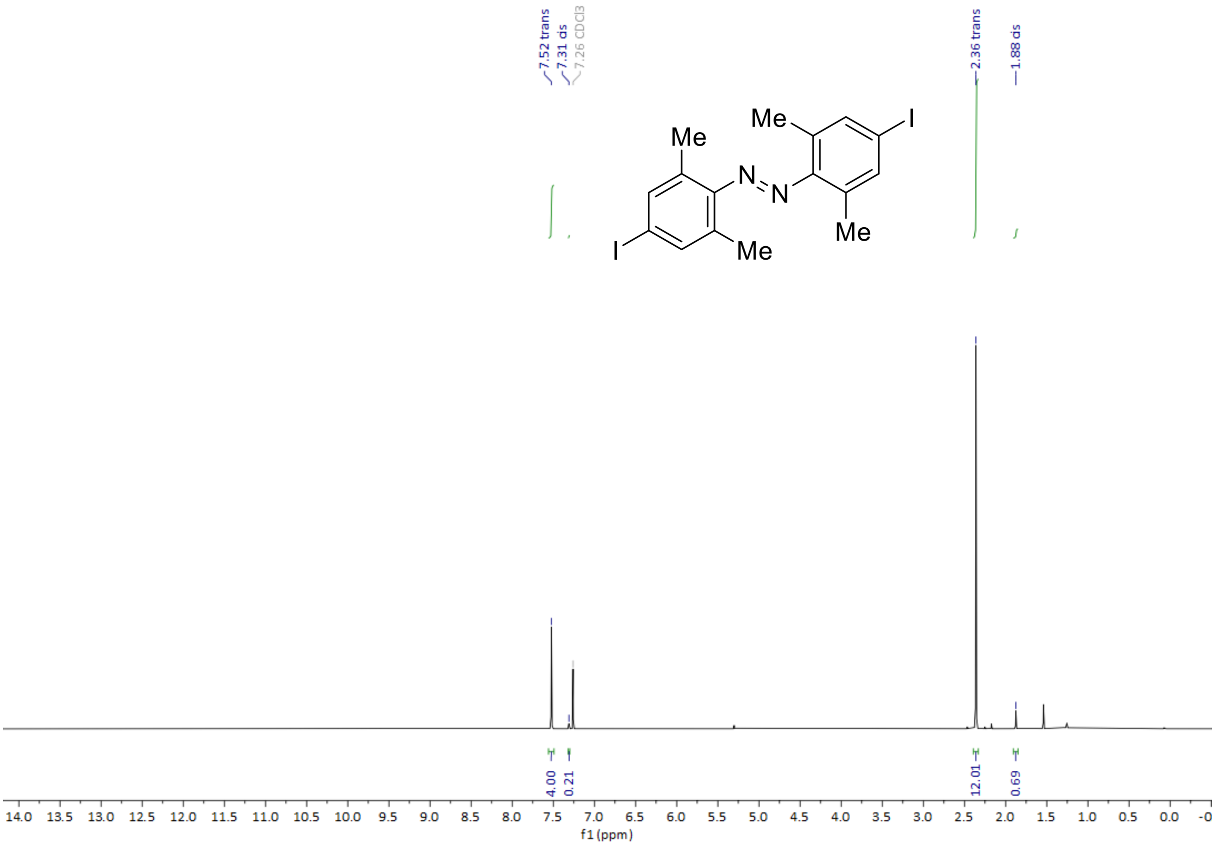

Figure S82: ^{1}H NMR spectrum of **4b** in $CDCl_3$.

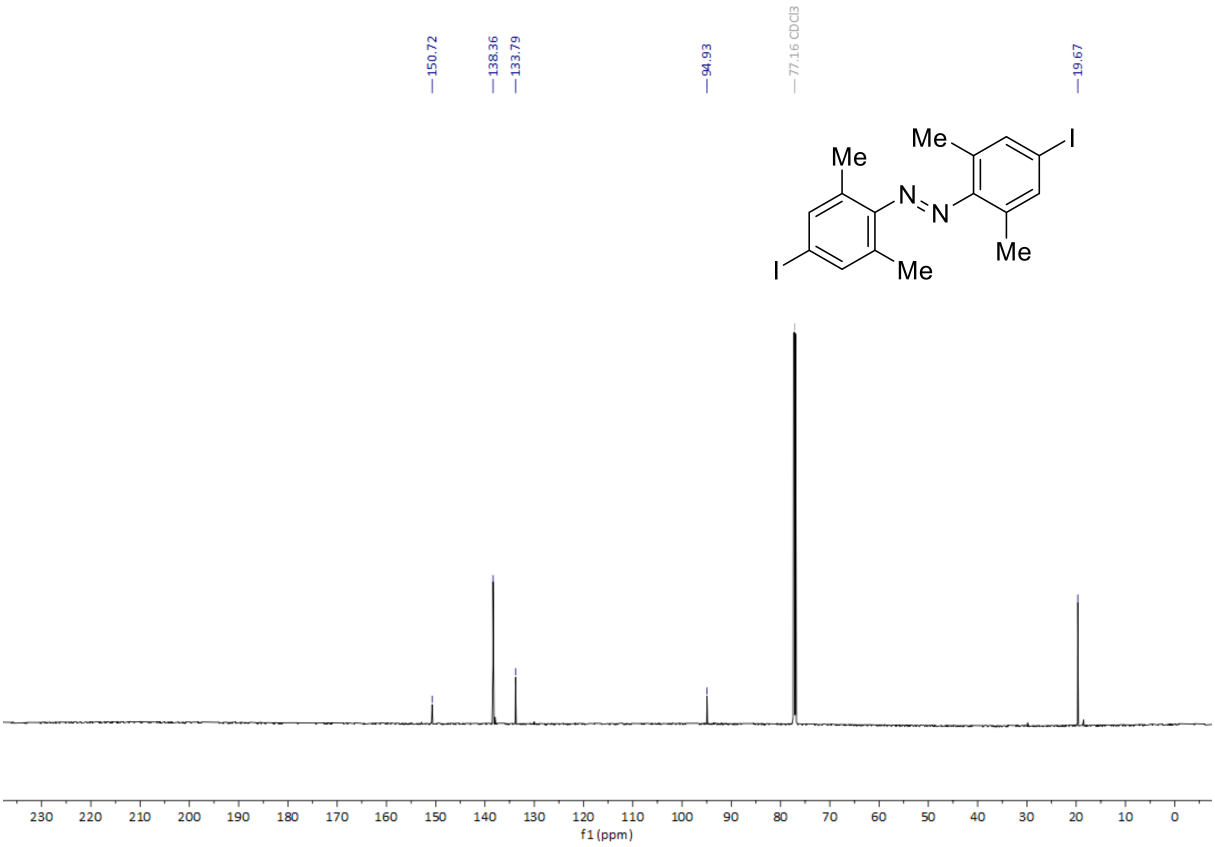

Figure S83: $^{13}C\{^{1}H\}$ NMR spectrum of **4b** in $CDCl_3$.

4-Bromo-2,6-dimethoxyaniline (**S4**)

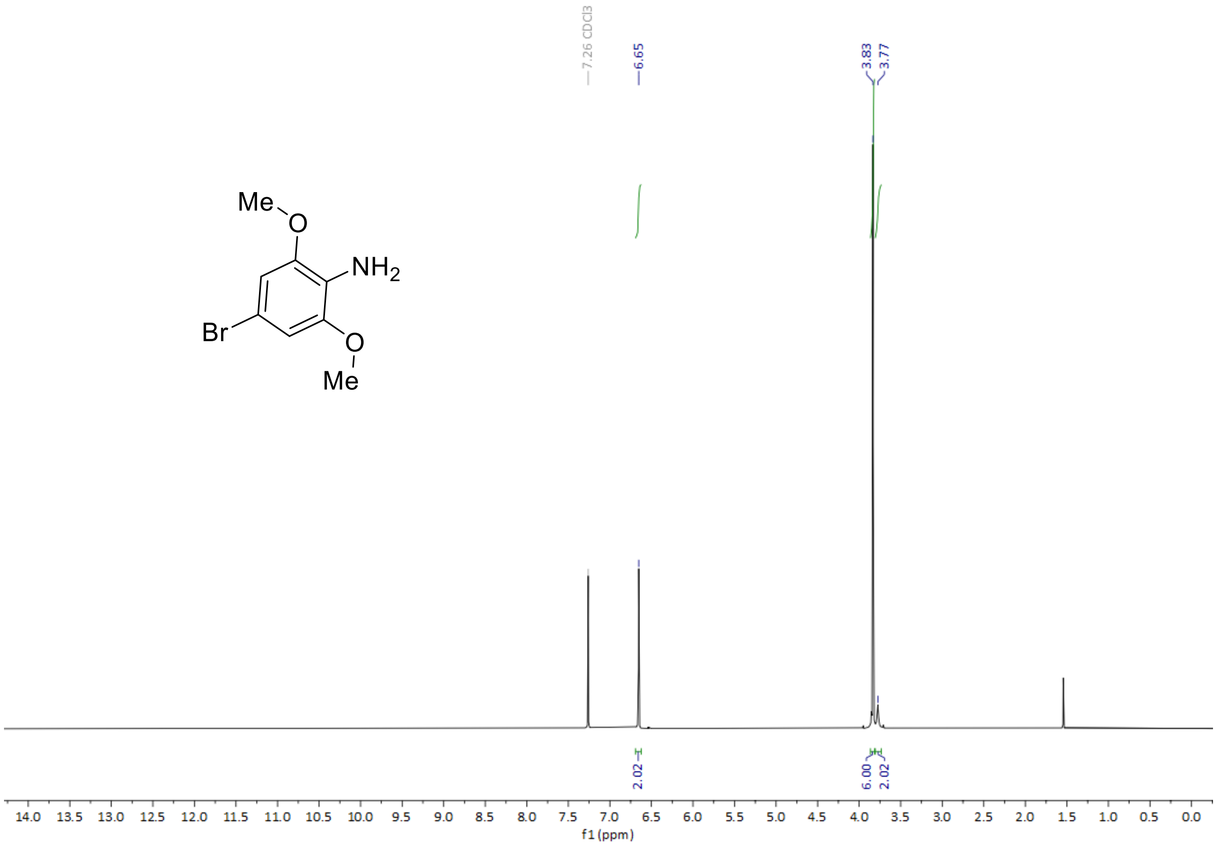

Figure S84:^{1}H NMR spectrum of **S4** in $CDCl_3$.

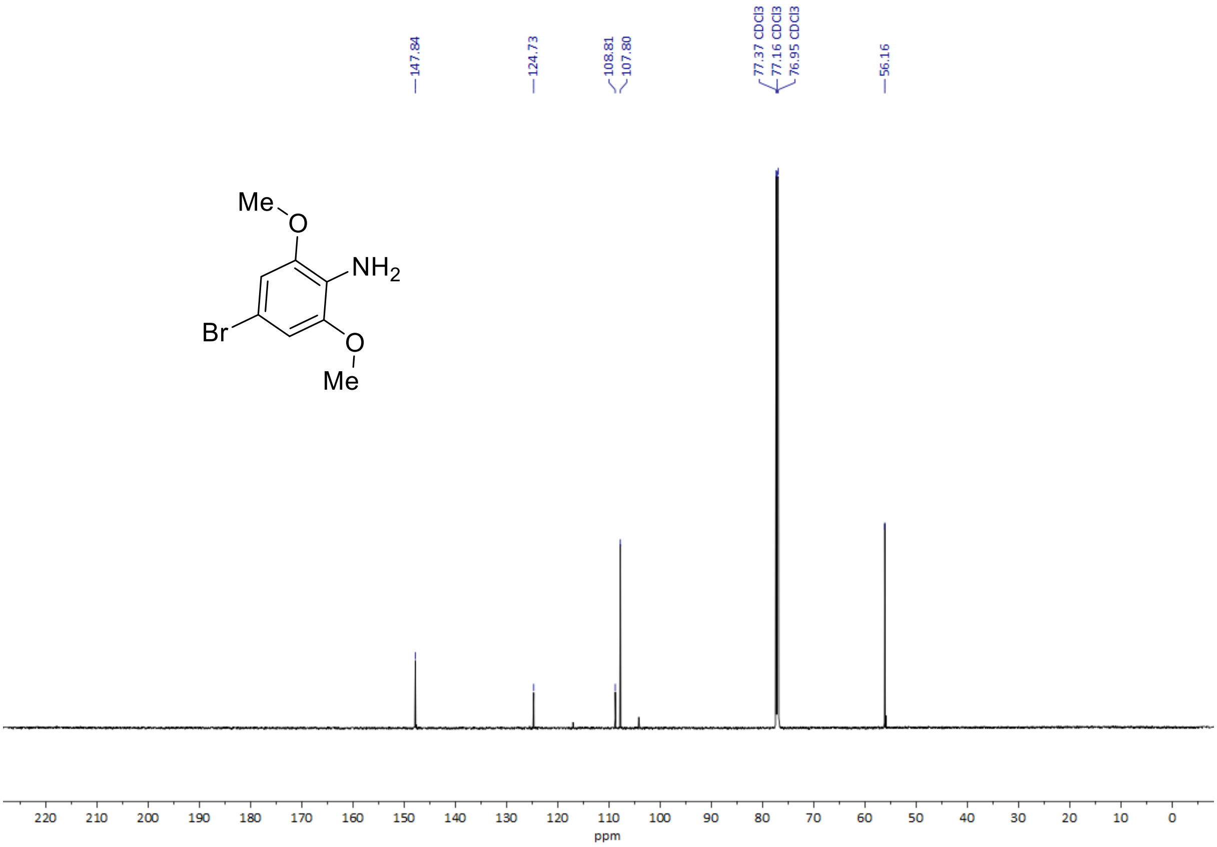

Figure S85:^{13}C NMR spectrum of **S4** in $CDCl_3$.

(*E*)-1,2-Bis(4-bromo-2,6-dimethoxyphenyl)diazene (**S5**)

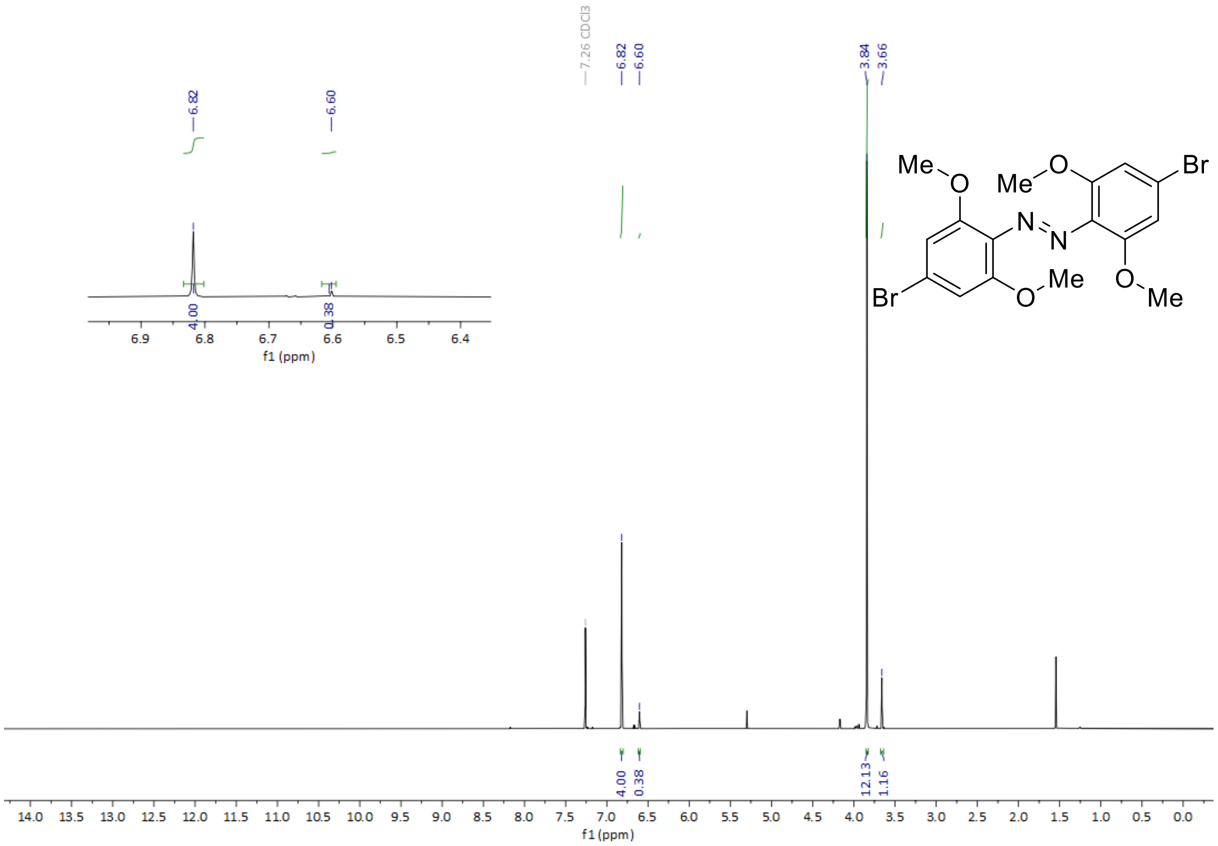

Figure S86: ^{1}H NMR spectrum of **S5** in $CDCl_3$.

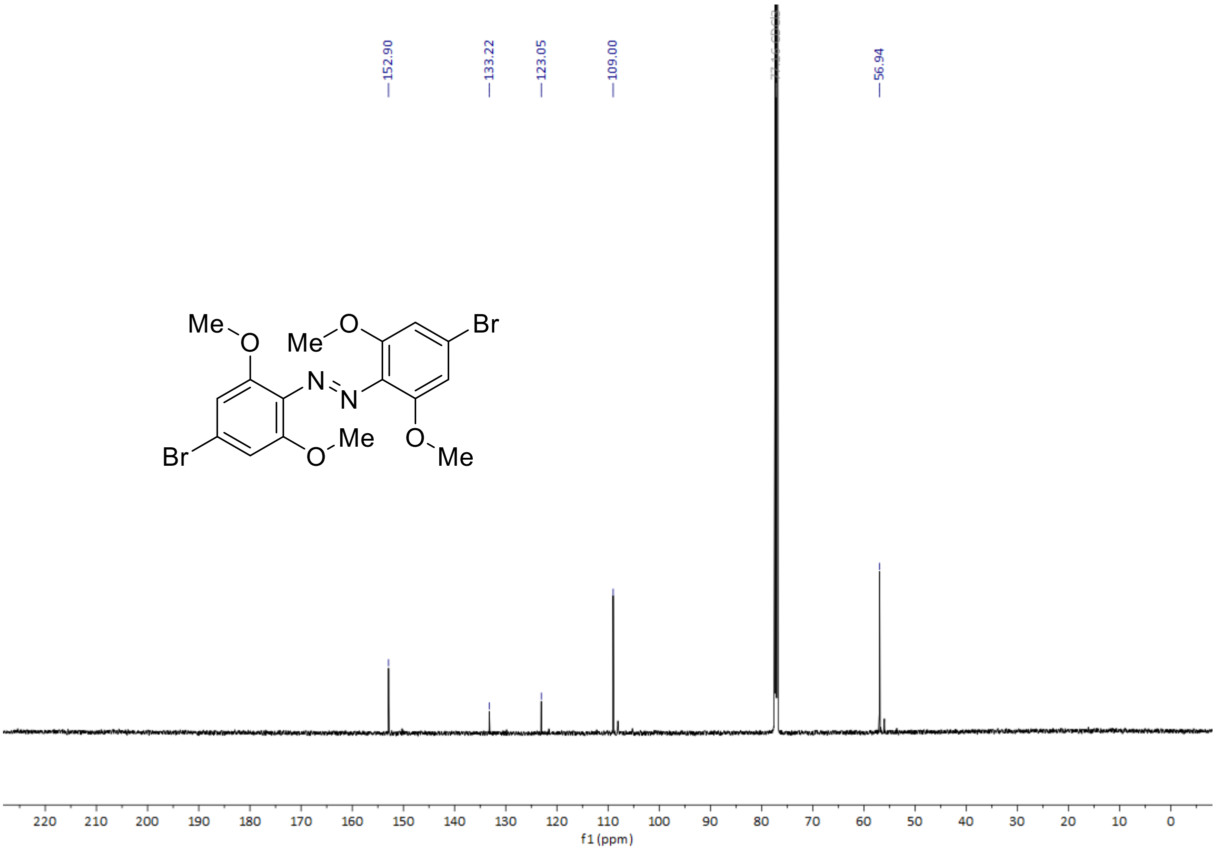

Figure S87: $^{13}C\{^{1}H\}$ NMR spectrum of **S5** in $CDCl_3$.

(*E*)-1,2-Bis(4-iodo-2,6-dimethoxyphenyl)diazene (**4c**)

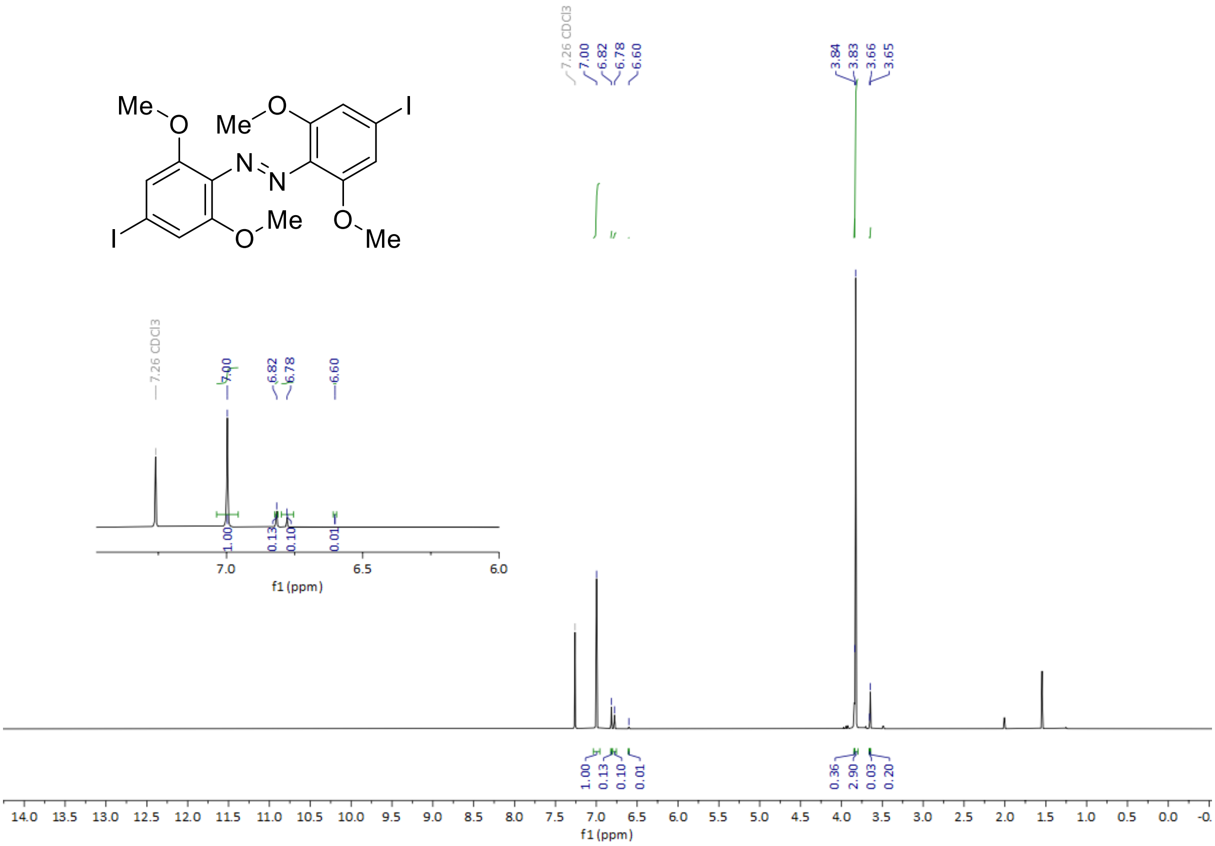

Figure S88: ^{1}H NMR spectrum of **4c** in $CDCl_3$.

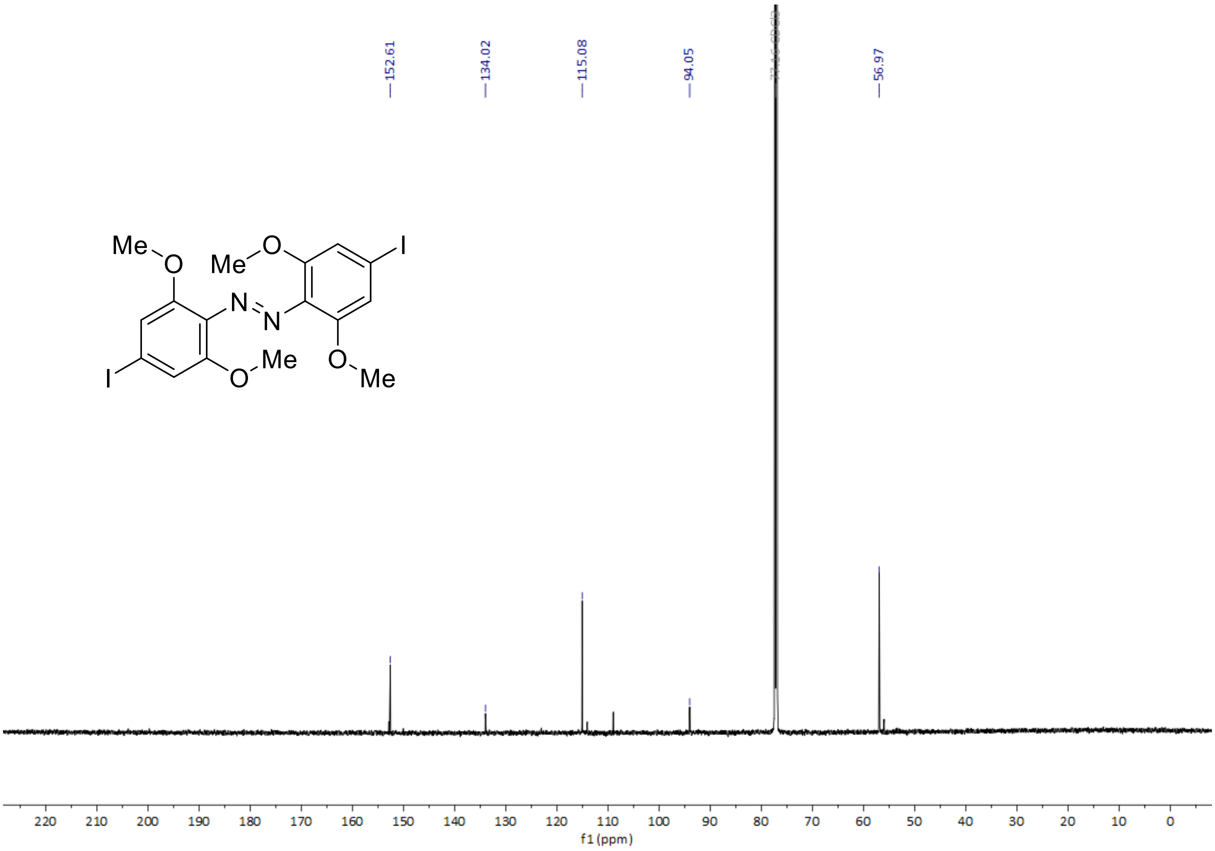

Figure S89: $^{13}C\{^{1}H\}$ NMR spectrum of **4c** in $CDCl_3$.

4-Iodo-2,6-difluoro-aniline (**S6**)

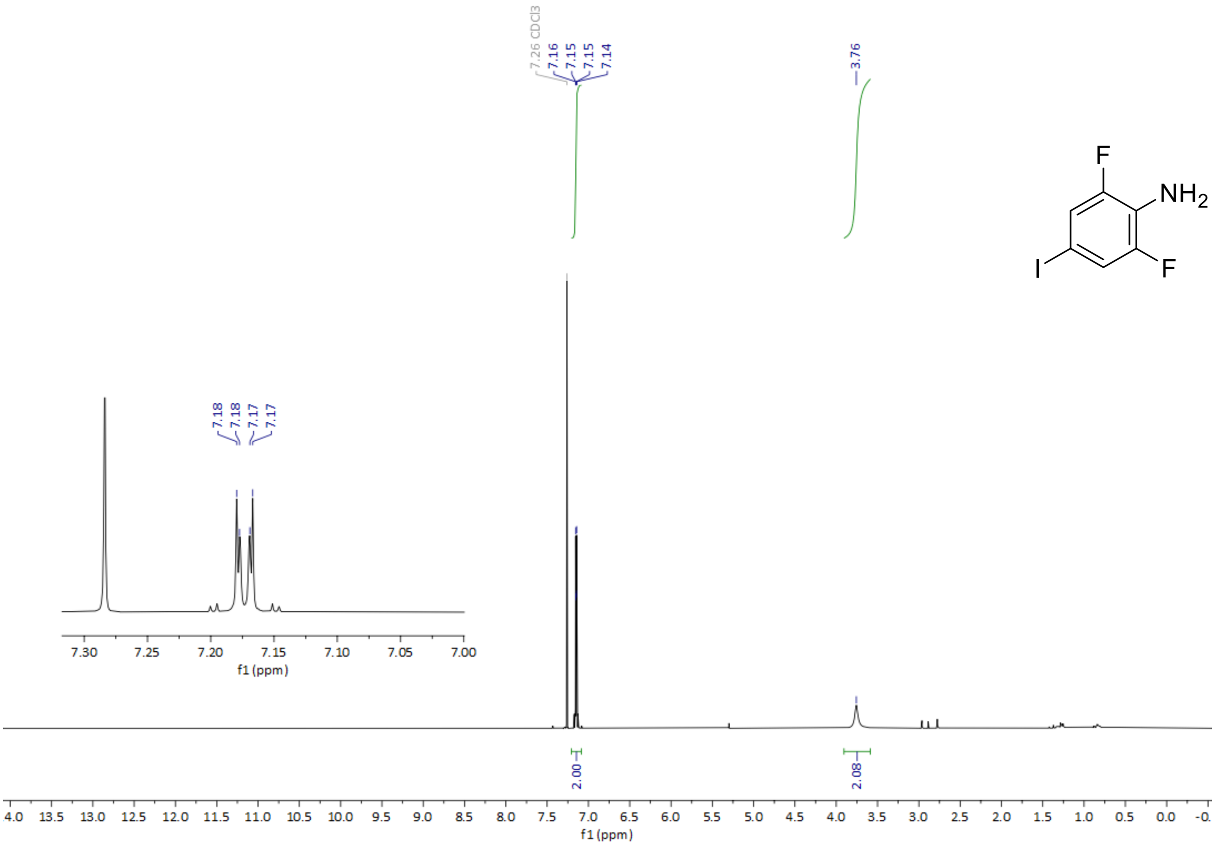

Figure S90: ^{1}H NMR spectrum of **S6** in $CDCl_3$.

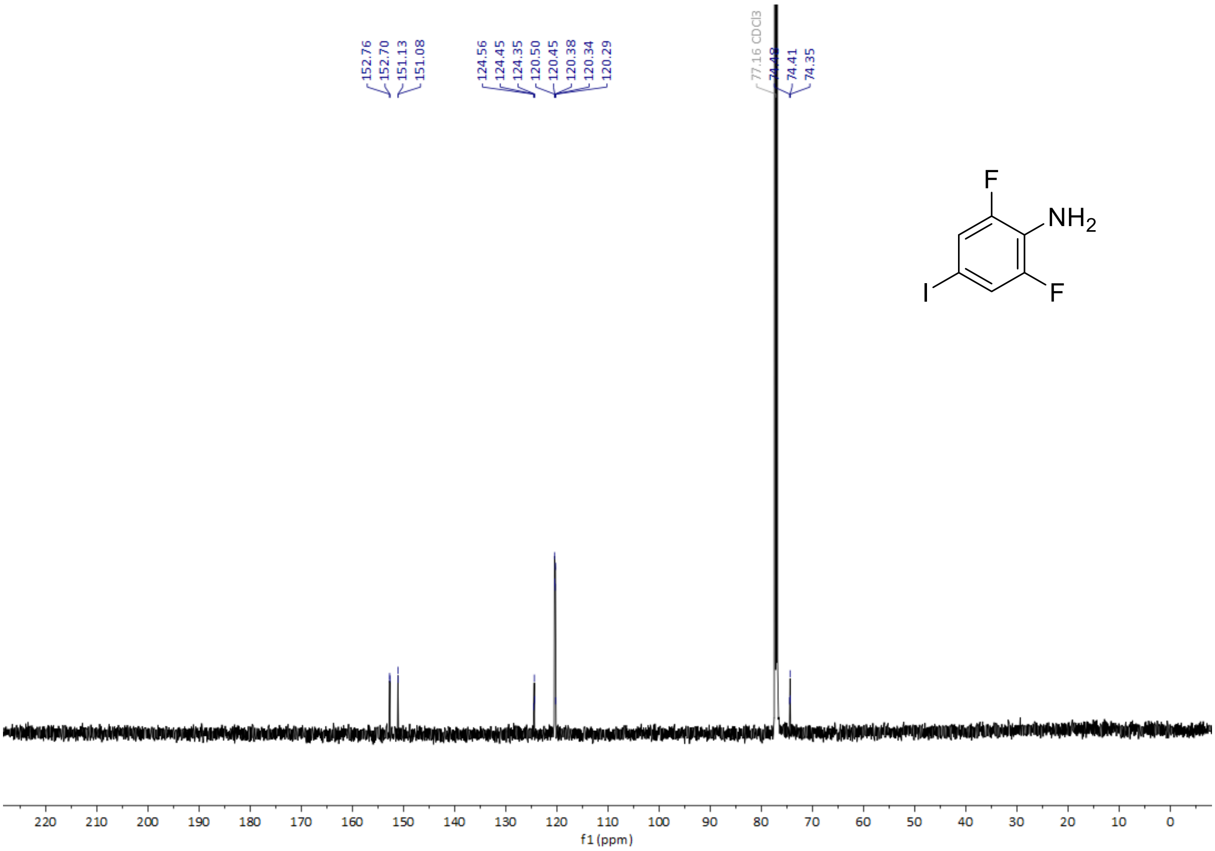

Figure S91: $^{13}C\{^{1}H\}$ NMR spectrum of **S6** in $CDCl_3$.

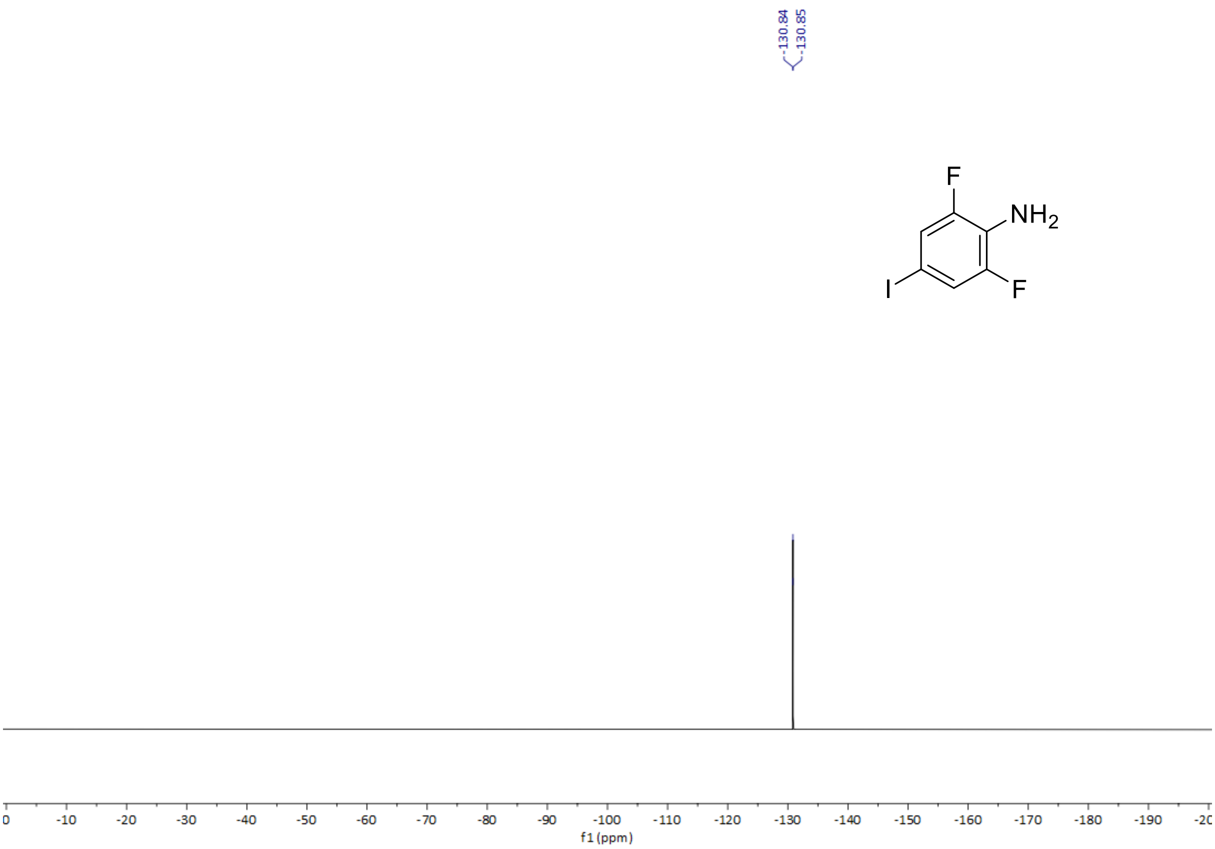

Figure S92: ^{19}F NMR spectrum of **S6** in $CDCl_3$.

(*E*)-1,2-bis(2,6-difluoro-4-iodophenyl)diazene (**4d**)

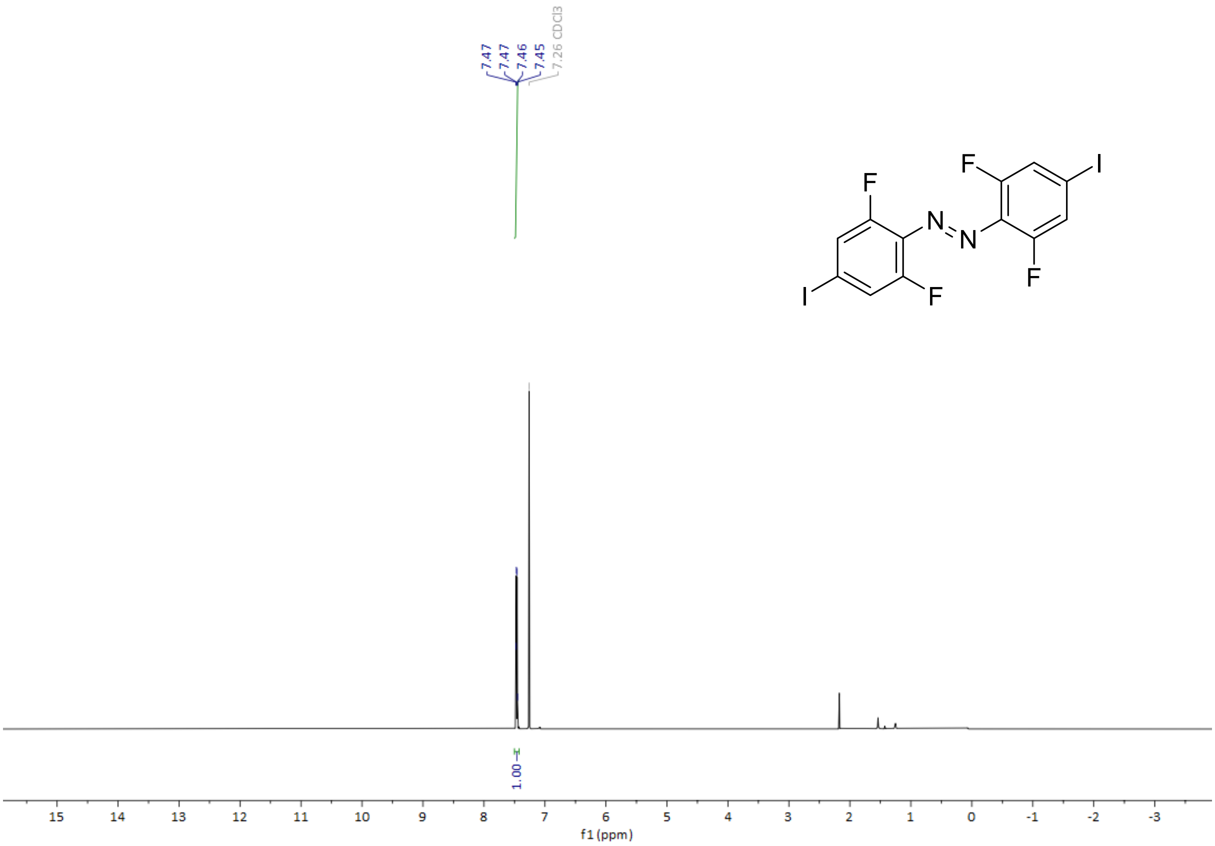

Figure S93: ^{1}H NMR spectrum of **4d** in $CDCl_3$.

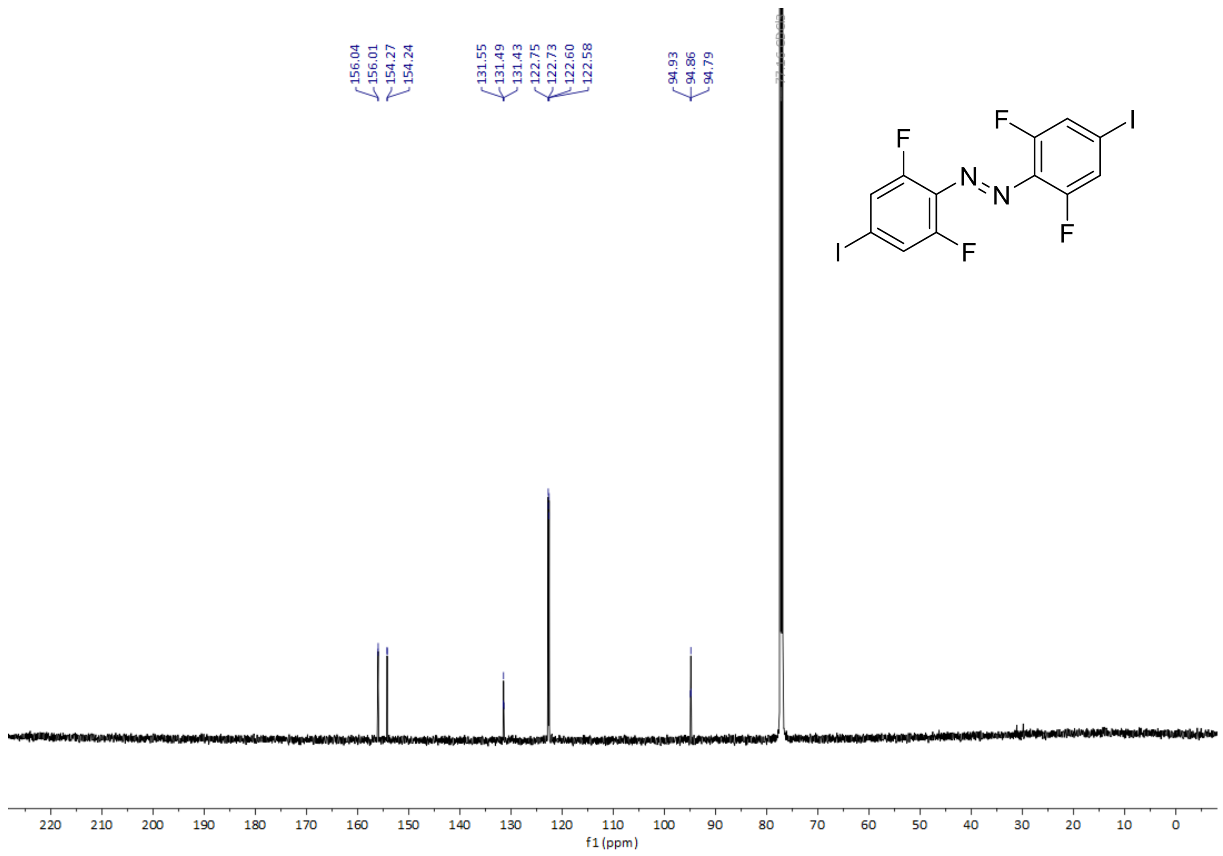

Figure S94: $^{13}C\{^{1}H\}$ NMR spectrum of **4d** in $CDCl_3$.

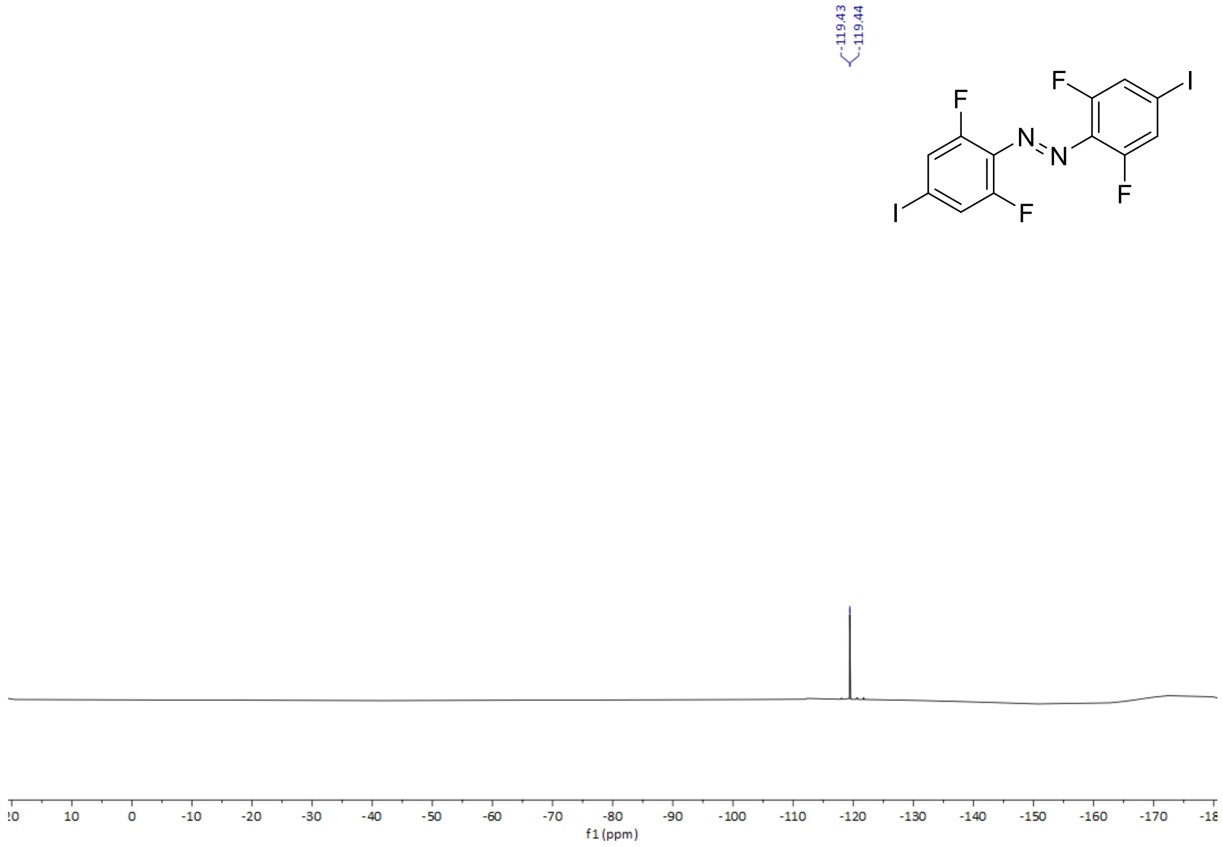

Figure S95: ^{19}F NMR spectrum of **4d** in $CDCl_3$.

4-Bromo-2,6-difluoroaniline (**S7**)

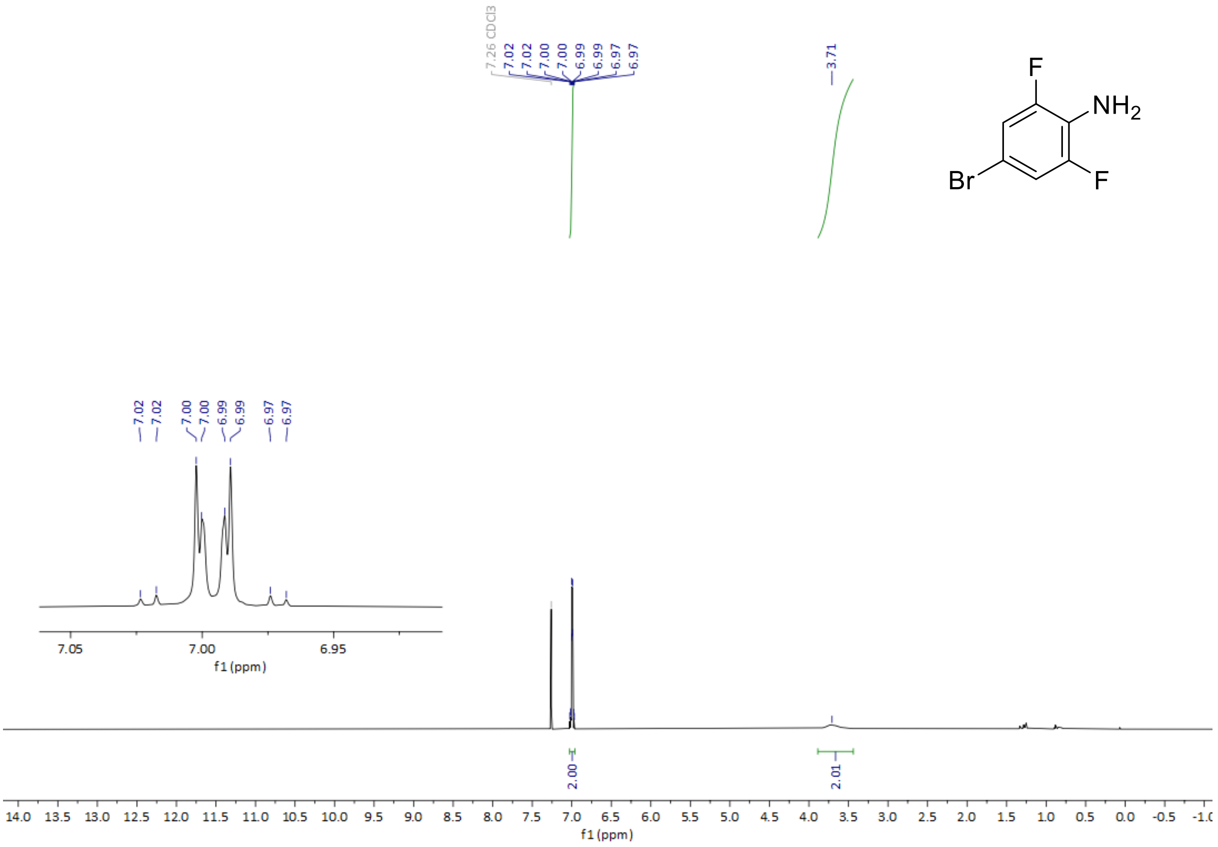

Figure S96:^{1}H NMR spectrum of **S7** in $CDCl_3$.

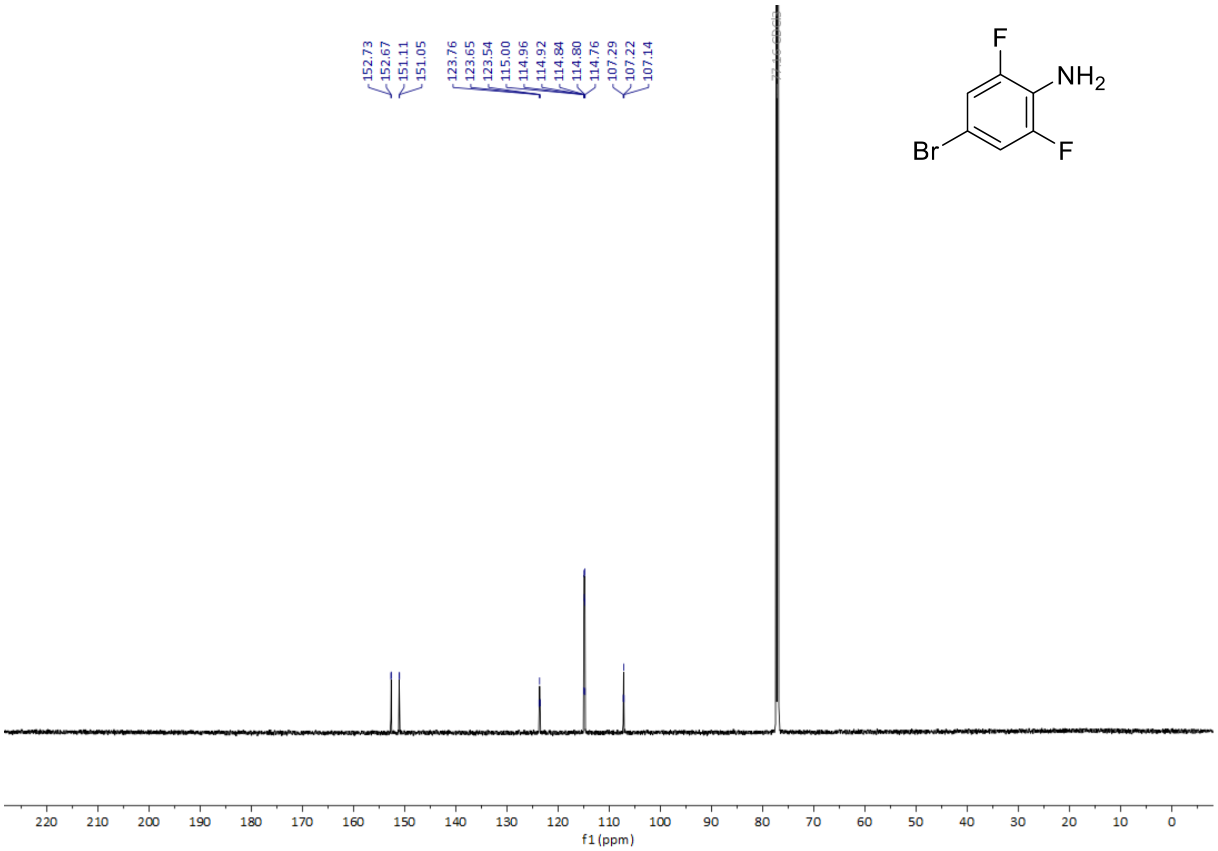

Figure S97:^{13}C NMR spectrum of **S7** in $CDCl_3$.

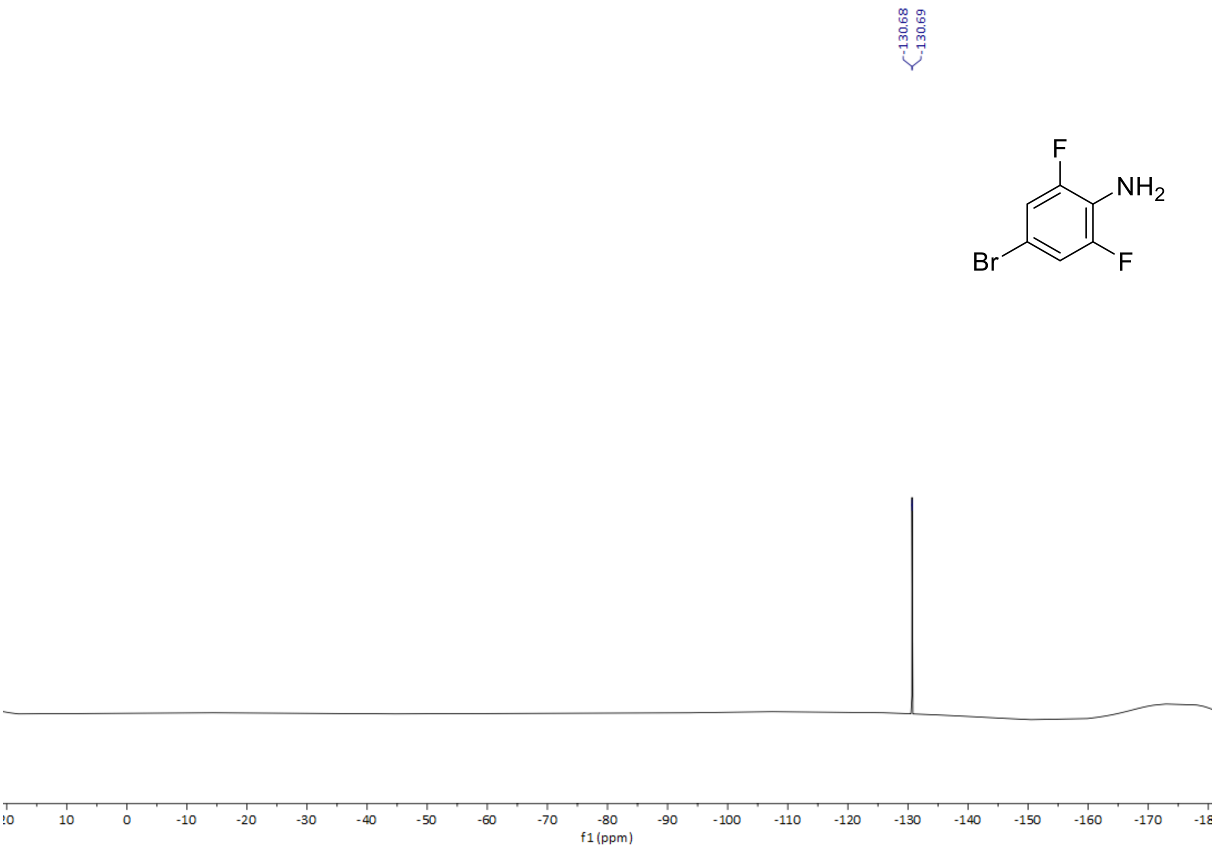

Figure S98: ^{19}F NMR spectrum of **S7** in $CDCl_3$.

4-Amino-3,5-difluorobenzonitrile (**S8**)

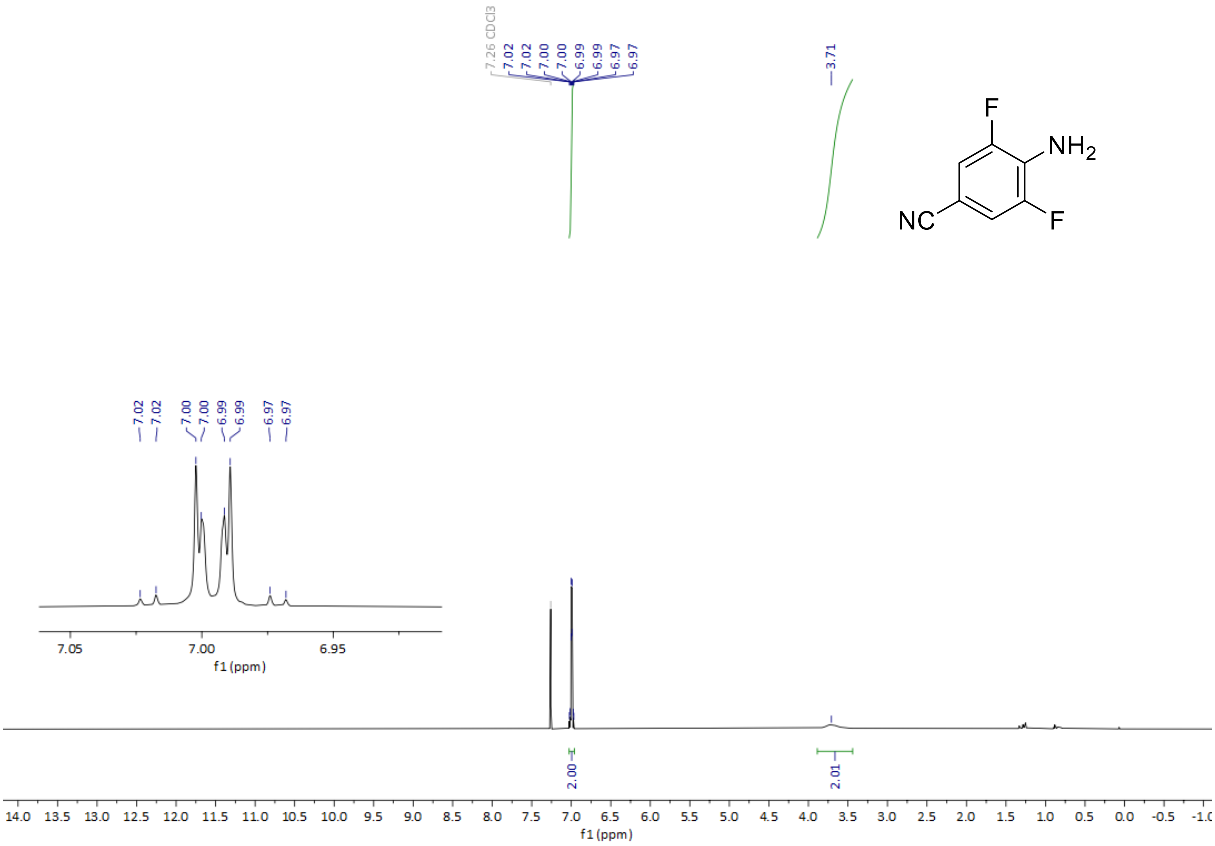

Figure S99:^{1}H NMR spectrum of **S8** in $CDCl_3$.

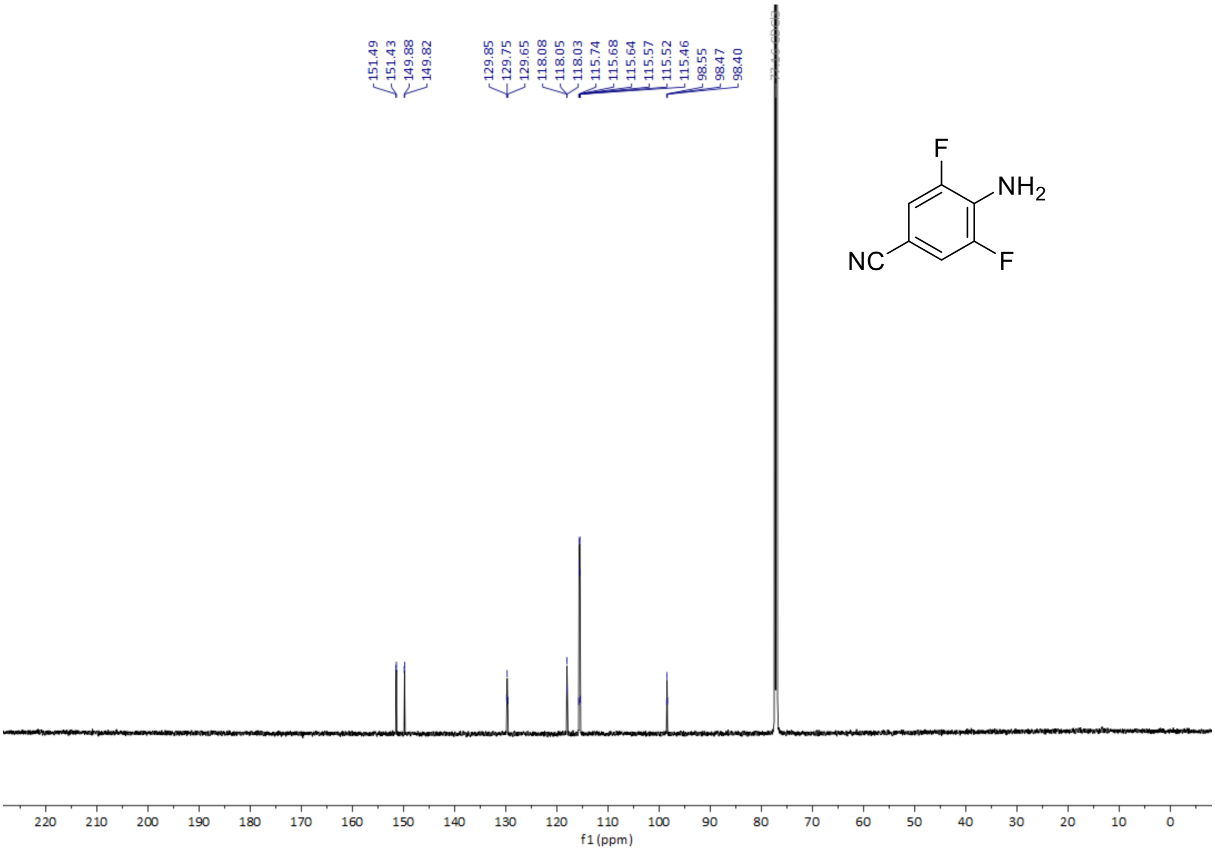

Figure S100: $^{13}C\{^{1}H\}$ NMR spectrum of **S8** in $CDCl_3$.

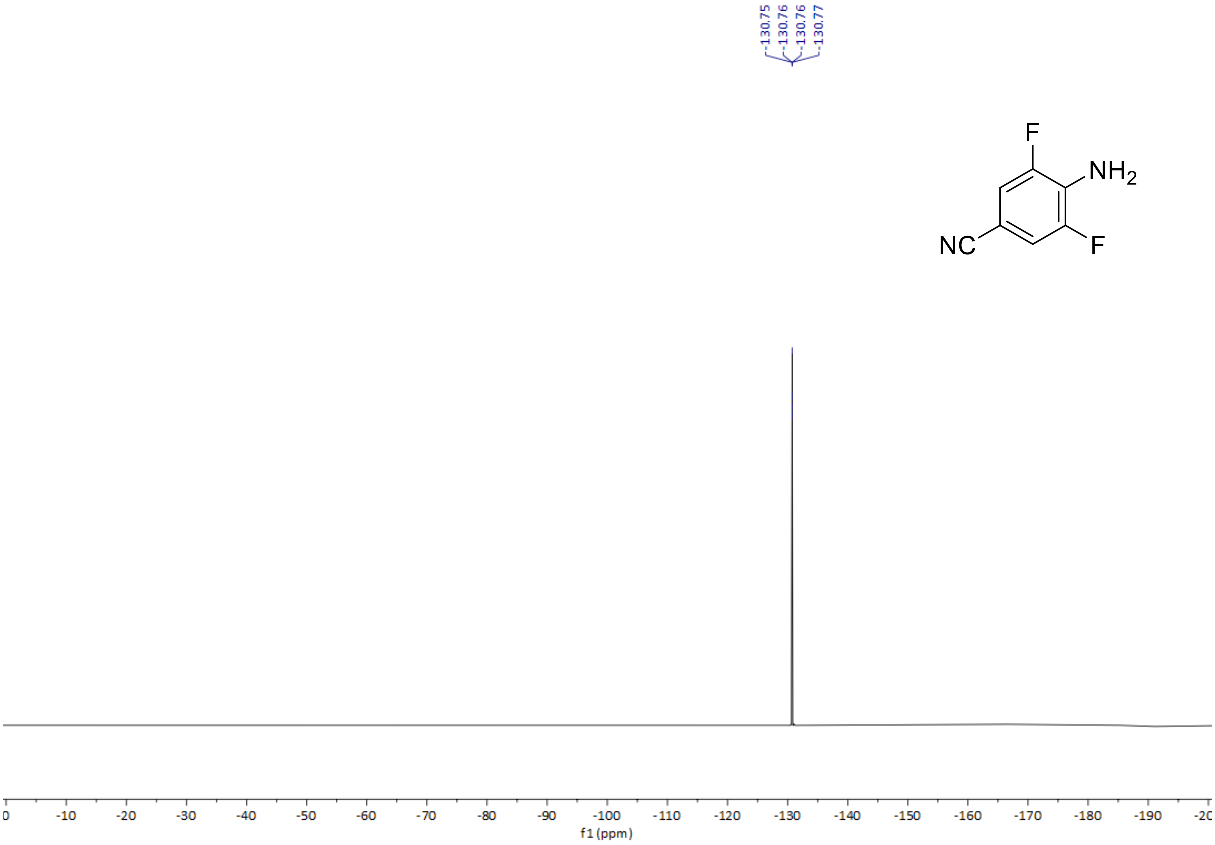

Figure S101: ^{19}F NMR spectrum of **S8** in $CDCl_3$.

4-Amino-3,5-difluorobenzoic acid (**S9**)

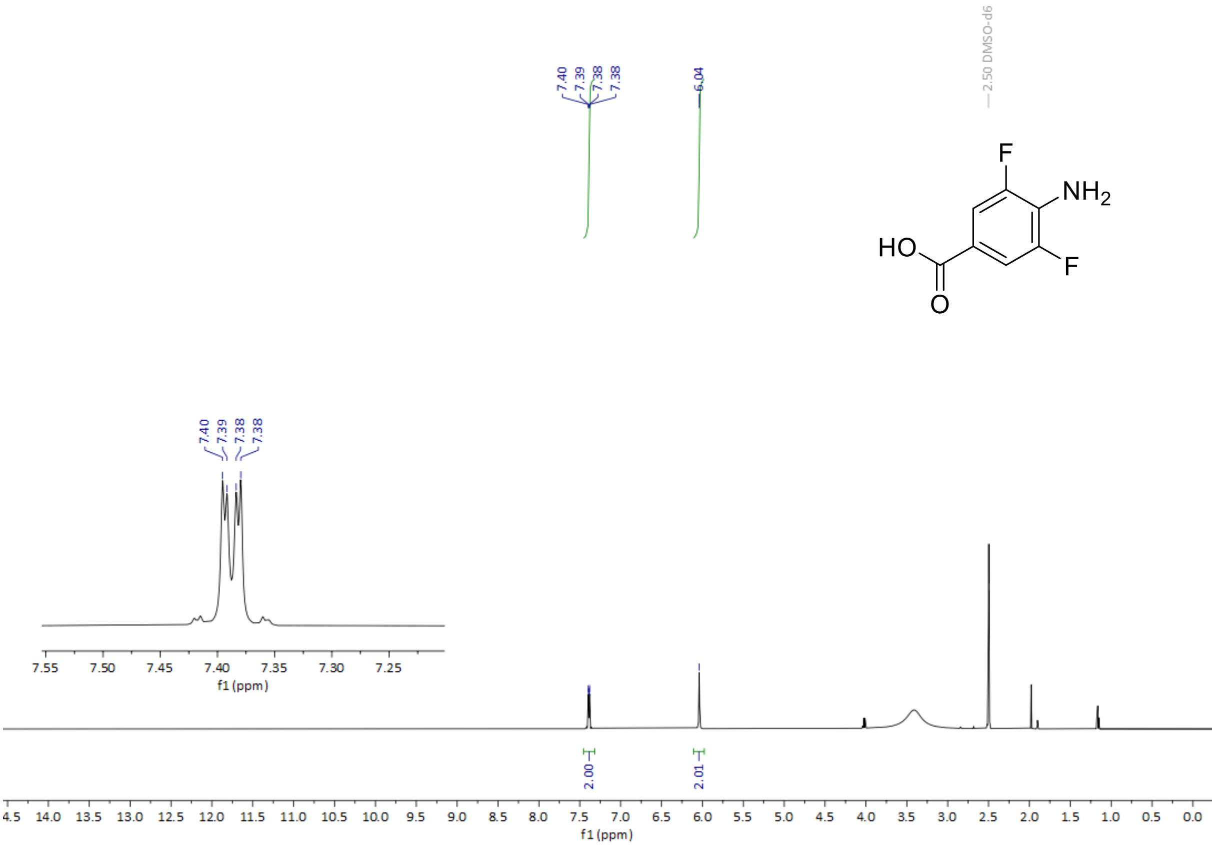

Figure S102: ^{1}H NMR spectrum of **S9** in DMSO-d_6.

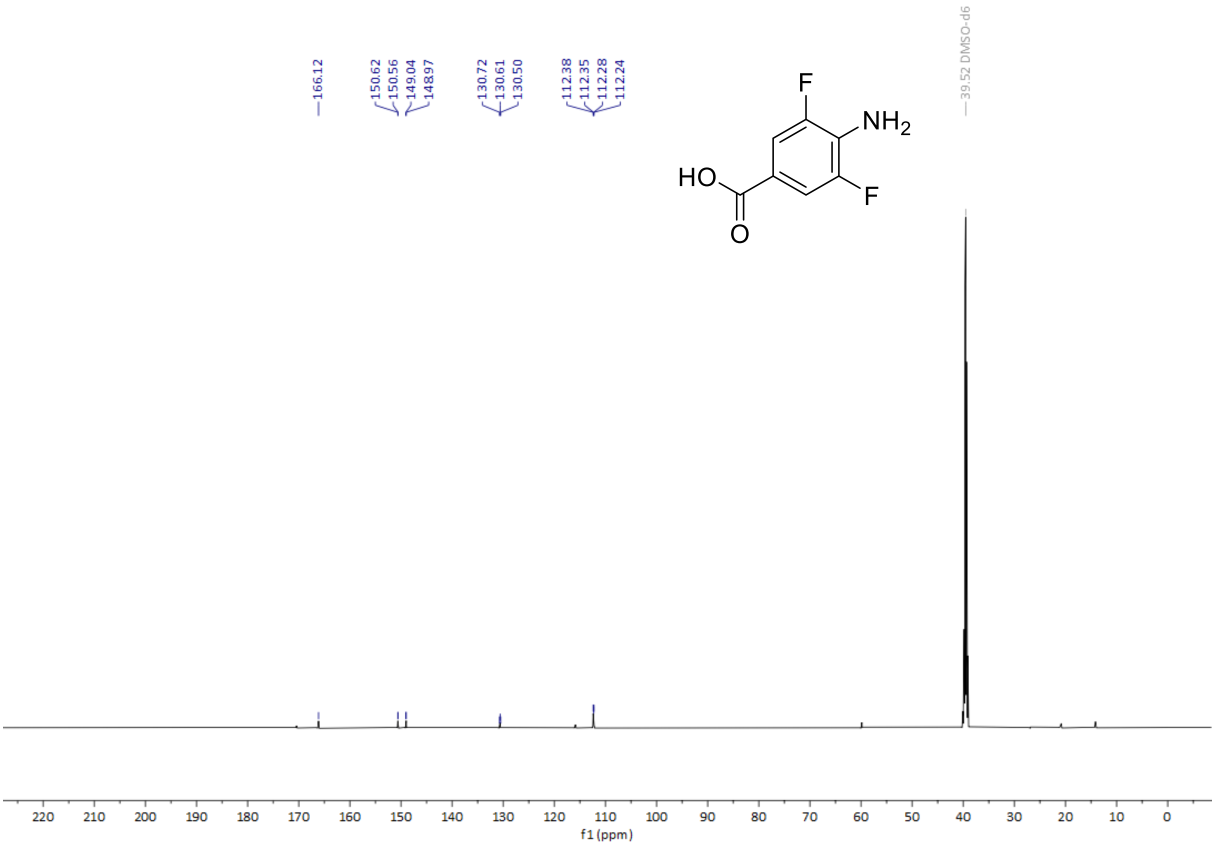

Figure S103: $^{13}C\{^{1}H\}$ NMR spectrum of **S9** in DMSO-d_6.

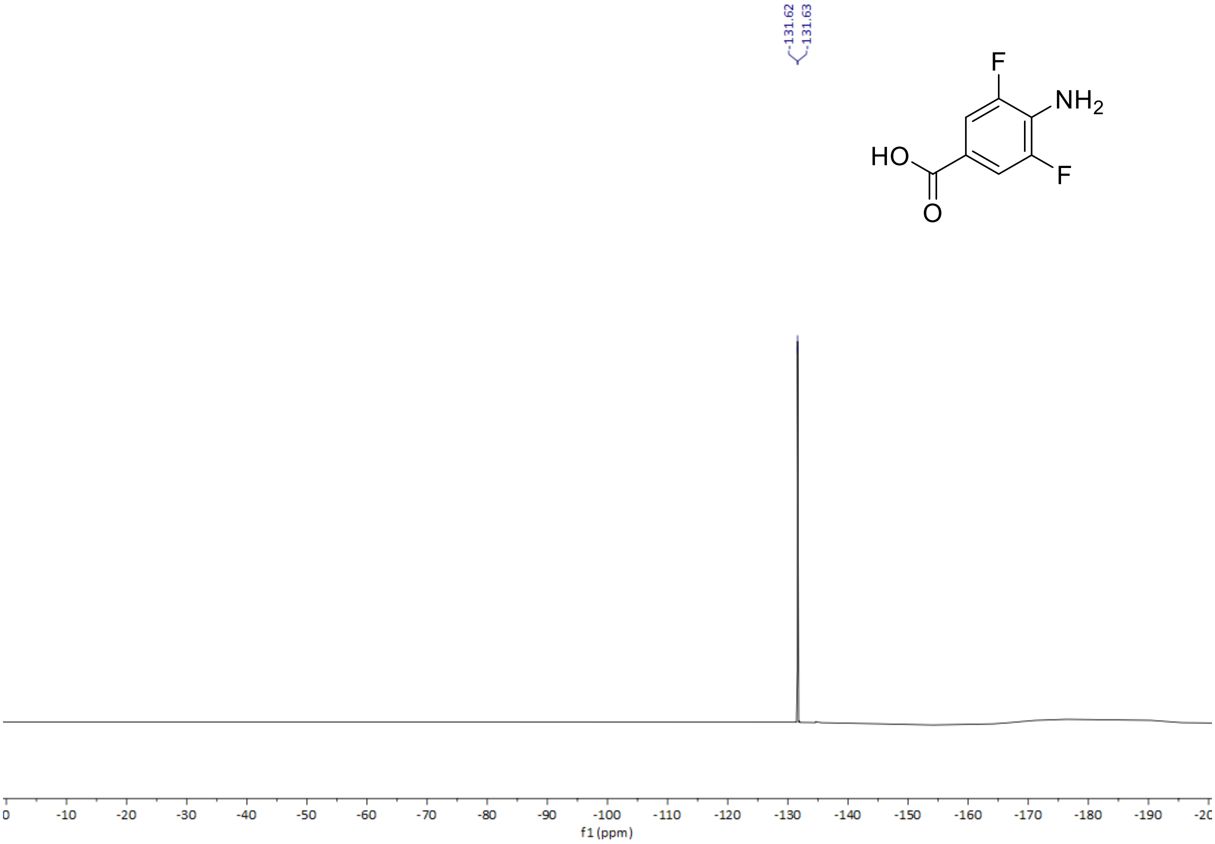

Figure S104: ^{19}F NMR spectrum of **S9** in DMSO-d_6.

Ethyl 4-amino-3,5-difluorobenzoate (**S10**)

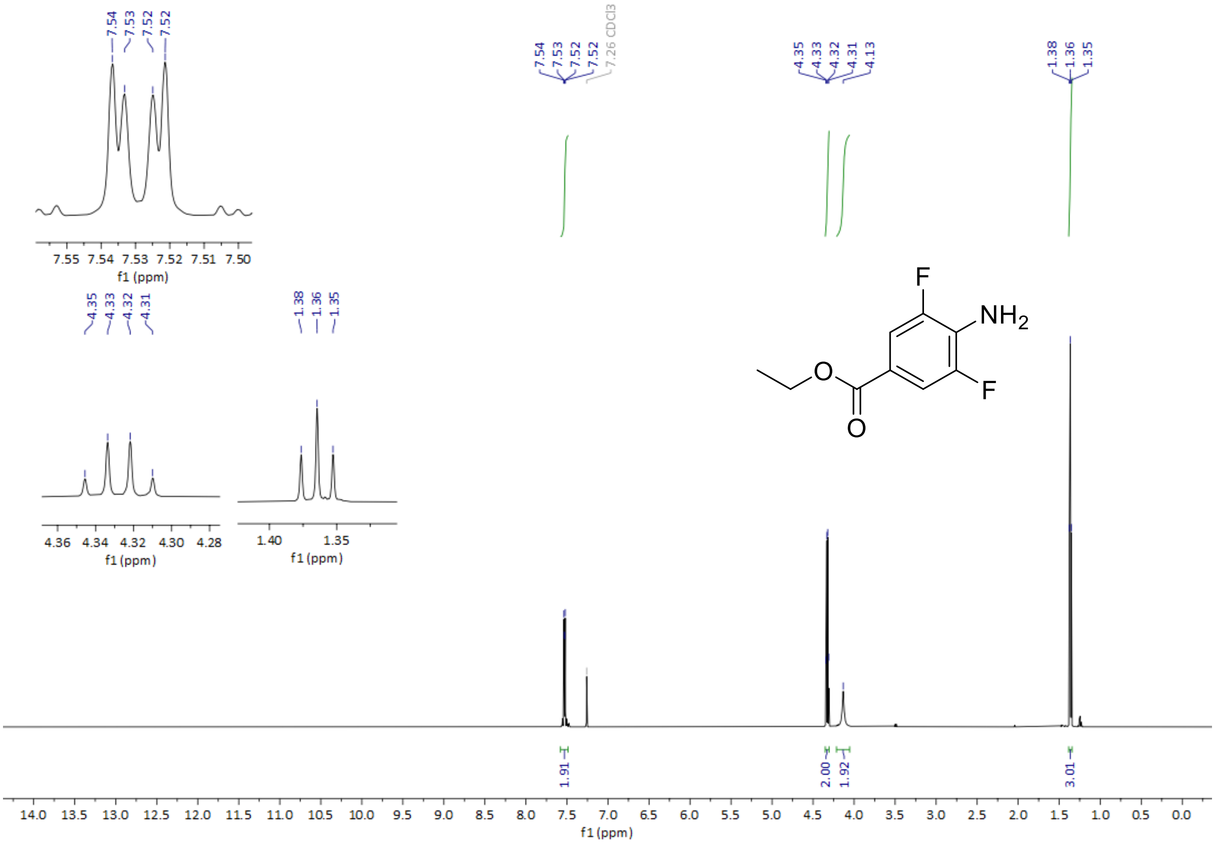

Figure S105: ^{1}H NMR spectrum of **S10** in $CDCl_3$.

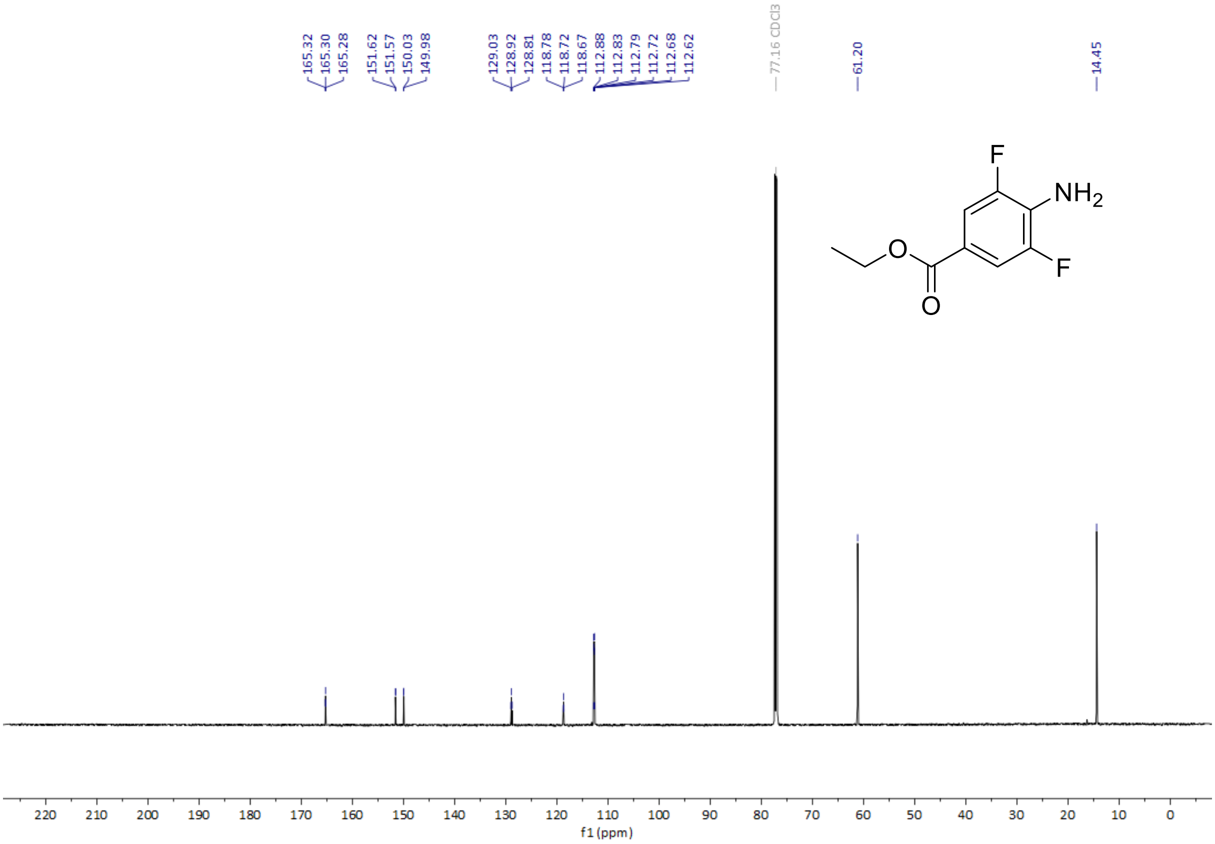

Figure S106: $^{13}C\{^{1}H\}$ NMR spectrum of **S10** in $CDCl_3$.

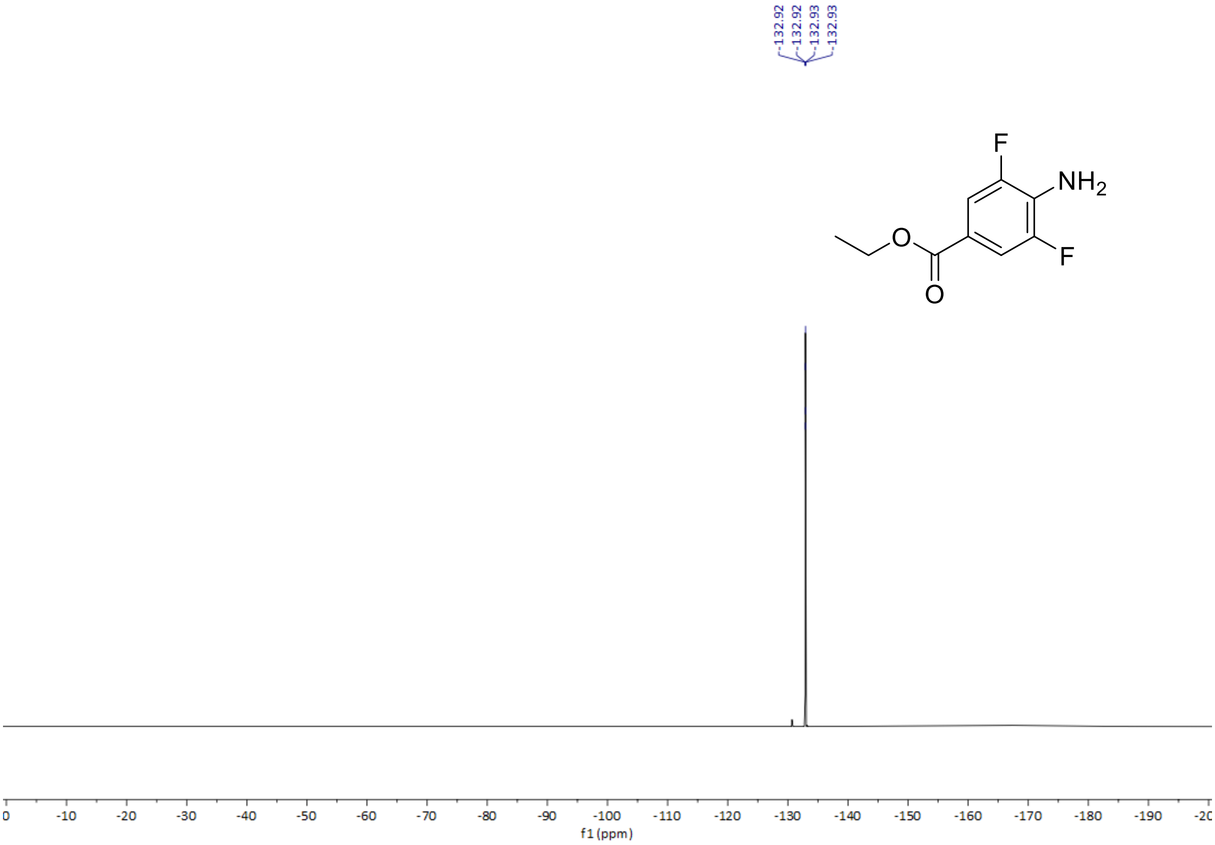

Figure S107: ^{19}F NMR spectrum of **S10** in $CDCl_3$.

Diethyl 4,4'-(diazene-1,2-diyl)(E)-bis(3,5-difluorobenzoate) (**S11**)

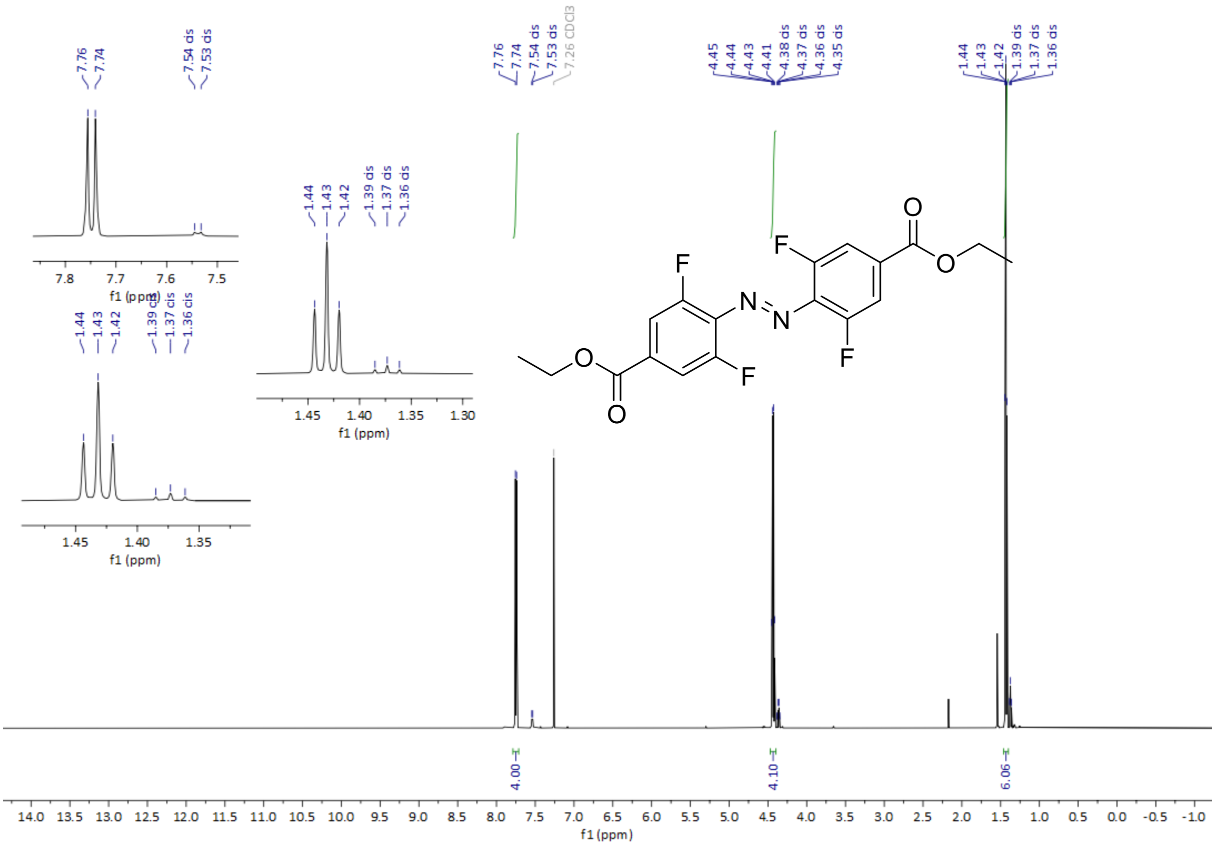

Figure S108:^{1}H NMR spectrum of **S11** in $CDCl_3$.

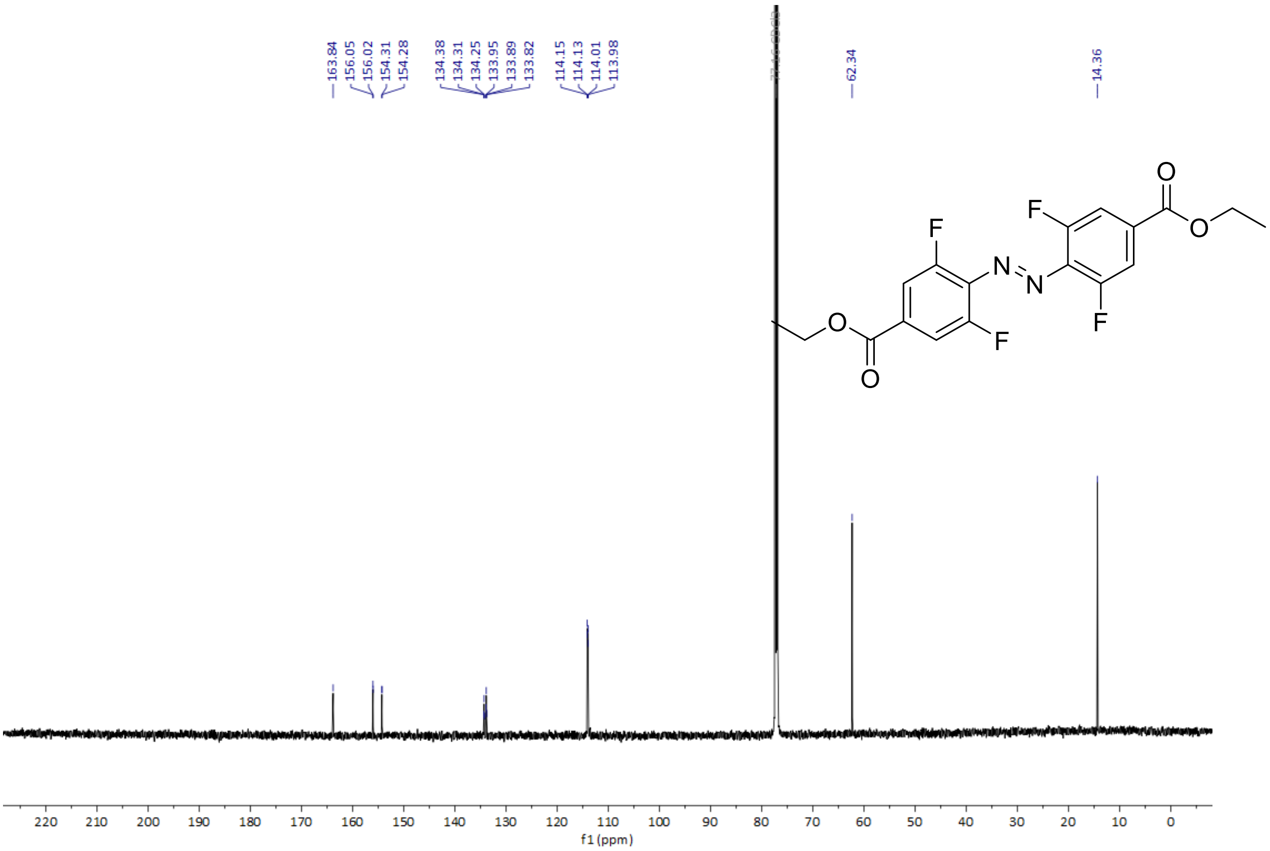

Figure S109: $^{13}C\{^{1}H\}$ NMR spectrum of **S11** in $CDCl_3$.

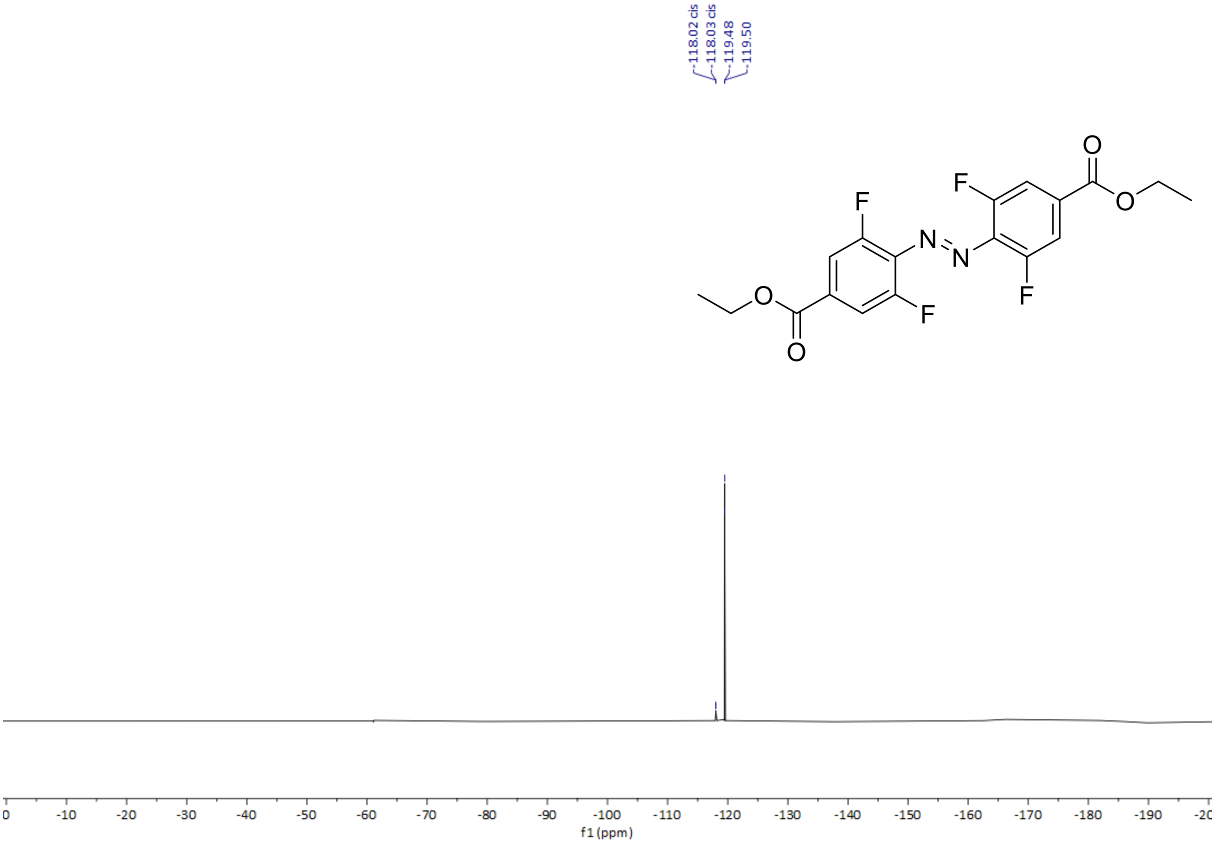

Figure S110: ^{19}F NMR spectrum of **S11** in $CDCl_3$.

(*E*)-4,4'-(Diazene-1,2-diyl)bis(3,5-difluorobenzoic acid) (**6**)

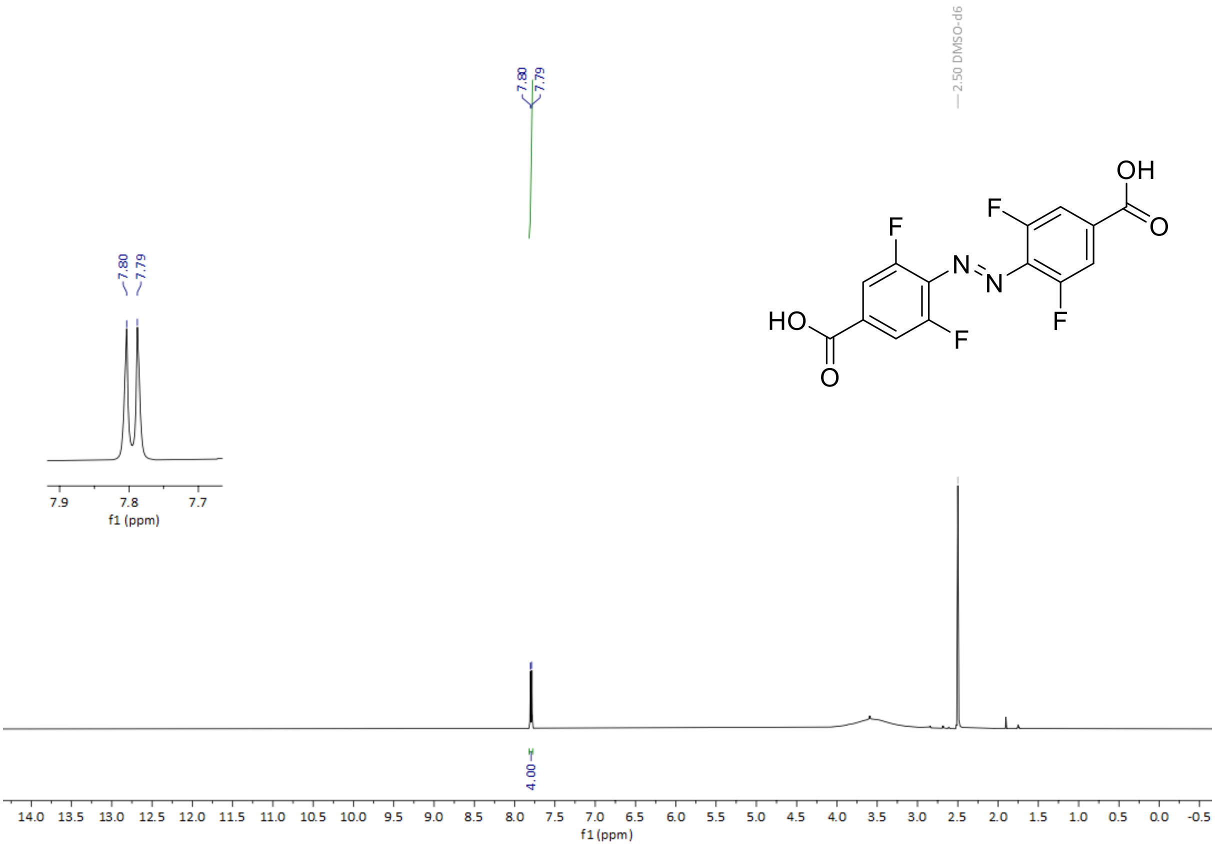

Figure S111:^{1}H NMR spectrum of **6** in DMSO-d_6.

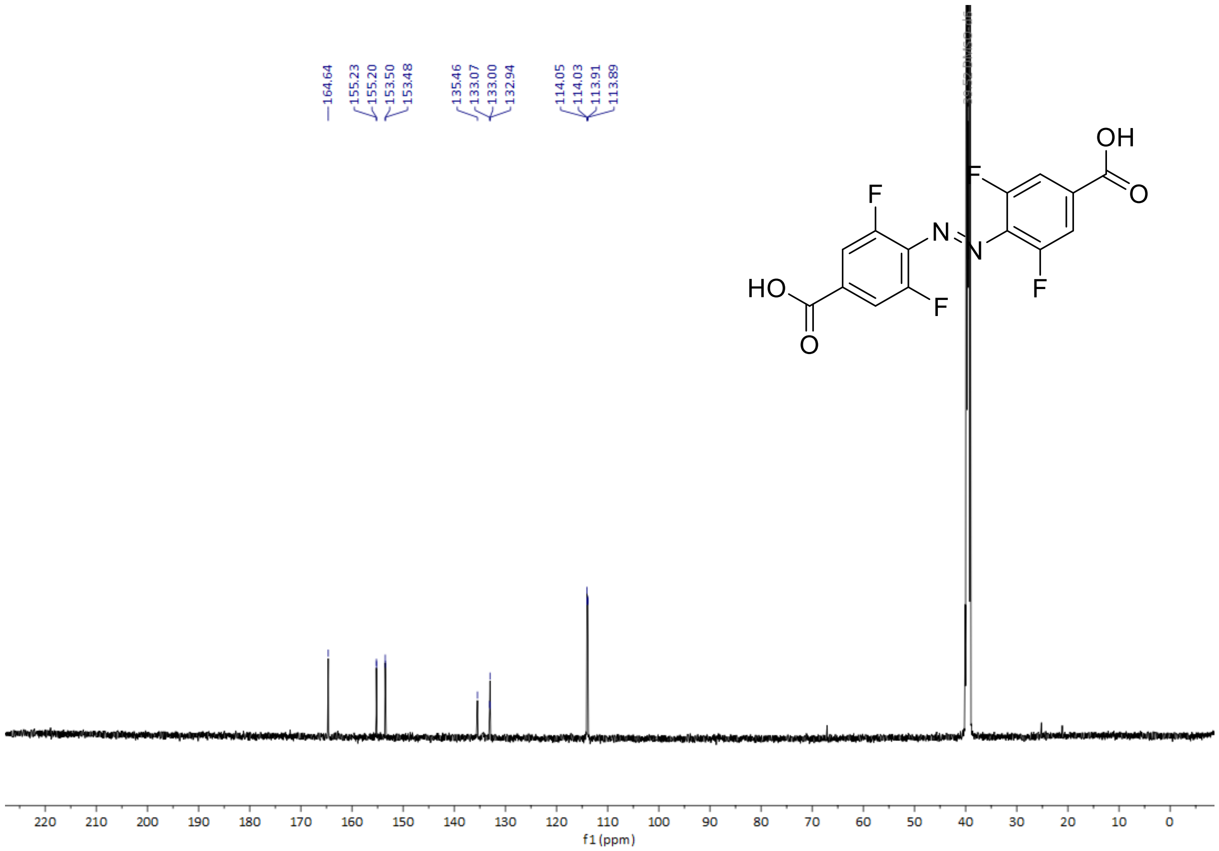

Figure S112: $^{13}C\{^{1}H\}$ NMR spectrum of **6** in DMSO-d_6.

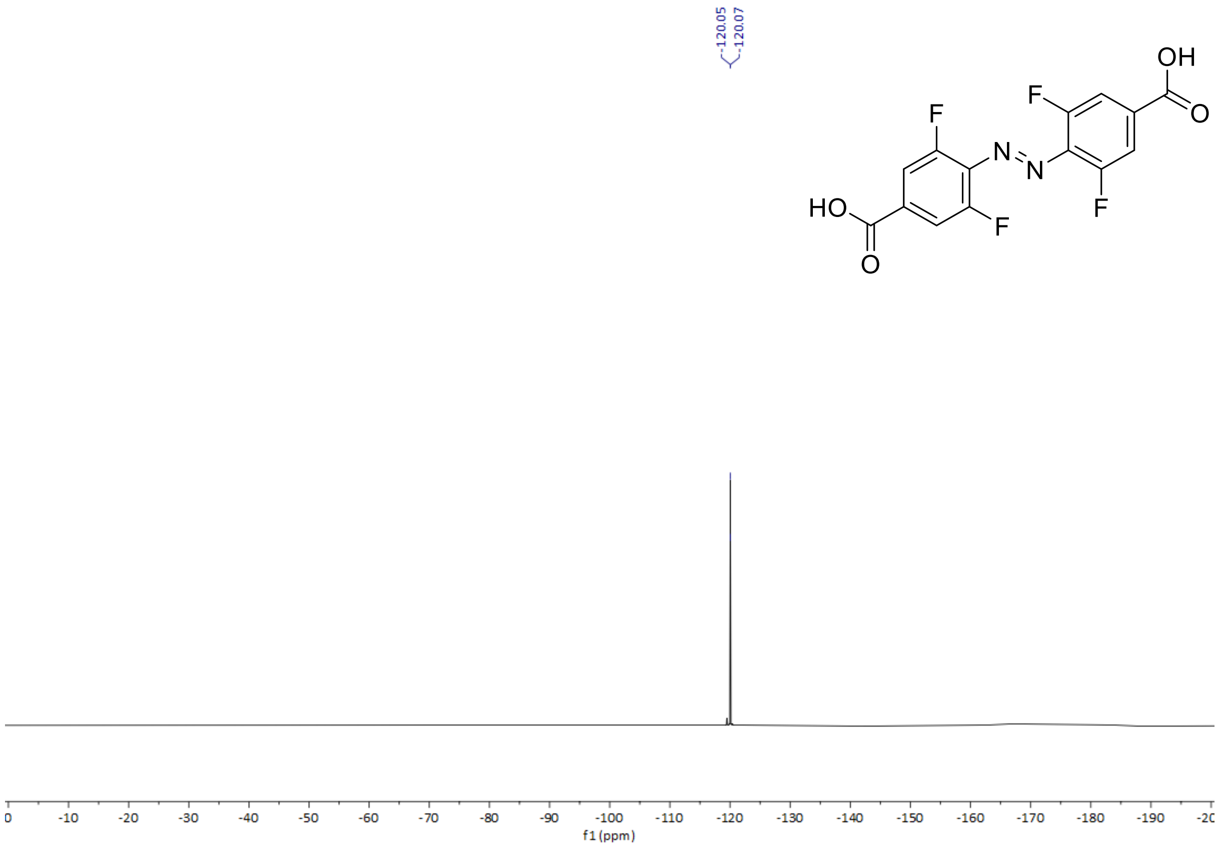

Figure S113: ^{19}F NMR spectrum of **6** in DMSO-d_6.

2-(2-(2-Aminophenethyl)phenyl)isoindoline-1,3-dione (**S12**)

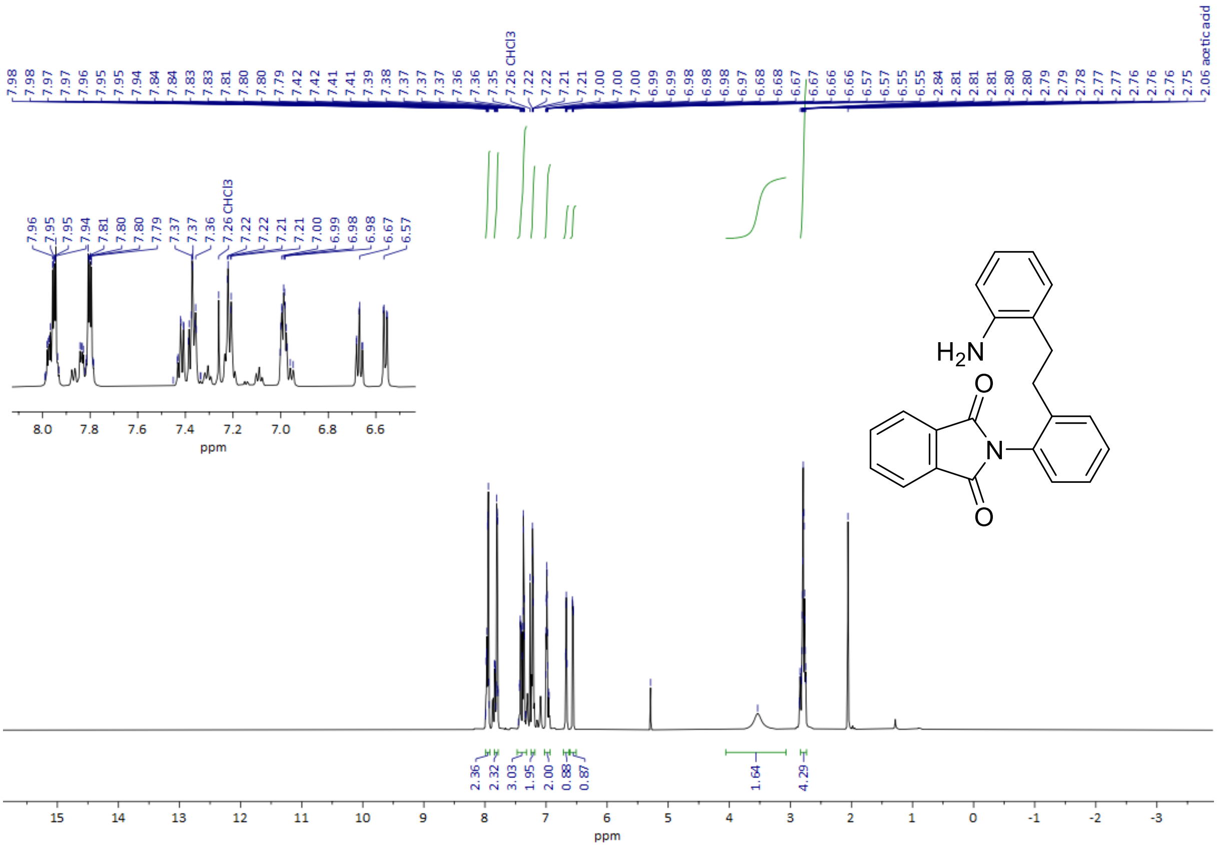

Figure S114: ^{1}H NMR spectrum of **S12** in $CDCl_3$.

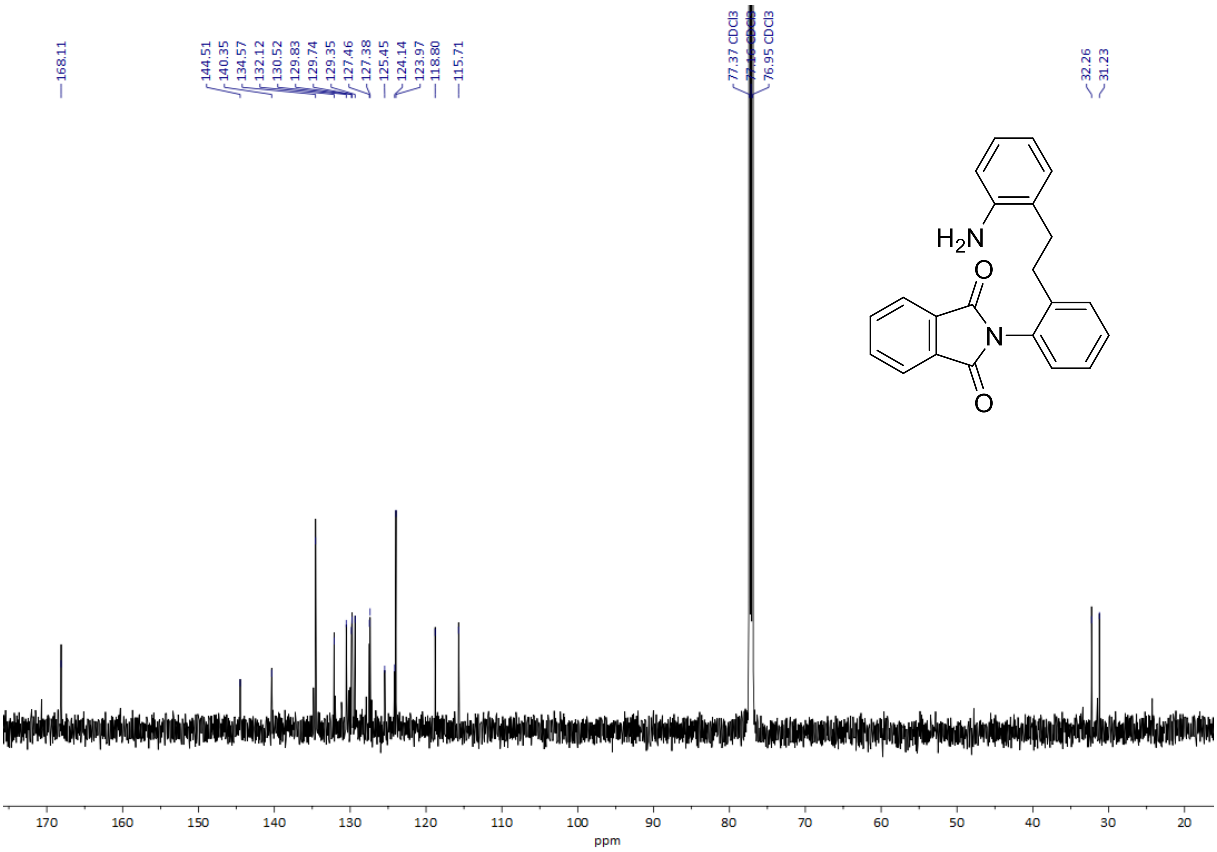

Figure S115: $^{13}C\{^{1}H\}$ NMR spectrum of **S12** in $CDCl_3$.

2-(2-Aminophenethyl)-4-iodoaniline (**S13**)

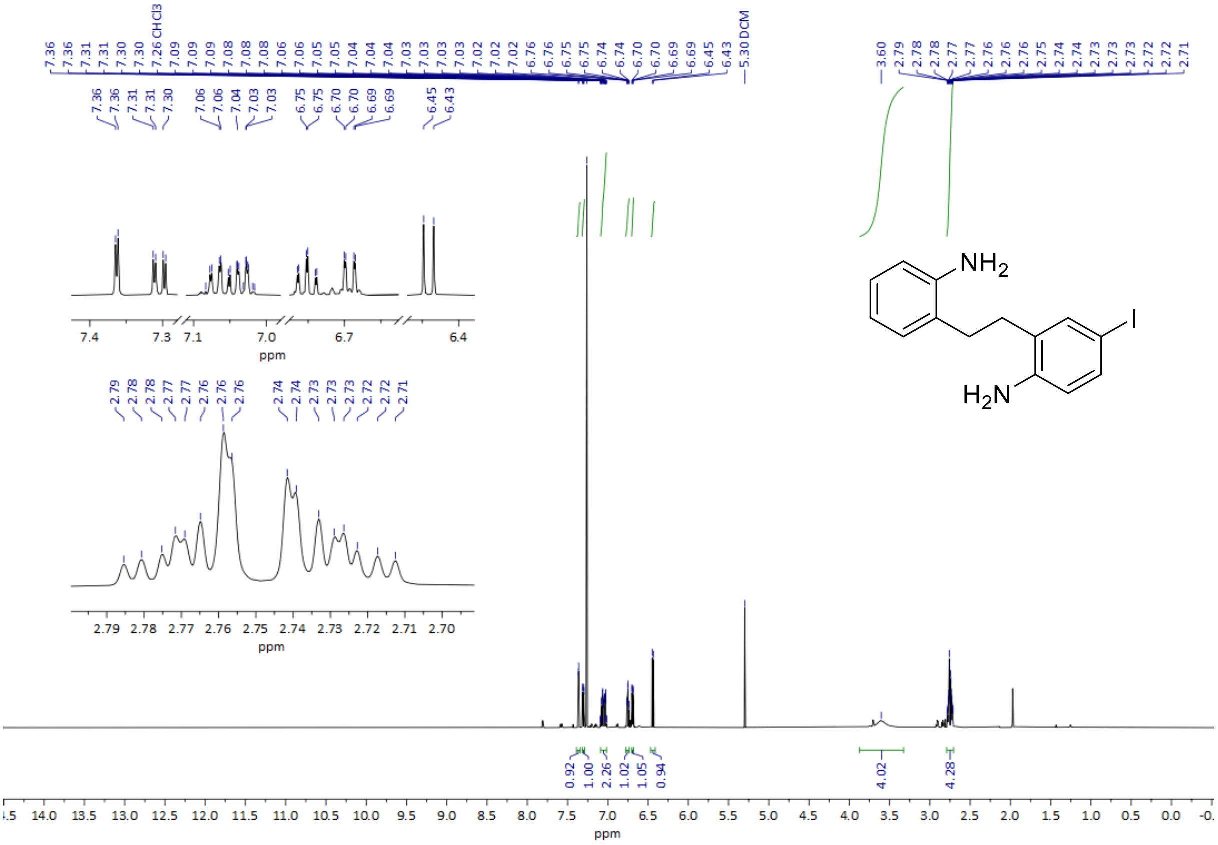

Figure S116: ^{1}H NMR spectrum of **S13** in $CDCl_3$.

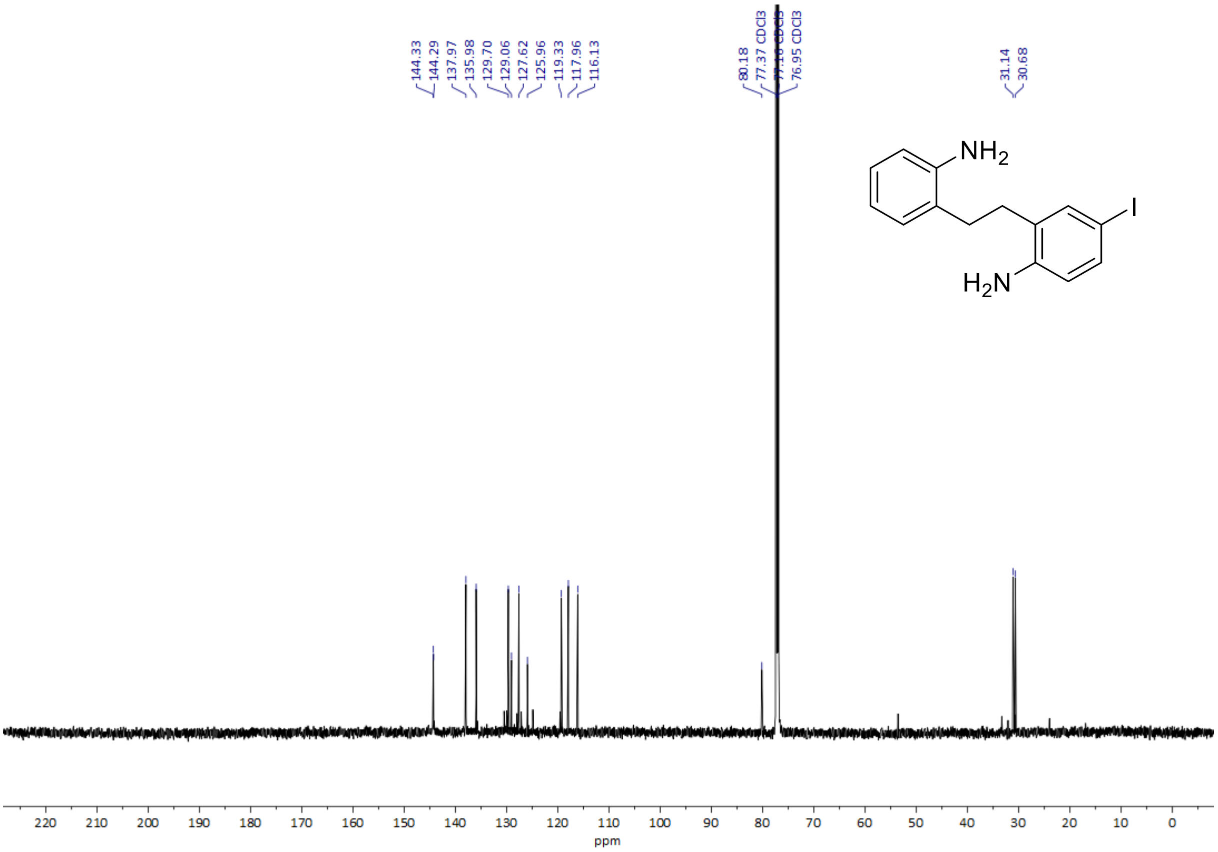

Figure S117: $^{13}C\{^{1}H\}$ NMR spectrum of **S13** in $CDCl_3$.

(*Z*)-2-Iodo-11,12-dihydrodibenzo[c,g][1,2]diazocine (**7a**)

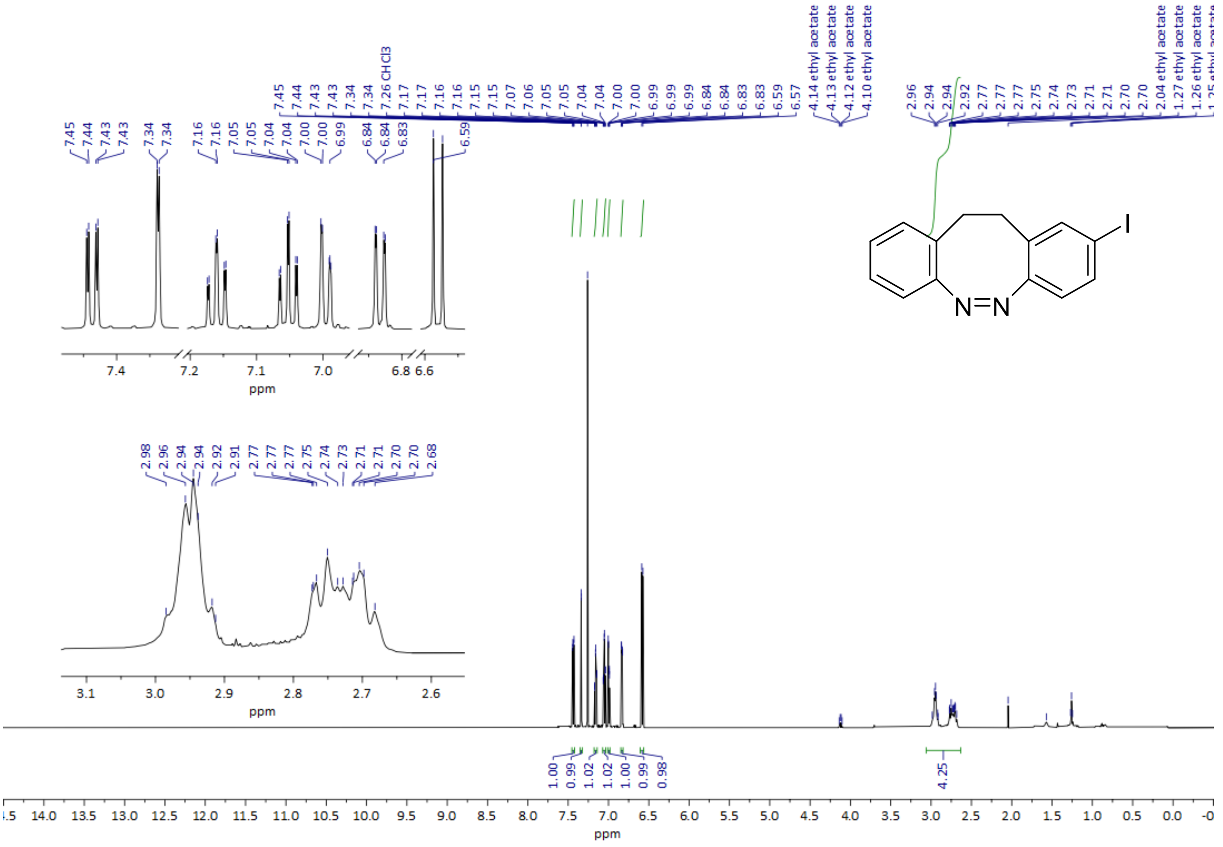

Figure S1218: ^{1}H NMR spectrum of **7a** in $CDCl_3$.

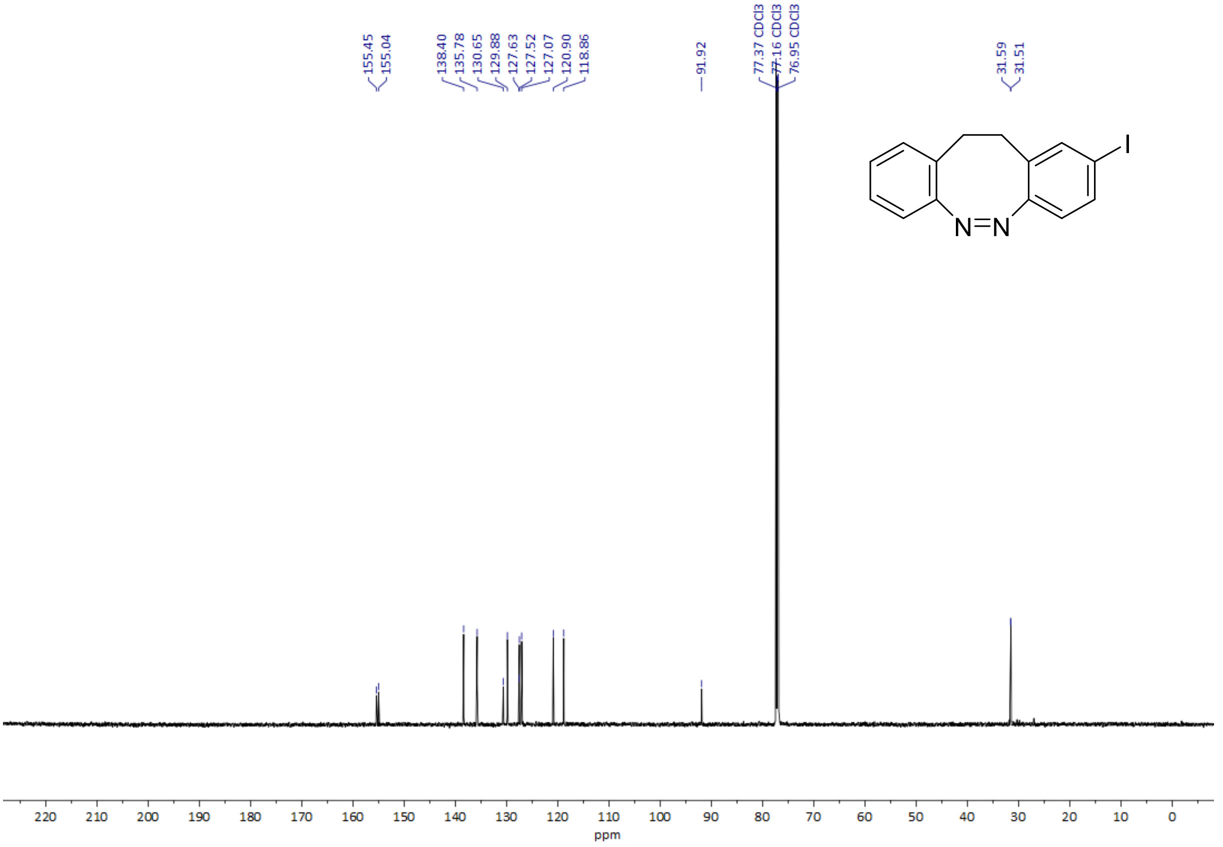

Figure S119: $^{13}C\{^{1}H\}$ NMR spectrum of **7a** in $CDCl_3$.

2,2'-(Ethane-1,2-diyl)bis(4-iodoaniline) (**S14**)

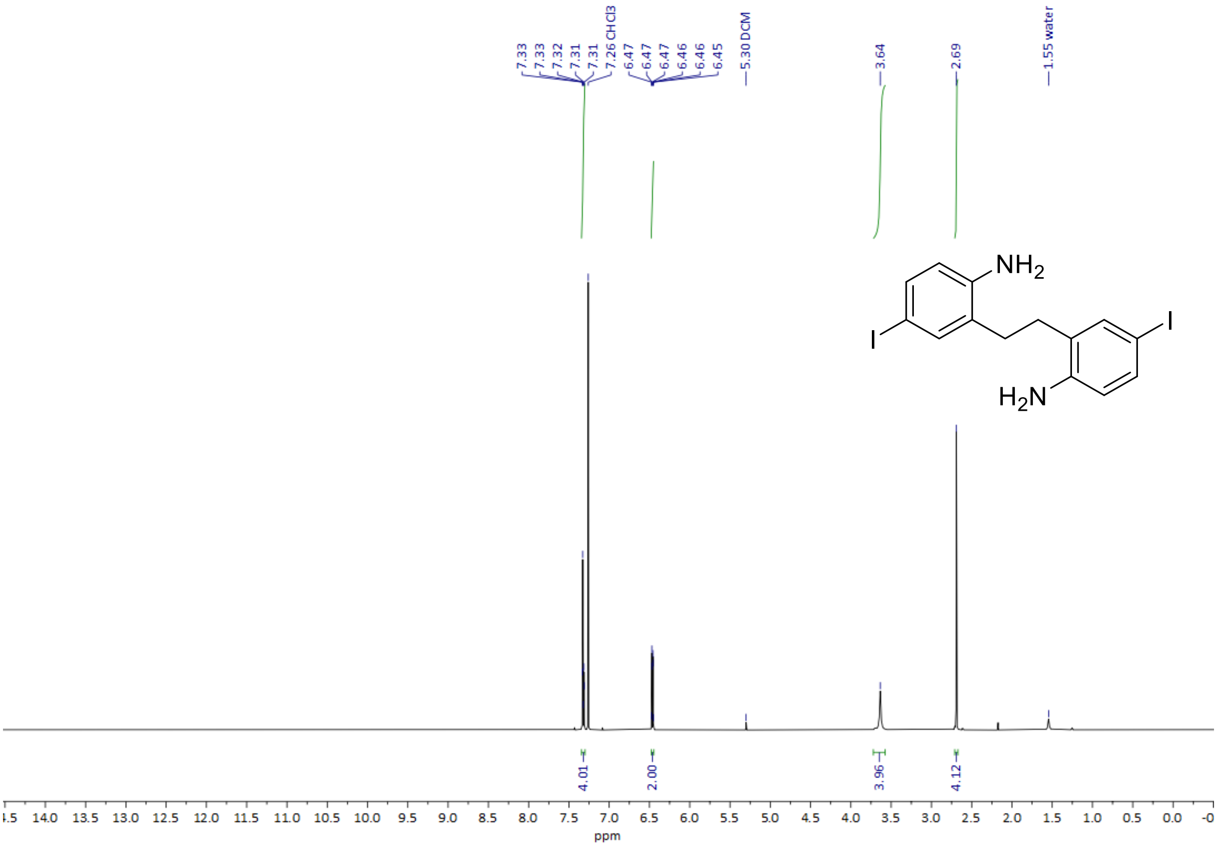

Figure S120: ^{1}H NMR spectrum of **S14** in $CDCl_3$.

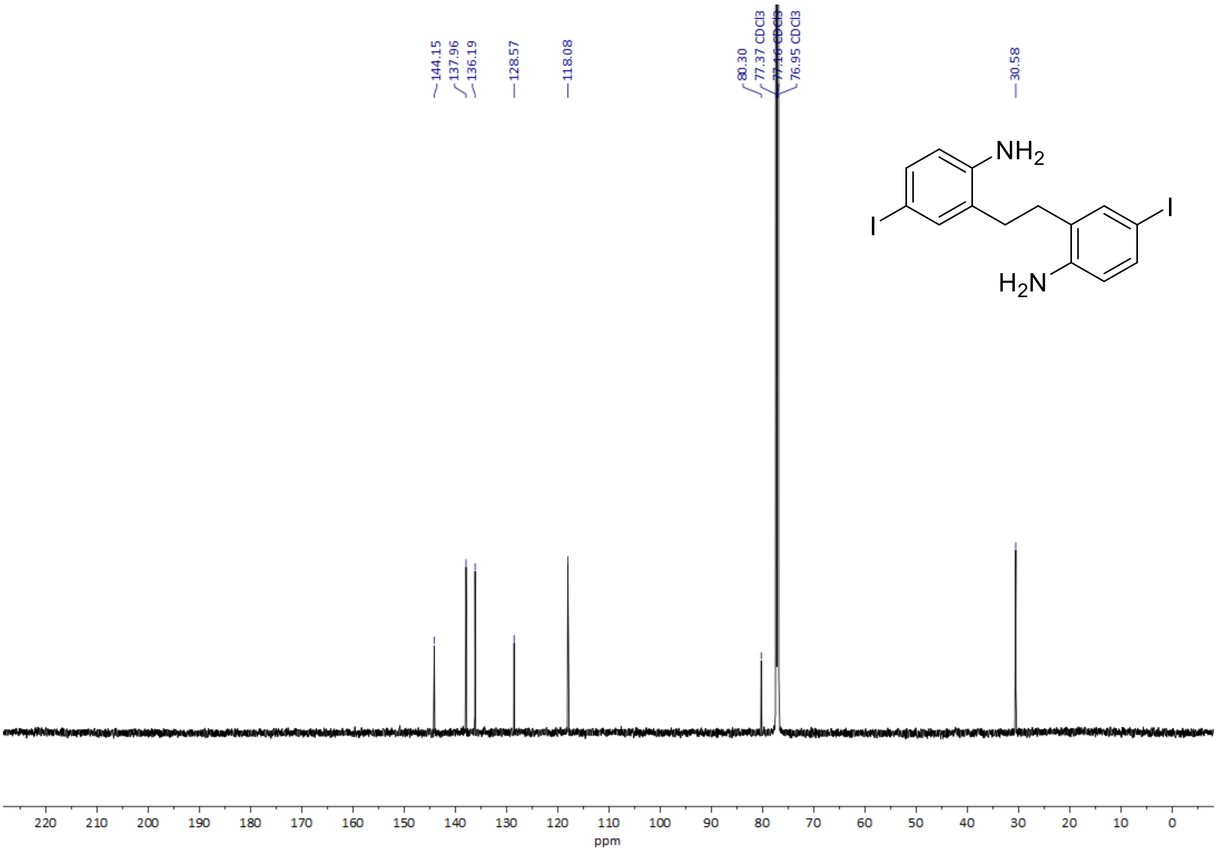

Figure S121: $^{13}C\{^{1}H\}$ NMR spectrum of **S14** in $CDCl_3$.

(*Z*)-2,9-Diiodo-11,12-dihydrodibenzo[c,g][1,2]diazocine (**7b**)

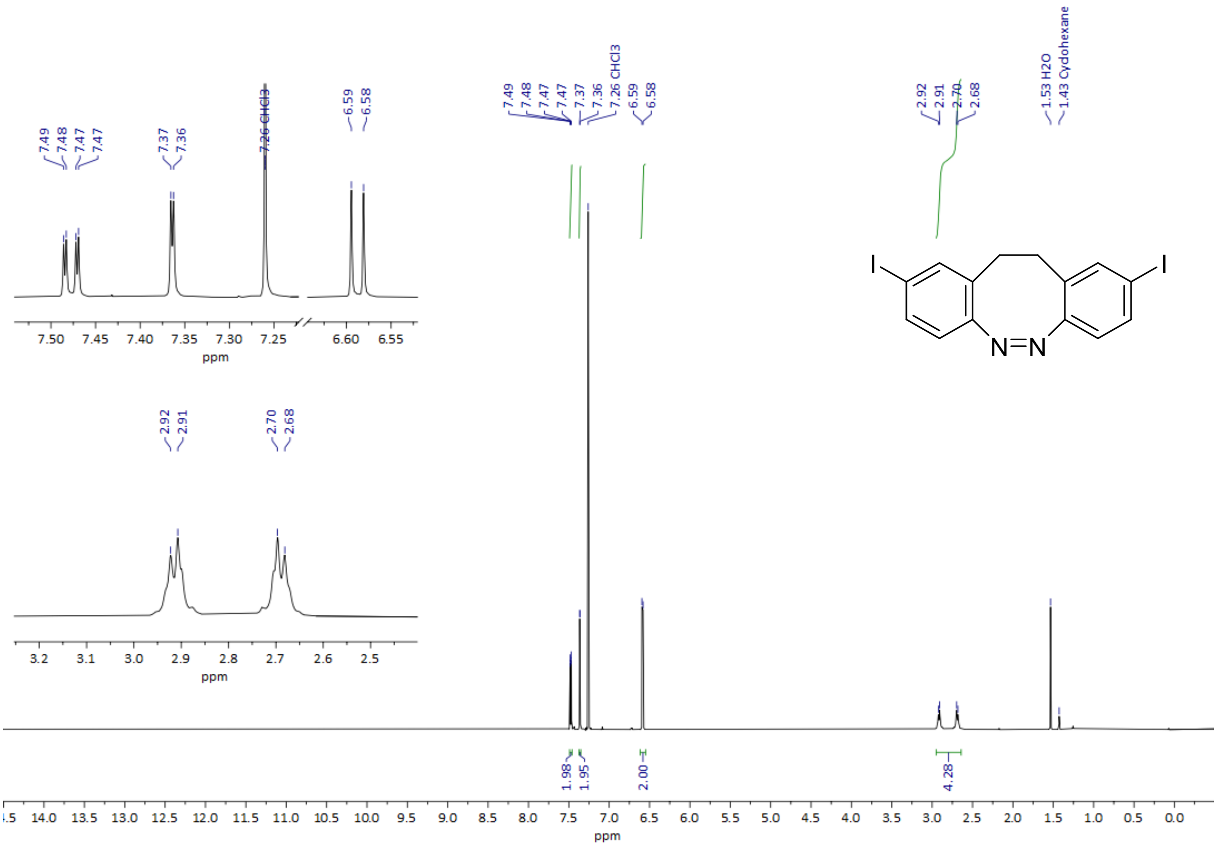

Figure S122: ^{1}H NMR spectrum of **7b** in $CDCl_3$.

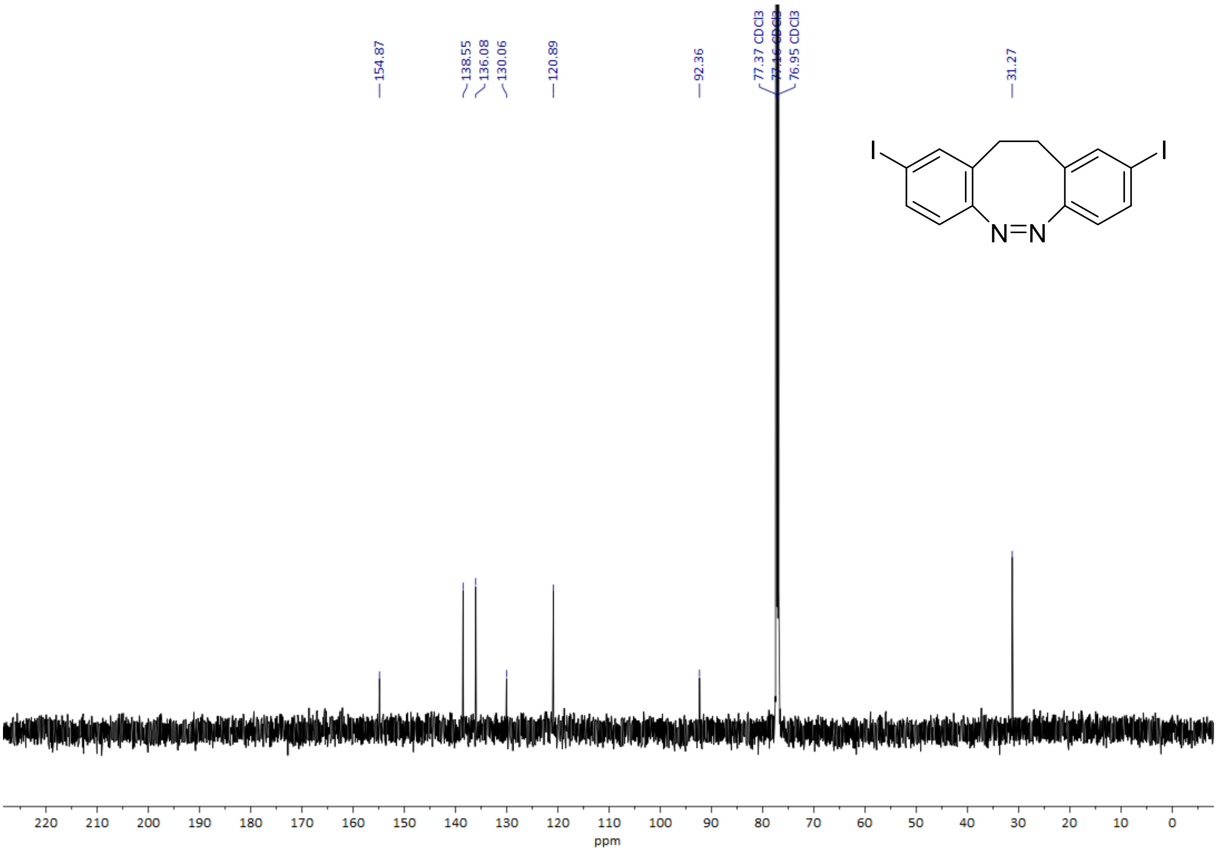

Figure S123: $^{13}C\{^{1}H\}$ NMR spectrum of **7b** in $CDCl_3$.

^{1}H NMR Spectra for the Determination of the ^{1}H NMR Yield

2,5-Dioxopyrrolidin-1-yl (*E*)-4-(phenyldiazenyl)benzoate (**3a**)

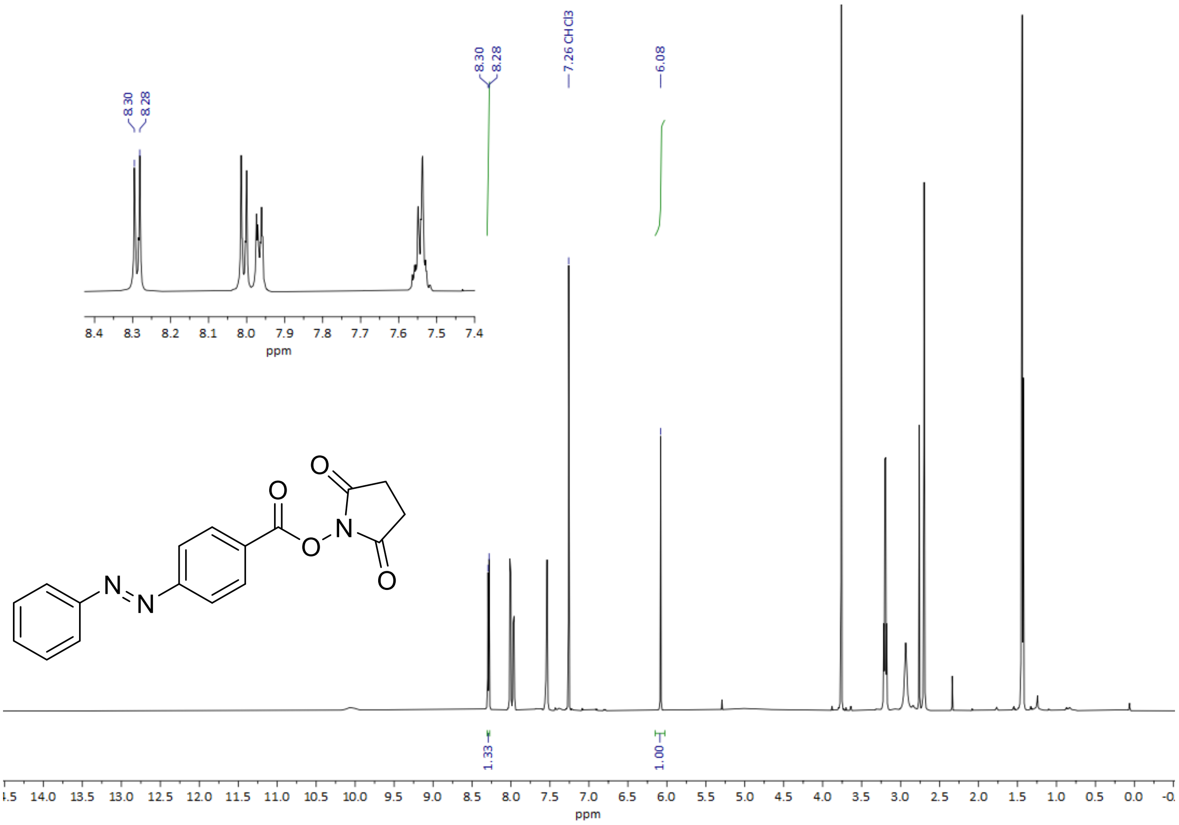

Figure S124: Crude ^{1}H NMR spectrum of **3a** in $CDCl_3$.

2,5-Dioxopyrrolidin-1-yl (*E*)-3-(phenyldiazenyl)benzoate (**3b**)

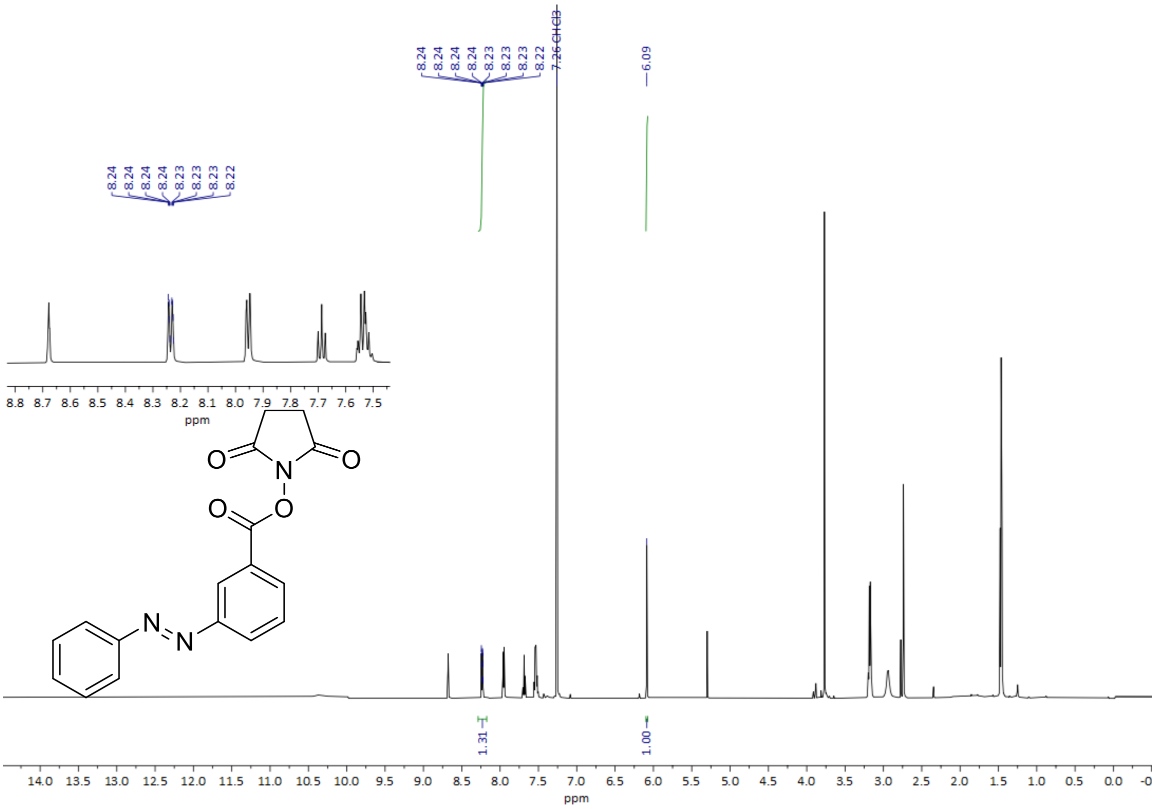

Figure S125: Crude ^{1}H NMR spectrum of **3b** in $CDCl_3$.

2,5-Dioxopyrrolidin-1-yl (*E*)-2-(phenyldiazenyl)benzoate (**3c**)

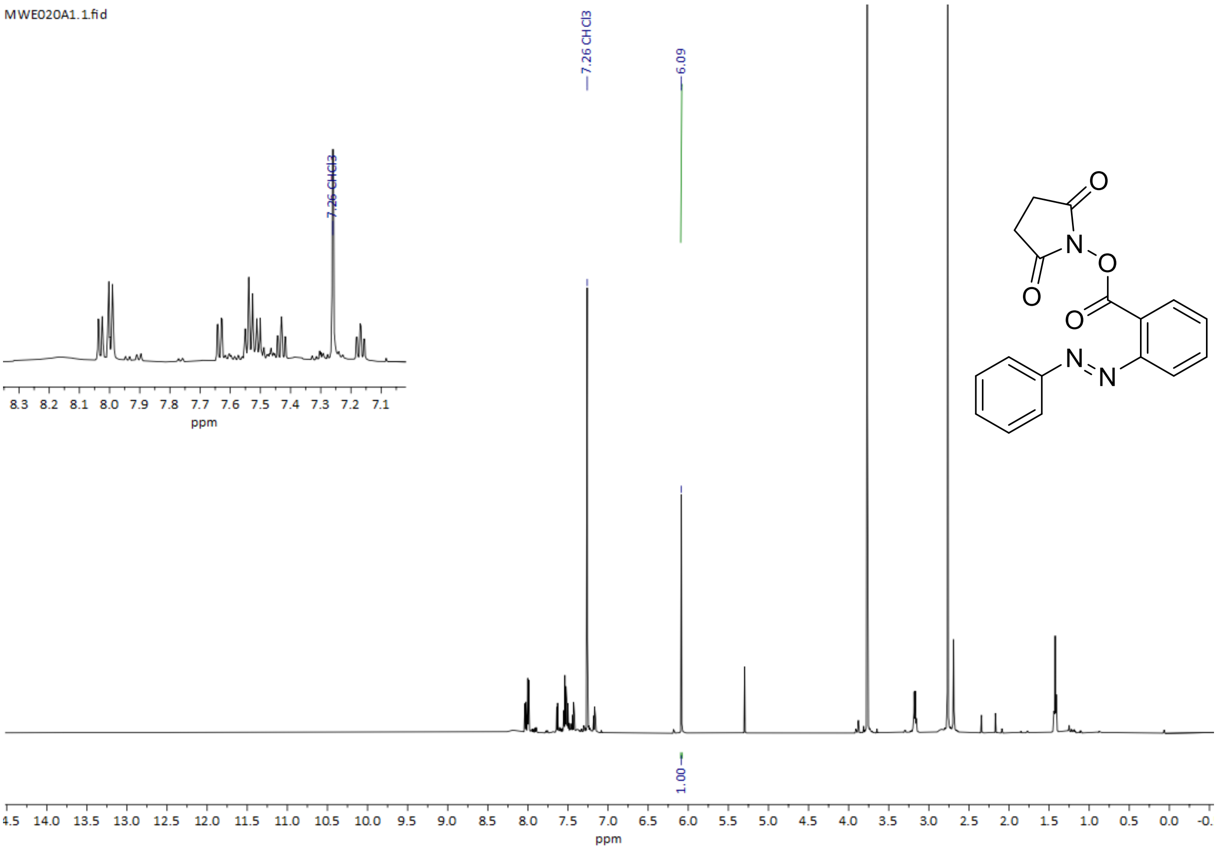

Figure S126: Crude ^{1}H NMR spectrum of **3c** in $CDCl_3$.

2,5-Dioxopyrrolidin-1-yl (*E*)-4-((4-hydroxyphenyl)diazenyl)benzoate (**3d**)

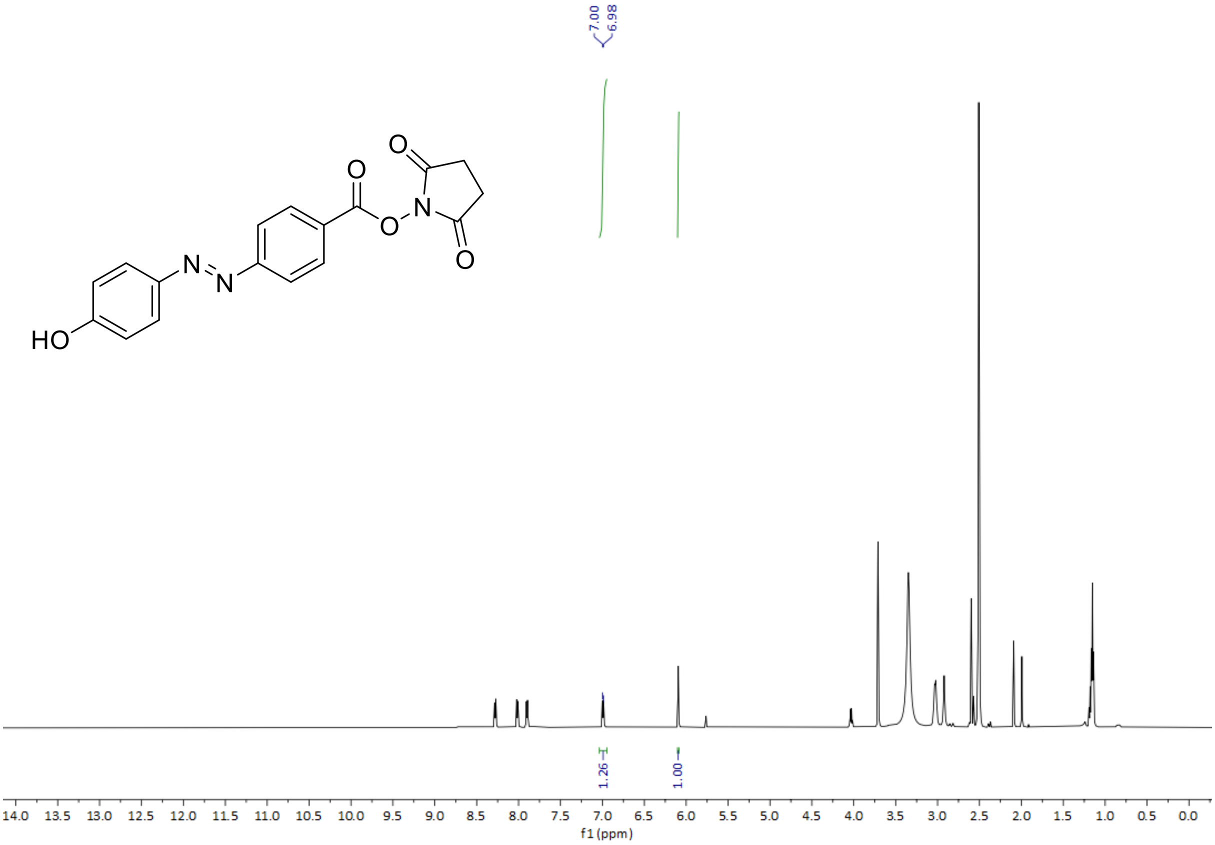

Figure S127: Crude ^{1}H NMR spectrum of **3d** in DMSO-d_6.

2,5-Dioxopyrrolidin-1-yl (*E*)-4-((4-aminophenyl)diazenyl)benzoate (**3e**)

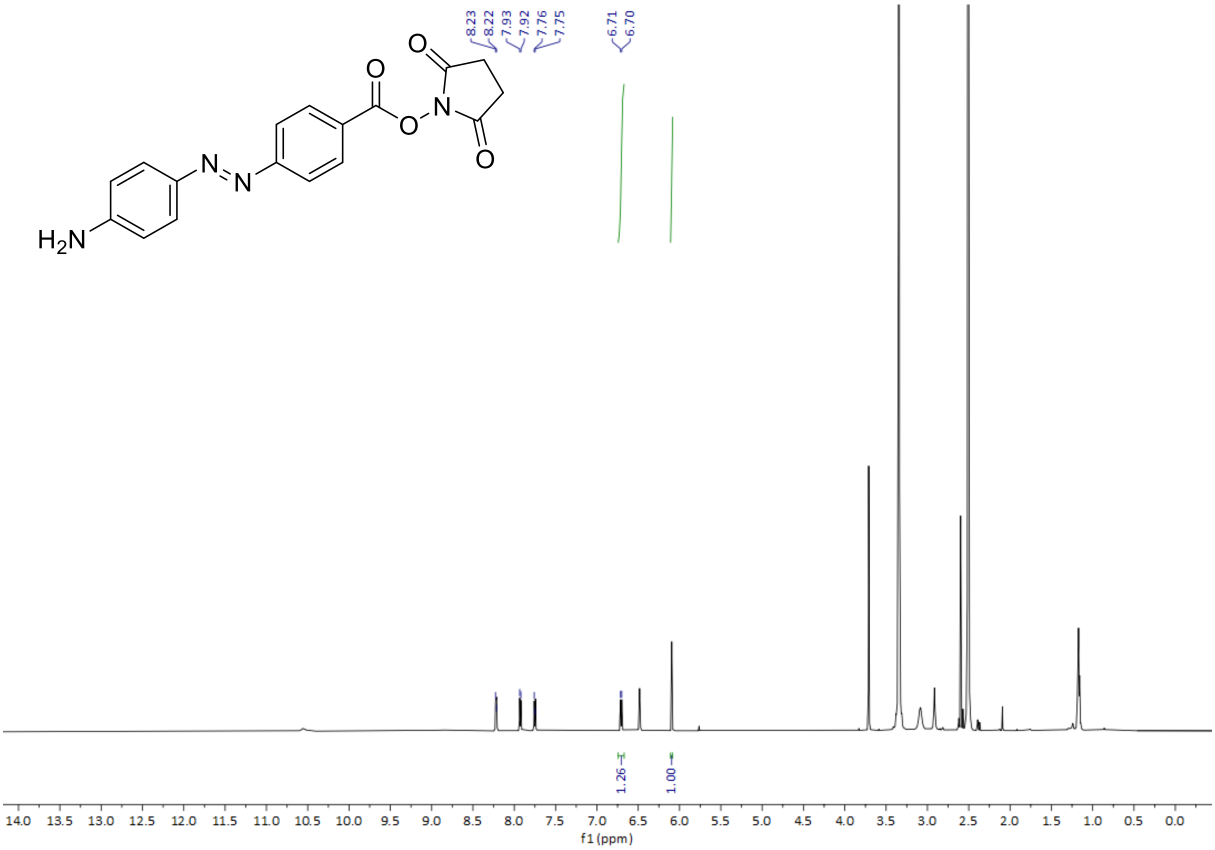

Figure S128: Crude ^{1}H NMR spectrum of **3e** in $CDCl_3$.

2,5-Dioxopyrrolidin-1-yl (*E*)-4-((4-acetamidophenyl)diazenyl)benzoate (**3f**)

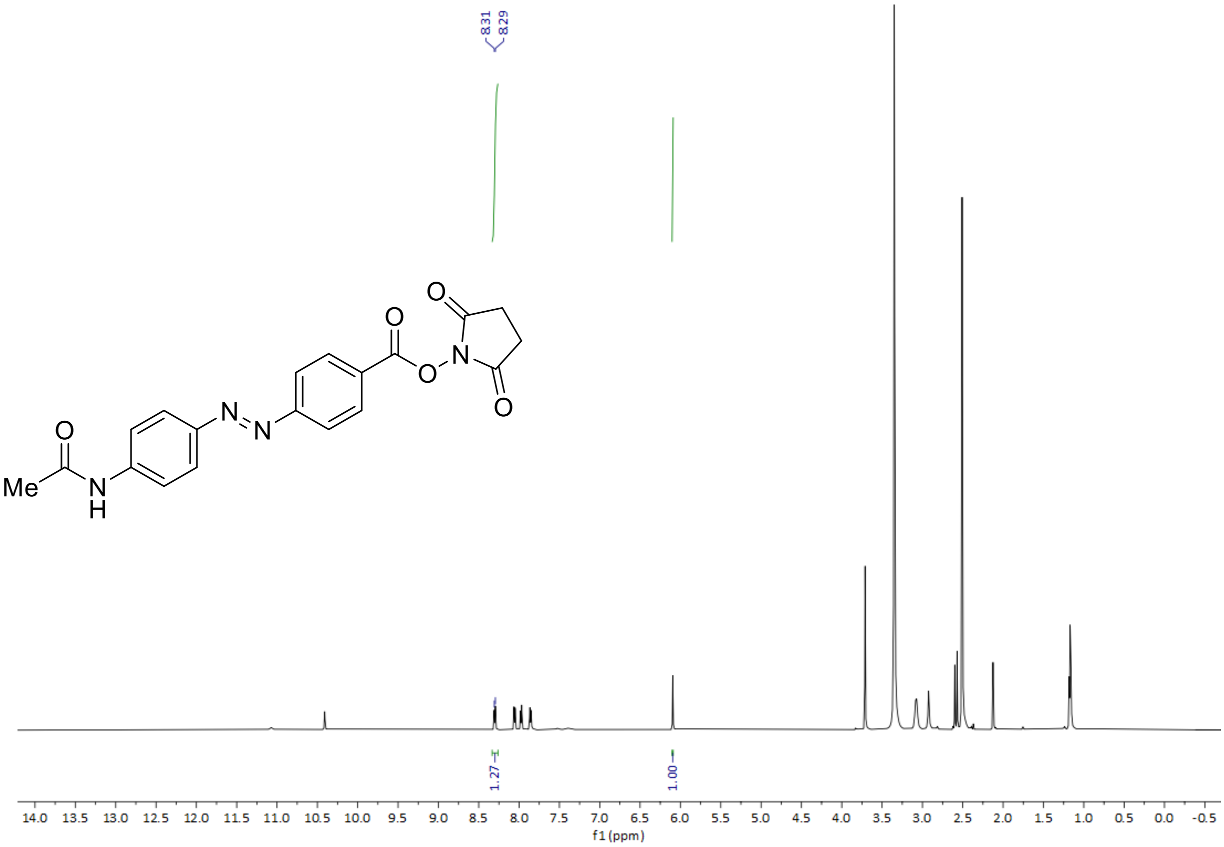

Figure S129: Crude ^{1}H NMR spectrum of **3f** in $CDCl_3$.

2,5-Dioxopyrrolidin-1-yl (*E*)-4-((4-(allyloxy)phenyl)diazenyl)benzoate (**3h**)

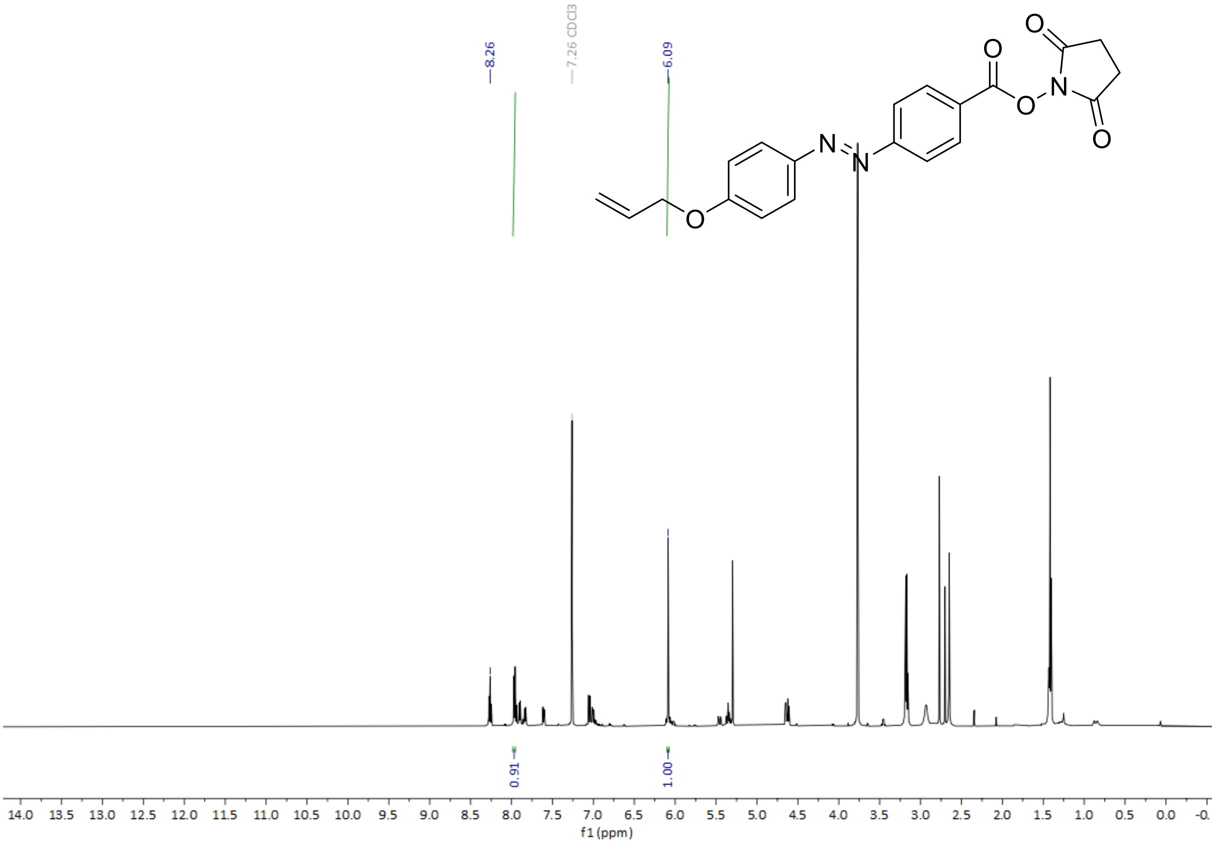

Figure S130: Crude ^{1}H NMR spectrum of **3h** in $CDCl_3$.

2,5-Dioxopyrrolidin-1-yl (*E*)-4-((4-((9-hydroxynonyl)oxy)phenyl)diazenyl)benzoate (**3i**)

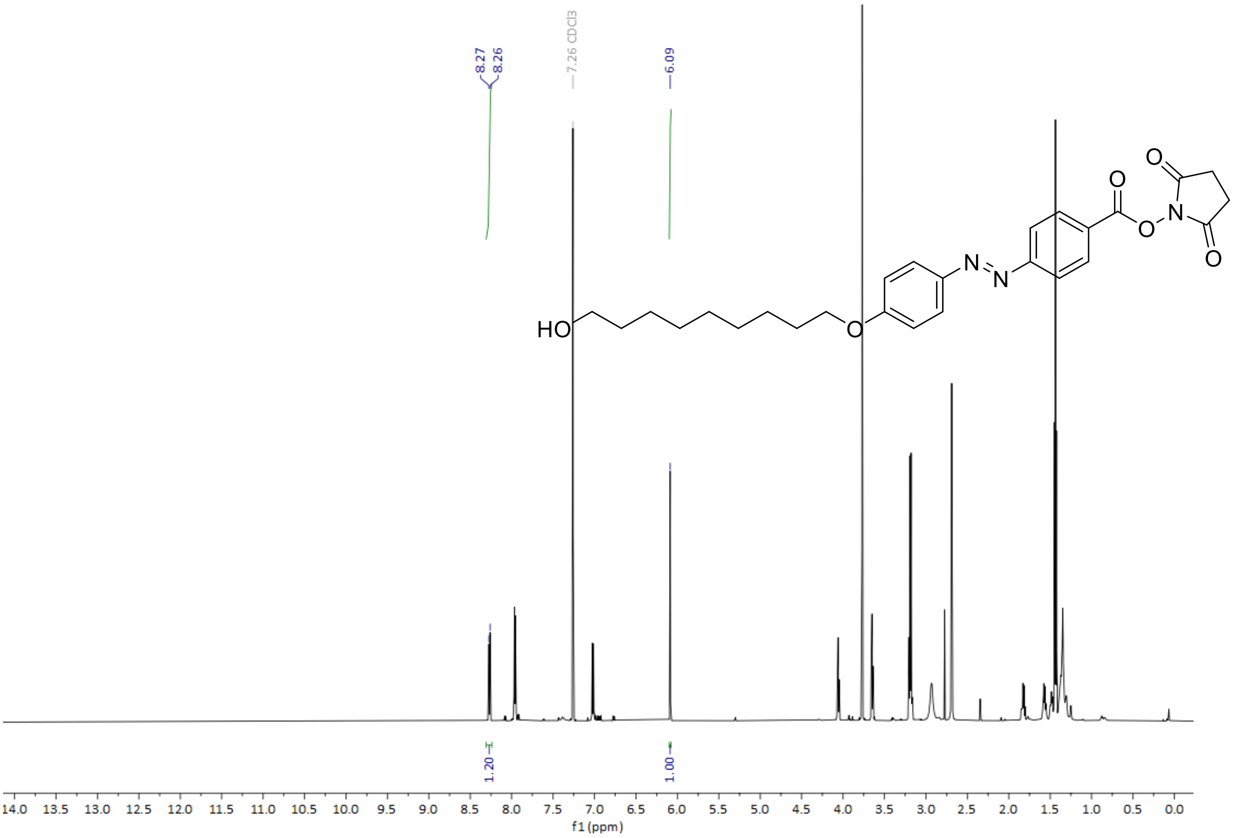

Figure S131: Crude ^{1}H NMR spectrum of **3h** in $CDCl_3$.

2,5-Dioxopyrrolidin-1-yl (*E*)-4-((2,6-dimethyl-4-(nonyloxy)phenyl)diazenyl)-3,5-dimethylbenzoate (**3j**)

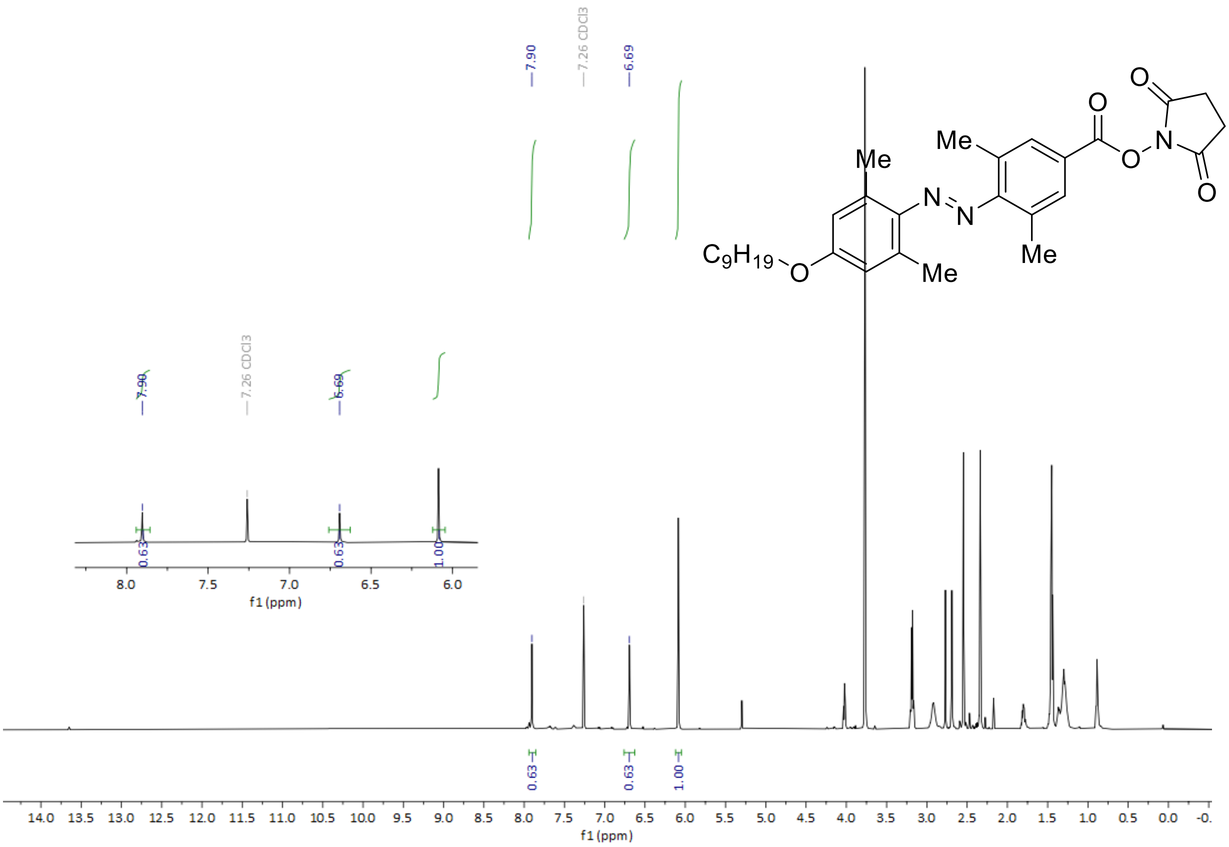

Figure S132: Crude ^1H NMR spectrum of **3j** in $CDCl_3$.

2,5-Dioxopyrrolidin-1-yl (*E*)-4-((2,7-bis(4-(hexyloxy)phenoxy)naphthalen-1-yl)diazenyl)-3,5-dimethylbenzoate (**3k**)

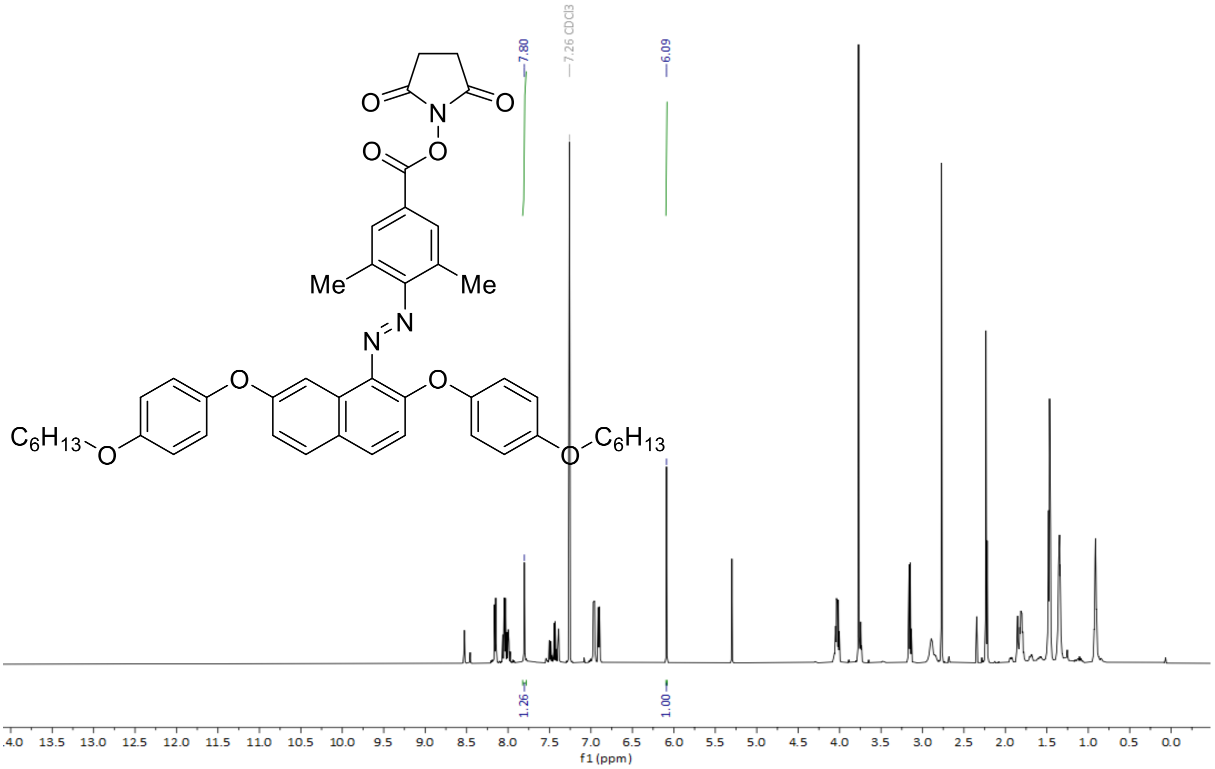

Figure S133: Crude ^1H NMR spectrum of **3k** in $CDCl_3$.

2,5-Dioxopyrrolidin-1-yl (*E*)-4-((4-bromophenyl)diazenyl)benzoate (**3l**)

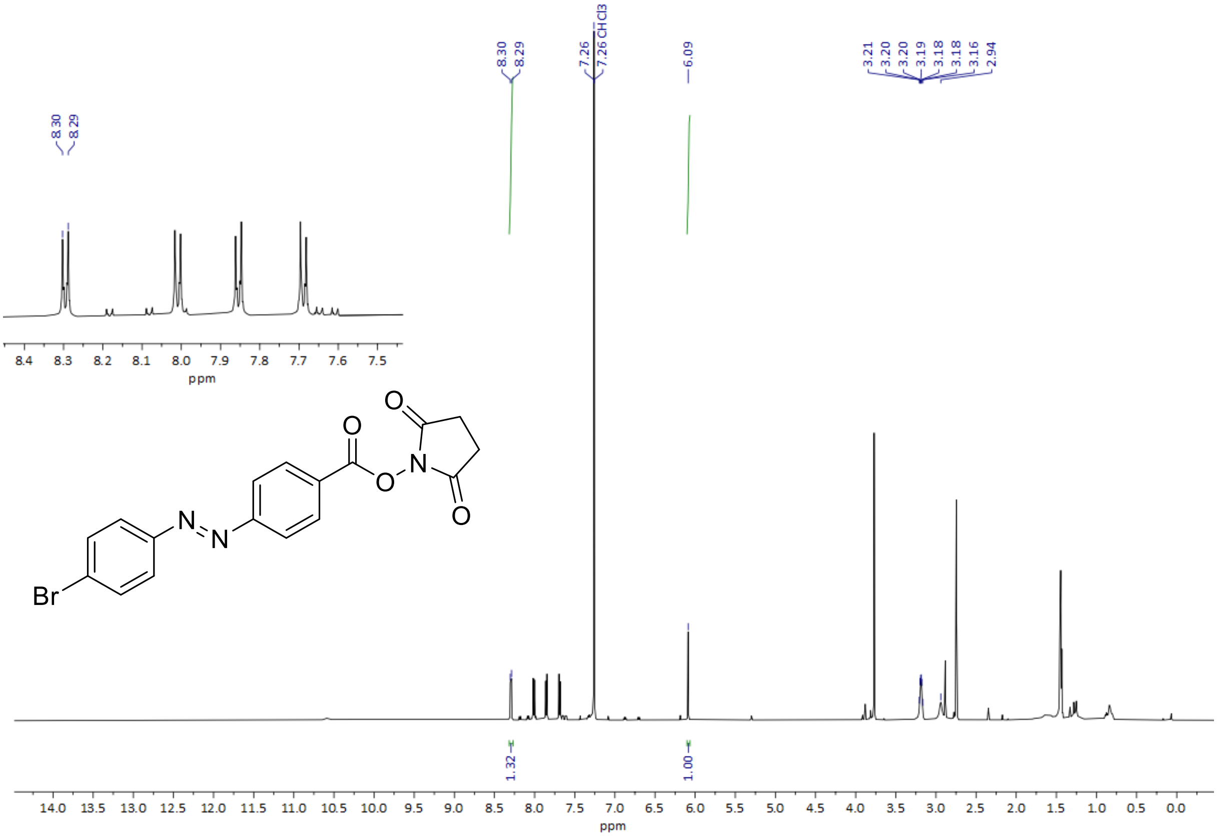

Figure S134: Crude ^{1}H NMR spectrum of **3l** in $CDCl_3$.

2,5-Dioxopyrrolidin-1-yl (*E*)-4-((3-bromophenyl)diazenyl)benzoate (**3m**)

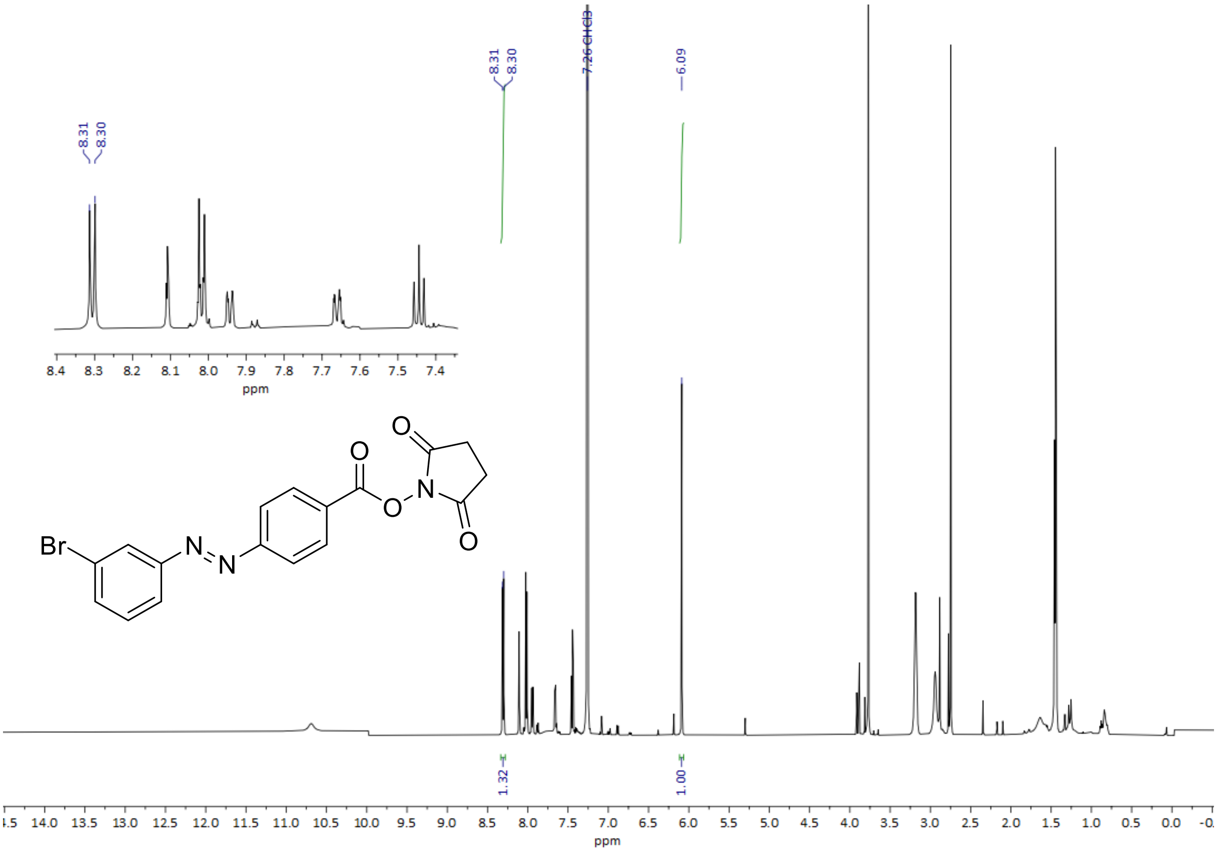

Figure S135: Crude ^{1}H NMR spectrum of **3m** in $CDCl_3$.

2,5-Dioxopyrrolidin-1-yl (*E*)-4-((4-bromophenyl)diazenyl)benzoate (**3n**)

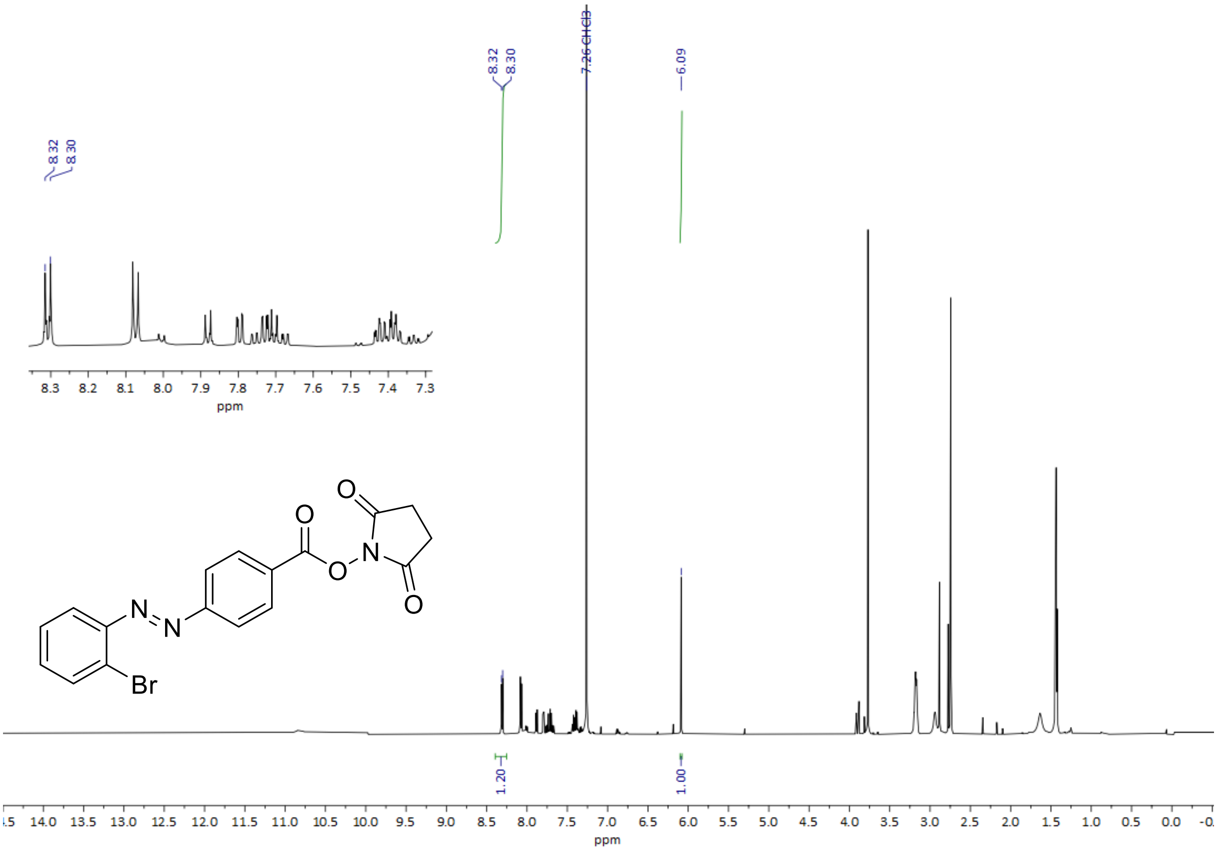

Figure S136: Crude ^{1}H NMR spectrum of **3n** in $CDCl_3$.

Bis(2,5-dioxopyrrolidin-1-yl) 4,4'-(diazene-1,2-diyl)(*E*)-dibenzoate (**5a**)

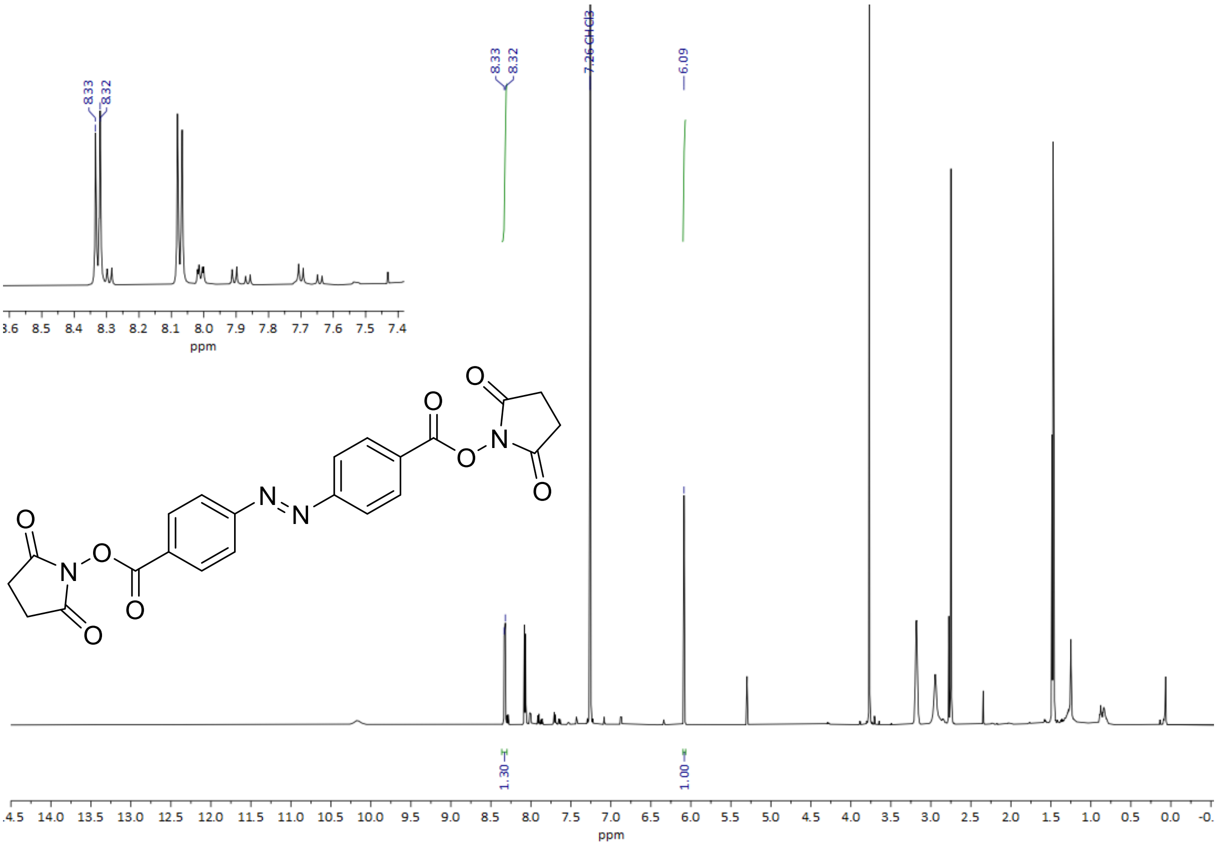

Figure S137: Crude ^{1}H NMR spectrum of **5a** in $CDCl_3$.

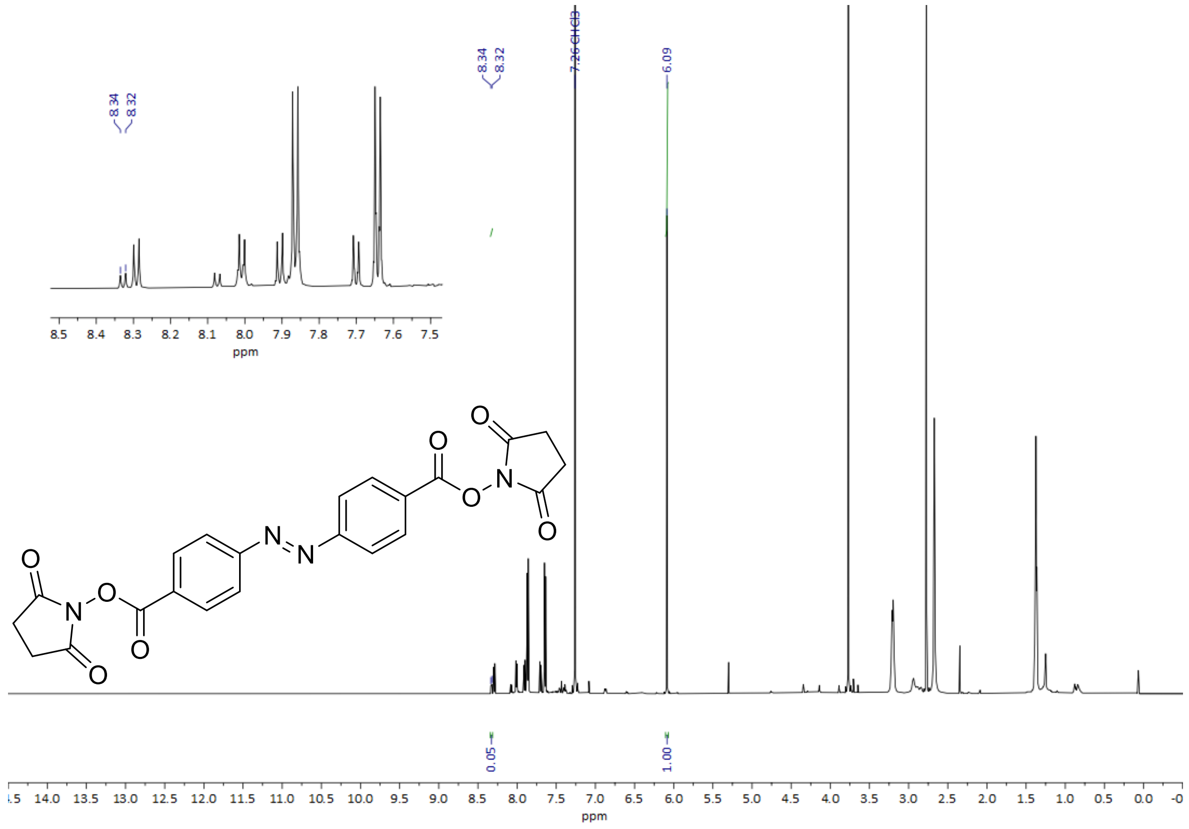

Figure S138: Crude ^{1}H NMR spectrum of **5a** in $CDCl_3$ using 1,1′-bis(diphenylphosphino)ferrocene as ligand.

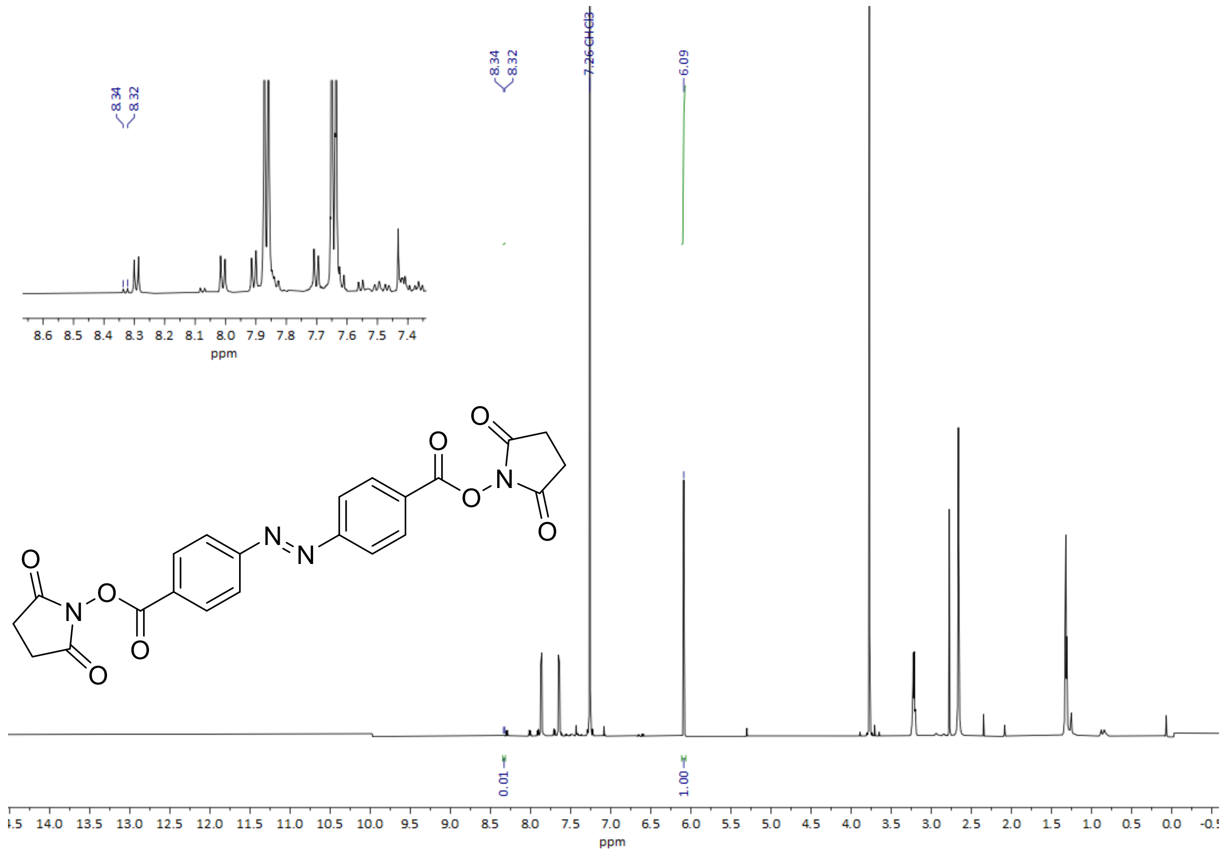

Figure S139: Crude ^{1}H NMR spectrum of **5a** in $CDCl_3$ using 2,2'-bis(diphenylphosphino)-1,1′-binaphthyl as ligand.

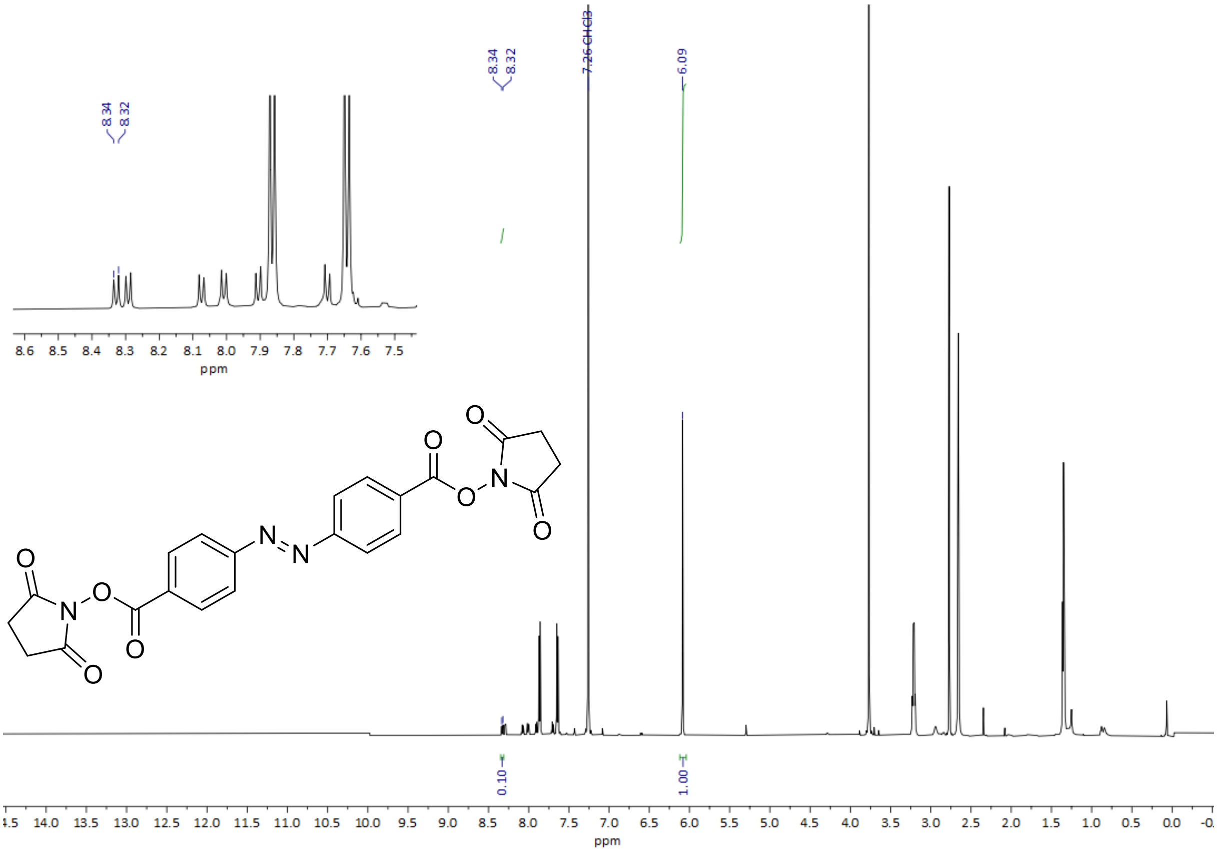

Figure S140: Crude ^{1}H NMR spectrum of **5a** in $CDCl_3$ using di-(1-adamantyl)-*n*-butylphosphine as ligand.

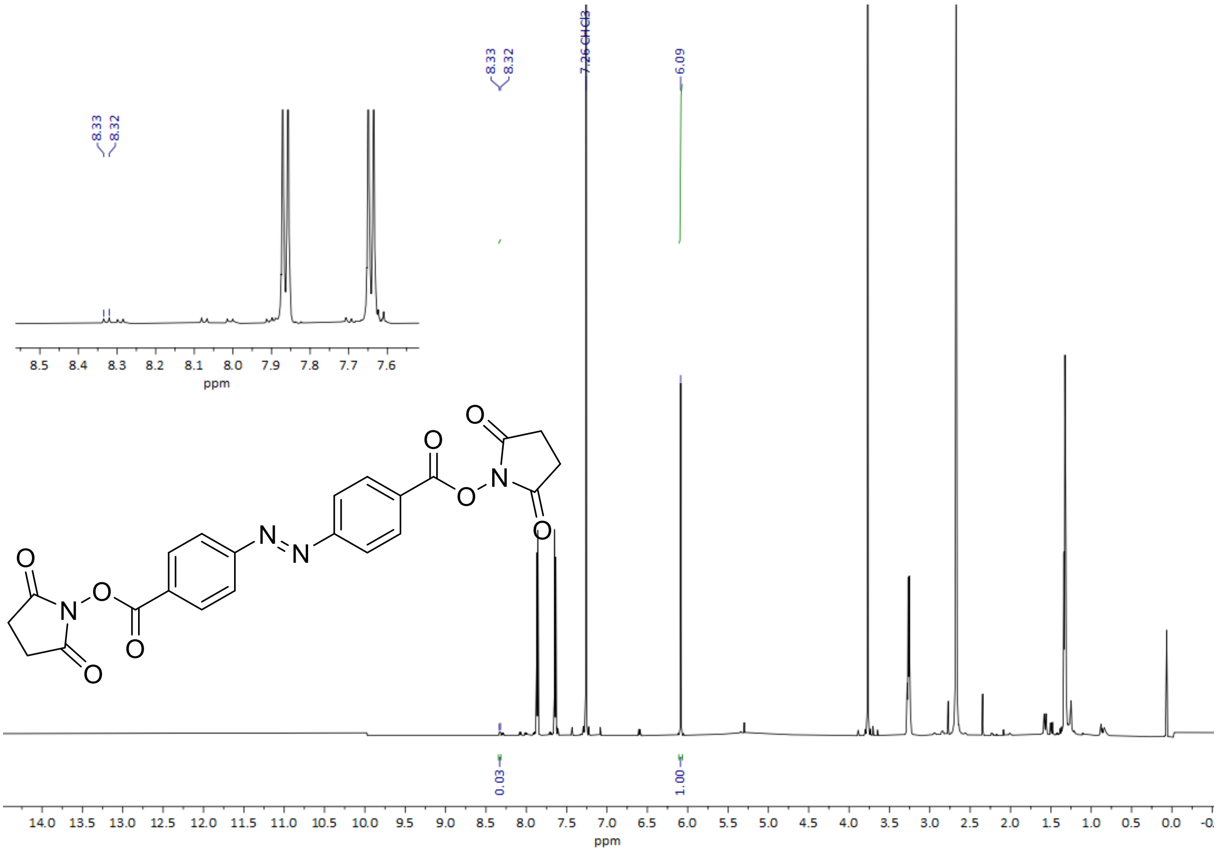

Figure S141: Crude ^{1}H NMR spectrum of **5a** in $CDCl_3$ using tri-*tert*-butylphosphine as ligand.

Bis(2,5-dioxopyrrolidin-1-yl) 4,4'-(diazene-1,2-diyl)(*E*)-bis(3,5-dimethylbenzoate) (**5b**)

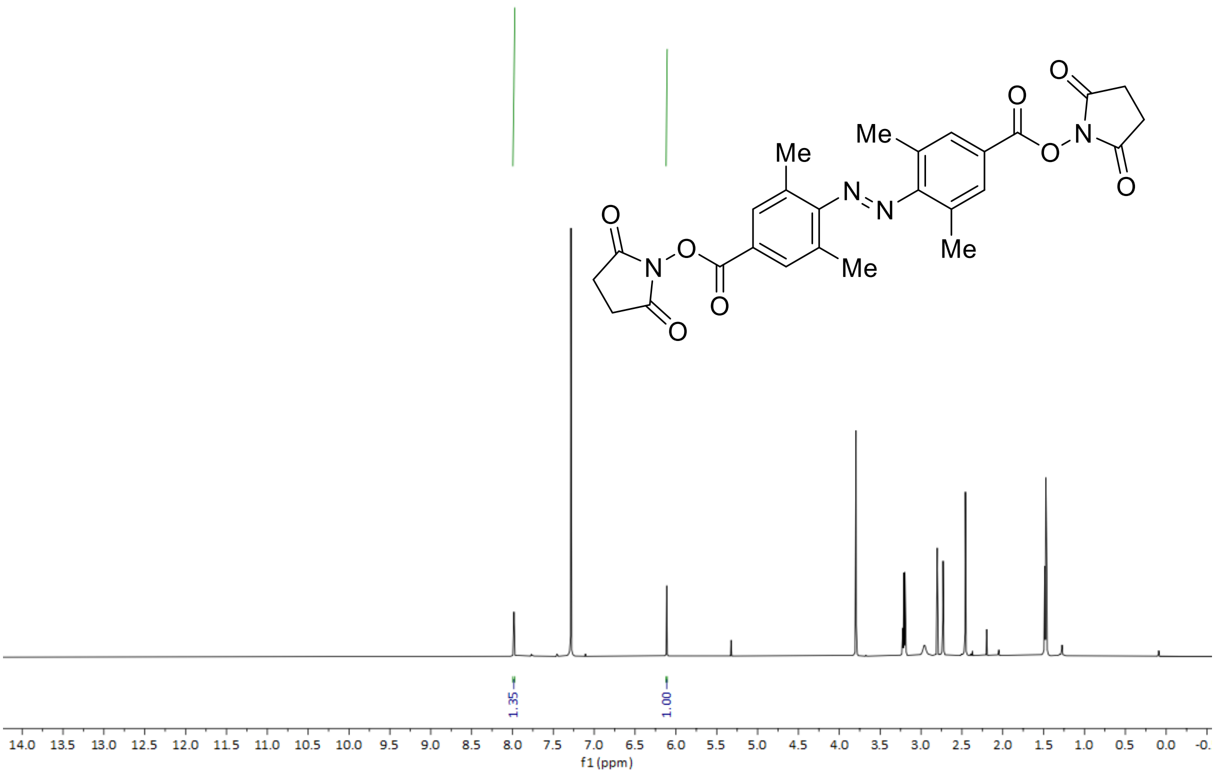

Figure S142: Crude ^{1}H NMR spectrum of **5b** in $CDCl_3$.
